# *syn*-Selective alkylarylation of terminal alkynes via the combination of photoredox and nickel catalysis

**DOI:** 10.1038/s41467-018-06904-9

**Published:** 2018-10-31

**Authors:** Lei Guo, Fan Song, Shengqing Zhu, Huan Li, Lingling Chu

**Affiliations:** State Key Laboratory for Modification of Chemical Fibers and Polymer Materials, College of Chemistry, Chemical Engineering and Biotechnology, Center for Advanced Low-Dimension Materials, Donghua University, Shanghai, 201620 China

## Abstract

Substituted alkenes are pivotal structural motifs found in pharmaceuticals and agrochemicals. Although numerous methods have been developed to construct substituted alkenes, a generally efficient, mild, catalytic platform for the conversion of alkynes to this highly functionalized scaffold via successive C–C bond forming steps remains in high demand. Here we describe an intermolecular, regio- and *syn*-stereoselective alkylarylation of terminal alkynes with tertiary alkyl oxalates via photoredox-Ni dual catalysis. This catalytic protocol, synergistically combining Ir/Ni-catalyzed alkyne difunctionalization with photoinduced alkene isomerization, affords trisubstituted alkenes with excellent efficiency and *syn*-stereoselectivity. The mild conditions tolerate many functional groups, allowing for a broad scope with respect to terminal alkynes, aryl bromides, and alkyl oxalates.

## Abstract (web summary)

Converting alkynes into alkenes with high stereoselectivity via two consecutive C-C bond forming steps is a desirable process, yet very challenging. Here, the authors describe a dual photoredox-nickel catalytic system for the regio- and *syn*-selective alkylarylation of terminal alkynes with alkyl oxalates and aryl bromides.

## Introduction

**Fig. 1:**
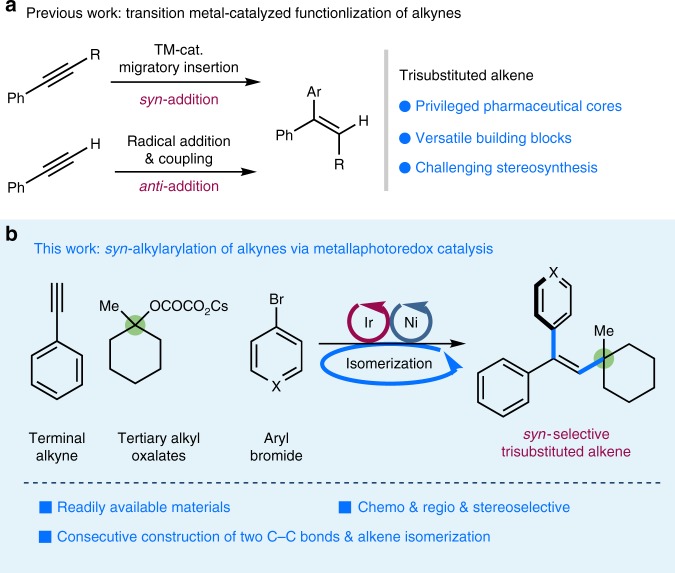
*syn*-Alkylarylation of terminal alkynes via the combination of photoredox and nickel catalysis. **a** Alkyne functionalizations via transition metal catalysis. **b** *syn*-Alkylarylation of alkynes via metallaphotoredox catalysis

Substituted alkenes are pivotal structural motifs found in pharmaceuticals, agrochemicals, and biologically active natural products^1–3^, as well as versatile synthetic building blocks in organic synthesis^4,5^. Consequently, the development of general protocols for the regio- and stereoselective synthesis of alkenes, particularly employing readily available and abundant precursors, is a long-standing goal of chemical synthesis^6,7^. Transition metal-catalyzed direct functionalization of alkynes is a powerful method to access substituted alkenes with stereoselectivity control^8–21^. Generally, two distinct strategies have been utilized: one is a catalytic generation of *syn*-substituted alkenyl metal species, via migratory insertion controlled by the stereoelectronic effect, followed by protonation or coupling to deliver trisubstituted alkenes with *syn*-stereoselectivity^8–28^; the other is transition metal-catalyzed radical addition/coupling reaction of alkynes, typically favoring *anti*-addition which is dominated by steric factors of vinyl radicals in the coupling step (Fig. 1a)^29–37^. Although numerous methods have been developed toward addressing the challenge of regio- and stereo-selectively forming trisubstituted alkenes, a generally efficient, mild, catalytic platform for the conversion of alkynes to this highly functionalized scaffold via successive C–C bond forming steps would be highly demanding and represent a valuable advance in synthetic methodology.

Over the last decade, visible light photocatalysis have emerged as a powerful platform in organic synthesis by activating organic molecules through either single-electron transfer or energy transfer^38–43^. Particularly, the ability of the photoredox catalyst to modulate the oxidation state of organometallic species has enabled the efficient construction of challenging C–C bonds^44–49^. Recently, this solar-energy-driven catalytic technology has been utilized to facilitate contra-thermodynamic *E* → *Z* isomerization of olefins through an energy-transfer manifold, enabling the facile synthesis of *Z*-olefins^50–56^. We recently questioned whether metallaphotoredox catalysis could serve as an alternative platform to access trisubstituted alkenes with high control over stereoselectivity^57–59^. Specifically, a catalytic protocol including three sequential events, (i) alkyl radical addition to the C≡C bond^29^, (ii) cross-coupling of the resulting alkenyl radical with nickel complex, and (iii) photochemical *E* → *Z* isomerization of olefins, would deliver the stereodefined trisubstituted alkenes. We envisioned that the unique metallaphotoredox manifold would enable the generation of alkyl radicals from readily available feedstocks^60^ such as alcohols^61^, and more importantly could be leveraged to enrich the stereoselectivity of alkyne addition reactions. Herein, we demonstrate the first example of alkylarylation of terminal alkynes employing simple tertiary alcohol derivatives and aryl halides through the synergistic merger of photoredox and nickel catalysis^62,63^, furnishing a wide array of trisubstituted alkenes with *syn*-stereoselectivity under mild conditions (Fig. 1b). This photoredox protocol provides complementary reactivity and stereoselectivity to a previous nickel system with alkyl halides as radical precursors, which affords the trisubstituted alkenes with *anti*-stereoselectivity at elevated temperature (80–120 °C)^34^.

## Results

### Design plan

**Fig. 2:**
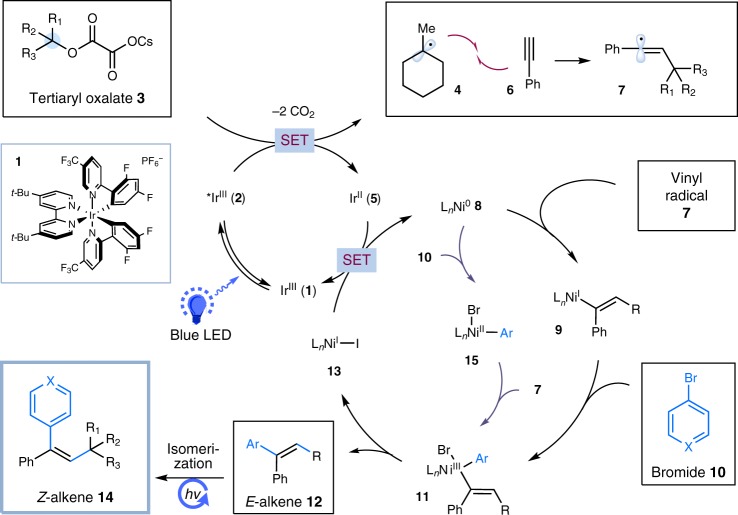
Proposed mechanism. Two possible reaction pathways proposed on the basis of previous literature

Inspired by Overman and MacMillan’s oxalate half-ester chemistry^64^, we chose tertiary alkyl oxalates as the alkyl precursors for our proposed metallaphotoredox alkyne chemistry. Tertiary oxalate salts are bench stable, and can be easily prepared from corresponding, abundant tertiary alcohols. As depicted in Fig. 2, we envisioned that a single-electron oxidation of tertiary alkyl oxalate **3** (*E*_1/2_ = + 1.28 V vs SCE in CH_3_CN for *tert*-BuOCOCO_2_Cs)^64^ by photoexcited Ir[dF(CF_3_)ppy]_2_(dtbbpy)(PF_6_) **1** (*E*_1/2_[Ir^*III^/Ir^II^ = + 1.21 V vs SCE in CH_3_CN]^65^ should generate alkyl radical **4** upon loss of two molecules of CO_2_ as well as reduced Ir(II) species **5**. Alkyl radical **4** is expected to undergo regioselective addition to terminal alkyne **6** to produce linearized alkenyl radical **7** due to resonance stabilization effect^29^. An *anti*-addition of the high-energy alkenyl radial **7** and Ni(0) **8** is expected to deliver the (*E*)-alkenyl-Ni(I) species **9**^66^. Subsequent oxidative addition of (*E*)-alkenyl-Ni(I) **9** with aryl bromide **10** would afford (*E*)-alkenyl-Ni(III) complex **11**^34,67^, which undergoes a facile reductive elimination to produce substituted alkene **12** with concomitant generation of Ni(I) complex **13**. Single-electron transfer between Ir(II) **5** {*E*_1/2_[Ir^III^/Ir^II^ = −1.37 V vs SCE in CH_3_CN]}^65^ and Ni(I) **13** (*E*_1/2_[Ni^II^/Ni^0^ = −1.2 V vs SCE in DMF]^68^ would regenerate ground-state Ir(III) **1** and Ni(0) to close the two catalytic cycles. Given the polarity of **12**, at this juncture, we hypothesized that a *E* → *Z* isomerization of **12** would be possible through a photoinduced energy transfer manifold^50–56^, delivering the desired alkene **14** with *syn*-stereoselectivity. Alternatively, another catalytic pathway involving oxidative addition of Ni(0) with aryl bromide **10**^62,63,69^, followed by trapping of the nucleophilic vinyl radical **7** by aryl-Ni(II) **15** to furnish the key Ni(III) intermediate **11**, is also plausible.

### Optimization study

We examined the feasibility of this proposed metallaphotoredox alkyne protocol by employing 4-*tert*-butylphenylacetylene **16** and 4-bromobenzaldehyde **17** as standard substrates. As shown in Table 1, upon irradiation by a 90 W blue LED of a solution of alkyne **16**, bromide **17**, and cesium oxalate **18** derived from 1-methyl-1-cyclohexanol in the presence of catalytic amounts of Ir[dF(CF_3_)ppy]_2_(dtbbpy)PF_6_ **1**, NiCl_2_•glyme, 4,4′-di-*tert*-butyl-2,2′-dipyridyl (dtbbpy) in DMSO, 81% yield of the desired trisubstituted alkene product **19** was obtained with excellent chemo-, regio-, and *syn*-selectivity (*Z*/*E* = 96:4) (entry 1). The structurally similar photocatalyst Ir[dF(CF_3_)ppy]_2_(phen)PF_6_ also promoted this transformation with moderate efficiency and excellent *syn*-selectivity (entry 2). Switching to other commonly employed photocatalysts, such as Ru(bpy)_3_(PF_6_)_2_ and 4CzlPN (2,4,5,6-tetra(9*H*-carbazol-9-yl)isophthalonitrile), resulted in a dramatic decrease in efficiency (entries 3–4). The reaction proceeded with moderate to good efficiency in the presence of NiCl_2_(PPh_3_)_2_ or precatalyst NiCl_2_(Py)_4_ (entries 5–6). The choice of solvent demonstrated a dramatic effect on the reaction efficiency, with DMSO proving to be optimal (entries 7–10). Interestingly, stereoselectivity of the alkene product appeared to be solvent independent (entries 7–10). Finally, control experiments demonstrated that light, photocatalyst, nickel catalyst, and ligand are all essential for the desired transformation to proceed (entries 11–14) (for additional control experiments, see Supplementary Tables 1, 2).

**Table 1:** Optimization of reaction conditions.^a^

^a^Reaction conditions: photocatalyst (3 mol%), NiCl_2_•glyme (20 mol%), dtbbpy (20 mol%), alkyne (0.1 mmol), oxalate (1.5 equiv.), aryl bromide (2.0 equiv.), DMSO [0.05 M], 90 W blue LED, 36 °C, 18 h. Yields determined by ^1^H NMR with an internal standard, and the ratio of the two isomers was determined by ^1^H NMR analysis of the crude reaction mixture. dtbbpy = 4,4′-di-*tert*-butyl-2,2′-dipyridyl; 4CzlPN = 2,4,5,6-tetra(9*H*-carbazol-9-yl)isophthalonitrile

### Substrate scope

**Fig. 3:**
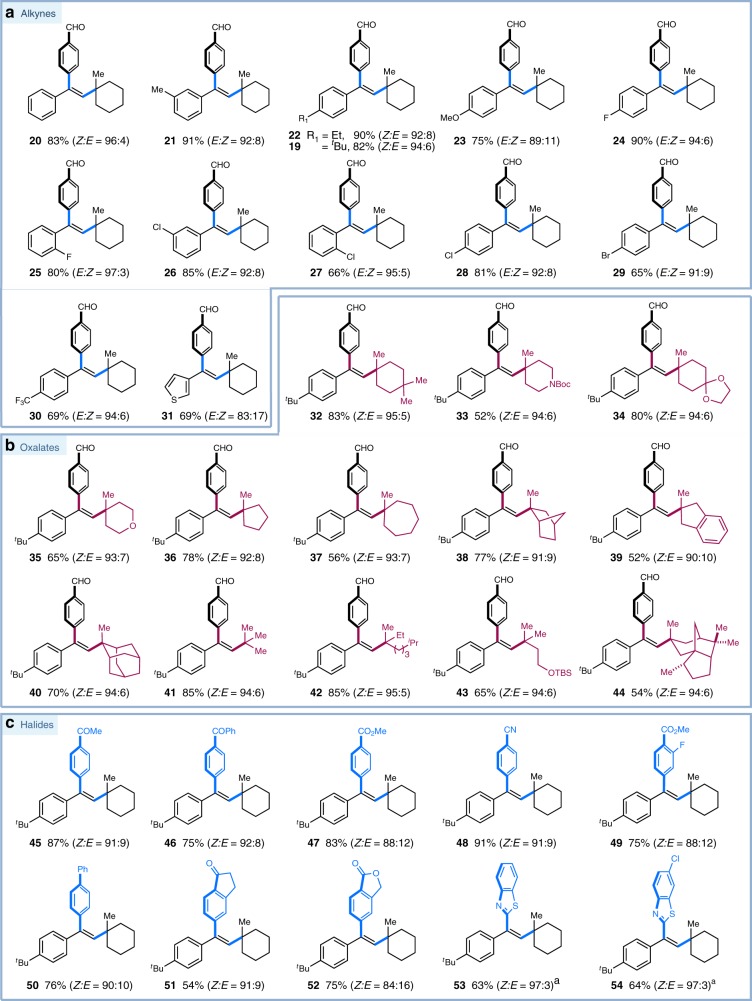
Substrate scope. **a** Scope of alkynes. **b** Scope of cesium oxalates. **c** Scope of aryl halides. Reaction conditions: Ir-**1** (3 mol%), NiCl_2_•glyme (20 mol%), dtbbpy (20 mol%), alkyne (0.1 mmol), oxalate (1.5 equiv.), bromide (2.0 equiv.), DMSO [0.05 M], 90 W blue LED, 36 °C, 18 h. All cited yields are isolated yields. The ratios of the two isomers were determined by ^1^H NMR analysis of the crude reaction mixtures. ^a^Heteroaryl chloride was employed. *dtbbpy* = 4,4′-di-*tert*-butyl-2,2′-dipyridyl

With optimal conditions in hand, we explored the generality of this metallaphotoredox protocol with respect to the alkyne fragment. As depicted in Fig. 3a, terminal arylalkynes bearing electron-neutral, -donating, and -withdrawing substituents proceeded smoothly under the optimal conditions, furnishing the corresponding trisubstituted alkenes with high to excellent yields and stereoselectivity (products **19**−**30**, 65−90% yields, *>*95:5 *syn*-selectivity). Notably, halides (F, Cl, Br) on the aryl ring of alkynes remained untouched, offering valuable handles for further manipulations (product **24**−**29**, 65−90% yields, >91:9 *syn*-selectivity). Moreover, ortho substituents on the aryl ring had little effect to the reaction efficiency and selectivity (products **25** and **27**, 80% and 66% yield, >95:5 *syn*-selectivity, respectively). A slight erosion of yield and selectivity was observed when 3-ethynylthiophene was subjected to this multicomponent system (products **31**, 69% yield, 83:17 *syn*-selectivity). However, internal alkynes are not compatible in this reaction protocol, a result we attribute to increased steric hindrance.

Next, we evaluated the scope of cesium tertiary alkyl oxalates in this protocol. As shown in Fig. 3b, a variety of tertiary cesium oxalates, readily prepared from the corresponding tertiary alcohols, can be successfully employed with high levels of efficiency and stereoselectivity. Cyclic oxalates, derived from cyclohexanols, cyclopentanols, and cycloheptanols, underwent the desired addition/coupling smoothly, yielding the (*Z*)-selective trisubstituted alkenes with high efficiency (products **32**−**40** and **44**, 52−83% yields, *Z/E* up to 95:5). Heterocycles, in the form of tetrahydropyran and piperidine, were also viable substrates (products **33** and **35**, 52% and 65% yields, *Z/E* > 93:7). A number of polycyclic oxalates could be effectively employed without loss in yield and selectivity (products **34**, **38**−**40**, and **44**, 49−84% yields, *Z*/*E* up to 94:6). Moreover, this photoredox protocol could further be applied to acyclic tertiary oxalates, furnishing the desired alkenes in good yields and excellent stereoselectivity favoring *syn*-addition (products **41**−**43**, 65−85% yields, *Z/E* > 94:6). Notably, natural-product-derived substrates, such as cedrol oxalate, proved successful, indicating the potential utility of this mild protocol with complex molecules (products **44**, 54% yield, *Z/E* = 94:6).

Finally, we turned our attention to the scope of aryl bromides that can participate in this catalytic protocol. As revealed in Fig. 3c, a variety of electron-deficient aryl bromides can be readily employed with high efficiency and moderate-to-high stereoselectivity (products **45**−**54**, 54−91% yields, *Z/E* up to 97:3). Many valuable functional groups, including ketones, esters, nitrile, and lactones were found to be well tolerated under the mild conditions (products **45**−**52**, 54−91% yields, *Z/E* up to 92:8). Gratifyingly, benzothiazole-derived heteroaromatic chlorides could be efficiently employed in this synergistic protocol without any loss in stereoselectivity, albeit with a slight decrease in yields (products **53** and **54**, 63% and 64% yields, *Z/E* = 97:3, respectively). At this stage in our studies on this metallaphotoredox protocol, the scope of aryl halides is currently limited to electron-poor and electron-neutral system, in which a conjugated substituent at the para position is crucial to achieve excellent *syn*-stereoselectivity control (see Supplementary Figs. 13, 14).

**Fig. 4:**
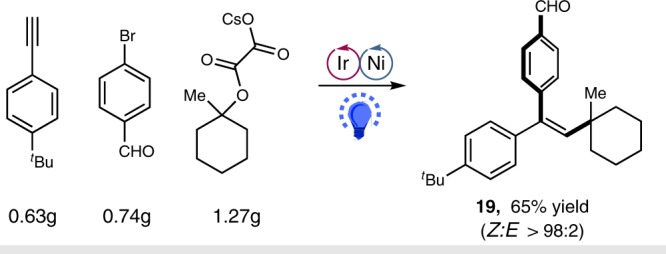
Reaction on large scale. Reaction condition: Ir-**1** (3 mol%), NiCl_2_•glyme (20 mol%), dtbbpy (20 mol%), alkyne (4 mmol), oxalate (1.5 equiv.), bromide (2.0 equiv.), DMSO [0.05 M], 90 W blue LED, 36 °C, 18 h. Isolated yield. The ratio of the two isomers were determined by ^1^H NMR analysis of the crude reaction mixture. dtbbpy = 4,4′-di-*tert*-butyl-2,2′-dipyridyl

To highlight the synthetic utility of this metallaphotoredox difunctionalization manifold, a gram-scale reaction of alkyne **16** was performed. The reaction proceeded smoothly, affording the desired alkylarylation product **19** in 65% yield with excellent stereoselectivity (*Z/E* > 98:2) (Fig. 4).

### Mechanistic studies

**Fig. 5:**
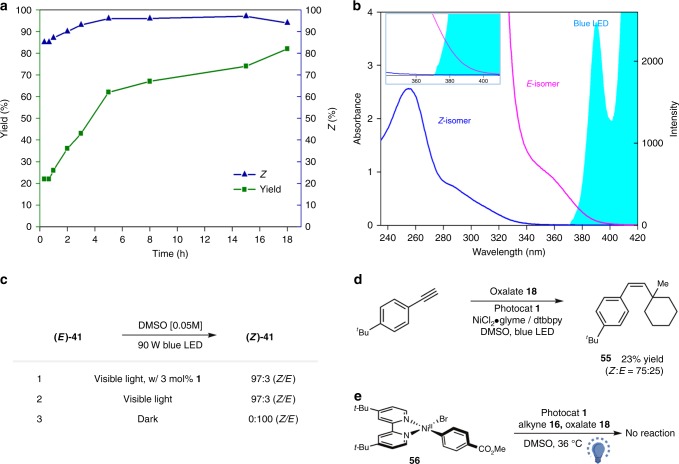
Mechanistic studies. **a** Time course studies; **b** UV–Vis absorption spectrometry; **c** Isomerization experiments; **d** Reaction of alkyne with oxalate; **e** Stoichiometric reaction of isolated Ni(II) complex. *dtbbpy* = 4,4′-di-*tert*-butyl-2,2′-dipyridyl

To probe the reaction pathway of this metallaphotoredox three-component coupling protocol, we conducted a series of preliminary mechanistic experiments. Time course studies showed that yields steadily increased overtime, while high stereoselectivity was observed at the early stage, suggesting that stereoselectivity of this transformation might be catalyst-independent (Fig. 5a). To probe the stereo-enrichment process, (*E*)-alkene **41** was prepared and subjected to the photoinduced system (Fig. 5c). In the presence or absence of photocatalyst **1**, (*E*)-**41** underwent the isomerization to yield (*Z*)-**41** with excellent efficiency, indicating that photocatalyst does not actually engage in the isomerization event (Fig. 5c). As expected, no isomerization of **41** was observed in the absence of visible light (Fig. 5c). On the basis of these results, we expected that trisubstituted alkene itself might act as a photosensitizer for this photochemical isomerization. This hypothesis was further confirmed by UV–Vis absorption spectrometry of (*E*)-**41**, which exhibited considerable overlap with the blue LED spectrum (Fig. 5b). Particularly, the (*E*)-isomer showed strong bathochromic shift relative to the (*Z*)-isomer (Fig. 5b). Predictably, deconjugation of the π-system in the (*Z*)-isomer product secures high levels of stereocontrol in this contra-thermodynamic, photoinduced *E* → *Z* isomerization (see Supplementary Fig. 3 for density functional theory (DFT)-based conformational analysis). Furthermore, reaction of oxalate **18** with alkyne **16** in the absence of aryl bromide afforded the alkene product **55** in 23% yield (Fig. 5d), providing supporting for the addition of alkyl radical to alkyne step shown in Fig. 2. Nevertheless, only a trace amount of *tert*-alkyl-aryl coupling product was observed in the reaction of oxalate **18** with aryl bromide **17** (see Supplementary Fig. 10). These results suggest that, under these metallaphotoredox conditions, tertiary alkyl radical would be more prone to undergo radical addition to alkyne, as opposed to capture by nickel species, probably due to steric hindrance^70^. Finally, we have prepared Ni(II) oxidative addition complex^69,71^ **56** to evaluate an alternative pathway involving radical capture by aryl-Ni(II) species **15** (Fig. 2). Irradiation with a 90 W blue LED of a solution of isolated aryl-Ni^II^-Br **56**, alkyne **16**, and oxalate **18** in the presence of stoichiometric amount of photocatalyst **1** led to no formation of the desired trisubstituted alkene product (Fig. 5e). The major byproduct was biaryl, which could be formed via homo-coupling from disproportionation of Ni(II) complex **56** (see Supplementary Fig. 12 and Supplementary Table 4). Therefore, we expected that a catalytic Ni^0/I/III^ pathway, proceeding via capture of alkenyl radical by Ni(0), followed by oxidative addition and then reductive elimination, could be operative in this metallaphotoredox manifold (Fig. 2).

## Discussion

In conclusion, we have developed a generic protocol for the intermolecular, regioselective, *syn*-alkylarylation of terminal alkynes with tertiary alkyl oxalates through a synergistic merger of photoredox and nickel catalysis. A one-pot, three-step sequence, involving radical addition, transition-metal-based coupling, and alkene isomerization, proceeds with high efficiency under the light-induced mild conditions. This manifold forges two vicinal C–C bonds, yielding a variety of trisubstituted alkenes with excellent regioselectivity and *syn*-stereoselectivity. We expect that the operational simplicity and generality of this methodology and readily availability of the starting materials will allow it to enjoy extensive application in the area of organic chemistry.

## Methods

### General procedure for the *syn*-selective alkylarylataion reaction

To a flame dried 8 mL reaction vial was charged with Ir[dF(CF_3_)ppy]_2_(dtbbpy)(PF_6_) (0.003 mmol, 3 mol%), NiCl_2_•DME (0.02 mmol, 20 mol%), 4,4′-di-*tert*-butyl-2,2′-dipyridyl (0.02 mmol, 20 mol%), aryl bromide (0.2 mmol, 2.0 equiv.), and cesium alkyl oxalate (0.15 mmol, 1.5 equiv.). The vial was capped. After evacuated and backfilled nitrogen three times, DMSO [0.05 M] was added via a syringe, followed by the addition of terminal alkyne (0.1 mmol, 1.0 equiv.). The reaction mixture was irritated with a 90 W blue LED, with cooling from a fan (36 °C). After 18 h, the reaction was quenched with H_2_O, extracted with ethyl acetate. The combined organic layers were dried with MgSO_4_, filtered, and concentrated in vacuo. The crude material was purified by flash chromatography to afford the products. See Supplementary Methods for further experimental details.

## Electronic supplementary material

Supplementary Data 1

Supplementary Information

Description of Additional Supplementary Files

## Data Availability

The authors declare that all the data supporting the findings of this work are available within the article and its Supplementary Information files, or from the corresponding author upon request.
