# Supplementary material for: syn-Selective alkylarylation of terminal alkynes via the combination of photoredox and nickel catalysis
Source: Nat Commun. 2018 Oct 31;9:4543. doi: 10.1038/s41467-018-06904-9 (PMC6208420; doi:10.1038/s41467-018-06904-9)
Supplement: Supplementary file 1 — Supplementary Data 1 [file 41467_2018_6904_MOESM1_ESM.docx]

**Cartesian coordinates and energies**

***E*-19**

SCF Done: E(RB3LYP) = -1085.31266854 a.u.

Zero-point correction = 0.515792 Hartree/Particle

Sum of electronic and thermal Free Energies = -1084.854168 a.u.

-----------------------------------------------------------------------------

C,0,-5.666596532,1.8370263014,1.2981746076

C,0,-5.6366185621,3.2030033819,0.5937286807

C,0,-4.3912609897,3.355581097,-0.2902957148

C,0,-4.2810025361,2.2018669195,-1.2966592618

C,0,-4.3213321921,0.8365950243,-0.5931762437

C,0,-5.5671588037,0.6217717231,0.3242890899

H,0,-4.4091055589,4.3189377062,-0.8135734549

H,0,-6.5386980411,3.3286933561,-0.0188814907

H,0,-5.666293044,3.9988159531,1.3477887392

H,0,-4.8253720362,1.7870518353,2.0035597646

H,0,-6.5786389528,1.7402734688,1.8979142973

H,0,-5.0925204166,2.2775958798,-2.0313688145

H,0,-3.3481813414,2.2837821331,-1.8675098199

H,0,-4.2726430935,0.0260456518,-1.3313627715

H,0,-3.4169572826,0.7464423708,0.0247377734

H,0,-3.4960887722,3.3633645585,0.3481767312

C,0,-6.8303100976,0.5090541671,-0.5527784513

H,0,-6.8053021514,-0.4082731476,-1.1490187851

H,0,-7.7447037425,0.4950538847,0.0414150986

H,0,-6.9020034891,1.3512224669,-1.2454464417

C,0,-5.2820708044,-0.626880549,1.1491967921

C,0,-6.0603383852,-1.622320789,1.6287433464

H,0,-4.2251528236,-0.7122554082,1.4025725249

C,0,-7.5545943241,-1.6749298964,1.5527766047

C,0,-8.2033143373,-2.656748103,0.794625388

C,0,-8.3548928674,-0.7884453298,2.288774747

C,0,-9.5958523834,-2.7256161664,0.7411335879

H,0,-7.6117019653,-3.3716861335,0.2296121444

C,0,-9.7448837248,-0.867462991,2.2398864496

H,0,-7.8804270907,-0.0348612163,2.9104379856

C,0,-10.4031191531,-1.8329684935,1.4602127707

H,0,-10.0480535698,-3.4956195844,0.1268465861

H,0,-10.3239349062,-0.1602652259,2.826553626

C,0,-5.4145983192,-2.7672485778,2.3393542119

C,0,-6.0503455092,-3.3820449311,3.4387778929

C,0,-4.1633071769,-3.2686449105,1.933276194

C,0,-5.4465423413,-4.4262670738,4.1236633065

H,0,-7.0227101723,-3.0209943355,3.7566048981

C,0,-3.5576872776,-4.3164061026,2.6178429242

H,0,-3.6797491115,-2.8455980621,1.0589417037

C,0,-4.1899711705,-4.9042983208,3.7208690355

H,0,-5.9276079347,-4.8914095123,4.9782772877

H,0,-2.5929397332,-4.695626852,2.287945904

C,0,-3.5422350877,-6.0196534835,4.4394507154

O,0,-4.0137617637,-6.5931225866,5.4044425203

H,0,-2.5510021909,-6.3182942575,4.0292139937

C,0,-11.9415440199,-1.881354836,1.4299093645

C,0,-12.4879372213,-0.5319956854,0.9058784682

C,0,-12.4736605518,-3.0017841005,0.5167945727

C,0,-12.4768348906,-2.1294398223,2.860239616

H,0,-12.1807906343,0.3056153917,1.5389273344

H,0,-12.1298617929,-0.3321923812,-0.1093014354

H,0,-13.5833341321,-0.5475967415,0.8830340976

H,0,-12.1481842029,-3.9915371716,0.8531225734

H,0,-13.5682524671,-2.9949778778,0.5252401002

H,0,-12.1513569965,-2.8695498644,-0.5212018111

H,0,-13.5721460763,-2.1603202116,2.857087208

H,0,-12.1113916809,-3.082837934,3.2553117615

H,0,-12.168691414,-1.3410288346,3.5531691259

-----------------------------------------------------------------------------

***Z*-19**

SCF Done: E(RB3LYP) = -1085.31192645 a.u.

Zero-point correction = 0.515679 Hartree/Particle

Sum of electronic and thermal Free Energies = -1084.853637a.u.

-----------------------------------------------------------------------------

C,0,-2.7815841933,-1.8026382144,-0.0789794024

C,0,-1.3289675033,-1.4444147705,-0.4319988788

C,0,-1.1630449725,0.0615426031,-0.6778071062

C,0,-1.6685843063,0.8776188341,0.5198191253

C,0,-3.1168368127,0.511279901,0.8793443487

C,0,-3.350590792,-1.0112419735,1.1392388967

H,0,-0.1141798913,0.301540426,-0.8886578693

H,0,-0.6576680326,-1.7598043703,0.3772330521

H,0,-1.0228497875,-2.009811618,-1.3205096651

H,0,-3.4161739499,-1.5983350138,-0.9525784204

H,0,-2.8684098401,-2.87708909,0.1200640509

H,0,-1.0074499342,0.7135271985,1.3801224591

H,0,-1.6145648034,1.9503130304,0.2981593442

H,0,-3.4479836537,1.0818685082,1.7562039422

H,0,-3.762680716,0.8227613023,0.0466205253

H,0,-1.7345216525,0.3444805376,-1.573575905

C,0,-2.6379459179,-1.4163378626,2.4449935362

H,0,-3.1209817844,-0.9504929789,3.3094852456

H,0,-2.6436015336,-2.495967392,2.6008524241

H,0,-1.5936821534,-1.0946496696,2.4383681228

C,0,-4.8606932132,-1.2136103955,1.1745409945

C,0,-5.6809078627,-2.0105589178,1.893315321

H,0,-5.3730226553,-0.5642445805,0.4644311578

C,0,-7.1620632577,-1.86163884,1.7520040374

C,0,-8.0145696349,-2.9740930232,1.800226362

C,0,-7.7618456883,-0.6035187117,1.5662547587

C,0,-9.3939015217,-2.8407708865,1.6392983939

H,0,-7.5955104085,-3.9637237828,1.9536421361

C,0,-9.1372152602,-0.4766312298,1.4070625921

H,0,-7.1420014498,0.2879032274,1.570641232

C,0,-9.9932436985,-1.5910702309,1.4353996291

H,0,-10.0014317336,-3.737761283,1.6747391034

H,0,-9.5511349948,0.5186182023,1.2728666172

C,0,-5.2410267497,-3.0858966581,2.8364439277

C,0,-5.5202127423,-2.9830847831,4.2129886243

C,0,-4.6087465876,-4.2466036271,2.3633954082

C,0,-5.1423062501,-3.9870745485,5.0929519616

H,0,-6.0309785569,-2.0990984762,4.5826933462

C,0,-4.237776997,-5.2614589665,3.2433906218

H,0,-4.4170173451,-4.348592934,1.3000368837

C,0,-4.4951023312,-5.1374015714,4.6135445354

H,0,-5.3384823242,-3.9095034826,6.1576335376

H,0,-3.7480050722,-6.1572285625,2.8679894847

C,0,-4.0939378737,-6.2153356499,5.5423278871

O,0,-4.2742973454,-6.1972811141,6.7455900423

H,0,-3.5945660094,-7.0825269348,5.0543662917

C,0,-11.5106065745,-1.4040397175,1.2596063897

C,0,-11.7915255718,-0.7457191367,-0.1121396041

C,0,-12.2749077451,-2.7400265705,1.3165084493

C,0,-12.0515465885,-0.4907255914,2.385372166

H,0,-11.30764356,0.2312769715,-0.201719888

H,0,-11.426664064,-1.3750646325,-0.9303713547

H,0,-12.8684521924,-0.597990668,-0.2509481031

H,0,-12.1436280954,-3.2441780536,2.2794422944

H,0,-13.3461444319,-2.5579517276,1.183455859

H,0,-11.9561747543,-3.4258710775,0.5248054385

H,0,-13.1311555001,-0.3422471683,2.270144915

H,0,-11.8728955457,-0.9352789681,3.3697899593

H,0,-11.5773378917,0.4950011936,2.3735036055
