# Supplementary material for: syn-Selective alkylarylation of terminal alkynes via the combination of photoredox and nickel catalysis
Source: Nat Commun. 2018 Oct 31;9:4543. doi: 10.1038/s41467-018-06904-9 (PMC6208420; doi:10.1038/s41467-018-06904-9)
Supplement: Supplementary file 2 — Supplementary Information [file 41467_2018_6904_MOESM2_ESM.pdf]

Supplementary Materials for

syn-Selective Alkylarylation of Terminal Alkynes via the Combination of Photoredox and Nickel Catalysis

chu *et al*

correspondence to: lingling.chu1@dhu.edu.cn

This PDF Files includes:

Supplementary Methods

Supplementary Discussion

Supplementary Table 1-4

Supplementary Figure 1-185

Supplementary References

Supplementary Methods

General Considerations: Commercial reagents were purchased from Aldrich, TCI, Energy Chemical and J&K chemical, and were used as received. All reactions were carried out in oven-dried glassware under an atmosphere of nitrogen unless otherwise noted. Chromatographic purification of products was accomplished by flash chromatography using silica gel. Thin-layer chromatography (TLC) was performed on Silicycle 250 mm silica gel F-254 plates. ^{1}H, ^{19}F and ^{13}C NMR spectra were recorded on Bruker 400 (400, 375 and 100 MHz) and Bruker 600 (600, 565 and 150 MHz), and are internally referenced to residual solvent signals (for $CDCl_3$, δ 7.26 ppm and 77.0 ppm; DMSO-d_6, 2.50 ppm and 39.52 ppm, CD_2Cl_2, 5.35 ppm). Data for ^{1}H NMR are reported as follows: chemical shift (δ ppm), multiplicity (s = singlet, d = doublet, t = triplet, m = multiplet, br = broad), integration, coupling constant (Hz). ^{13}C spectra were reported as chemical shifts in ppm and multiplicity where appropriate. High resolution mass spectra were obtained at Shanghai Institute of Organic Chemistry mass spectrometry facilities.

General procedure A (GPA) for the synthesis of methyl oxalates: A round-bottom flask was charged with tertiary alcohol (1.0 equiv.) and CH_2Cl_2 [0.1M]. Triethylamine (1.2 equiv.) and DMAP (0.1 equiv.) were added followed by drop-wise addition of methyl chlorooxoacetate (1.2 equiv.). The reaction was stirred for 4 hour at room temperature, then quenched with sat. NH_4Cl (aq). The aqueous phase was extracted with DCM, and the organic extracts were dried over Na_2SO_4 and concentrated. The crude material was purified by flash column chromatography on silica gel.

General procedure B (GPB) for the synthesis of methyl oxalates: A round-bottom flask was charged with tertiary alcohol (1.0 equiv.) and Et_2O [0.27 M]. Pyridine (1.2 equiv.) were added followed by dropwise addition of methyl chlorooxoacetate (1.2 equiv.). The reaction was stirred for 4 hour at room temperature, then the organic phase was washed with water and saturated aqueous $NaHCO_3$ solution, dried over

$MgSO_4$ and concentrated. The crude material was purified by flash column chromatography on silica gel.

General procedure C (GPC) for the synthesis of cesium salts: A round-bottom flask was charged with methyl oxalate (1.0 equiv.) followed by the addition of THF [1 M]. To this solution, 1 N aq. CsOH (0.9 equiv. or 0.8 equiv.) was added dropwise. The mixture was stirred vigorously for 1 hour at room temperature, then extracted with hexane three times, the combined water layers were concentrated under reduced pressure to give the target product.

General procedure D (GPD) for the photoredox/Ni catalyzed alkylarylation of alkynes: To a flame-dried 8 mL reaction vial was charged with $NiCl_2$•DME (0.02 mmol, 20 mol%), dtbbpy (0.02 mmol, 20 mol%), Ir[dF(CF_3)ppy]$_2$(dtbbpy)PF_6 (0.003 mmol, 3 mol%), aryl bromide (0.2 mmol, 2.0 equiv., if solid) and cesium salt (0.15 mmol, 1.5 equiv.), the vial was capped. After evacuated and backfilled nitrogen three times, DMSO [0.05 M] was added via a syringe, aryl bromide (0.2 mmol, 2.0 equiv., if liquid), followed by the addition of terminal alkyne (0.1 mmol, 1.0 equiv.). The reaction mixture was then irradiated with a 90 W blue LED lamp (at approximately 3 cm away from the light source) with cooling from a fan for 18h. The reaction was quenched with H_2O, extracted with ethyl acetate. The combined organic layers were dried with Mg_2SO_4, filtered, and concentrated in vacuo. The crude material was purified by flash chromatography to afford the product.

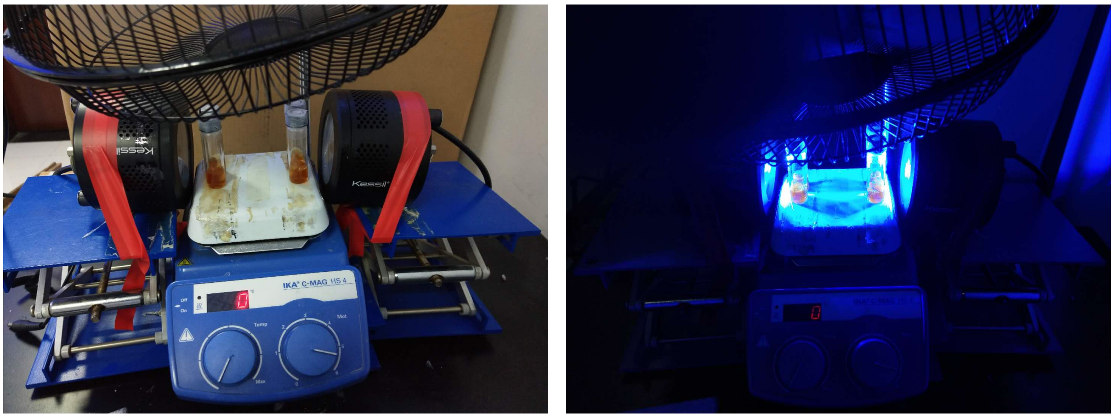

Supplementary Figure 1. Setup of this metallaphotoredox manifold.

Large scale reaction

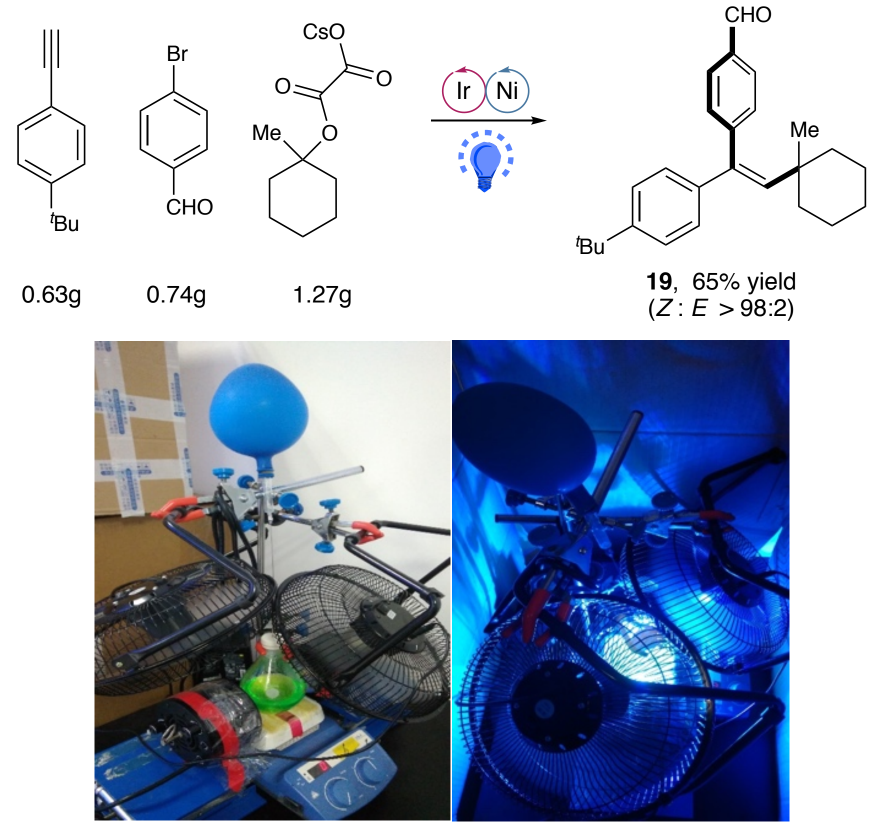

Supplementary Figure 2. Large scale reaction

A 100 mL round bottom flask containing a stirring bar was charged with $NiCl_2$•DME (175.8 mg, 0.8 mmol, 20 mol%), dtbbpy (214.7mg, 0.8 mmol, 20 mol%), Ir[dF(CF_3)ppy]$_2$(dtbbpy)PF_6 (134.6 mg, 0.12 mmol, 3 mol%), 4-bromobenzaldehyde (1.5 g, 8 mmol, 2.0 equiv) and cesium 2-((1-methylcyclohexyl)oxy)-2-oxoacetate (1.9 g, 6 mmol, 1.5 equiv.) The flask was capped. After evacuated and backfilled

nitrogen three times, DMSO (80 mL) was added via a syringe, followed by the addition of 1-(tert-butyl)-4-ethynylbenzene (721 uL, 4 mmol, 1.0 equiv.). The reaction mixture was then irradiated with two 90 W blue LED lamps (at approximately 3 cm away from the light source, add a nitrogen ball protection) with cooling from two fans for 72 h. The reaction was quenched with H_2O, extracted with ethyl acetate. The combined organic layers were dried with Mg_2SO_4, filtered, and concentrated in vacuo. The crude material was purified by flash chromatography (PE: EA= 60:1) to afford the product as a pale yellow oil (936 mg, 65%, *Z*:*E* > 98:2).

Analytical Data of Compounds

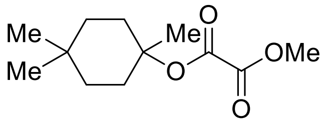

Methyl (1,4,4-trimethylcyclohexyl) oxalate (S1): According to **GPA**, 1,4,4-trimethylcyclohexanol (0.7 g, 5 mmol, 1.0 equiv.), Triethylamine (834 uL, 6 mmol, 1.2 equiv.), DMAP (61 mg, 0.5 mmol, 0.1 equiv.) and methyl chlorooxoacetate (552 uL, 6 mmol, 1.2 equiv.) in DCM (50 mL, 0.1M) were used, After 4 h, The crude material was purified by flash column chromatography on silica gel (PE: EA= 30:1) to give the target product as colorless oil (1.0 g, 89% yield). ^{1}H NMR (600 MHz, $CDCl_3$) δ 3.88 (s, 3H), 2.18-2.15 (m, 2H), 1.64 -1.59 (m, 2H), 1.57 (s, 3H), 1.45 -1.41 (m, 2H), 1.26-1.22 (m, 2H), 0.94 (s, 3H), 0.92 (s, 3H). ^{13}C NMR (150 MHz, $CDCl_3$) δ 159.02, 156.69, 86.56, 53.19, 34.71, 32.30, 29.19, 25.74, 24.69. HRMS (ESI+): calcd for $C_{12}H_{24}NO_4^+$ (M+NH_4) 246.1700, found 246.1701.

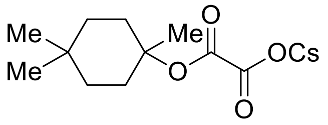

Cesium 2-oxo-2-((1,4,4-trimethylcyclohexyl)oxy) acetate (S2): According to **GPC**, methyl (1,4,4-trimethylcyclohexyl) oxalate (685 mg, 3 mmol, 1.0 equiv.) and 1 N aq. CsOH (2.7 mL, 2.7 mmol, 0.9 equiv.) in THF (3 mL, 1 M) were used. After 1 h,

the combined water layers were concentrated under reduced pressure to give the target product as a colorless solid (860 mg, 92% yield). ^{1}H NMR (600 MHz, DMSO-d_6) δ 1.98-1.95 (m, 2H), 1.48 (t, J = 11.5 Hz, 2H), 1.40-1.36 (m, 5H), 1.13-1.11 (m, 2H), 0.88 (s, 6H). ^{13}C NMR (150 MHz, DMSO-d_6) δ 167.60, 163.54, 79.22, 34.38, 32.21, 30.55, 29.04, 24.94.

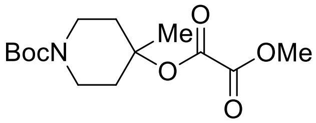

1-(tert-Butoxycarbonyl)-4-methylpiperidin-4-yl methyl oxalate (S3): According to **GPB**, tert-butyl 4-hydroxy-4-methylpiperidine-1-carboxylate (431 mg, 2 mmol, 1.0 equiv.), pyridine (0.2 mL, 2. 4 mmol, 1.2 equiv.) and methyl chlorooxoacetate (0.22 mL, 2.4 mmol, 1.2 equiv.) in Et_2O (7.0 mL, 0.27 M) were used. After 4 h, The crude material was purified by flash column chromatography on silica gel (PE: EA= 15:1) to give the target product as colorless solid (410 mg, 68% yield). ^{1}H NMR (400 MHz, $CDCl_3$) δ 3.88 (s, 3H), 3.81 (br, 2H), 3.13-3.07 (m, 2H), 2.27-2.24 (m, 2H), 1.66-1.59 (m, 5H), 1.46 (s, 9H). ^{13}C NMR (150 MHz, $CDCl_3$) δ 158.68, 156.63, 154.74, 84.02, 79.77, 53.43, 35.58, 30.97, 28.46, 24.75. HRMS (ESI+): calcd for $C_{14}H_{24}NO_6^+$ (M+H) 302.1598, found 302.1603.

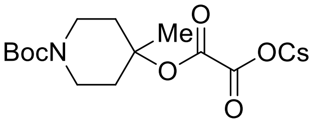

Cesium 2-((1-(tert-butoxycarbonyl)-4-methylpiperidin-4-yl)oxy)-2-oxoacetate (S4): According to **GPC**, 1-(tert-butoxycarbonyl)-4-methylpiperidin-4-yl methyl oxalate (301 mg, 1 mmol, 1.0 equiv.) and 1 N aq. CsOH (0.8 mL, 0.8 mmol, 0.8 equiv.) in THF (1 mL, 1 M) were used. After 1 h, the combined water layers were concentrated under reduced pressure to give the target product as a colorless solid (281 mg, 84% yield). ^{1}H NMR (600 MHz, DMSO-d_6) δ 3.62-3.60 (m, 2H), 3.03 (br, 2H), 2.05-2.03 (m 2H), 1.49-1.46 (m, 2H), 1.44 (s, 3H), 1.40 (s, 9H). ^{13}C NMR (150

MHz, DMSO-d_6) δ 167.43, 163.13, 154.00, 78.61 (s), 77.09, 35.38, 35.34, 28.07, 24.72.

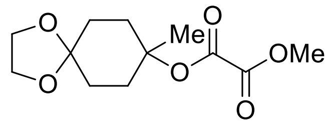

Methyl (8-methyl-1,4-dioxaspiro[4.5]decan-8-yl) oxalate (S5): According to **GPB**, 8-methyl-1,4-dioxaspiro[4.5]decan-8-ol (0.7 g, 4 mmol, 1.0 equiv.), Pyridine (0.39 mL, 4.8 mmol, 1.2 equiv.) and methyl chlorooxoacetate (0.44 mL, 4.8 mmol, 1.2 equiv.) in Et_2O (15 mL, 0.27 M) were used. after 4 h, The crude material was purified by flash column chromatography on silica gel (PE: EA= 15:1) to give the target product as colorless solid (723 mg, 70% yield). ^{1}H NMR (400 MHz, $CDCl_3$) δ 3.97-3.91 (m, 4H), 3.86 (s, 3H), 2.36-2.33 (m, 2H), 1.82-1.70 (m, 4H), 1.62-1.58 (m, 5H). ^{13}C NMR (150 MHz, $CDCl_3$) δ 158.77, 156.72, 107.81, 85.13, 64.38, 64.23, 53.28, 33.76, 30.40, 24.59. HRMS (ESI+): calcd for $C_{12}H_{22}O_6N^+$ (M+NH_4) 276.1442, found 276.1443.

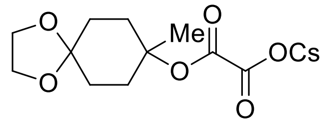

Cesium-2-((8-methyl-1,4-dioxaspiro[4.5]decan-8-yl)oxy)-2-oxoacetate (S6): According to **GPC**, methyl (8-methyl-1,4-dioxaspiro[4.5]decan-8-yl) oxalate (517 mg, 2 mmol, 1.0 equiv.) and 1 N aq. CsOH (1.6 mL, 1.6 mmol, 0.8 equiv.) in THF (2 mL, 1 M) were used. After 1 h, the combined water layers were concentrated under reduced pressure to give the target product as a colorless solid (554 mg, 92% yield). ^{1}H NMR (400 MHz, DMSO-d_6) δ 3.85 (s, 4H), 2.15-2.12 (m, 2H), 1.70-1.63 (m, 2H), 1.57-1.45 (m, 4H), 1.42 (s, 3H). ^{13}C NMR (150 MHz, DMSO-d_6) δ 167.59, 163.33, 107.49, 78.10, 63.67, 63.55, 33.76, 30.05, 24.86.

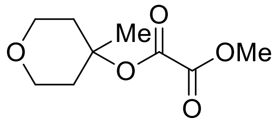

Methyl (4-methyltetrahydro-2H-pyran-4-yl) oxalate (S7): According to **GPA**, 4-methyltetrahydro-2H-pyran-4-ol (581 mg, 5 mmol, 1.0 equiv.), Triethylamine (0.83 mL, 6 mmol, 1.2 equiv.), DMAP (61 mg, 0.5 mmol, 0.1 equiv.) and methyl chlorooxoacetate (0.6 mL, 6 mmol, 1.2 equiv.) in CH_2Cl_2 (DCM) (50 mL, 0.1M) were used. after 4 h, The crude material was purified by flash column chromatography on silica gel (PE: EA= 20:1) to give the target product as yellow oil (1.0 g, 94% yield). ^{1}H NMR (400 MHz, $CDCl_3$) δ 3.89 (s, 3H), 3.78-3.66 (m, 4H), 2.24-2.21 (m, 2H), 1.83-1.76 (m, 2H), 1.63 (s, 3H). ^{13}C NMR (150 MHz, $CDCl_3$) δ 158.65, 156.59, 83.24, 63.54, 53.35, 36.41, 24.76. HRMS (ESI+): calcd for $C_9H_{15}O_5^+$ (M+H) 203.0914, found 203.0914.

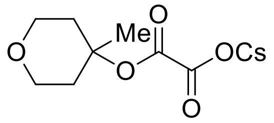

Cesium 2-((4-methyltetrahydro-2H-pyran-4-yl)oxy)-2-oxoacetate (S8): According to **GPC**, methyl (4-methyltetrahydro-2H-pyran-4-yl) oxalate (404 mg, 2 mmol, 1.0 equiv.) and 1 N aq. CsOH (1.6 mL, 1.6 mmol, 0.8 equiv.) in THF (2 mL, 1 M) were used. After 1 h, the combined water layers were concentrated under reduced pressure to give the target product as a colorless solid (482 mg, 94% yield). ^{1}H NMR (600 MHz, DMSO-d_6) δ 3.59-3.54 (m, 4H), 2.00-1.98 (m, 2H), 1.64-1.59 (m, 2H), 1.46 (s, 3H). ^{13}C NMR (150 MHz, DMSO-d_6) δ 167.40, 163.24, 76.65, 62.86, 36.57, 24.85.

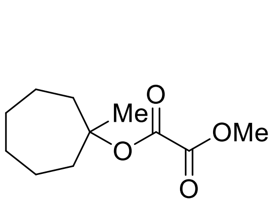

Methyl (1-methylcycloheptyl) oxalate (S9): According to **GPA**, 1-methylcycloheptanol (513 mg, 4 mmol, 1.0 equiv.), triethylamine (0.7 mL, 4.8 mmol, 1.2 equiv.), DMAP (48.8 mg, 0.4 mmol, 0.1 equiv.) and methyl

chlorooxoacetate (0.4 mL, 4.8 mmol, 1.2 equiv.) in CH_2Cl_2 (DCM) (40 mL, 0.1M) were used. After 4 h, The crude material was purified by flash column chromatography on silica gel (PE: EA= 30:1) to give the target product as colorless oil (583 mg, 68% yield). ^{1}H NMR (400 MHz, $CDCl_3$) δ 3.80 (s, 3H), 2.18-2.12 (m, 2H), 1.81-1.75 (m, 2H), 1.60-1.45 (m, 9H), 1.42-1.35 (m, 2H). ^{13}C NMR (150 MHz, $CDCl_3$) δ 159.10, 156.79, 91.04, 53.08, 39.78, 29.26, 26.40, 22.42. HRMS (ESI+): calcd for $C_{11}H_{19}O_4^+$ (M+H) 215.1278, found 215.1274.

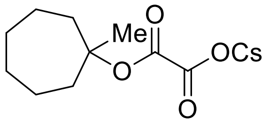

Cesium 2-((1-methylcycloheptyl)oxy)-2-oxoacetate (S10): According to **GPC**, methyl (1-methylcycloheptyl) oxalate (429 mg, 2 mmol, 1.0 equiv.) and 1 N aq. CsOH (1.6 mL, 1.6 mmol, 0.8 equiv.) in THF (2 mL, 1 M) were used. After 1 h, the combined water layers were concentrated under reduced pressure to give the target product as a colorless solid (478 mg, 72% yield). ^{1}H NMR (600 MHz, DMSO-d_6) δ 2.04-2.00 (m, 2H), 1.70-1.66 (m, 2H), 1.54-1.46 (m, 6H), 1.42 (s, 3H), 1.36-1.33 (m, 2H). ^{13}C NMR (150 MHz, DMSO-d_6) δ 167.51, 164.02, 84.43, 40.17, 29.30, 27.17, 22.51.

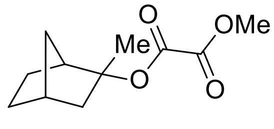

Methyl ((1S,2R,4R)-2-methylbicyclo[2.2.1]heptan-2-yl) oxalate (S11): According to **GPA**, (1S,2R,4R)-2-methylbicyclo[2.2.1]heptan-2-ol (631 mg, 5 mmol, 1.0 equiv.), Triethylamine (0.8 mL, 6 mmol, 1.2 equiv.), DMAP (62 mg, 0.5 mmol, 0.1 equiv.) and methyl chlorooxoacetate (0.6 mL, 6 mmol, 1.2 equiv.) in CH_2Cl_2 (DCM) (50 mL, 0.1M) were used. The crude material was purified by flash column chromatography on silica gel (PE: EA= 30:1) to give the target product as colorless oil (1.0 g, 94% yield). ^{1}H NMR (600 MHz, $CDCl_3$) δ 3.88 (s, 3H), 2.66-2.65 (m, 1H), 2.25-2.24 (m, 1H), 1.76-1.72 (m, 1H), 1.66-1.62 (m, 2H), 1.58-1.57 (m, 1H), 1.56 (s, 3H), 1.50-1.48

(m, 1H), 1.45-1.40 (m, 1H), 1.34-1.32 (m, 1H), 1.29-1.25 (m, 1H). ^{13}C NMR (150 MHz, $CDCl_3$) δ 158.94, 157.10, 91.38, 53.25, 46.84, 44.99, 37.44, 36.10, 27.96, 25.19, 22.50. HRMS (ESI+): calcd for $C_{11}H_{17}O_4^+$ (M+H) 213.1121, found 213.1118.

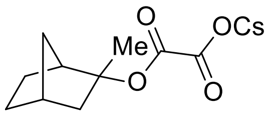

Cesium 2-(((1S,2R,4R)-2-methylbicyclo[2.2.1]heptan-2-yl)oxy)-2-oxoacetate (S12): According to GPC, methyl ((1S,2R,4R)-2-methylbicyclo[2.2.1]heptan-2-yl) oxalate (637 mg, 3 mmol, 1.0 equiv.) and 1 N aq. CsOH (2.7 mL, 2.7 mmol, 0.9 equiv.) in THF (3 mL, 1 M) were used. After 1 h, the combined water layers were concentrated under reduced pressure to give the target product as a colorless solid (766 mg, 86% yield). ^{1}H NMR (600 MHz, DMSO-d_6) δ 2.13 (s, 1H), 1.66-1.63 (m, 1H), 1.57 -1.54 (m, 1H), 1.49-1.41 (m, 3H), 1.39 (br, 4H), 1.27 -1.22 (m, 1H), 1.20-1.18 (m, 1H), 1.15-1.11(m, 1H). ^{13}C NMR (150 MHz, DMSO-d_6) δ 167.42, 163.49, 84.74, 46.52, 45.30, 36.96, 35.60, 27.88, 25.46, 21.92.

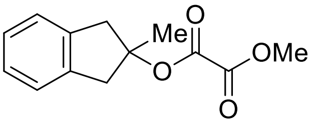

Methyl (2-methyl-2,3-dihydro-1H-inden-2-yl) oxalate (S13): According to **GPA**, 2-methyl-2,3-dihydro-1H-inden-2-ol (296 mg, 2 mmol, 1.0 equiv.), Triethylamine (0.3 mL, 2.4 mmol, 1.2 equiv.), DMAP (24.4 mg, 0.2 mmol, 0.1 equiv.) and methyl chlorooxoacetate (0.2 mL, 2.4 mmol, 1.2 equiv.) in CH_2Cl_2 (DCM) (20 mL, 0.1M) were used. After 4 h, The crude material was purified by flash column chromatography on silica gel (PE: EA= 10:1) to give the target product as colorless solid (403 mg, 86% yield). ^{1}H NMR (600 MHz, $CDCl_3$) δ 7.20-7.17 (m, 4H), 3.86 (s, 3H), 3.52-3.50 (m, 2H), 3.26-3.23 (m, 2H), 1.74 (s, 3H). ^{13}C NMR (150 MHz, $CDCl_3$) δ 158.64, 157.04, 139.75, 126.96, 124.63, 92.12, 53.43, 45.73, 24.16. HRMS (ESI+): calcd for $C_{13}H_{18}O_4N^+$ (M+NH_4) 252.1230, found 252.1233.

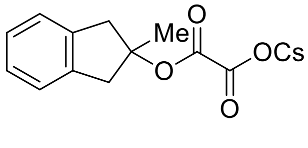

Cesium 2-((2-methyl-2,3-dihydro-1H-inden-2-yl)oxy)-2-oxoacetate (S14)： According to **GPC**, methyl (1-methylcycloheptyl) oxalate (351 mg, 1.5 mmol, 1.0 equiv.) and 1 N aq. CsOH (1.2 mL, 1.2 mmol, 0.8 equiv.) in THF (2 mL, 1 M) were used. After 1 h, the combined water layers were concentrated under reduced pressure to give the target product as a colorless solid (359 mg, 85% yield). ^{1}H NMR (600 MHz, DMSO-d_6) δ 7.20-7.19 (m, 2H), 7.14-7.13 (m, 2H), 3.31-3.28 (m, 2H), 3.10-3.07 (m, 2H), 1.57 (s, 3H). ^{13}C NMR (150 MHz, DMSO-d_6) δ 167.74, 163.44, 141.01, 126.92, 124.90, 86.97, 45.95, 24.86.

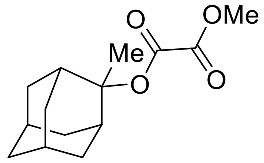

Methyl ((1r,3r,5r,7r)-2-methyladamantan-2-yl) oxalate (S15)： According to **GPA**, (1r,3r,5r,7r)-2-methyladamantan-2-ol (1.0 g, 6 mmol, 1.0 equiv.) Triethylamine (1.0 mL, 7.2 mmol, 1.2 equiv.), DMAP (73.3 mg, 0.6 mmol, 0.1 equiv.) and methyl chlorooxoacetate (0.7 mL, 7.2 mmol, 1.2 equiv.) in CH_2Cl_2 (DCM) (60 mL, 0.1M) were used. After 4 h, the crude material was purified by flash column chromatography on silica gel (PE: EA= 30:1) to give the target product as colorless solid (1.2 g, 75% yield). ^{1}H NMR (600 MHz, $CDCl_3$) δ 3.88 (s, 3H), 2.37 (br, 2H), 2.07-2.04 (m, 2H), 1.90-1.88 (m, 2H), 1.83(br, 2H), 1.79-1.77 (m, 2H), 1.72 (br, 2H), 1.69 (s, 3H), 1.61-1.59 (m, 2H). ^{13}C NMR (150 MHz, $CDCl_3$) δ 159.22, 156.46, 92.06, 53.28, 38.00, 36.05, 34.58, 32.92, 27.23, 26.53, 22.05. HRMS (ESI+): calcd for $C_{14}H_{21}O_4^+$ (M+H) 253.1434, found 253.1432.

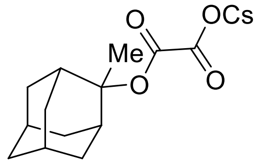

Cesium 2-(((1r,3r,5r,7r)-2-methyladamantan-2-yl)oxy)-2-oxoacetate (S16): According to **GPC**, methyl ((1r,3r,5r,7r)-2-methyladamantan-2-yl) oxalate (757 mg, 3 mmol, 1.0 equiv.) and 1 N aq. CsOH (2.4 mL, 2.4 mmol, 0.8 equiv.) in THF (3 mL, 1 M) were used. After 1 h, the combined water layers were concentrated under reduced pressure to give the target product as a colorless solid (780 mg, 88% yield). ^{1}H NMR (600 MHz, DMSO-d_6) δ 2.20 (br, 2H), 2.09-2.07 (m, 2H), 1.86-1.83 (m, 2H), 1.76-1.73 (m, 2H), 1.68-1.66 (m, 4H), 1.54 (s, 3H), 1.46-1.44 (m, 2H). ^{13}C NMR (150 MHz, DMSO-d_6) δ 167.25, 163.60, 83.99, 37.65, 35.73, 33.78, 32.18, 26.82, 26.16, 22.10.

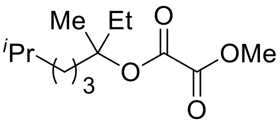

3,7-Dimethyloctan-3-yl methyl oxalate (S17): According to **GPA**, 3,7-dimethyloctan-3-ol (1.1 g, 7 mmol, 1.0 equiv.), Triethylamine (1.2 mL, 8.4 mmol, 1.2 equiv.), DMAP (85.5 mg, 0.7 mmol, 0.1 equiv.) and methyl chlorooxoacetate (0.8 mL, 8.4 mmol, 1.2 equiv.) in CH_2Cl_2 (70 mL, 0.1M) were used. After 4 h, the crude material was purified by flash column chromatography on silica gel (PE: EA= 30:1) to give the target product as colorless solid (1.7 g, 99% yield). ^{1}H NMR (600 MHz, $CDCl_3$) δ 3.81 (s, 3H), 1.94-1.88 (m, 1H), 1.86-1.76 (m, 2H), 1.75-1.70 (m, 1H), 1.53-1.46 (m, 1H), 1.44 (s, 3H), 1.28-1.23 (m, 2H), 1.15-1.11 (m, 2H), 0.86 (t, J = 7.5 Hz, 3H), 0.82 (d, J = 6.8 Hz, 6H). ^{13}C NMR (150 MHz, $CDCl_3$) δ 159.02, 156.66, 90.16, 53.07, 39.01, 37.61, 30.60, 27.68, 22.93, 22.47, 21.19, 7.87. HRMS (ESI+): calcd for $C_{13}H_{28}O_4N^+$ (M+NH$_4$) 262.2013, found 262.2013.

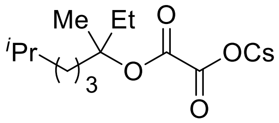

Cesium 2-((3,7-dimethyloctan-3-yl)oxy)-2-oxoacetate (S18): According to **GPC**, 3,7-dimethyloctan-3-yl methyl oxalate (1.0 g, 2.8 mmol, 1.0 equiv.) and 1 N aq. CsOH (2.5 mL, 2.5 mmol, 0.9 equiv.) in THF (3 mL, 1 M) were used. After 1 h, the combined water layers were concentrated under reduced pressure to give the target product as a colorless solid (452 mg, 50% yield). ^{1}H NMR (600 MHz, DMSO-d_6) δ 1.82 -1.52 (m, 5H), 1.31-1.26 (m, 5H), 1.13 (br, 2H), 0.86-0.81 (m, 9H). ^{13}C NMR (100 MHz, DMSO-d_6) δ 167.00, 163.48, 83.06, 38.85, 37.70, 30.43, 27.28, 23.19, 22.49, 20.66, 7.78.

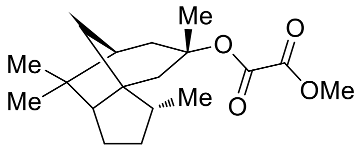

Methyl((3R,3aS,5S,7S)-3,5,8,8-tetramethyloctahydro-1H-3a,7-methanoazulen-5-yl) oxalate (S19): According to **GPA**, (3R,3aS,5S,7S)-3,5,8,8-tetramethyloctahydro-1H- 3a,7-methanoazulen-5-ol (1.0 g, 4.5 mmol, 1.0 equiv.), triethylamine (0.8 mL, 5.4 mmol, 1.2 equiv.), DMAP (61.1 mg, 0.5 mmol, 0.1 equiv.) and methyl chlorooxoacetate (0.5 mL, 5.4 mmol, 1.2 equiv.) in CH_2Cl_2 (45 mL, 0.1 M) were used. After 4 h, the crude material was purified by flash column chromatography on silica gel (PE: EA= 30:1) to give the target product as colorless solid (1.2 g, 86% yield). ^{1}H NMR (600 MHz, $CDCl_3$) δ 3.82 (s, 3H), 2.42 -2.41 (m, 1H), 2.14-2.11 (m, 1H), 2.07-2.01 (m, 1H), 1.87-1.82 (m, 1H), 1.79 (t, J = 8.1 Hz, 1H), 1.68-1.63 (m, 2H), 1.59 (s, 3H), 1.54-1.46 (m, 2H), 1.41-1.36 (m, 2H), 1.35-1.32 (m, 1H), 1.29-1.23 (m, 1H), 1.14 (s, 3H), 0.95 (s, 3H), 0.81 (d, J = 7.1 Hz, 3H). ^{13}C NMR (150 MHz, $CDCl_3$) δ 159.02, 156.55, 91.15, 56.75, 56.66, 53.86, 53.16, 43.48, 41.16, 41.04, 36.89, 32.82, 31.22, 28.31, 26.92, 25.38, 25.23, 15.47. HRMS (ESI+): calcd for $C_{18}H_{32}O_4N^+$ (M+H) 326.2326, found 326.2325.

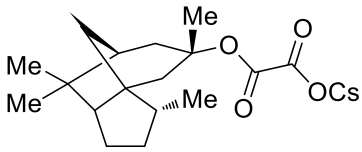

Cesium-2-oxo-2-(((3R,3aS,5S,7S)-3,5,8,8-tetramethyloctahydro-1H-3a,7-methano azulen-5-yl)oxy) acetate (S20): According to **GPC**, methyl ((3R,3aS,5S,7S)-3,5,8,8-tetramethyloctahydro-1H-3a,7-methanoazulen-5-yl) oxalate (830 mg, 2.7 mmol, 1.0 equiv.) and 1 N aq. CsOH (2.2 mL, 2.2 mmol, 0.8 equiv.) in THF (3 mL, 1 M) were used. After 1 h, the combined water layers were concentrated under reduced pressure to give the target product as a colorless solid (765 mg, 83% yield). ^{1}H NMR (600 MHz, DMSO-d_6) δ 2.35-2.34 (m, 1H), 1.93-1.82 (m, 3H), 1.75 (t, J = 8.0 Hz, 1H), 1.64-1.61 (m, 1H), 1.59-1.56 (m, 1H), 1.50- 1.46(m, 1H), 1.45 (s, 3H) 1.39-1.31 (m, 4H), 1.27-1.22 (m, 1H), 1.15 (s, 3H), 0.90 (s, 3H), 0.81 (d, J = 7.1 Hz, 3H). ^{13}C NMR (150 MHz, DMSO-d_6) δ 167.70, 164.07, 84.46, 56.80, 56.68, 54.05, 43.48, 41.15, 40.79, 36.88, 33.46, 31.05, 28.87, 27.79, 26.20, 25.35, 15.92.

(*Z*)-4-(1-(4-(tert-Butyl)phenyl)-2-(1-methylcyclohexyl)vinyl)benzaldehyde (19)

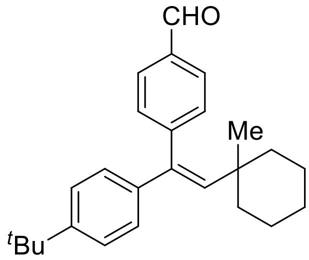

According to **GPD**, $NiCl_2$•DME (4.4 mg, 0.02 mmol, 20 mol%), dtbbpy (5.4 mg, 0.02 mmol, 20 mol%), Ir[dF(CF_3)ppy]$_2$(dtbbpy)PF_6 (3.4 mg, 0.003 mmol, 3 mol%), 4-bromobenzaldehyde (37.0 mg, 0.2 mmol, 2.0 equiv.), cesium 2-((1-methylcyclohexyl)oxy)-2-oxoacetate (47.7 mg, 0.15 mmol, 1.5 equiv.) and 1-(tert-butyl)-4-ethynylbenzene (18.1 uL, 0.1 mmol, 1.0 equiv.) in DMSO (2 mL) were used. After 18 hour, the product was isolated by flash chromatography (PE: EA= 60:1) as a pale yellow oil (29.6 mg, 82%, Z:E = 94:6). ^{1}H NMR (600 MHz, $CDCl_3$) δ 10.04 (s, 1H), 7.86 (d, J = 8.0 Hz, 2H), 7.39 (d, J = 8.0 Hz, 2H), 7.27 (d, J = 8.5 Hz, 2H), 7.09 (d, J = 8.5 Hz, 2H), 6.08 (s, 1H), 1.48-1.39 (m, 8H), 1.29 (s, 9H), 1.10-1.05 (m, 2H), 0.98 (s, 3H). ^{13}C NMR (150 MHz, $CDCl_3$) δ 192.05, 150.02, 148.25, 140.46,

139.37, 138.82, 135.04, 130.76, 129.29, 126.40, 125.10, 39.51, 37.21, 34.43, 31.29, 29.30, 26.06, 22.91. HRMS (ESI+): calcd for $C_{26}H_{33}O^+$ (M+H) 361.2531, found 361.2526.

(*Z*)-4-(2-(1-Methylcyclohexyl)-1-phenylvinyl)benzaldehyde (20)

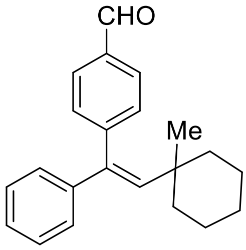

According to **GPD**, $NiCl_2$•DME (4.4 mg, 0.02 mmol, 20 mol%), dtbbpy (5.4 mg, 0.02 mmol, 20 mol%), Ir[dF(CF_3)ppy]$_2$(dtbbpy)PF_6 (3.4 mg, 0.003 mmol, 3 mol%), 4-bromobenzaldehyde (37.0 mg, 0.2 mmol, 2.0 equiv.), cesium 2-((1-methylcyclohexyl)oxy)-2-oxoacetate (47.7 mg, 0.15 mmol, 1.5 equiv.) and ethynylbenzene (11.0 uL, 0.1 mmol, 1.0 equiv.) in DMSO (2 mL) were used. After 18 hour, the product was isolated by flash chromatography (PE: EA= 60:1) as a pale yellow oil (25.3 mg, 83%, *Z:E* = 96:4). ^{1}H NMR (400 MHz, $CDCl_3$) δ 10.04 (s, 1H), 7.87 (d, *J* = 8.2 Hz, 2H), 7.40 (d, *J* = 8.1 Hz, 2H), 7.25-7.21 (m, 3H), 7.17-7.14 (m, 2H), 6.08 (s, 1H), 1.48-1.38 (m, 7H), 1.28-1.21 (m, 1H), 1.12- 1.06 (m, 2H), 1.00 (s, 3H). ^{13}C NMR (100 MHz, $CDCl_3$) δ 192.07, 148.11, 143.53, 140.21, 139.21, 135.10, 130.77, 129.34, 128.20, 127.00, 126.91, 39.46, 37.28, 29.28, 26.05, 22.92. HRMS (ESI+): calcd for $C_{22}H_{25}O^+$ (M+H) 305.1900, found 305.1899.

(*E*)-4-(2-(1-Methylcyclohexyl)-1-(m-tolyl)vinyl)benzaldehyde (21)

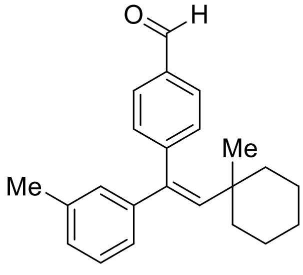

According to **GPD**, $NiCl_2$•DME (4.4 mg, 0.02 mmol, 20 mol%), dtbbpy (5.4 mg, 0.02 mmol, 20 mol%), Ir[dF(CF_3)ppy]$_2$(dtbbpy)PF_6 (3.4 mg, 0.003 mmol, 3 mol%), 4-bromobenzaldehyde (37.0 mg, 0.2 mmol, 2.0 equiv.), cesium 2-((1-methylcyclohexyl)oxy)-2-oxoacetate (47.7 mg, 0.15 mmol, 1.5 equiv.) and 1-ethynyl-3-methylbenzene (12.9 uL, 0.1 mmol, 1.0 equiv.) in DMSO (2 mL) were used. After 18 hour, the product was isolated by flash chromatography (PE: EA= 60:1) as a pale yellow oil (29.0 mg, 91%, *E:Z* = 92:8). ^{1}H NMR (400 MHz, $CDCl_3$) δ 10.04 (s, 1H), 7.86 (d, *J* = 8.2 Hz, 2H), 7.39 (d, *J* = 8.0 Hz, 2H), 7.16-7.12 (m, 1H), 7.04-7.02 (m, 1H), 6.97-6.93 (m, 2H), 6.05 (s, 1H), 2.29 (s, 3H), 1.46-1.38 (m, 7H), 1.25-1.20 (m, 1H), 1.11-1.05 (m, 2H), 0.99 (s, 3H). ^{13}C NMR (100 MHz, $CDCl_3$) δ 192.10, 148.23, 143.56, 140.04, 139.30, 137.77, 135.06, 130.74, 129.32, 128.09, 127.79, 127.57, 124.16, 39.47, 37.27, 29.30, 26.07, 22.93, 21.50. HRMS (ESI+): calcd for $C_{23}H_{27}O^+$ (M+H) 319.2056, found 319.2057.

(Z)-4-(1-(4-Ethylphenyl)-2-(1-methylcyclohexyl)vinyl)benzaldehyde (22)

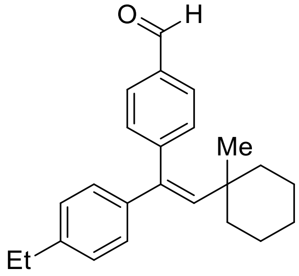

According to **GPD**, $NiCl_2$•DME (4.4 mg, 0.02 mmol, 20 mol%), dtbbpy (5.4 mg, 0.02 mmol, 20 mol%), Ir[dF(CF_3)ppy]$_2$(dtbbpy)PF_6 (3.4 mg, 0.003 mmol, 3 mol%), 4-bromobenzaldehyde (37.0 mg, 0.2 mmol, 2.0 equiv.), cesium 2-((1-methylcyclohexyl)oxy)-2-oxoacetate (47.7 mg, 0.15 mmol, 1.5 equiv.) and 1-ethyl-4-ethynylbenzene (14.0 uL, 0.1 mmol, 1.0 equiv.) in DMSO (2 mL) were used. After 18 hour, the product was isolated by flash chromatography (PE: EA= 60:1) as a pale yellow oil (29.9 mg, 90%, *Z:E* = 92:8). ^{1}H NMR (600 MHz, $CDCl_3$) δ 10.03 (s, 1H), 7.86 (d, *J* = 8.0 Hz, 2H), 7.39 (d, *J* = 7.9 Hz, 2H), 7.07 (br, 4H), 6.05 (s, 1H), 2.60 (q, *J* = 7.6 Hz, 2H), 1.48-1.37 (m, 8H), 1.20 (t, *J* = 7.6 Hz, 3H), 1.10-1.06 (m, 2H), 0.98 (s, 3H). ^{13}C NMR (150 MHz, $CDCl_3$) δ 192.06, 148.32, 143.18, 140.92,

139.37, 139.00, 135.03, 130.74, 129.30, 127.68, 126.79, 39.50, 37.20, 29.31, 28.42, 26.06, 22.91, 15.55. HRMS (ESI+): calcd for $C_{24}H_{29}O^+$ (M+H) 333.2213, found 333.2213.

(*E*)-4-(1-(4-Methoxyphenyl)-2-(1-methylcyclohexyl)vinyl)benzaldehyde (23)

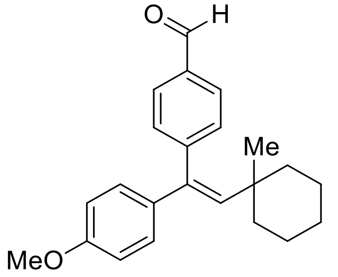

According to **GPD**, $NiCl_2$•DME (4.4 mg, 0.02 mmol, 20 mol%), dtbbpy (5.4 mg, 0.02 mmol, 20 mol%), Ir[dF(CF_3)ppy]$_2$(dtbbpy)PF_6 (3.4 mg, 0.003 mmol, 3 mol%), 4-bromobenzaldehyde (37.0 mg, 0.2 mmol, 2.0 equiv.), cesium 2-((1-methylcyclohexyl)oxy)-2-oxoacetate (47.7 mg, 0.15 mmol, 1.5 equiv.) and 1-ethynyl-4-methoxybenzene (13.0 uL, 0.1 mmol, 1.0 equiv.) in DMSO (2 mL) were used. After 18 hour, the product was isolated by flash chromatography (PE: EA= 40:1) as a pale yellow oil (25.1 mg, 75%, *E:Z* = 89:11). ^{1}H NMR (400 MHz, $CDCl_3$) δ 10.04 (s, 1H), 7.86 (d, *J* = 8.2 Hz, 2H), 7.38 (d, *J* = 8.0 Hz, 2H), 7.07 (d, *J* = 8.9 Hz, 2H), 6.78 (d, *J* = 8.9 Hz, 2H), 5.98 (s, 1H), 3.77(s, 3H), 1.51-1.38 (m, 8H), 1.12-1.03 (m, 2H), 0.98 (s, 3H). ^{13}C NMR (100 MHz, $CDCl_3$) δ 192.07, 158.79, 148.43, 138.61, 138.56, 136.28, 135.05, 130.73, 129.33, 127.99, 113.55, 55.32, 39.52, 37.15, 29.39, 26.07, 22.91. HRMS (ESI+): calcd for $C_{23}H_{27}O_2^+$ (M+H) 335.2006, found 335.2005.

(*E*)-4-(1-(4-Fluorophenyl)-2-(1-methylcyclohexyl)vinyl)benzaldehyde (24)

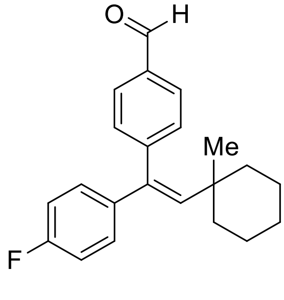

According to **GPD**, $NiCl_2$•DME (4.4 mg, 0.02 mmol, 20 mol%), dtbbpy (5.4 mg, 0.02 mmol, 20 mol%), Ir[dF(CF_3)ppy]$_2$(dtbbpy)PF_6 (3.4 mg, 0.003 mmol, 3 mol%), 4-bromobenzaldehyde (37.0 mg, 0.2 mmol, 2.0 equiv.), cesium 2-((1-methylcyclohexyl)oxy)-2-oxoacetate (47.7 mg, 0.15 mmol, 1.5 equiv.) and 1-ethynyl-4-fluorobenzene (11.5 uL, 0.1 mmol, 1.0 equiv.) in DMSO (2 mL) were used. After 18 hour, the product was isolated by flash chromatography (PE: EA= 60:1) as a pale yellow oil (29.0 mg, 90%, *E:Z* = 94:6). ^{1}H NMR (400 MHz, $CDCl_3$) δ 10.04 (s, 1H), 7.87 (d, *J* = 8.2 Hz, 2H), 7.38 (d, *J* = 8.0 Hz, 2H), 7.12-7.09 (m, 2H), 6.95-6.91 (m, 2H), 6.01 (s, 1H), 1.48-1.37 (m, 7H), 1.24-1.21 (m, 1H), 1.15-1.04 (m, 2H), 0.99 (s, 3H). ^{19}F NMR (565 MHz, $CDCl_3$) δ -115.81 – -115.86 (m). ^{13}C NMR (150 MHz, $CDCl_3$) δ 191.93, 162.02 (d, *J* = 246.6 Hz), 147.85, 140.14, 139.68 (d, *J* = 3.2 Hz), 138.25, 135.19, 130.66, 129.39, 128.45 (d, *J* = 7.9 Hz), 114.97 (d, *J* = 21.3 Hz), 39.41, 37.27, 29.20, 26.01, 22.89. HRMS (ESI+): calcd for $C_{22}H_{24}FO^+$ (M+H) 323.1806, found 323.1805.

(*E*)-4-(1-(2-Fluorophenyl)-2-(1-methylcyclohexyl)vinyl)benzaldehyde (25)

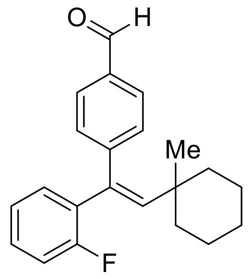

According to **GPD**, $NiCl_2$•DME (4.4 mg, 0.02 mmol, 20 mol%), dtbbpy (5.4 mg, 0.02 mmol, 20 mol%), Ir[dF(CF_3)ppy]$_2$(dtbbpy)PF_6 (3.4 mg, 0.003 mmol, 3 mol%), 4-bromobenzaldehyde (37.0 mg, 0.2 mmol, 2.0 equiv.), cesium 2-((1-methylcyclohexyl)oxy)-2-oxoacetate (47.7 mg, 0.15 mmol, 1.5 equiv.) and 1-ethynyl-2-fluorobenzene (11.3 uL, 0.1 mmol, 1.0 equiv.) in DMSO (2 mL) were used. After 18 hour, the product was isolated by flash chromatography (PE: EA= 60:1) as a pale yellow oil (25.8 mg, 80%, *E:Z* = 97:3). ^{1}H NMR (400 MHz, $CDCl_3$) δ 10.00 (s, 1H), 7.82 (d, *J* = 8.2 Hz, 2H), 7.44 (d, *J* = 8.0 Hz, 2H), 7.22-7.12 (m, 2H), 7.07-6.95 (m, 2H), 5.89 (s, 1H), 1.52-1.35 (m, 7H), 1.25-1.19 (m, 1H), 1.11-1.07 (m, 2H), 1.05 (s, 3H). ^{19}F NMR (565 MHz, $CDCl_3$) δ -114.54 – -114.59 (m). ^{13}C NMR

(150 MHz, $CDCl_3$) δ 191.97, 159.80 (d, *J* = 247.4 Hz), 147.83, 143.96, 134.97, 134.16, 132.23 (d, *J* = 13.7 Hz), 130.82 (d, *J* = 3.3 Hz), 130.27, 129.13, 128.67 (d, *J* = 8.2 Hz), 123.84 (d, *J* = 3.6 Hz), 115.89 (d, *J* = 22.7 Hz), 39.32, 37.60, 29.49, 26.01, 22.81. HRMS (ESI+): calcd for $C_{22}H_{24}FO^+$ (M+H) 323.1806, found 323.1806.

(*E*)-4-(1-(3-Chlorophenyl)-2-(1-methylcyclohexyl)vinyl)benzaldehyde (26)

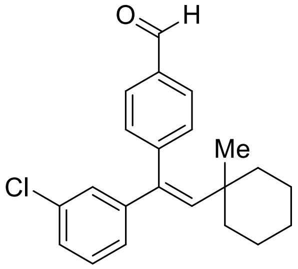

According to **GPD**, $NiCl_2$•DME (4.4 mg, 0.02 mmol, 20 mol%), dtbbpy (5.4 mg, 0.02 mmol, 20 mol%), $Ir[dF(CF_3)ppy]_2(dtbbpy)PF_6$ (3.4 mg, 0.003 mmol, 3 mol%), 4-bromobenzaldehyde (37.0 mg, 0.2 mmol, 2.0 equiv.), cesium 2-((1-methylcyclohexyl)oxy)-2-oxoacetate (47.7 mg, 0.15 mmol, 1.5 equiv.) and 1-chloro-3-ethynylbenzene (12.3 uL, 0.1 mmol, 1.0 equiv.) in DMSO (2 mL) were used. After 18 hour, the product was isolated by flash chromatography (PE: EA= 60:1) as a pale yellow oil (28.8 mg, 85%, *E:Z* = 92:8). ^1H NMR (400 MHz, $CDCl_3$) δ 10.05 (s, 1H), 7.88 (d, *J* = 8.2 Hz, 2H), 7.38 (d, *J* = 8.0 Hz, 2H), 7.18-7.14 (m, 3H), 7.01 – 6.99 (m, 1H), 6.07 (s, 1H), 1.45-1.37 (m, 7H), 1.28-1.21 (m, 1H), 1.13-1.07 (m, 2H), 0.99 (s, 3H). ^{13}C NMR (100 MHz, $CDCl_3$) δ 191.92, 147.18, 145.34, 141.43, 138.12, 135.26, 134.14, 130.66, 129.43, 129.34 , 126.96, 126.89, 125.13, 39.31, 37.38, 29.06, 25.94, 22.87. HRMS (ESI+): calcd for $C_{22}H_{24}ClO^+$ (M+H) 339.1510, found 339.1512.

(*E*)-4-(1-(2-Chlorophenyl)-2-(1-methylcyclohexyl)vinyl)benzaldehyde (27)

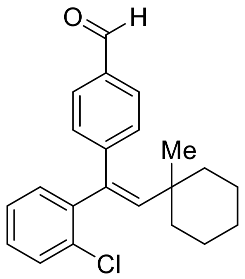

According to **GPD**, $NiCl_2$•DME (4.4 mg, 0.02 mmol, 20 mol%), dtbbpy (5.4 mg, 0.02 mmol, 20 mol%), $Ir[dF(CF_3)ppy]_2(dtbbpy)PF_6$ (3.4 mg, 0.003 mmol, 3 mol%), 4-bromobenzaldehyde (37.0 mg, 0.2 mmol, 2.0 equiv.), cesium 2-((1-methylcyclohexyl)oxy)-2-oxoacetate (47.7 mg, 0.15 mmol, 1.5 equiv.) and 1-chloro-2-ethynylbenzene (12.2 uL, 0.1 mmol, 1.0 equiv.) in DMSO (2 mL) were used. After 18 hour, the product was isolated by flash chromatography (PE: EA= 60:1) as a pale yellow oil (22.4 mg, 66%, *E:Z* = 95:5). ^{1}H NMR (400 MHz, $CDCl_3$) δ 9.98 (s, 1H), 7.81-7.79 (m, 2H), 7.52-7.50 (m, 2H), 7.36-7.33 (m, 1H), 7.27-7.24 (m, 2H), 7.21-7.14 (m, 2H), 5.72 (s, 1H), 1.53-1.33 (m, 8H), 1.22-1.10 (m, 2H), 1.08 (m, 3H). ^{13}C NMR (100 MHz, $CDCl_3$) δ 191.99, 147.60, 144.03, 143.38, 136.94, 134.95, 132.84, 131.19, 130.20, 129.97, 129.12, 128.27, 126.59, 39.34, 37.61, 29.56, 26.05, 22.84. HRMS (ESI+): calcd for $C_{22}H_{24}ClO^+$ (M+H) 339.1510, found 339.1509.

(*E*)-4-(1-(4-chlorophenyl)-2-(1-methylcyclohexyl)vinyl)benzaldehyde (28)

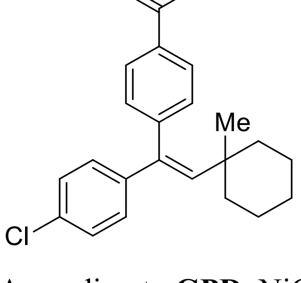

According to **GPD**, $NiCl_2$•DME (4.4 mg, 0.02 mmol, 20 mol%), dtbbpy (5.4 mg, 0.02 mmol, 20 mol%), $Ir[dF(CF_3)ppy]_2(dtbbpy)PF_6$ (3.4 mg, 0.003 mmol, 3 mol%), 4-bromobenzaldehyde (37.0 mg, 0.2 mmol, 2.0 equiv.), cesium 2-((1-methylcyclohexyl)oxy)-2-oxoacetate (47.7 mg, 0.15 mmol, 1.5 equiv.) and 1-chloro-4-ethynylbenzene (13.7 mg, 0.1 mmol, 1.0 equiv.) in DMSO (2 mL) were

used. After 18 hour, the product was isolated by flash chromatography (PE: EA= 60:1) as a pale yellow oil (27.5 mg, 81%, *E:Z* = 92:8). ^{1}H NMR (600 MHz, $CDCl_3$) δ 10.07 (s, 1H), 7.90 (d, *J* = 7.9 Hz, 2H), 7.39 (d, *J* = 7.9 Hz, 2H), 7.23 (d, *J* = 8.5 Hz, 2H), 7.09 (d, *J* = 8.5 Hz, 2H), 6.08 (s, 1H), 1.49-1.40 (m, 7H), 1.28-1.24 (m, 1H), 1.13-1.10 (m, 2H), 1.01 (s, 3H). ^{13}C NMR (150 MHz, $CDCl_3$) δ 191.91, 147.50, 141.98, 140.71, 138.18, 135.24, 132.89, 130.68, 129.41, 128.28, 128.15, 39.38, 37.35, 29.09, 25.99, 22.89. HRMS (ESI+): calcd for $C_{22}H_{24}ClO^+$ (M+H) 339.1510, found 339.1510.

(*E*)-4-(1-(4-Bromophenyl)-2-(1-methylcyclohexyl)vinyl)benzaldehyde (29)

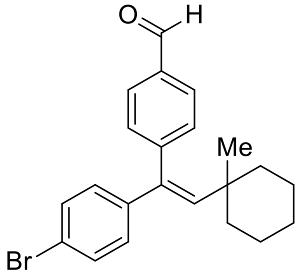

According to **GPD**, $NiCl_2$•DME (4.4 mg, 0.02 mmol, 20 mol%), dtbbpy (5.4 mg, 0.02 mmol, 20 mol%), Ir[dF(CF_3)ppy]$_2$(dtbbpy)PF_6 (3.4 mg, 0.003 mmol, 3 mol%), 4-bromobenzaldehyde (37.0 mg, 0.2 mmol, 2.0 equiv.), cesium 2-((1-methylcyclohexyl)oxy)-2-oxoacetate (47.7 mg, 0.15 mmol, 1.5 equiv.) and 1-bromo-4-ethynylbenzene (18.1 mg, 0.1 mmol, 1.0 equiv.) in DMSO (2 mL) were used. After 18 hour, the product was isolated by flash chromatography (PE: EA= 60:1) as a pale yellow oil (24.9 mg, 65%, *E:Z* = 91:9). ^{1}H NMR (400 MHz, $CDCl_3$) δ 10.04 (s, 1H), 7.87 (d, *J* = 8.1 Hz, 2H), 7.38-7.35 (m, 4H), 7.01 (d, *J* = 8.6 Hz, 2H), 6.06 (s, 1H), 1.45-1.37 (m, 7H), 1.23-1.21 (m, 1H), 1.13-1.07 (m, 2H), 0.99 (s, 3H). ^{13}C NMR (100 MHz, $CDCl_3$) δ 191.95, 147.43, 142.46, 140.81, 138.25, 135.26, 131.25, 130.70, 129.44, 128.52, 121.06, 39.37, 37.38, 29.11, 25.99, 22.90. HRMS (ESI+): calcd for $C_{22}H_{24}BrO^+$ (M+H) 383.1005, found 383.1005.

(*E*)-4-(2-(1-Methylcyclohexyl)-1-(4-(trifluoromethyl)phenyl)vinyl)benzaldehyde (30)

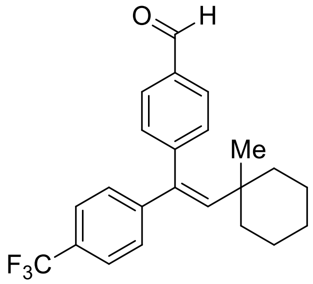

According to **GPD**, $NiCl_2$•DME (4.4 mg, 0.02 mmol, 20 mol%), dtbbpy (5.4 mg, 0.02 mmol, 20 mol%), Ir[dF(CF_3)ppy]$_2$(dtbbpy)PF_6 (3.4 mg, 0.003 mmol, 3 mol%), 4-bromobenzaldehyde (37.0 mg, 0.2 mmol, 2.0 equiv.), cesium 2-((1-methylcyclohexyl)oxy)-2-oxoacetate (47.7 mg, 0.15 mmol, 1.5 equiv.) and 1-ethynyl-4-(trifluoromethyl)benzene (16.3 uL, 0.1 mmol, 1.0 equiv.) in DMSO (2 mL) were used. After 18 hour, the product was isolated by flash chromatography (PE: EA= 60:1) as a pale yellow oil (25.7 mg, 69%, *E:Z* = 94:6). ^{1}H NMR (400 MHz, $CDCl_3$) δ 10.05 (s, 1H), 7.89 (d, *J* = 8.1 Hz, 2H), 7.49 (d, *J* = 8.3 Hz, 2H), 7.39 (d, *J* = 8.1 Hz, 2H), 7.25 (d, *J* = 8.1 Hz, 2H), 6.15 (s, 1H), 1.50-1.39 (m, 7H), 1.24-1.22 (m, 1H), 1.14-1.07 (m, 2H), 1.00 (s, 3H). ^{19}F NMR (565 MHz, $CDCl_3$) δ -62.46 (s). ^{13}C NMR (100 MHz, $CDCl_3$) δ 191.90, 147.06, 146.91, 142.39), 138.24, 135.37, 130.71, 129.51, 128.97 (q, *J* = 32.5 Hz), 127.11, 125.15 (q, *J* = 3.7 Hz), 124.19 (q, *J* = 271.8 Hz), 39.33, 37.52, 28.99, 25.96, 22.90. HRMS (ESI+): calcd for $C_{23}H_{24}F_3O^+$ (M+H) 373.1779, found 373.1774.

(*E*)-4-(2-(1-Methylcyclohexyl)-1-(thiophen-3-yl)vinyl)benzaldehyde (*E*-31)

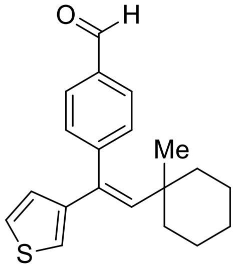

According to **GPD**, $NiCl_2$•DME (4.4 mg, 0.02 mmol, 20 mol%), dtbbpy (5.4 mg, 0.02 mmol, 20 mol%), Ir[dF(CF_3)ppy]$_2$(dtbbpy)PF_6 (3.4 mg, 0.003 mmol, 3 mol%), 4-bromobenzaldehyde (37.0 mg, 0.2 mmol, 2.0 equiv.), cesium 2-((1-methylcyclohexyl)oxy)-2-oxoacetate (47.7 mg, 0.15 mmol, 1.5 equiv.) and

3-ethynylthiophene (9.9 uL, 0.1 mmol, 1.0 equiv.) in DMSO (2 mL) were used. After 18 hour, the product was isolated by flash chromatography (PE: EA= 60:1) as a pale yellow oil (21.4 mg, 69%, *E:Z* = 83:17). ^{1}H NMR (400 MHz, $CDCl_3$) δ 10.05 (s, 1H), 7.89 (d, *J* = 8.2 Hz, 2H), 7.42 (d, *J* = 8.0 Hz, 2H), 7.27-7.25 (m, 2H), 7.19-7.17 (m, 1H), 6.51-6.50 (m, 1H), 6.17 (s, 1H), 1.46-1.38 (m, 7H), 1.23-1.22 (m, 1H), 1.10-1.03 (m, 2H), 0.96 (s, 3H). ^{13}C NMR (100 MHz, $CDCl_3$) δ 192.04, 147.89, 145.21, 138.37, 135.21, 134.34, 130.46, 129.40, 125.61, 125.27, 121.28, 39.48, 37.10, 29.11, 26.01, 22.86. HRMS (ESI+): calcd for $C_{20}H_{23}SO^+$ (M+H) 311.1470, found 311.1467.

(Z)-4-(2-(1-methylcyclohexyl)-1-(thiophen-3-yl)vinyl)benzaldehyde (Z-31)

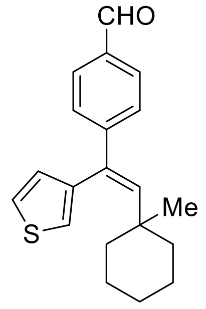

^{1}H NMR (400 MHz, $CDCl_3$) δ 9.96 (s, 1H), 7.75 (d, *J* = 8.2 Hz, 2H), 7.35 (d, *J* = 8.2 Hz, 2H), 7.11-7.10 (m, 1H), 6.89-6.88 (m, 1H), 6.23 (s, 1H), 1.53-1.39 (m, 7H), 1.24-1.18 (m, 1H), 1.15-1.09(m, 2H), 1.03 (s, 3H). ^{13}C NMR (100 MHz, $CDCl_3$) δ 191.98, 149.95, 143.95, 139.42, 134.82, 134.78, 129.72, 129.38, 127.14, 125.32, 123.84, 39.31, 37.66, 29.17, 26.17, 23.18.

(Z)-4-(1-(4-(tert-Butyl)phenyl)-2-(1,4,4-trimethylcyclohexyl)vinyl)benzaldehyde (32)

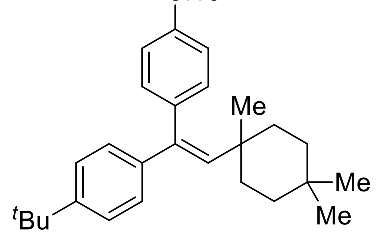

According to **GPD**, $NiCl_2$•DME (4.4 mg, 0.02 mmol, 20 mol%), dtbbpy (5.4 mg, 0.02 mmol, 20 mol%), Ir[dF(CF_3)ppy]$_2$(dtbbpy)PF_6 (3.4 mg, 0.003 mmol, 3 mol%), 4-bromobenzaldehyde (37.0 mg, 0.2 mmol, 2.0 equiv.), cesium

2-oxo-2-((1,4,4-trimethylcyclohexyl)oxy)acetate (51.9 mg, 0.15 mmol, 1.5 equiv.) and 1-(tert-butyl)-4-ethynylbenzene (18.1uL, 0.1 mmol, 1.0 equiv.) in DMSO (2 mL) were used. After 18 hour, the product was isolated by flash chromatography (PE: EA= 60:1) as a pale yellow oil (32.3 mg, 83%, *Z:E* = 95:5). ^{1}H NMR (600 MHz, $CDCl_3$) δ 10.04 (s, 1H), 7.86 (d, *J* = 7.9 Hz, 2H), 7.39 (d, *J* = 7.9 Hz, 2H), 7.27 (d, *J* = 8.4 Hz, 2H), 7.09 (d, *J* = 8.4 Hz, 2H), 6.07 (s, 1H), 1.35-1.33 (m, 2H), 1.30-1.25 (m, 11H), 1.20-1.12 (m, 4H), 0.99 (s, 3H), 0.86 (s, 3H), 0.83 (s, 3H). ^{13}C NMR (100 MHz, $CDCl_3$) δ 192.05, 150.04, 148.16, 140.41, 139.23, 138.98, 135.03, 130.74, 129.30, 126.38, 125.11, 36.90, 35.80, 35.19, 34.42, 31.43, 31.27, 29.51, 29.32. HRMS (ESI+): calcd for $C_{28}H_{37}O^+$ (M+H) 389.2844, found 389.2840.

(Z)-tert-Butyl-4-(2-(4-(tert-butyl)phenyl)-2-(4-formylphenyl)vinyl)-4-methylpiperidine-1-carboxylate (33)

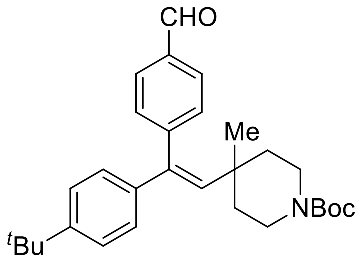

According to **GPD**, $NiCl_2$•DME (4.4 mg, 0.02 mmol, 20 mol%), dtbbpy (5.4 mg, 0.02 mmol, 20 mol%), Ir[dF(CF_3)ppy]$_2$(dtbbpy)PF_6 (3.4 mg, 0.003 mmol, 3 mol%), 4-bromobenzaldehyde (37.0 mg, 0.2 mmol, 2.0 equiv.), cesium 2-((1-(tert-butoxycarbonyl)-4-methylpiperidin-4-yl)oxy)-2-oxoacetate (62.9 mg, 0.15 mmol, 1.5 equiv.) and 1-(tert-butyl)-4-ethynylbenzene (18.1uL, 0.1 mmol, 1.0 equiv.) in DMSO (2 mL) were used. After 18 hour, the product was isolated by flash chromatography (PE: EA= 20:1) as a pale yellow oil (24.0 mg, 52%, *Z:E* = 94:6). ^{1}H NMR (600 MHz, $CDCl_3$) δ 10.05 (s, 1H), 7.89 (d, *J* = 7.6 Hz, 2H), 7.38 (d, *J* = 7.6 Hz, 2H), 7.28 (d, *J* = 8.1 Hz, 2H), 7.07 (d, *J* = 8.0 Hz, 2H), 6.03 (s, 1H), 3.52 (br, 2H), 3.15-3.11 (m, 2H), 1.45-1.43 (m, 11H), 1.29 (s, 9H), 1.21-1.18 (m, 2H), 1.07 (s, 3H). ^{13}C NMR (100 MHz, $CDCl_3$) δ 191.90, 154.91, 150.50, 147.69, 140.38, 139.93, 136.96, 135.27, 130.60, 129.52, 126.44, 125.21, 79.34, 40.79, 38.29, 35.69, 34.46,

31.26, 28.46, 28.31. HRMS (ESI+): calcd for $C_{30}H_{40}NO_3^+$ (M+H) 462.3008, found 462.3002.

(Z)-4-(1-(4-(tert-Butyl)phenyl)-2-(8-methyl-1,4-dioxaspiro[4.5]decan-8-yl)vinyl)benzaldehyde (34)

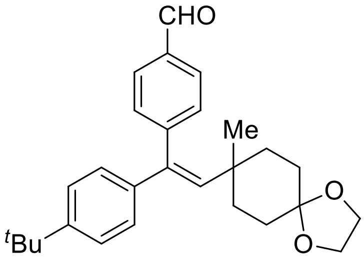

According to **GPD**, $NiCl_2$•DME (4.4 mg, 0.02 mmol, 20 mol%), dtbbpy (5.4 mg, 0.02 mmol, 20 mol%), Ir[dF(CF_3)ppy]$_2$(dtbbpy)PF_6 (3.4 mg, 0.003 mmol, 3 mol%), 4-bromobenzaldehyde (37.0 mg, 0.2 mmol, 2.0 equiv.), cesium 2-((8-methyl-1,4-dioxaspiro[4.5]decan-8-yl)oxy)-2-oxoacetate (56.4 mg, 0.15 mmol, 1.5 equiv.) and 1-(tert-butyl)-4-ethynylbenzene (18.1uL, 0.1 mmol, 1.0 equiv.) in DMSO (2 mL) were used. After 18 hour, the product was isolated by flash chromatography (PE: EA= 20:1) as a colorless oil (33.5 mg, 80%, *Z:E* = 94:6). ^{1}H NMR (400 MHz, $CDCl_3$) δ 10.04 (s, 1H), 7.87 (d, *J* = 8.2 Hz, 2H), 7.40 (d, *J* = 8.0 Hz, 2H), 7.26 (d, *J* = 8.5 Hz, 2H), 7.07 (d, *J* = 8.5 Hz, 2H), 6.06 (s, 1H), 3.91-3.89 (m, 4H), 1.69-1.61 (m, 2H), 1.50-1.48 (m, 4H), 1.38-1.34 (m, 2H), 1.28 (s, 9H), 1.07 (s, 3H). ^{13}C NMR (150 MHz, $CDCl_3$) δ 191.96, 150.25, 147.73, 140.19, 137.61, 135.18, 130.61, 129.35, 126.46, 125.13, 108.69, 64.17, 64.15, 36.51, 36.38, 34.44, 31.79, 31.28, 28.81. HRMS (ESI+): calcd for $C_{28}H_{35}O_3^+$ (M+H) 419.2581. found 419.2578.

(Z)-4-(1-(4-(tert-Butyl)phenyl)-2-(4-methyltetrahydro-2H-pyran-4-yl)vinyl)benzaldehyde (35)

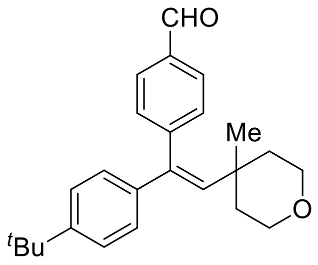

According to **GPD**, $NiCl_2$•DME (4.4 mg, 0.02 mmol, 20 mol%), dtbbpy (5.4 mg, 0.02 mmol, 20 mol%), Ir[dF(CF_3)ppy]$_2$(dtbbpy)PF_6 (3.4 mg, 0.003 mmol, 3 mol%), 4-bromobenzaldehyde (37.0 mg, 0.2 mmol, 2.0 equiv.), cesium 2-((4-methyltetrahydro-2H-pyran-4-yl)oxy)-2-oxoacetate (48.0 mg, 0.15 mmol, 1.5 equiv.) and 1-(tert-butyl)-4-ethynylbenzene (18.1uL, 0.1 mmol, 1.0 equiv.) in DMSO (2 mL) were used. After 18 hour, the product was isolated by flash chromatography (PE: EA= 20:1) as a colorless oil (23.6 mg, 65%, *Z:E* = 93:7).^{1}H NMR (400 MHz, $CDCl_3$) δ 10.04 (s, 1H), 7.88 (d, *J* = 8.1 Hz, 2H), 7.38 (d, *J* = 8.0 Hz, 2H), 7.29 (d, *J* = 8.4 Hz, 2H), 7.10 (d, *J* = 8.5 Hz, 2H), 6.06 (s, 1H), 3.61-3.59 (m, 4H), 1.53-1.47 (m, 2H), 1.34-1.32 (m, 2H), 1.29 (s, 9H), 1.10 (s, 3H). ^{13}C NMR (150 MHz, $CDCl_3$) δ 191.85, 150.46, 147.77, 140.00, 139.95, 137.64, 135.24, 130.65, 129.49, 127.44, 126.44, 125.21, 64.58, 39.19, 35.02, 34.47, 31.27, 28.48. HRMS (ESI+): calcd for $C_{25}H_{31}O_2^+$ (M+H) 363.2319, found 363.2319.

(Z)-4-(1-(4-(tert-Butyl)phenyl)-2-(1-methylcyclopentyl)vinyl)benzaldehyde (36)

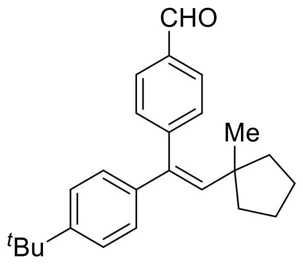

According to **GPD**, $NiCl_2$•DME (4.4 mg, 0.02 mmol, 20 mol%), dtbbpy (5.4 mg, 0.02 mmol, 20 mol%), Ir[dF(CF_3)ppy]$_2$(dtbbpy)PF_6 (3.4 mg, 0.003 mmol, 3 mol%), 4-bromobenzaldehyde (37.0 mg, 0.2 mmol, 2.0 equiv.), cesium 2-((1-methylcyclopentyl)oxy)-2-oxoacetate (45.6 mg, 0.15 mmol, 1.5 equiv.) and 1-(tert-butyl)-4-ethynylbenzene (18.1uL, 0.1 mmol, 1.0 equiv.) in DMSO (2 mL)

were used. After 18 hour, the product was isolated by flash chromatography (PE: EA= 60:1) as a pale yellow oil (27.0 mg, 78%, *Z:E* = 92:8). ^{1}H NMR (400 MHz, $CDCl_3$) δ 10.04 (s, 1H), 7.87 (d, *J* = 8.2 Hz, 2H), 7.39 (d, *J* = 8.0 Hz, 2H), 7.27 (d, *J* = 7.5 Hz, 2H), 7.07 (d, *J* = 8.5 Hz, 2H), 6.27 (s, 1H), 1.57-1.47 (m, 7H), 1.29 (s, 9H), 1.27-1.26 (m, 1H),

0.96 (s, 3H). ^{13}C NMR (150 MHz, $CDCl_3$) δ 192.05, 150.00, 148.33, 140.12, 139.92, 137.97, 135.03, 131.08, 129.25, 126.47, 125.08, 45.09, 40.88, 34.42, 31.28, 26.87, 23.46. HRMS (ESI+): calcd for $C_{25}H_{31}O^+$ (M+H) 347.2369, found 347.2370.

(Z)-4-(1-(4-(tert-Butyl)phenyl)-2-(1-methylcycloheptyl)vinyl)benzaldehyde (37)

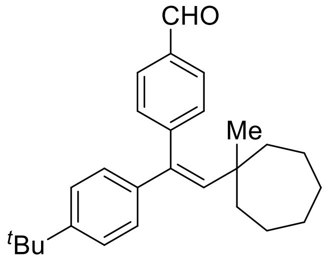

According to **GPD**, $NiCl_2$•DME (4.4 mg, 0.02 mmol, 20 mol%), dtbbpy (5.4 mg, 0.02 mmol, 20 mol%), Ir[dF(CF_3)ppy]$_2$(dtbbpy)PF_6 (3.4 mg, 0.003 mmol, 3 mol%), 4-bromobenzaldehyde (37.0 mg, 0.2 mmol, 2.0 equiv.), cesium 2-((1-methylcycloheptyl)oxy)-2-oxoacetate (49.8 mg, 0.15 mmol, 1.5 equiv.) and 1-(tert-butyl)-4-ethynylbenzene (18.1uL, 0.1 mmol, 1.0 equiv.) in DMSO (2 mL) were used. After 18 hour, the product was isolated by flash chromatography (PE: EA= 60:1) as a pale yellow oil (21.0 mg, 56%, *Z:E* = 93:7).^{1}H NMR (400 MHz, $CDCl_3$) δ 10.04 (s, 1H), 7.86 (d, *J* = 8.0 Hz, 2H), 7.39 (d, *J* = 7.9 Hz, 2H), 7.27 (d, *J* = 8.4 Hz, 2H), 7.08 (d, *J* = 8.4 Hz, 2H), 6.18 (s, 1H), 1.64-1.58 (m, 2H), 1.49-1.38 (m, 10H), 1.28 (s, 9H), 0.86 (s, 3H). ^{13}C NMR (100 MHz, $CDCl_3$) δ 192.05, 149.93, 148.43, 140.56, 140.55, 137.79, 134.94, 130.96, 129.20, 126.43, 125.07, 42.46, 40.14, 34.39, 31.26, 29.88, 29.76, 23.31. HRMS (ESI+): calcd for $C_{27}H_{35}O^+$ (M+H) 375.2688, found 375.2681.

4-((Z)-1-(4-(tert-Butyl)phenyl)-2-((1S,2R,4R)-2-methylbicyclo[2.2.1]heptan-2-yl)vinyl)benzaldehyde (38)

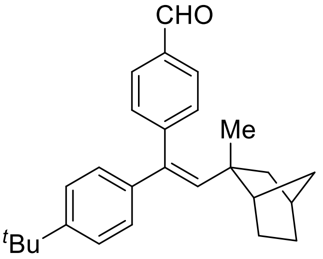

According to **GPD**, $NiCl_2$•DME (4.4 mg, 0.02 mmol, 20 mol%), dtbbpy (5.4 mg, 0.02 mmol, 20 mol%), $Ir[dF(CF_3)ppy]_2(dtbbpy)PF_6$ (3.4 mg, 0.003 mmol, 3 mol%), 4-bromobenzaldehyde (37.0 mg, 0.2 mmol, 2.0 equiv.), cesium 2-(((1R,2S,4S)-2-methylbicyclo[2.2.1]heptan-2-yl)oxy)-2-oxoacetate (49.5 mg, 0.15 mmol, 1.5 equiv.) and 1-(tert-butyl)-4-ethynylbenzene (18.1uL, 0.1 mmol, 1.0 equiv.) in DMSO (2 mL) were used. After 18 hour, the product was isolated by flash chromatography (PE: EA= 60:1) as a pale yellow oil (28.7 mg, 77%, *Z:E* = 91:9). ^{1}H NMR (600 MHz, $CDCl_3$) δ 10.04 (s, 1H), 7.86 (d, J = 7.9 Hz, 2H), 7.38 (d, J = 7.9 Hz, 2H), 7.26 (d, J = 7.6 Hz, 3H), 7.06 (d, J = 8.4 Hz, 2H), 6.18 (s, 1H), 2.11 (br, 1H), 1.96-1.95 (m, 1H), 1.74-1.70 (m, 1H), 1.59-1.54 (m, 2H), 1.44-1.41 (m, 1H), 1.29 (s, 9H), 1.26-1.23 (m, 1H), 1.16-1.15 (m, 1H), 1.05 (s, 3H), 1.03-1.00 (m, 1H), 0.85-0.83 (m, 1H). ^{13}C NMR (150 MHz, $CDCl_3$) δ 192.02, 149.92, 148.45, 143.69, 140.65, 137.40, 134.89, 131.01, 129.40, 126.60, 125.05, 48.67, 47.85, 43.26, 38.88, 37.76, 34.40, 31.28, 28.63, 25.73, 23.93. HRMS (ESI+): calcd for $C_{27}H_{33}O^+$ (M+H) : 373.2526, found : 373.2525.

(Z)-4-(1-(4-(tert-Butyl)phenyl)-2-(2-methyl-2,3-dihydro-1H-inden-2-yl)vinyl)benzaldehyde (39)

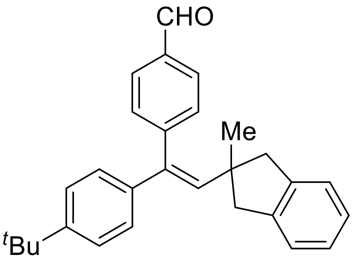

According to **GPD**, $NiCl_2$•DME (4.4 mg, 0.02 mmol, 20 mol%), dtbbpy (5.4 mg, 0.02 mmol, 20 mol%), $Ir[dF(CF_3)ppy]_2(dtbbpy)PF_6$ (3.4 mg, 0.003 mmol, 3 mol%), 4-bromobenzaldehyde (37.0 mg, 0.2 mmol, 2.0 equiv.), cesium 2-((2-methyl-2,3-dihydro-1H-inden-2-yl)oxy)-2-oxoacetate (52.8 mg, 0.15 mmol, 1.5 equiv.) and 1-(tert-butyl)-4-ethynylbenzene (18.1uL, 0.1 mmol, 1.0 equiv.) in DMSO (2 mL) were used. After 18 hour, the product was isolated by flash chromatography (PE: EA= 40:1) as a pale yellow oil (20.5 mg, 52%, *Z:E* = 90:10). ^1H NMR (400 MHz, $CDCl_3$) δ 10.08 (s, 1H), 7.91 (d, *J* = 8.1 Hz, 2H), 7.42 (d, *J* = 8.0 Hz, 2H), 7.28 (d, *J* = 8.5 Hz, 2H), 7.11-7.07 (m, 6H), 6.42 (s, 1H), 2.99-2.95 (m, 2H), 2.55-2.51 (m, 2H), 1.29 (s, 9H), 1.10 (s, 3H). ^{13}C NMR (150 MHz, $CDCl_3$) δ 192.04, 150.29, 147.83, 141.96, 139.72, 138.69, 138.12, 135.19, 131.07, 129.40, 126.53, 126.18, 125.15, 124.60, 47.45, 45.74, 34.44, 31.26, 27.99. HRMS (ESI+): calcd for $C_{29}H_{31}O^+$ (M+H) 395.2375, found 395.2367.

4-((Z)-1-(4-(tert-Butyl)phenyl)-2-((1R,3S,5r,7r)-2-methyladamantan-2-yl)vinyl)benzaldehyde (40)

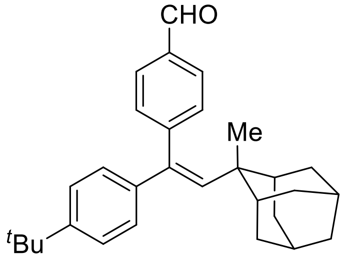

According to **GPD**, $NiCl_2$•DME (4.4 mg, 0.02 mmol, 20 mol%), dtbbpy (5.4 mg, 0.02 mmol, 20 mol%), $Ir[dF(CF_3)ppy]_2(dtbbpy)PF_6$ (3.4 mg, 0.003 mmol, 3 mol%), 4-bromobenzaldehyde (37.0 mg, 0.2 mmol, 2.0 equiv.), cesium 2-(((1R,3S,5r,7r)-2-methyladamantan-2-yl)oxy)-2-oxoacetate (55.5 mg, 0.15 mmol, 1.5 equiv.) and 1-(tert-butyl)-4-ethynylbenzene (18.1uL, 0.1 mmol, 1.0 equiv.) in DMSO (2 mL) were used. After 18 hour, the product was isolated by flash chromatography (PE: EA= 60:1) as a pale yellow oil (28.9 mg, 70%, *Z:E* = 94:6). ^1H NMR (400 MHz, $CDCl_3$) δ 10.04 (s, 1H), 7.85 (d, *J* = 8.2 Hz, 2H), 7.42 (d, *J* = 8.1 Hz, 2H), 7.27 (d, *J* = 9.6 Hz, 2H), 7.10 (d, *J* = 8.5 Hz, 2H), 6.13 (s, 1H), 2.10-2.08 (m,

2H), 1.94-1.91 (m, 2H), 1.80-1.72 (m, 2H), 1.61 (br, 2H), 1.48-1.41 (m, 4H), 1.34 (br, 2H), 1.29 (s, 9H), 1.26 (s, 3H). ^{13}C NMR (150 MHz, $CDCl_3$) δ 192.11, 149.89, 148.29, 142.11, 140.71, 136.91, 134.99, 130.50, 129.30, 126.35, 125.11, 42.21, 38.51, 36.50, 34.76, 34.42, 32.56, 31.29, 27.54, 27.36, 26.18. HRMS (ESI+): calcd for $C_{30}H_{37}O^+$ (M+H) 413.2839, found 413.2840.

(Z)-4-(1-(4-(tert-Butyl)phenyl)-3,3-dimethylbut-1-en-1-yl)benzaldehyde (41)

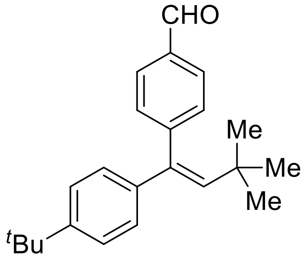

According to **GPD**, $NiCl_2$•DME (4.4 mg, 0.02 mmol, 20 mol%), dtbbpy (5.4 mg, 0.02 mmol, 20 mol%), Ir[dF(CF_3)ppy]$_2$(dtbbpy)PF_6 (3.4 mg, 0.003 mmol, 3 mol%), 4-bromobenzaldehyde (37.0 mg, 0.2 mmol, 2.0 equiv.), cesium 2-(tert-butoxy)-2-oxoacetate (41.7 mg, 0.15 mmol, 1.5 equiv.) and 1-(tert-butyl)-4-ethynylbenzene (18.1uL, 0.1 mmol, 1.0 equiv.) in DMSO (2 mL) were used. After 18 hour, the product was isolated by flash chromatography (PE: EA= 60:1) as a pale yellow solid (27.2 mg, 85%, *Z:E* = 94:6). ^{1}H NMR (600 MHz, $CDCl_3$) δ 10.07 (s, 1H), 7.90 (d, *J* = 8.0 Hz, 2H), 7.41 (d, *J* = 7.9 Hz, 2H), 7.29 (d, *J* = 8.5 Hz, 2H), 7.10 (d, *J* = 8.4 Hz, 2H), 6.15 (s, 1H), 1.31 (s, 9H), 0.98 (s, 9H). ^{13}C NMR (150 MHz, $CDCl_3$) δ 192.04, 150.02, 148.07, 140.26, 140.11, 137.53, 135.03, 131.11, 129.25, 126.38, 125.10, 34.43, 34.04, 31.34, 31.29. HRMS (ESI+): calcd for $C_{23}H_{29}O^+$ (M+H) 321.2213, found 321.2212.

(Z)-4-(1-(4-(tert-Butyl)phenyl)-3-ethyl-3,7-dimethyloct-1-en-1-yl)benzaldehyde (42)

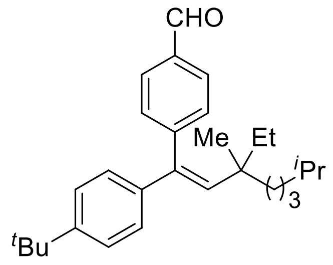

According to **GPD**, $NiCl_2$•DME (4.4 mg, 0.02 mmol, 20 mol%), dtbbpy (5.4 mg, 0.02 mmol, 20 mol%), Ir[dF(CF_3)ppy]$_2$(dtbbpy)PF_6 (3.4 mg, 0.003 mmol, 3 mol%), 4-bromobenzaldehyde (37.0 mg, 0.2 mmol, 2.0 equiv.), cesium 2-((3,7-dimethyloctan-3-yl)oxy)-2-oxoacetate (54.3 mg, 0.15 mmol, 1.5 equiv.) and 1-(tert-butyl)-4-ethynylbenzene (18.1uL, 0.1 mmol, 1.0 equiv.) in DMSO (2 mL) were used. After 18 hour, the product was isolated by flash chromatography (PE: EA= 60:1) as a pale yellow oil (34.4 mg, 85%, *Z:E* = 95:5). ^{1}H NMR (600 MHz, $CDCl_3$) δ 10.04 (s, 1H), 7.86 (d, *J* = 7.8 Hz, 2H), 7.37 (d, *J* = 7.7 Hz, 2H), 7.27 (d, *J* = 8.3 Hz, 2H), 7.08 (d, *J* = 8.2 Hz, 2H), 5.95 (s, 1H), 1.55-1.48 (m, 1H), 1.38-1.35 (m, 1H), 1.28 (s, 9H), 1.27-1.26 (m, 4H), 1.22-1.18 (m, 1H), 1.09-1.06 (m, 2H), 0.87-0.84 (m, 9H), 0.66 (s, 3H). ^{13}C NMR (150 MHz, $CDCl_3$) δ 192.05, 149.94, 148.25, 140.44, 138.85, 138.70, 134.96, 130.89, 129.16, 126.39, 125.10, 43.00, 40.71, 39.77, 35.58, 34.42, 31.29, 27.97, 24.25, 22.72, 22.69, 22.33, 8.99. HRMS (ESI+): calcd for $C_{29}H_{41}O^+$ (M+H) 405.3157, found 405.3151.

(Z)-4-(1-(4-(tert-Butyl)phenyl)-5-((tert-butyldimethylsilyl)oxy)-3,3-dimethylpent-1-en-1-yl)benzaldehyde (43)

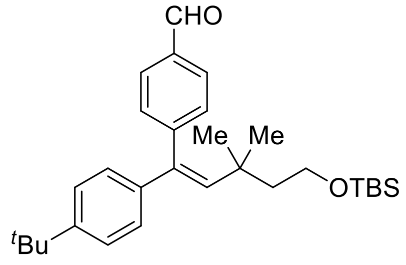

According to **GPD**, $NiCl_2$•DME (4.4 mg, 0.02 mmol, 20 mol%), dtbbpy (5.4 mg, 0.02 mmol, 20 mol%), Ir[dF(CF_3)ppy]$_2$(dtbbpy)PF_6 (3.4 mg, 0.003 mmol, 3 mol%), 4-bromobenzaldehyde (37.0 mg, 0.2 mmol, 2.0

equiv.),cesium-2-((4-((tert-butyldimethylsilyl)oxy)-2-methylbutan-2-yl)oxy)-2-oxoacetate (63.3 mg, 0.15 mmol, 1.5 equiv.) and 1-(tert-butyl)-4-ethynylbenzene (18.1uL, 0.1 mmol,1.0 equiv.) in DMSO (2 mL) were used. After 18 hour, the product was isolated by flash chromatography (PE: EA= 60:1) as a pale yellow oil (30.2 mg, 65%, *Z:E* = 94:6). ^{1}H NMR (600 MHz, $CDCl_3$) δ 10.04 (s, 1H), 7.86 (d, *J* = 7.8 Hz, 2H), 7.37 (d, *J* = 7.7 Hz, 2H), 7.26 (d, *J* = 7.1 Hz, 2H), 7.06 (d, *J* = 8.2 Hz, 2H), 6.07 (s, 1H), 3.69 (t, *J* = 7.5 Hz, 2H), 1.60 (t, *J* = 7.5 Hz, 2H), 1.28 (s, 9H), 0.89 (s, 6H), 0.88 (s, 9H), 0.05 (s, 6H). ^{13}C NMR (100 MHz, $CDCl_3$) δ 192.00, 150.09, 147.91, 140.06, 138.85, 138.26, 135.04, 130.98, 129.24, 126.38, 125.09, 60.47, 47.42, 36.19, 34.41, 31.26, 29.29, 25.94, 18.28, -5.23. HRMS (ESI+): calcd for $C_{30}H_{45}O_2Si^+$ (M+H) : 465.3183, found : 465.3186.

4-((*Z*)-1-(4-(tert-Butyl)phenyl)-2-((3S,3aR,5R,7S)-3,5,8,8-tetramethyloctahydro-1H-3a,7-methanoazulen-5-yl)vinyl)benzaldehyde (44)

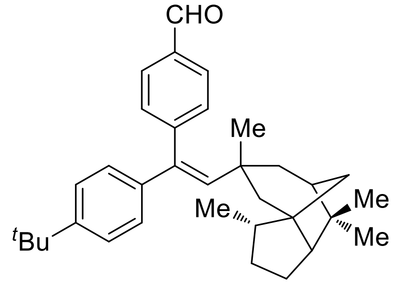

According to **GPD**, $NiCl_2$•DME (4.4 mg, 0.02 mmol, 20 mol%), dtbbpy (5.4 mg, 0.02 mmol, 20 mol%), Ir[dF(CF_3)ppy]$_2$(dtbbpy)PF_6 (3.4 mg, 0.003 mmol, 3 mol%), 4-bromobenzaldehyde (37.0 mg, 0.2 mmol, 2.0 equiv.), cesium 2-oxo-2-(((3S,3aR,5R,7R)-3,5,8,8-tetramethyloctahydro-1H-3a,7-methanoazulen-5-yl)oxy)acetate (63.9 mg, 0.15 mmol, 1.5 equiv.) and 1-(tert-butyl)-4-ethynylbenzene (18.1uL, 0.1 mmol, 1.0 equiv.) in DMSO (2 mL) were used. After 18 hour, the product was isolated by flash chromatography (PE: EA= 60:1) as a pale yellow oil (25.3 mg, 54%, *Z:E* = 94:6). ^{1}H NMR (400 MHz, $CDCl_3$) δ 10.05 (s, 1H), 7.87 (d, *J* = 8.2 Hz, 2H), 7.40 (d, *J* = 8.0 Hz, 2H), 7.27 (d, *J* = 8.7 Hz, 2H), 7.09 (d, *J* = 8.5 Hz, 2H), 6.11 (s, 1H), 1.88-1.81 (m, 1H), 1.71-1.58 (m, 4H), 1.56-1.40 (m, 5H), 1.37-1.32 (m, 2H), 1.29 (s, 9H), 1.19 (s, 3H), 1.11 (s, 3H), 0.87 (s, 3H), 0.81 (d, *J* = 7.1 Hz, 3H).

^{13}C NMR (150 MHz, $CDCl_3$) δ 192.07, 149.85, 148.43, 142.62, 140.60, 135.73, 134.96, 130.68, 129.30, 126.30, 125.11, 60.30, 57.69, 53.71, 43.97, 43.20, 42.78, 41.80, 36.91, 34.42, 33.11, 31.53, 31.29, 29.59, 29.03, 28.30, 25.41, 15.48. HRMS (ESI+): calcd for $C_{34}H_{45}O^+$ (M+H) : 469.3465, found : 469.3475.

(Z)-1-(4-(1-(4-(tert-Butyl)phenyl)-2-(1-methylcyclohexyl)vinyl)phenyl)ethenone (45)

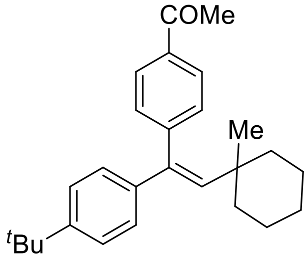

According to **GPD**, $NiCl_2$•DME (4.4 mg, 0.02 mmol, 20 mol%), dtbbpy (5.4 mg, 0.02 mmol, 20 mol%), Ir[dF(CF_3)ppy]$_2$(dtbbpy)PF_6 (3.4 mg, 0.003 mmol, 3 mol%), 1-(4-bromophenyl)ethan-1-one (39.8 mg, 0.2 mmol, 2.0 equiv.), cesium 2-((1-methylcyclohexyl)oxy)-2-oxoacetate (47.7 mg, 0.15 mmol, 1.5 equiv.) and 1-(tert-butyl)-4-ethynylbenzene (18.1uL, 0.1 mmol, 1.0 equiv.) in DMSO (2 mL) were used. After 18 hour, the product was isolated by flash chromatography (PE: EA= 60:1) as a pale yellow oil (32.6 mg, 87%, *Z:E* = 91:9). ^{1}H NMR (600 MHz, $CDCl_3$) δ 7.97 (d, *J* = 8.2 Hz, 2H), 7.35 (d, *J* = 8.2 Hz, 2H), 7.29 (d, *J* = 8.2 Hz, 2H), 7.12 (d, *J* = 8.5 Hz, 2H), 6.10 (s, 1H), 2.66 (s, 3H), 1.51-1.40 (m, 8H), 1.31 (s, 9H), 1.11-1.07 (m, 2H), 1.00 (s, 3H). ^{13}C NMR (150 MHz, $CDCl_3$) δ 197.99, 149.91, 146.72, 140.62, 139.14, 138.94, 135.64, 130.30, 127.94, 126.36, 125.07, 39.50, 37.19, 34.43, 31.31, 29.43, 26.68, 26.09, 22.94. HRMS (ESI+): calcd for $C_{27}H_{35}O^+$ (M+H) 375.2688, found 375.2685.

(Z)-(4-(1-(4-(tert-Butyl)phenyl)-2-(1-methylcyclohexyl)vinyl)phenyl)(phenyl) Methanone (46)

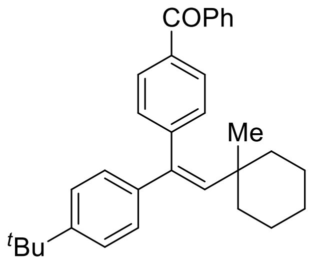

According to **GPD**, $NiCl_2$•DME (4.4 mg, 0.02 mmol, 20 mol%), dtbbpy (5.4 mg, 0.02 mmol, 20 mol%), Ir[dF(CF_3)ppy]$_2$(dtbbpy)PF_6 (3.4 mg, 0.003 mmol, 3 mol%), (4-bromophenyl)(phenyl)methanone (52.2 mg, 0.2 mmol, 2.0 equiv.), cesium 2-((1-methylcyclohexyl)oxy)-2-oxoacetate (47.7 mg, 0.15 mmol, 1.5 equiv.) and 1-(tert-butyl)-4-ethynylbenzene (18.1uL, 0.1 mmol, 1.0 equiv.) in DMSO (2 mL) were used. After 18 hour, the product was isolated by flash chromatography (PE: EA= 60:1) as a pale yellow oil (32.7 mg, 75%, *Z:E* = 92:8). ^{1}H NMR (400 MHz, $CDCl_3$) δ 7.84-7.80 (m, 4H), 7.61-7.58 (m, 1H), 7.52-7.48 (m, 2H), 7.34 (d, *J* = 8.2 Hz, 2H), 7.29 (d, *J* = 8.5 Hz, 2H), 7.14 (d, *J* = 8.5 Hz, 2H), 6.09 (s, 1H), 1.47-1.41 (m, 7H), 1.30 (s, 9H), 1.23-1.21 (m, 1H), 1.14-1.06 (m, 2H), 1.00 (s, 3H). ^{13}C NMR (150 MHz, $CDCl_3$) δ 196.56, 149.92, 146.08, 140.70, 139.21, 139.02, 137.76, 135.90, 132.34, 130.02 (2c), 129.77, 128.29, 126.43, 125.07, 39.56, 37.22, 34.44, 31.32, 29.39, 26.11, 22.96. HRMS (ESI+): calcd for $C_{32}H_{37}O^+$ (M+H) 437.2839, found 437.2876.

(Z)-Methyl-4-(1-(4-(tert-butyl)phenyl)-2-(1-methylcyclohexyl)vinyl)benzoate (Z-47)

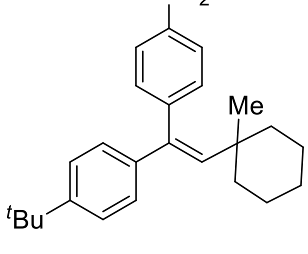

According to **GPD**, $NiCl_2$•DME (4.4 mg, 0.02 mmol, 20 mol%), dtbbpy (5.4 mg, 0.02 mmol, 20 mol%), Ir[dF(CF_3)ppy]$_2$(dtbbpy)PF_6 (3.4 mg, 0.003 mmol, 3 mol%), methyl 4-bromobenzoate (43.0 mg, 0.2 mmol, 2.0 equiv.), cesium

2-((1-methylcyclohexyl)oxy)-2-oxoacetate (47.7 mg, 0.15 mmol, 1.5 equiv.) and 1-(tert-butyl)-4-ethynylbenzene (18.1uL, 0.1 mmol, 1.0 equiv.) in DMSO (2 mL) were used. After 18 hour, the product was isolated by flash chromatography (PE: EA= 60:1) as a pale yellow oil (32.4 mg, 83%, *Z:E* = 88:12). ^{1}H NMR (400 MHz, $CDCl_3$) δ 8.01 (d, *J* = 8.1 Hz, 2H), 7.30-7.25 (m, 4H), 7.09 (d, *J* = 8.4 Hz, 2H), 6.06 (s, 1H), 3.93 (s, 3H), 1.46-1.39 (m, 8H), 1.28 (s, 9H), 1.09-1.01 (m, 2H), 0.97 (s, 3H). ^{13}C NMR (100 MHz, $CDCl_3$) δ 167.12, 149.88, 146.44, 140.68, 139.08, 139.04, 130.14, 129.14, 128.66, 126.38, 125.04, 52.09, 39.51, 37.18, 34.41, 31.30, 29.32, 26.10, 22.94. HRMS (ESI+): calcd for $C_{27}H_{35}O_2^+$ (M+H) 391.2632, found 391.2629.

Methyl(*E*)-4-(1-(4-(tert-butyl)phenyl)-2-(1-methylcyclohexyl)vinyl)benzoate (*E*-47)

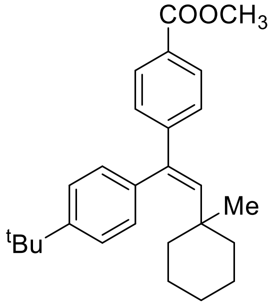

^{1}H NMR (400 MHz, $CDCl_3$) δ 7.88 (d, *J* = 8.5 Hz, 2H), 7.34 (d, *J* = 8.3 Hz, 2H), 7.24 (d, *J* = 8.5 Hz, 2H), 7.08 (d, *J* = 8.3 Hz, 2H), 6.10 (s, 1H), 3.88 (s, 3H), 1.50-1.39 (m, 8H), 1.34 (s, 9H), 1.10-1.03 (m, 2H), 0.99 (s, 3H). ^{13}C NMR (100 MHz, $CDCl_3$) δ 167.20, 150.10, 149.32, 141.33, 139.89, 137.06, 129.55, 129.39, 128.05, 126.86, 124.82, 52.06, 39.49, 37.49, 34.63, 31.51, 29.32, 26.21, 23.12. HRMS (ESI+): calcd for $C_{27}H_{35}O_2^+$ (M+H) 391.2632, found 391.2629.

(Z)-4-(1-(4-(Tert-butyl)phenyl)-2-(1-methylcyclohexyl)vinyl)benzonitrile (48)

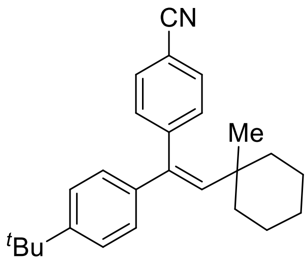

According to **GPD**, $NiCl_2$•DME (4.4 mg, 0.02 mmol, 20 mol%), dtbbpy (5.4 mg, 0.02 mmol, 20 mol%), Ir[dF(CF_3)ppy]$_2$(dtbbpy)PF_6 (3.4 mg, 0.003 mmol, 3 mol%), methyl 4-bromobenzonitrile (36.4 mg, 0.2 mmol, 2.0 equiv.), cesium 2-((1-methylcyclohexyl)oxy)-2-oxoacetate (47.7 mg, 0.15 mmol, 1.5 equiv.) and 1-(tert-butyl)-4-ethynylbenzene (18.1uL, 0.1 mmol, 1.0 equiv.) in DMSO (2 mL) were used. After 18 hour, the product was isolated by flash chromatography (PE: EA= 60:1) as a pale yellow oil (32.5 mg, 91%, *Z:E* = 91:9). ^{1}H NMR (600 MHz, $CDCl_3$) δ 7.63 (d, *J* = 8.2 Hz, 2H), 7.33 (d, *J* = 8.2 Hz, 2H), 7.27 (d, *J* = 8.5 Hz, 2H), 7.05 (d, *J* = 8.5 Hz, 2H), 6.08 (s, 1H), 1.41-1.39 (m, 7H), 1.29 (s, 9H), 1.24-1.18 (m, 1H), 1.10-1.05 (m, 2H), 0.96 (s, 3H). ^{13}C NMR (150 MHz, $CDCl_3$) δ 150.19, 146.57, 140.19, 139.74, 138.30, 131.66, 130.85, 126.39, 125.16, 118.99, 110.76, 39.50, 37.22, 34.45, 31.28, 29.26, 26.01, 22.87. HRMS (ESI+): calcd for $C_{26}H_{32}N^+$ (M+H) 358.2529, found 358.2530.

(Z)-Methyl4-(1-(4-(tert-butyl)phenyl)-2-(1-methylcyclohexyl)vinyl)-2-fluorobenzoate (Z-49)

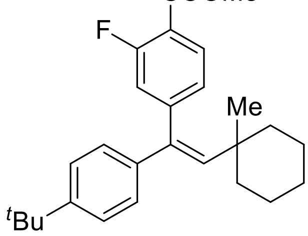

According to **GPD**, $NiCl_2$•DME (4.4 mg, 0.02 mmol, 20 mol%), dtbbpy (5.4 mg, 0.02 mmol, 20 mol%), Ir[dF(CF_3)ppy]$_2$(dtbbpy)PF_6 (3.4 mg, 0.003 mmol, 3 mol%), methyl 4-bromo-2-fluorobenzoate (46.6 mg, 0.2 mmol, 2.0 equiv.), cesium

2-((1-methylcyclohexyl)oxy)-2-oxoacetate (47.7 mg, 0.15 mmol, 1.5 equiv.) and 1-(tert-butyl)-4-ethynylbenzene (18.1uL, 0.1 mmol, 1.0 equiv.) in DMSO (2 mL) were used. After 18 hour, the product was isolated by flash chromatography (PE: EA= 60:1) as a pale yellow oil (30.6 mg, 75%, *Z:E* = 88:12). ^{1}H NMR (400 MHz, $CDCl_3$) δ 7.91 (t, *J* = 7.8 Hz, 1H), 7.28-7.26 (m, 2H), 7.09-7.07 (m, 3H), 7.03-7.00 (m, 1H), 6.06 (s, 1H), 3.95 (s, 3H), 1.45-1.38 (m, 7H), 1.29 (s, 9H), 1.23-1.21 (m, 1H), 1.13-1.08 (m, 2H), 0.98 (s, 3H). ^{19}F NMR (565 MHz, $CDCl_3$) δ -109.98 (dd, *J* = 11.3, 7.7 Hz). ^{13}C NMR (150 MHz, $CDCl_3$) δ 164.94, 161.51 (d, *J* = 260.6 Hz), 150.18, 148.68 (d, *J* = 8.6 Hz), 140.08, 139.69, 137.86, 131.55, 126.38, 125.95 (d, *J* = 3.3 Hz), 125.16, 118.54 (d, *J* = 22.2 Hz), 116.99 (d, *J* = 9.8 Hz), 52.36, 39.50, 37.25, 34.47, 31.32, 29.17, 26.07, 22.92. HRMS (ESI+): calcd for $C_{27}H_{34}FO_2^+$ (M+H) 409.2537, found 409.2538.

Methyl(*E*)-4-(1-(4-(tert-butyl)phenyl)-2-(1-methylcyclohexyl)vinyl)-2-fluorobenzoate (*E*-49)

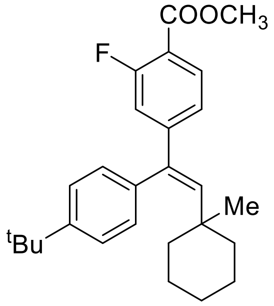

^{1}H NMR (400 MHz, $CDCl_3$) δ 7.79 (t, *J* = 8.0 Hz, 1H), 7.35 (d, *J* = 8.2 Hz, 2H), 7.07 (d, *J* = 8.2 Hz, 2H), 7.05-7.02 (m, 1H), 6.94-6.90 (m, 1H), 6.14 (s, 1H), 3.90 (s, 3H), 1.50-1.38 (m, 7H), 1.35 (s, 9H), 1.22-1.18 (m, 1H), 1.10-1.03 (m, 2H), 0.98 (s, 3H). ^{19}F NMR (375 MHz, $CDCl_3$) δ -110.11 (dd, *J* = 12.8, 7.8 Hz). 13C NMR (100 MHz, CDCl3) δ 164.94 (d, J = 3.9 Hz), 161.85 (d, J = 258.5 Hz), 151.47 (d, J = 8.3 Hz), 150.34, 142.26, 138.83, 136.24, 131.62, 129.43, 124.91, 122.19, 115.99 (d, J = 10.4 Hz), 115.07 (d, J = 23.5 Hz), 52.18, 39.32, 37.49, 34.58, 31.42, 29.08, 26.06, 23.02.

(Z)-4-(1-(4-(Tert-Butyl)phenyl)-2-(1-methylcyclohexyl)vinyl)-1,1'-biphenyl (50)

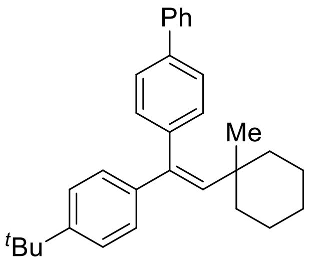

According to **GPD**, $NiCl_2$•DME (4.4 mg, 0.02 mmol, 20 mol%), dtbbpy (5.4 mg, 0.02 mmol, 20 mol%), $Ir[dF(CF_3)ppy]_2(dtbbpy)PF_6$ (3.4 mg, 0.003 mmol, 3 mol%), 4-bromo-1,1'-biphenyl (46.6 mg, 0.2 mmol, 2.0 equiv.), cesium 2-((1-methylcyclohexyl)oxy)-2-oxoacetate (47.7 mg, 0.15 mmol, 1.5 equiv.) and 1-(tert-butyl)-4-ethynylbenzene (18.1uL, 0.1 mmol, 1.0 equiv.) in DMSO (2 mL) were used. After 18 hour, the product was isolated by flash chromatography (PE: EA= 60:1) as a pale yellow oil (31.1 mg, 76%, *Z:E* = 90:10). ^{1}H NMR (400 MHz, $CDCl_3$) δ 7.66-7.64 (m, 2H), 7.59-7.57 (m, 2H), 7.46-7.42 (m, 2H), 7.35-7.34 (m, 1H), 7.29-7.26 (m, 4H), 7.19-7.17 (m, 2H), 6.06 (s, 1H), 1.50-1.40 (m, 8H), 1.29 (s, 9H), 1.10-1.03 (m, 2H), 1.01 (s, 3H). ^{13}C NMR (100 MHz, $CDCl_3$) δ 149.62, 141.44, 140.90, 140.22, 139.63, 139.40, 138.67, 130.44, 128.79, 127.23, 127.02, 126.45, 126.42, 124.99, 39.61, 37.21, 34.43, 31.37, 29.58, 26.23, 23.06. HRMS (ESI+): calcd for $C_{31}H_{37}^+$ (M+H) 409.2890, found 409.2877.

(Z)-5-(1-(4-(tert-Butyl)phenyl)-2-(1-methylcyclohexyl)vinyl)-2,3-dihydro-1H-inden-1-one (51)

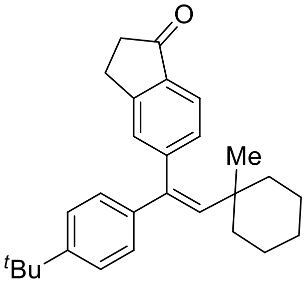

According to **GPD**, $NiCl_2$•DME (4.4 mg, 0.02 mmol, 20 mol%), dtbbpy (5.4 mg, 0.02 mmol, 20 mol%), $Ir[dF(CF_3)ppy]_2(dtbbpy)PF_6$ (3.4 mg, 0.003 mmol, 3 mol%), 5-bromo-2,3-dihydro-1H-inden-1-one (39.8 mg, 0.2 mmol, 2.0 equiv.), cesium 2-((1-methylcyclohexyl)oxy)-2-oxoacetate (47.7 mg, 0.15 mmol, 1.5 equiv.) and

1-(tert-butyl)-4-ethynylbenzene (18.1uL, 0.1 mmol, 1.0 equiv.) in DMSO (2 mL) were used. After 18 hour, the product was isolated by flash chromatography (PE: EA= 60:1) as a pale yellow oil (20.9 mg, 54%, *Z:E* = 91:9). ^{1}H NMR (600 MHz, $CDCl_3$) δ 7.73 (d, *J* = 7.7 Hz, 1H), 7.32 (s, 1H), 7.28-7.25 (m, 2H), 7.23 (d, *J* = 7.8 Hz, 1H), 7.10 (d, *J* = 8.3 Hz, 2H), 6.06 (s, 1H), 3.15-3.13 (m, 2H), 2.73-2.71 (m, 2H), 1.46-1.39 (m, 7H), 1.29 (s, 9H), 1.23-1.21 (m, 1H), 1.09-1.06 (m, 2H), 0.98 (s, 3H). ^{13}C NMR (150 MHz, $CDCl_3$) δ 206.72, 154.86, 149.95, 148.45, 140.64, 139.18, 139.14, 135.79, 129.55, 128.02, 126.40, 125.08, 123.08, 39.53, 37.21, 36.42, 34.43, 31.30, 29.34, 26.08, 25.80, 22.93. HRMS (ESI+): calcd for $C_{28}H_{35}O^+$ (M+H) 387.2682, found 387.2681.

(Z)-5-(1-(4-(tert-Butyl)phenyl)-2-(1-methylcyclohexyl)vinyl)isobenzofuran-1(3H)-one (Z-52)

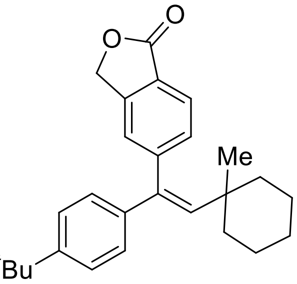

According to **GPD**, $NiCl_2$•DME (4.4 mg, 0.02 mmol, 20 mol%), dtbbpy (5.4 mg, 0.02 mmol, 20 mol%), Ir[dF(CF_3)ppy]$_2$(dtbbpy)PF_6 (3.4 mg, 0.003 mmol, 3 mol%), 5-bromoisobenzofuran-1(3H)-one (42.6 mg, 0.2 mmol, 2.0 equiv.), cesium 2-((1-methylcyclohexyl)oxy)-2-oxoacetate (47.7 mg, 0.15 mmol, 1.5 equiv.) and 1-(tert-butyl)-4-ethynylbenzene (18.1uL, 0.1 mmol, 1.0 equiv.) in DMSO (2 mL) were used. After 18 hour, the product was isolated by flash chromatography (PE: EA= 60:1) as a pale yellow oil (29.1 mg, 75%, *Z:E* = 84:16). ^{1}H NMR (400 MHz, $CDCl_3$) δ 7.90 (d, *J* = 7.8 Hz, 1H), 7.41 (d, *J* = 7.7 Hz, 1H), 7.33 (s, 1H), 7.28 (d, *J* = 8.5 Hz, 2H), 7.08 (d, *J* = 8.5 Hz, 2H), 6.10 (s, 1H), 5.32 (s, 2H), 1.44-1.39 (m, 8H), 1.29 (s, 9H), 1.13-1.04 (m, 2H), 0.97 (s, 3H). ^{13}C NMR (100 MHz, $CDCl_3$) δ 171.01, 150.22, 148.06, 146.35, 140.40, 139.83, 138.55, 131.27, 129.43, 126.44, 125.18, 124.39,

123.40, 69.57, 39.51, 37.24, 34.46, 31.29, 29.27, 26.01, 22.87. HRMS (ESI+): calcd for $C_{27}H_{32}O_2^+$ (M+H) 389.2475, found 389.2476.

(*E*)-5-(1-(4-(tert-butyl)phenyl)-2-(1-methylcyclohexyl)vinyl)isobenzofuran-1(3H)-one (*E*-52)

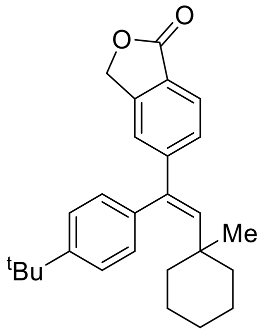

^{1}H NMR (400 MHz, $CDCl_3$) δ 7.77 (d, *J* = 8.1 Hz, 1H), 7.42 (d, *J* = 8.1 Hz, 1H), 7.36 (d, *J* = 8.2 Hz, 2H), 7.22 (s, 1H), 7.09 (d, *J* = 8.2 Hz, 2H), 6.13 (s, 1H), 5.23 (s, 2H), 1.49-1.38 (m, 8H), 1.35 (s, 9H), 1.12-1.05 (m, 2H), 1.00 (s, 3H). ^{13}C NMR (100 MHz, $CDCl_3$) δ 171.25, 151.12, 150.47, 146.81, 142.84, 139.78, 136.71, 129.51, 128.15, 125.28, 125.04, 123.72, 120.37, 69.68, 39.43, 37.68, 34.67, 31.49, 29.79, 26.15, 23.14.

(Z)-2-(1-(4-(tert-Butyl)phenyl)-2-(1-methylcyclohexyl)vinyl)benzo[d]thiazole (53)

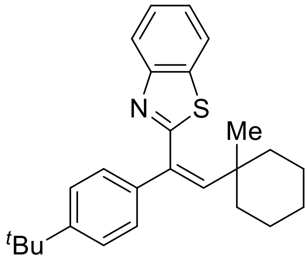

According to **GPD**, $NiCl_2$•DME (4.4 mg, 0.02 mmol, 20 mol%), dtbbpy (5.4 mg, 0.02 mmol, 20 mol%), Ir[dF(CF_3)ppy]$_2$(dtbbpy)PF_6 (3.4 mg, 0.003 mmol, 3 mol%), 2-chlorobenzo[*d*]thiazole (26.0 uL, 0.2 mmol, 2.0 equiv.), cesium 2-((1-methylcyclohexyl)oxy)-2-oxoacetate (47.7 mg, 0.15 mmol, 1.5 equiv.) and 1-(tert-butyl)-4-ethynylbenzene (18.1uL, 0.1 mmol, 1.0 equiv.) in DMSO (2 mL) were used. After 18 hour, the product was isolated by flash chromatography (PE: EA=

60:1) as a pale yellow oil (24.5 mg, 63%, *Z:E* = 97:3). ^{1}H NMR (400 MHz, $CDCl_3$) δ 8.11 (d, *J* = 8.1 Hz, 1H), 7.86 (d, *J* = 7.8 Hz, 1H), 7.52-7.48 (m, 1H), 7.42-7.38 (m, 1H), 7.31-7.28 (m, 2H), 7.24-7.22 (m, 2H), 6.27 (s, 1H), 1.59-1.55(m, 3H), 1.51-1.44 (m, 5H), 1.29 (s, 9H), 1.22-1.18 (m, 2H), 1.16 (s, 3H). ^{13}C NMR (100 MHz, $CDCl_3$) δ 167.76, 153.04, 150.60, 145.29, 138.92, 136.42, 133.03, 126.67, 125.92, 125.25,125.17, 123.58, 121.51, 39.23, 37.80, 34.53, 31.33, 28.35, 26.14, 23.10. HRMS (ESI+): calcd for $C_{26}H_{32}NS^+$ (M+H) 390.2250, found 390.2250.

(Z)-2-(1-(4-(tert-Butyl)phenyl)-2-(1-methylcyclohexyl)vinyl)-6-chlorobenzo[d] thiazole (54)

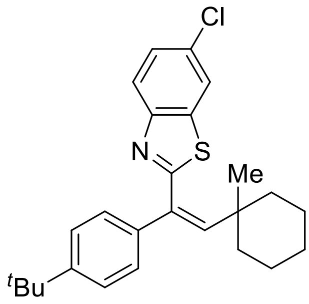

According to **GPD**, $NiCl_2$•DME (4.4 mg, 0.02 mmol, 20 mol%), dtbbpy (5.4 mg, 0.02 mmol, 20 mol%), Ir[dF(CF_3)ppy]$_2$(dtbbpy)PF_6 (3.4 mg, 0.003 mmol, 3 mol%), 2,6-dichlorobenzo[*d*]thiazole (40.8 mg, 0.2 mmol, 2.0 equiv.), cesium 2-((1-methylcyclohexyl)oxy)-2-oxoacetate (47.7 mg, 0.15 mmol, 1.5 equiv.) and 1-(tert-butyl)-4-ethynylbenzene (18.1uL, 0.1 mmol, 1.0 equiv.) in DMSO (2 mL) were used. After 18 hour, the product was isolated by flash chromatography (PE: EA= 60:1) as a pale yellow oil (27.1 mg, 64%, *Z:E* = 97:3).^{1}H NMR (600 MHz, $CDCl_3$) δ 8.02-8.00 (m, 1H), 7.83 (br, 1H), 7.46-7.45 (m, 1H), 7.31-7.29 (m, 2H), 7.22-7.20 (m, 2H), 6.27 (s, 1H), 1.50-1.45 (m, 7H), 1.29 (s, 9H), 1.21-1.17 (m, 3H), 1.15 (s, 3H). ^{13}C NMR (100 MHz, $CDCl_3$) δ 168.34, 151.57, 150.80, 145.77, 138.69, 137.63, 132.66, 131.20, 126.78, 126.69, 125.33, 124.30, 121.12, 39.20, 37.84, 34.55, 31.32, 28.33, 26.11, 23.09. HRMS (ESI+): calcd for $C_{26}H_{31}NSCl^+$ (M+H) 424.1860, found 424.1898.

Supplementary Discussion

Conformational analysis of Z/E isomers using density functional theory (DFT).

All calculations were performed with Gaussian 16 package. The density functional theory (DFT) method was employed using the B3LYP hybrid functional. The standard 6-31G(d,p) basis set was used for all atoms. Gibbs free energies were discussed unless otherwise specified. The 3D geometric structures were visualized with CYLview3.

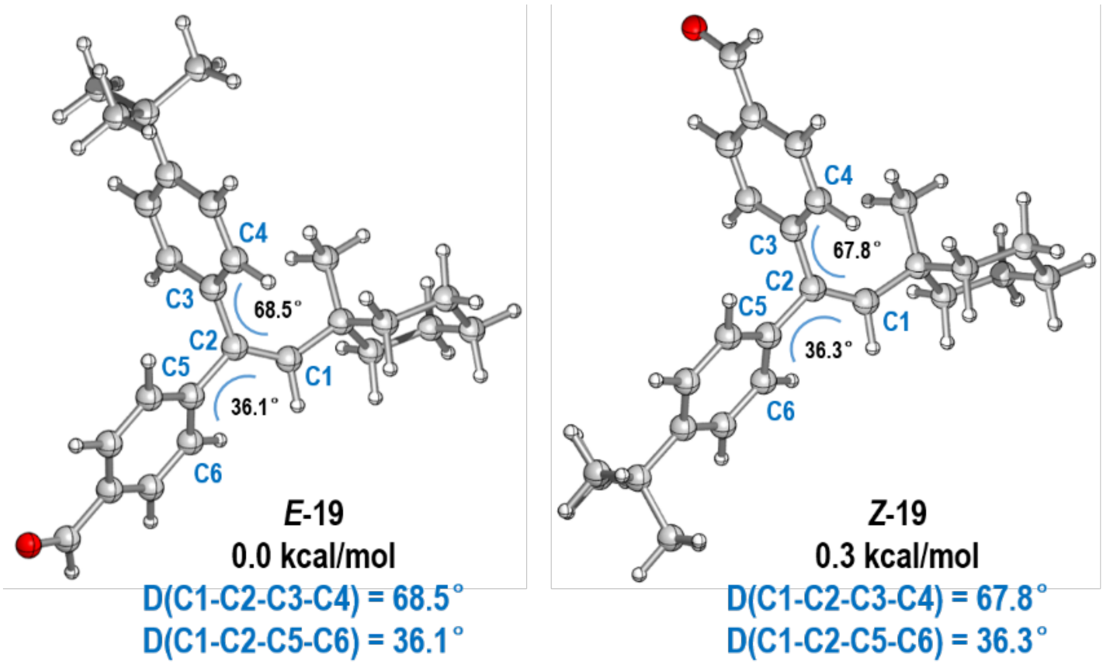

Supplementary Figure 3. Conformational analysis of *Z*-19 and *E*-19.

Evaluation of light sources.

To a flame-dried 8 mL reaction vials was charged with $NiCl_2$•DME (4.4 mg, 0.02 mmol, 20 mol%), dtbbpy (5.4 mg, 0.02 mmol, 20 mol%), $Ir[dF(CF_3)ppy]_2$ (dtbbpy)PF_6 (3.4 mg, 0.003 mmol, 3 mol%), 4-bromobenzaldehyde (37.0 mg, 0.2 mmol, 2.0 equiv.), and cesium salt (47.7 mg, 0.15 mmol, 1.5 equiv.) The vial was capped. After evacuated and backfilled nitrogen three times, DMSO (2 mL) was added via a syringe, followed by the addition of 1-(*tert*-butyl)-4-ethynylbenzene (18.1 uL, 0.1 mmol, 0.1equiv.). The reaction mixtures were then irradiated with a LED lamp or light bulb (at approximately 3 cm away from the light source) with cooling

from fan for 18 h. The reaction mixtures were analyzed by ^{1}H NMR with an internal standard.

Supplementary Table 1. Evaluation of light sources.[a]

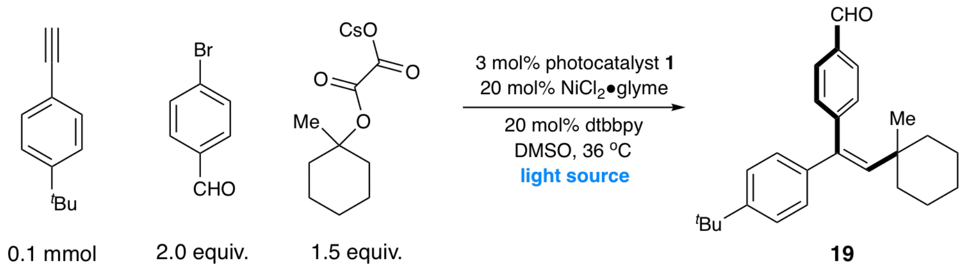

entry	light source	yield	*Z/E*
1	90 W blue LED	81%	96 : 4
2	10 W 460-465 nm LED	64%	95 : 5
3	10 W 425-430 nm LED	50%	94 : 6
4	10 W 385-390 nm LED	46%	94 : 6
5	10 W 375-377 nm LED	20%	88 : 12
6	10 W 365-367 nm LED	18%	80 : 20
7	10 W 512-520 nm LED	2%	50 : 50
8	3 W blue LED strip	6%	80 : 20
9	5 W CFL	8%	75 : 25
10	Sun light	trace	--

[a]Reaction conditions: photocatalyst (3 mol%), $NiCl_2$•glyme (20 mol%), dtbbpy (20 mol%), alkyne (0.1 mmol), oxalate (1.5 equiv.), aryl bromide (2.0 equiv.), DMSO [0.05 M], LED, 36 °C, 18 h. Yields determined by ^{1}H NMR with an internal standard, and the ratio of the two isomers was determined by ^{1}H NMR analysis of the crude reaction mixture.

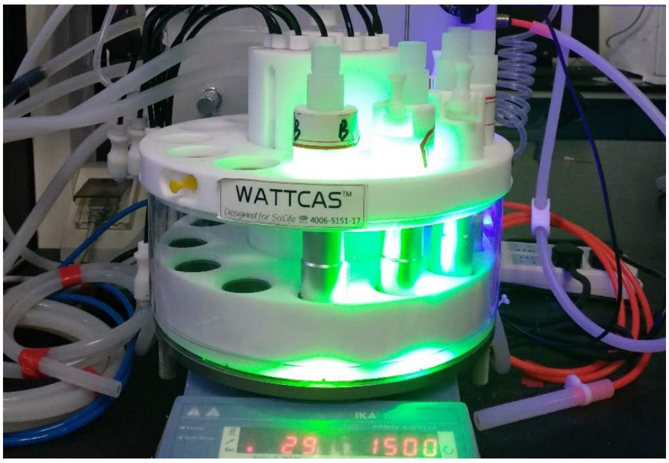

Supplementary Figure 4. Evaluation of light sources

Evaluation of loading of nickel catalyst.

To a flame-dried 8 mL reaction vials was charged with $NiCl_2$•DME (5-20 mol%), dtbbpy (5-20 mol%), Ir[dF(CF_3)ppy]$_2$ (dtbbpy)PF_6 (3.4 mg, 0.003 mmol, 3 mol%), 4-bromobenzaldehyde (37.0 mg, 0.2 mmol, 2.0 equiv.), and cesium salt (47.7 mg, 0.15 mmol, 1.5 equiv.) The vial was capped. After evacuated and backfilled nitrogen three times, DMSO (2 mL) was added via a syringe, followed by the addition of 1-(*tert*-butyl)-4-ethynylbenzene (18.1 uL, 0.1 mmol, 0.1equiv.). The reaction mixture was then irradiated with a LED lamp or light bulb (at approximately 3 cm away from the light source) with cooling from fan for 18 h. The reaction mixture was analyzed by ^{1}H NMR with an internal standard.

Supplementary Table 2. Evaluation of nickel loading.[a]

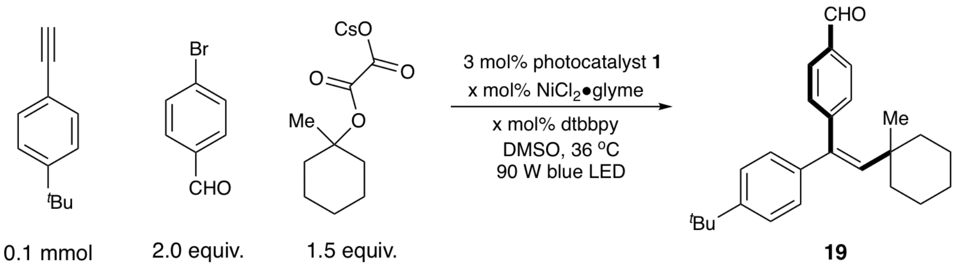

entry	loading of nickel	yield	*Z*/*E*
1	20 mol%	83%	96 : 4
2	15 mol%	73%	96 : 4
3	10 mol%	57%	97 : 3
4	5 mol%	41%	97 : 3

[a]Reaction conditions: photocatalyst (3 mol%), $NiCl_2$•glyme, dtbbpy, alkyne (0.1 mmol), oxalate (1.5 equiv.), aryl bromide (2.0 equiv.), DMSO [0.05 M], LED, 36 °C, 18 h. Yields determined by ^{1}H NMR with an internal standard, and the ratio of the two isomers was determined by ^{1}H NMR analysis of the crude reaction mixture.

Time course studies.

To nine flame-dried 8 mL reaction vials were charged with $NiCl_2$•DME (4.4 mg, 0.02 mmol, 20 mol%), dtbbpy (5.4 mg, 0.02 mmol, 20 mol%), $Ir[dF(CF_3)ppy]_2(dtbbpy)PF_6$ (3.4 mg, 0.003 mmol, 3 mol%), 4-bromobenzaldehyde (37.0 mg, 0.2 mmol, 2.0 equiv.), and cesium salt (47.7 mg, 0.15 mmol, 1.5 equiv.) The vials were capped. After evacuated and backfilled nitrogen three times, DMSO (2 mL) was added via a syringe, followed by the addition of 1-(tert-butyl)-4-ethynylbenzene (18.1 uL, 0.1 mmol, 0.1equiv.). The reaction mixtures were then irradiated with 90 W blue LED lamp (at approximately 3 cm away from the light source) with cooling from fan for 20 min, 40 min, 1 h, 2 h, 3h, 5 h, 8 h, 15 h and 18 h. The reaction mixtures were analyzed by ^{1}H NMR with an internal standard.

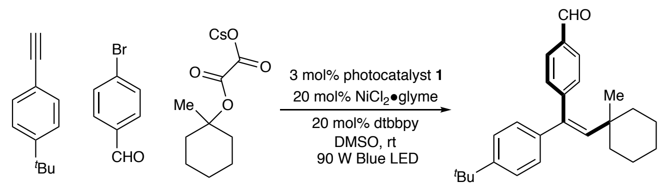

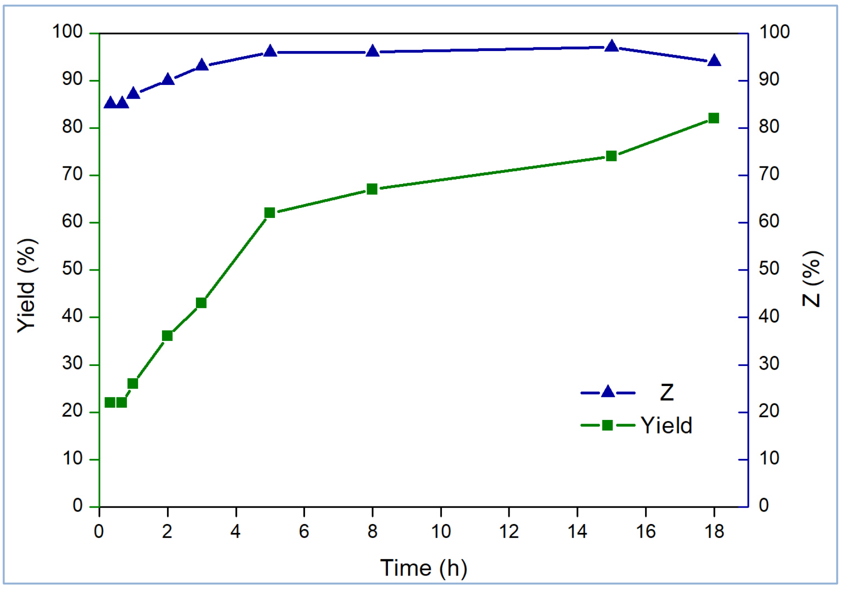

Supplementary Figure 5. Time course studies

Isomerization studies of *Z* and *E* isomers.

*Preparation of E-**41**.*

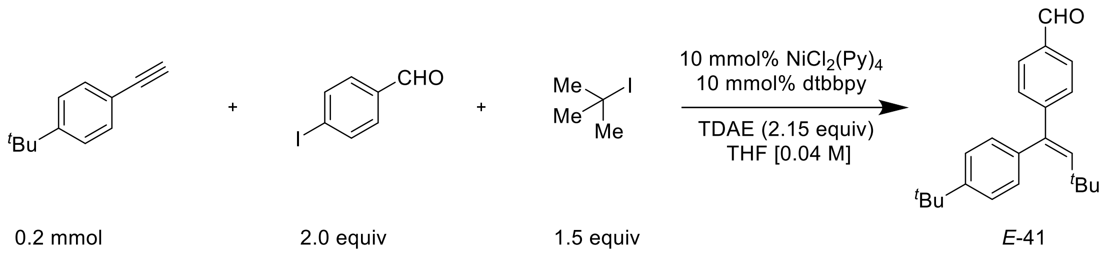

Supplementary Figure 6. Synthesis of compound *E*-41.

***E*-41** was prepared according to a modified procedure of Nevado's work.[1] To an oven dried Schlenk flask was added $NiCl_2(Py)_4$ (8.9 mg, 0.02 mmol, 10 mol%), dtbbpy (5.4 mg, 0.02 mmol, 10 mol%), and 4-iodobenzaldehyde (92.8 mg, 0.4 mmol, 2.0 equiv). The reaction vessel was evacuated and filled back with nitrogen three times. THF (0.5 mL), 1-(tert-butyl)-4-ethynylbenzene (36.2 uL, 0.2 mmol, 1.0 equiv), and 2-iodo-2-methylpropane (35.8 uL, 0.3 mmol, 1.5 equiv) were sequentially added and stirred one minute at 25 ºC. TDAE (0.1 mL, 0.43 mmol, 2.15 equiv) was added dropwise and the reaction mixture was stirred for additional 15 h at 25 ºC under N_2. The mixture was diluted with Et_2O (5 mL) and transferred into a separatory funnel containing aqueous HCl (1M, 5 mL). The phases were separated and the aqueous phase extracted with Et_2O twice. The organic extracts were dried over Na_2SO_4 and concentrated, the product was purified by flash chromatography (PE: EA= 60:1) as a pale yellow oil (25.6 mg, 40%).

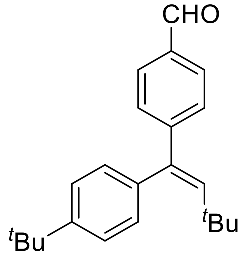

(*E*)-4-(1-(4-(*tert*-Butyl)phenyl)-3,3-dimethylbut-1-en-1-yl)benzaldehyde (*E*-41): ^{1}H NMR (600 MHz, $CDCl_3$) δ 9.95 (s, 1H), 7.73 (d, *J* = 8.4 Hz, 2H), 7.35 (m, 4H), 7.08 (d, *J* = 8.3 Hz, 2H), 6.21 (s, 1H), 1.35 (s, 9H), 0.97 (s, 9H). ^{13}C NMR (150 MHz, $CDCl_3$) δ 191.95, 150.46, 150.21, 143.09, 138.55, 136.53, 134.56, 129.86, 129.58, 127.41, 124.85, 34.60, 34.31, 31.47, 31.19. HRMS (ESI+): calcd for $C_{23}H_{29}O^+$ (M+H) 321.2213, found 321.2212.

Isomerization of E and Z-isomers

To a flame-dried 8 mL reaction vial was charged with (*E*)-**41** (8.0 mg, 0.025 mmol, 1.0 equiv.), Ir[dF(CF_3)ppy]$_2$(dtbbpy)PF_6 (0.8 mg, 0.00075 mmol, 3 mol%), and TEMPO (4.0 mg, 0.025 mmol, 1.0 equiv.). The vial was capped. After evacuated and backfilled nitrogen three times, DMSO [0.05 M] was added via a syringe. The reaction mixture was then irradiated with a 90 W blue LED lamp (at approximately 3 cm away from the light source) with cooling from a fan for 18h. The reaction mixture was analyzed by ^{1}H NMR with an internal standard.

Supplementary Table 3. Isomerizations of *E* and *Z*-isomers

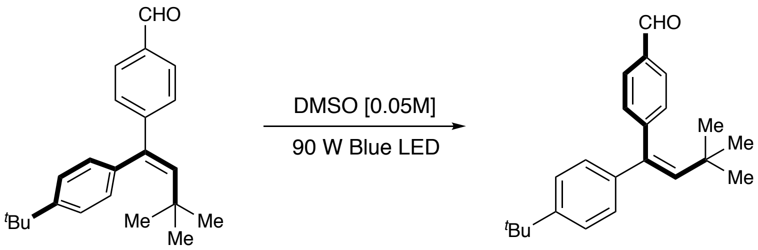

(*E*)/(*Z*)-41 **(*Z*)-41**

entry	substrate	conditions	yield	*Z*/*E*
1	(*E*)-**41**	visible light, w/ 3 mol% **1**	>99%	97:3
2	(*E*)-**41**	visible light	>99%	97:3
3	(*E*)-**41**	dark	>99%	0:100
4	(*Z*)-**41**	visible light	>99%	96:4
5	(*E*)-**41**	visible light, w/ TEMPO	>99%	67:33
6	(*E*)-**41**	visible light, w/ BHT	>99%	97:3
7	(*E*)-**41**	visible light, w/ air	>99%	97:3

Reaction conditions: (*E*)/(Z)-**41** (0.025 mmol), Ir-**1** (3 mol%), additive (1.0 equiv), DMSO [0.05 M], 90W blue LED, 18h.

UV-Vis absorption spectra of *Z* and *E* isomers as well as substrates.

Solutions of *Z* and *E*-isomers (1.0×10^{-4} M), alkyne (1.0×10^{-4} M), aryl bromide (1.0×10^{-4} M) in 2 mL DCM, were recorded in 1 cm path quartz cuvettes using a Shimadzu UV-2550 spectrometer.

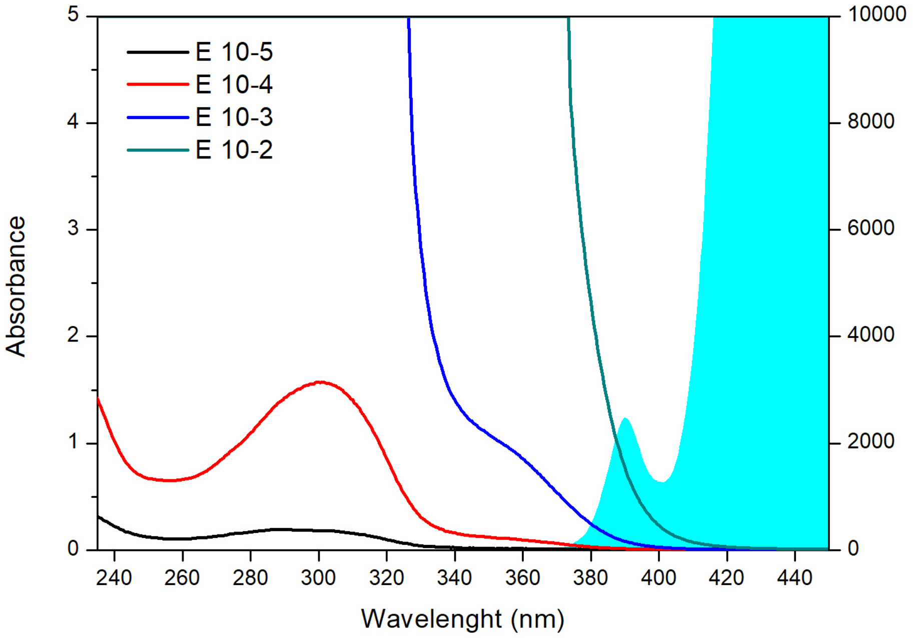

Supplementary Figure 7. UV-vis absorption of *E*-41.

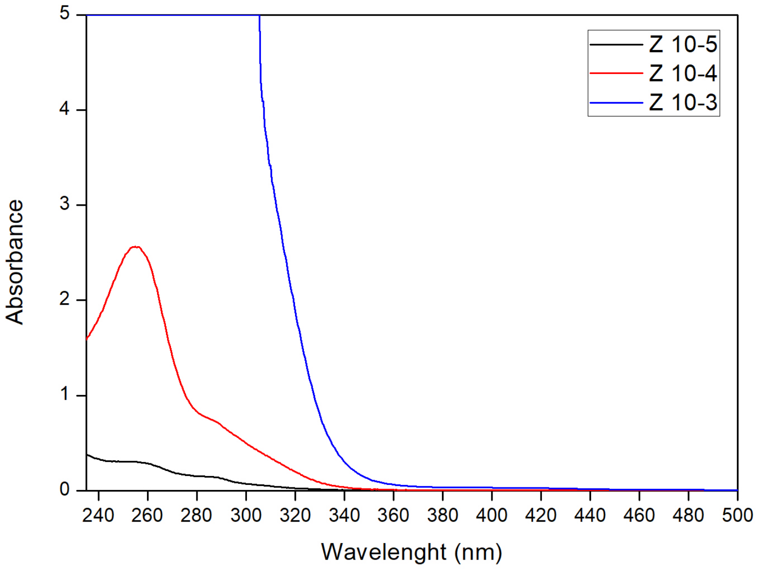

Supplementary Figure 8. UV-vis absorption of *Z*-19.

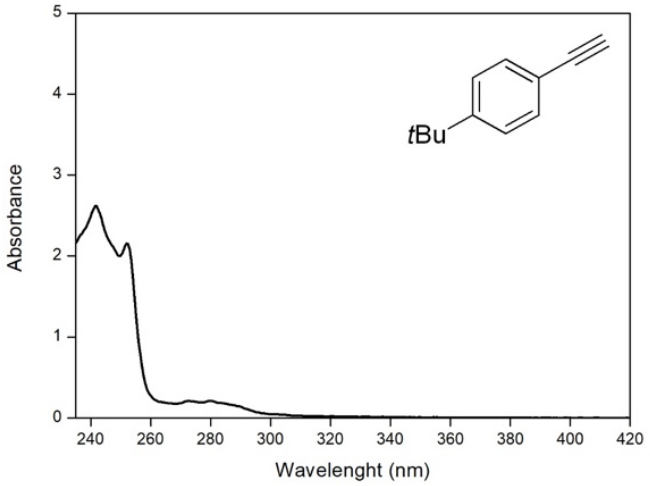

Supplementary Figure 9. UV-vis absorption of alkyne 16.

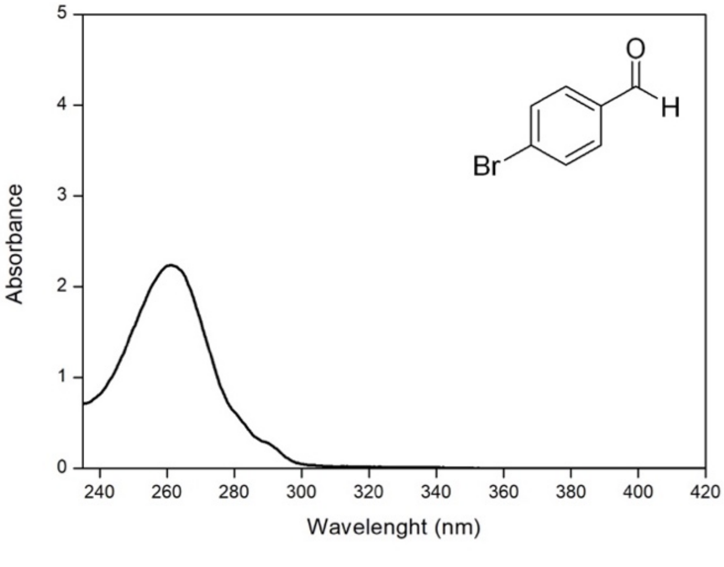

Supplementary Figure 10. UV-vis absorption of bromide 17.

Control experiments between two components

To a flame-dried 8 mL reaction vial was charged with $NiCl_2$•DME (0.02 mmol, 20 mol%), dtbbpy (0.02 mmol, 20 mol%), Ir[dF(CF_3)ppy]$_2$(dtbbpy)PF_6 (0.003 mmol, 3 mol%), halide or alkyne (0.1 mmol, 1.0 equiv) and cesium salt (0.15 mmol, 1.5 equiv.), the vial was capped. After evacuated and backfilled nitrogen three times,

DMSO [0.05 M] was added via a syringe. The reaction mixture was then irradiated with a 90 W blue LED lamp (at approximately 3 cm away from the light source) with cooling from a fan for 18h. The reaction was quenched with H_2O, extracted with ethyl acetate. The combined organic layers were dried with Mg_2SO_4, filtered, and concentrated in vacuo. The crude material was purified by flash chromatography to afford the product.

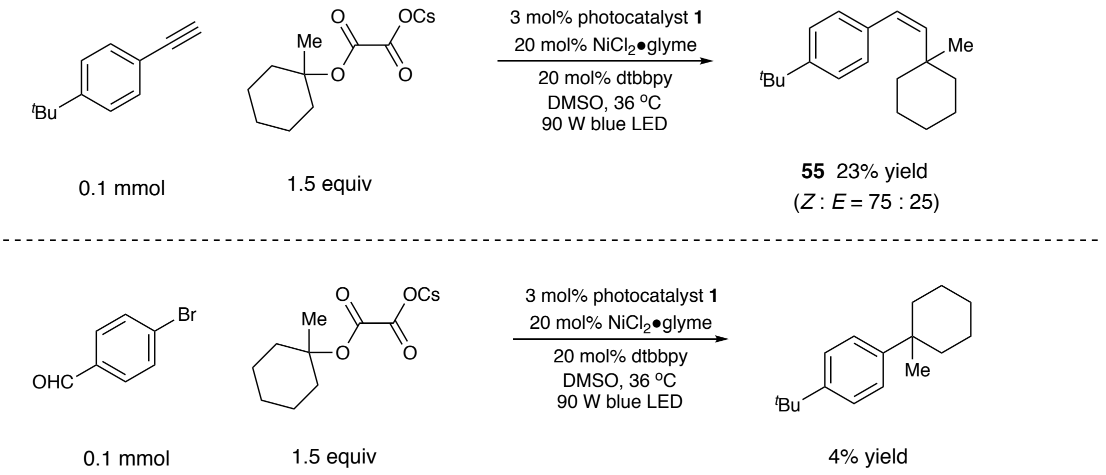

Supplementary Figure 11. Two-component control reactions.

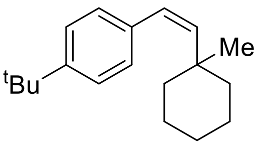

(Z)-1-(tert-Butyl)-4-(2-(1-methylcyclohexyl)vinyl)benzene (55): ^{1}H NMR (400 MHz, $CDCl_3$) δ 7.30 (d, J = 8.2 Hz, 2H), 7.12 (d, J = 8.2 Hz, 2H), 6.44 (d, J = 12.8 Hz, 1H), 5.50 (d, J = 12.8 Hz, 1H), 1.55-1.46 (m, 7H), 1.31 (s, 9H), 1.18-1.16 (m, 1H), 1.11-1.08 (m, 2H), 1.03 (s, 3H). m/z: 256.2191, GC-MS found 256.2.

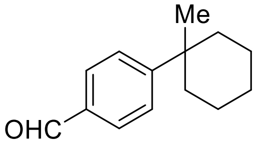

4-(1-Methylcyclohexyl)benzaldehyde (S21) : ^{1}H NMR (400 MHz, $CDCl_3$) δ 9.99 (s, 1H), 7.84 (d, J = 8.4 Hz, 2H), 7.55 (d, J = 8.4 Hz, 2H), 2.07 -2.02 (m, 3H), 1.65-1.63 (m, 2H), 1.48-1.39 (m, 5H), 1.21 (s, 3H). m/z: 202.1358, GC-MS found 202.1.

Stoichiometric reactions of aryl-Ni(II) complex.

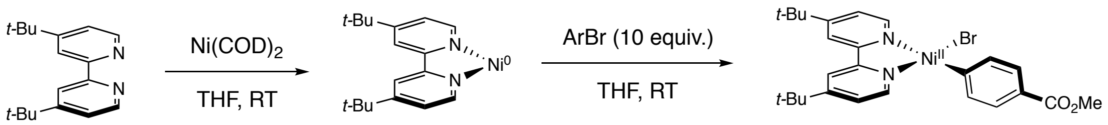

Supplementary Figure 12. Synthesis of Ni(II) complex 56

Preparation of Ni(II) complex ***56*** [2]

In a nitrogen filled glove box, a 50 mL round bottom flask containing a stirring bar was charged with $Ni(COD)_2$ (138 mg, 0.5 mmol, 1.0 equiv), 4,4'-di-tert-butyl-2,2'-bipyridine (134 mg, 0.5 mmol, 1.0 equiv) and dry THF (5 mL) giving a dark purple mixture which was stirred for 12 hours at 25 °C. Methyl 4-bromobenzoate (1.1 g, 5 mmol, 10.0 equiv) was added and stirred for additional 4 h. Dry pentane (30 mL) was added to the deep red colored mixture and filtered. The resulting precipitate was washed with pentane (3 x 10 mL) and dried under vacum to afford Ni(II) complex **56** as a brown solid (176 mg, 65% yield). The product was used without further purification. The complex was stored in a nitrogen filled glove box at -35 °C. ^{1}H NMR (600 MHz, CD_2Cl_2) δ 9.25 (s, 1H), 7.88 (d, J = 16.7 Hz, 2H), 7.78 (br, 2H), 7.59 (m, 3H), 7.12 (m, 2H), 3.88 (s, 3H), 1.41 (m, 18H).

Stoichiometric reactions of Ni(II) complex ***56****.*

An oven-dried screw-cap vial containing a stirring bar was charged with $Ir[dF(CF_3)ppy]_2(dtbbpy)PF_6$ (28.0 mg, 0.025 mmol, 1.0 equiv) and cesium 2-((1-methylcyclohexyl)oxy)-2-oxoacetate (8.0 mg, 0.025 mmol, 1.0 equiv). Then the vial was introduced into a nitrogen-filled glove box and charged with Ni(II) complex **56** (13.6 mg, 0.025 mmol, 1 equiv), DMSO (1 mL). The tube was sealed with a

Teflon-lined screw cap and taken out from the glovebox. 1-(*tert*-butyl)-4-ethynylbenzene (4.6 uL, 0.025 mmol, 1.0 equiv) was added by syringe. Once added, the reaction mixture was then irradiated with a 90 W blue LED lamp (at approximately 3 cm away from the light source) with cooling from a fan for 18h. The reaction mixtures were analyzed by ^{1}H NMR with an internal standard.

Supplementary Table 4. Reactions of Ni(II) complex 56

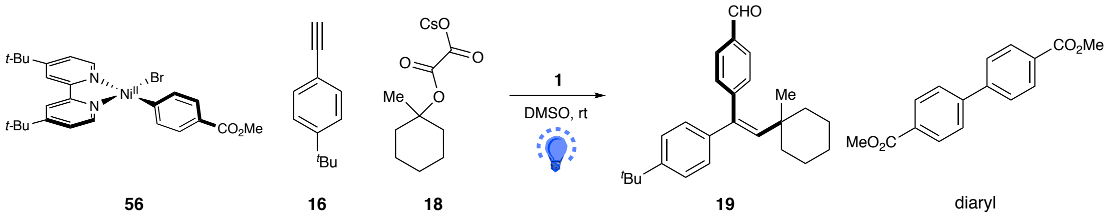

entry	conditions	pdt 19	diaryl
1	**57** (0.025 mmol), **16** (1.0 equiv.), **18** (1.0 equiv), **1** (1.0 equiv.)	0%	28%
2	**57** (0.025 mmol), **16** (3.0 equiv.), **18** (3.0 equiv), **1** (0.4 equiv.)	0%	28%
3	**57** (0.025 mmol), **16** (0.4 equiv.), **18** (0.4 equiv), **1** (0.4 equiv.)	0%	22%

Reaction conditions: 56 (0.025 mmol), Ir-**1** (1.0 equiv.), **16** (1.0 equiv.), **18** (1.0 equiv.), DMSO [0.025 M], 90W blue LED, 18h.

Other successful and unsuccessful aryl halides.

To a flame-dried 8 mL reaction vial was charged with $NiCl_2$•DME (0.02 mmol, 20 mol%), dtbbpy (0.02 mmol, 20 mol%), Ir[dF(CF_3)ppy]$_2$(dtbbpy)PF_6 (0.003 mmol, 3 mol%), aryl halide (0.2 mmol, 2.0 equiv) and cesium salt (0.15 mmol, 1.5 equiv.), the vial was capped. After evacuated and backfilled nitrogen three times, DMSO [0.05 M] was added via a syringe, aryl bromide (0.2 mmol, 2.0 equiv., if liquid), followed by the addition of terminal alkyne (0.1 mmol, 1.0 equiv.). The reaction mixture was then irradiated with a 90 W blue LED lamp (at approximately 3 cm away from the light source) with cooling from a fan for 18h. The reaction was quenched with H_2O, extracted with ethyl acetate. The combined organic layers were dried with Mg_2SO_4, filtered, and concentrated in vacuo. The crude material was purified by flash chromatography to afford the product.

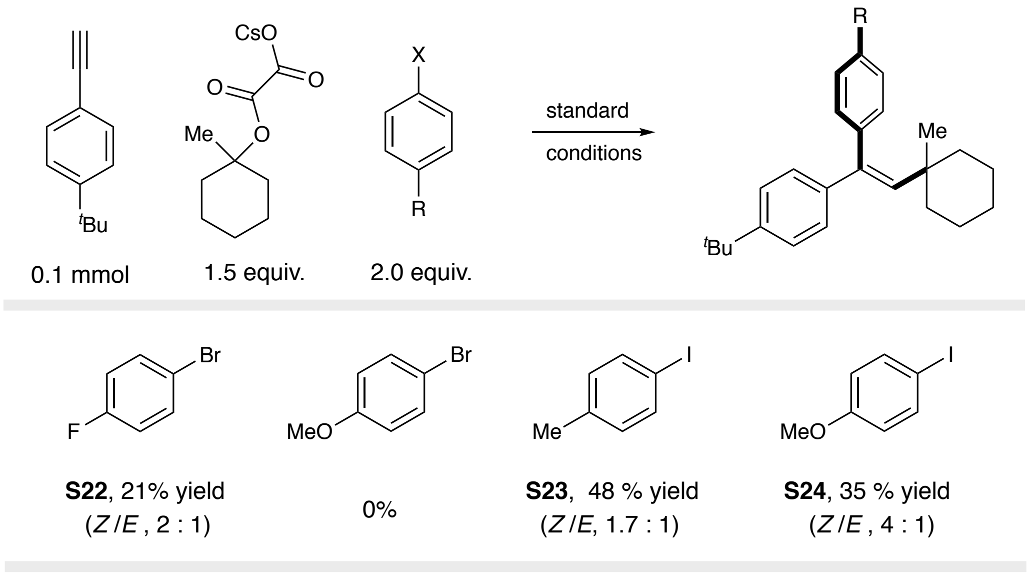

Supplementary Figure 13. Other aryl halides.

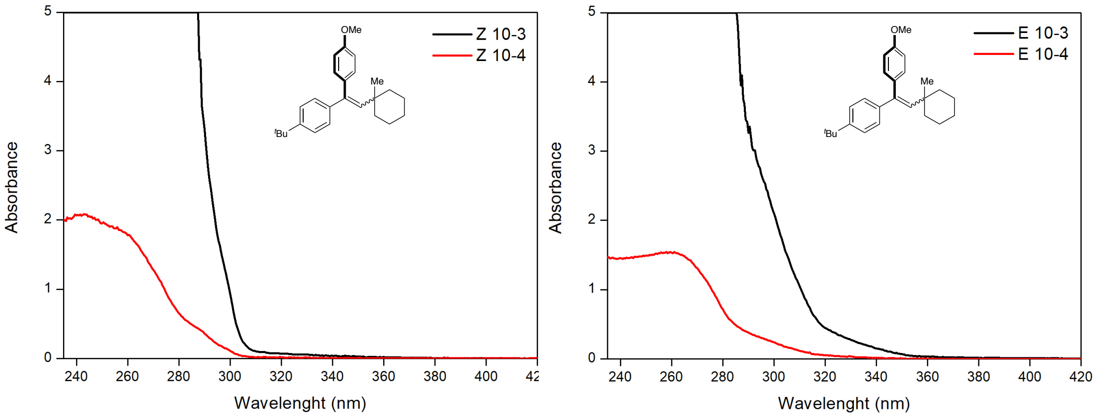

Supplementary Figure 14. UV-vis absorption of S24.

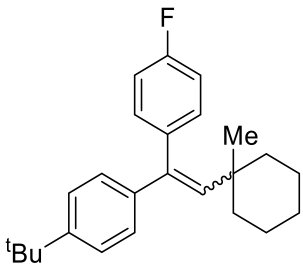

1-(tert-butyl)-4-(1-(4-fluorophenyl)-2-(1-methylcyclohexyl)vinyl)benzene (S22):

^{1}H NMR (400 MHz, $CDCl_3$) δ 7.32 (d, *J* = 8.2 Hz, 1H), 7.26 (d, *J* = 7.3 Hz, 1H), 7.17-7.14 (m, 2H), 7.12-7.07 (m, 2H), 7.05-7.00 (m, 1H), 6.93-6.88 (m, 1H), 6.04 (s,

0.54H), 5.92 (s, 0.46H), 1.46-1.38 (m, 7H), 1.33-1.29 (s, 9H), 1.21-1.18 (m, 1H), 1.08-1.02 (m, 2H), 0.98-0.97 (s, 3H). ^{19}F NMR (375 MHz, $CDCl_3$) δ -115.90 – -115.97 (m), δ -116.92 – -116.99 (m). ^{13}C NMR (100 MHz, $CDCl_3$) δ 161.88 (d, *J* = 245.3 Hz) [161.81 (d, *J* = 245.3 Hz)], 149.81 (149.72), 141.22 (139.46), 140.92 (d, *J* = 3.2 Hz)[136.87 (d, *J* = 3.5 Hz)], 138.96 (138.90), 138.88 (137.61), 131.49 (d, *J* = 7.8 Hz) [128.40 (d, *J* = 7.8 Hz)], 129.41 (126.32), 124.97 (124.67), 114.79 (d, *J* = 9.7 Hz) [114.58 (d, *J* = 9.8 Hz)], 39.47(39.51), 37.15, 34.53(34.40), 31.45(31.32), 29.73(29.46), 26.18(26.14), 23.03(23.00). HRMS (ESI+): calcd for $C_{25}H_{32}F^+$ (M+H) 351.2488, found 351.2496.

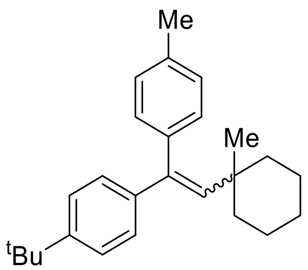

1-(tert-Butyl)-4-(2-(1-methylcyclohexyl)-1-(p-tolyl)vinyl)benzene (S23)： ^{1}H NMR (400 MHz, $CDCl_3$) δ 7.31 (d, *J* = 8.2 Hz, 1H), 7.24 (br, 1H), 7.15-7.02 (m, 6H), 6.01 (s, 0.69H), 5.96 (s, 0.31H), 2.33 (s, 3H), 1.48-1.38 (m, 8H), 1.33 (s, 3H), 1.28 (s, 6H), 1.06-1.04 (m, 2H), 0.97 (s, 3H). ^{13}C NMR (150 MHz, $CDCl_3$) δ 149.39 (149.50), 141.66 (142.02), 139.90 (140.16), 138.23(138.15), 138.01(137.94), 136.18 (136.11), 129.81 (129.44), 128.45 (128.63), 126.32 (126.76), 124.87 (124.51), 39.54, 37.10 (37.08), 34.37 (34.50), 31.33 (31.46), 29.72 (29.57), 26.21 (26.23), =23.02 , 21.28 (21.00). HRMS (ESI+): calcd for $C_{26}H_{35}^+$ (M+H) 347.2733, found 347.2727.

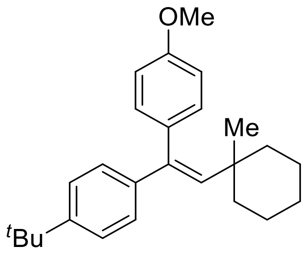

(Z)-1-(tert-Butyl)-4-(1-(4-methoxyphenyl)-2-(1-methylcyclohexyl)vinyl)benzene (Z-S24): ^{1}H NMR (600 MHz, $CDCl_3$) δ 7.25 (d, J = 8.4 Hz, 2H), 7.14 (d, J = 8.4 Hz, 2H), 7.10 (d, J = 8.5 Hz, 2H), 6.87 (d, J = 8.5 Hz, 2H), 6.01 (s, 1H), 3.83 (s, 3H), 1.47-1.36 (m, 7H), 1.28 (s, 9H), 1.23-1.18 (m, 1H), 1.07-1.02 (m, 2H), 0.98 (s, 3H). ^{13}C NMR (150 MHz, $CDCl_3$) δ 158.36, 149.41, 141.74, 139.51, 138.43, 133.25, 130.97, 126.32, 124.84, 113.13, 55.15, 39.50, 37.11, 34.35, 31.31, 29.59, 26.18, 23.00 .

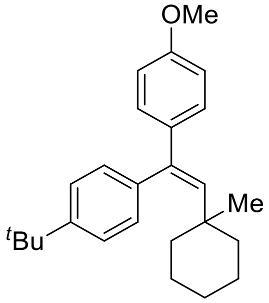

(*E*)-1-(tert-Butyl)-4-(1-(4-methoxyphenyl)-2-(1-methylcyclohexyl)vinyl)benzene (*E*-S24): ^{1}H NMR (600 MHz, $CDCl_3$) δ 7.31 (d, J = 8.2 Hz, 2H), 7.13 (d, J = 8.8 Hz, 2H), 7.08 (d, J = 8.2 Hz, 2H), 6.77 (d, J = 8.8 Hz, 2H), 5.91 (s, 1H), 3.77 (s, 3H), 1.46-1.37 (m, 7H), 1.33 (s, 9H), 1.22-1.20 (m, 1H), 1.05-1.01 (m, 2H), 0.97 (s, 3H). ^{13}C NMR (150 MHz, $CDCl_3$) δ 158.40, 149.52, 139.72, 138.00, 137.55, 137.34, 129.44, 127.90, 124.51, 113.29, 55.27, 39.55, 37.02, 34.49, 31.45, 29.58, 26.22, 23.02.

Reaction with secondary alkyl oxalate.

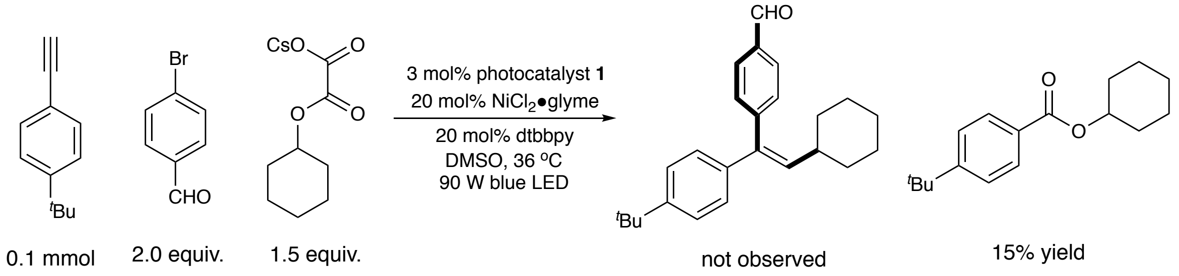

Supplementary Figure 15. Reaction of secondary alkyl oxalates.

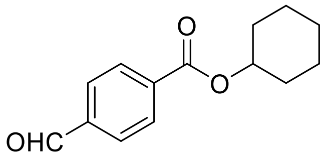

cyclohexyl 4-formylbenzoate (S25): ^{1}H NMR (400 MHz, $CDCl_3$) δ 10.11 (s, 1H), 8.20 (d, J = 8.2 Hz, 2H), 7.95 (d, J = 8.2 Hz, 2H), 5.10 - 5.03 (m, 1H), 2.01-1.94 (m, 1H), 1.85-1.76 (m, 2H), 1.49-1.42 (m, 4H), 1.32-1.28 (m, 3H).

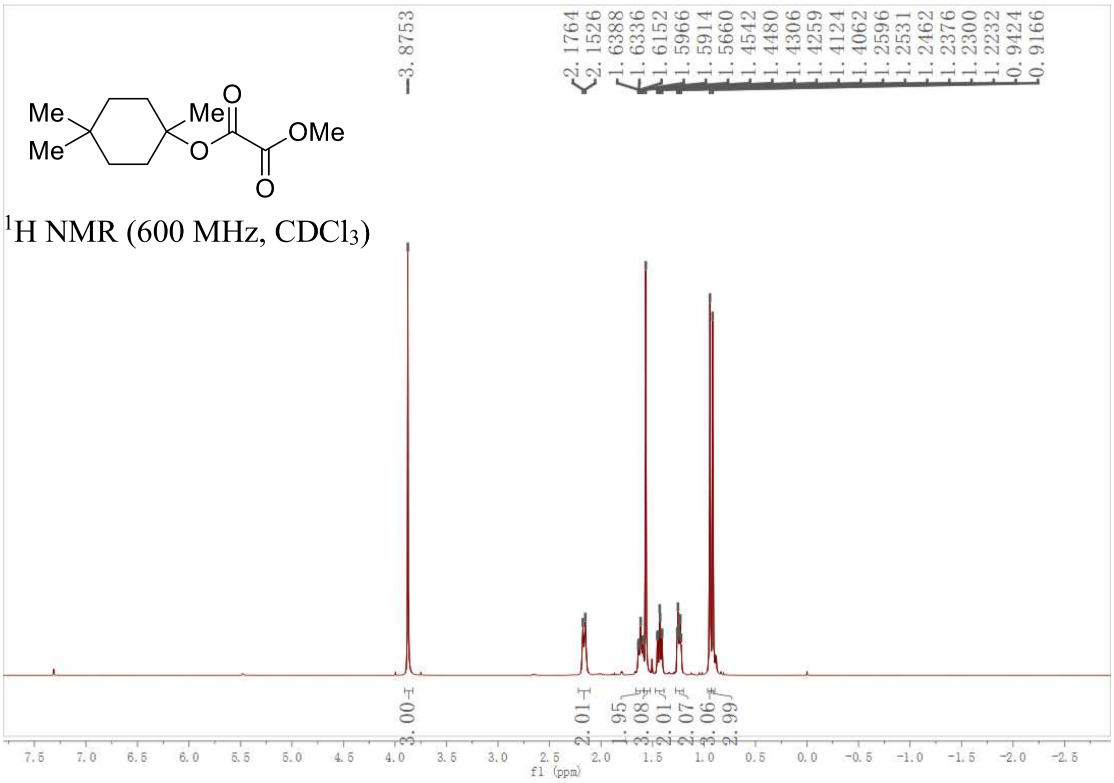

Supplementary Figure 16: ^{1}H NMR Spectra of Methyl (1,4,4-trimethyl cyclo hexyl) oxalate (S1)

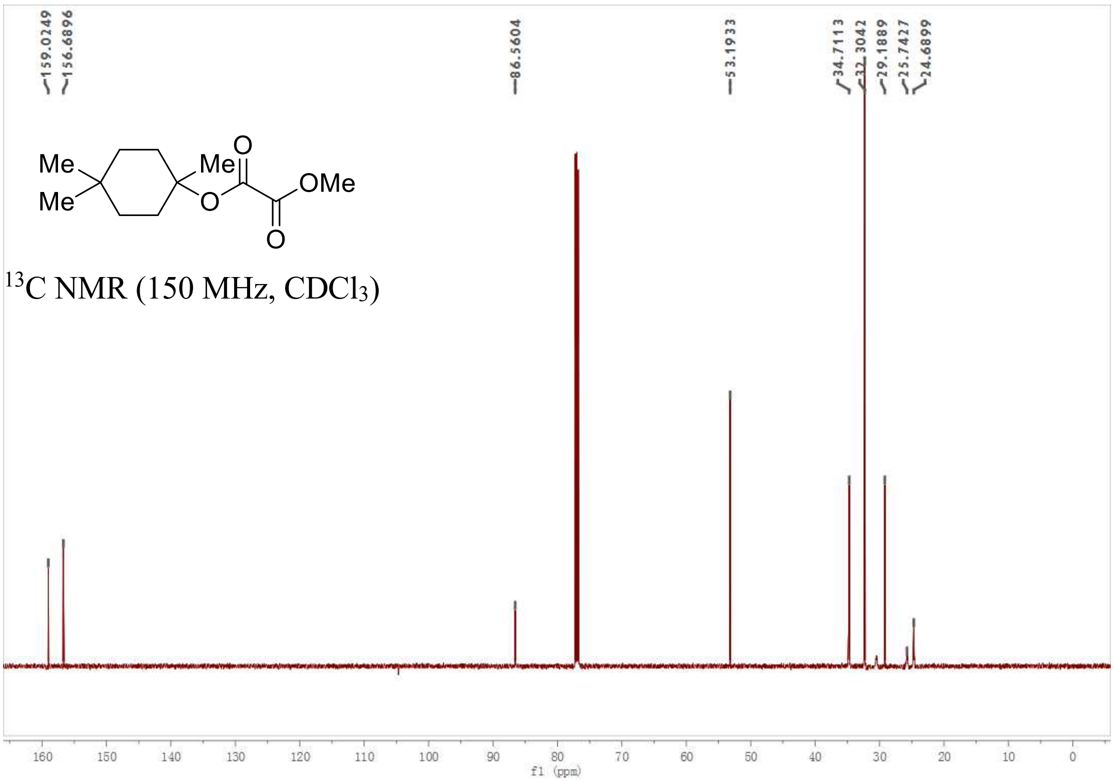

Supplementary Figure 17: ^{13}C NMR Spectra of Methyl (1,4,4-trimethylcyclohexyl) oxalate (S1)

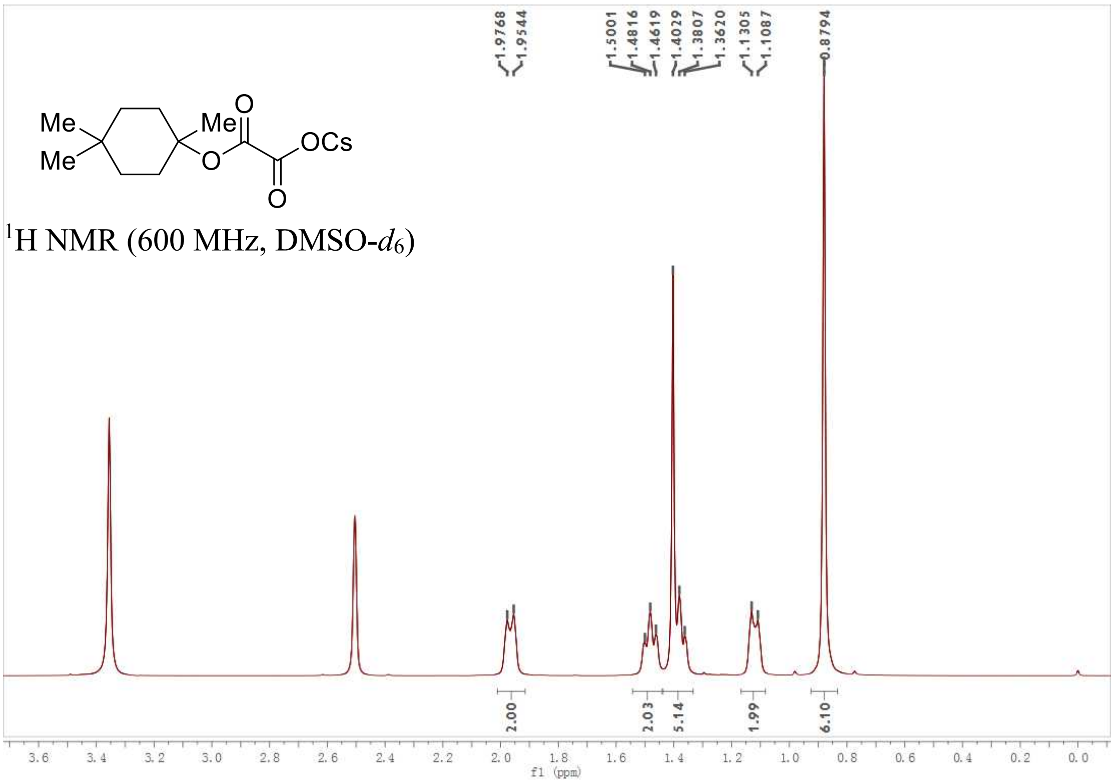

Supplementary Figure 18: ^{1}H NMR Spectra of Cesium 2-oxo-2-((1,4,4-trimethylcyclohexyl)oxy) acetate (S2)

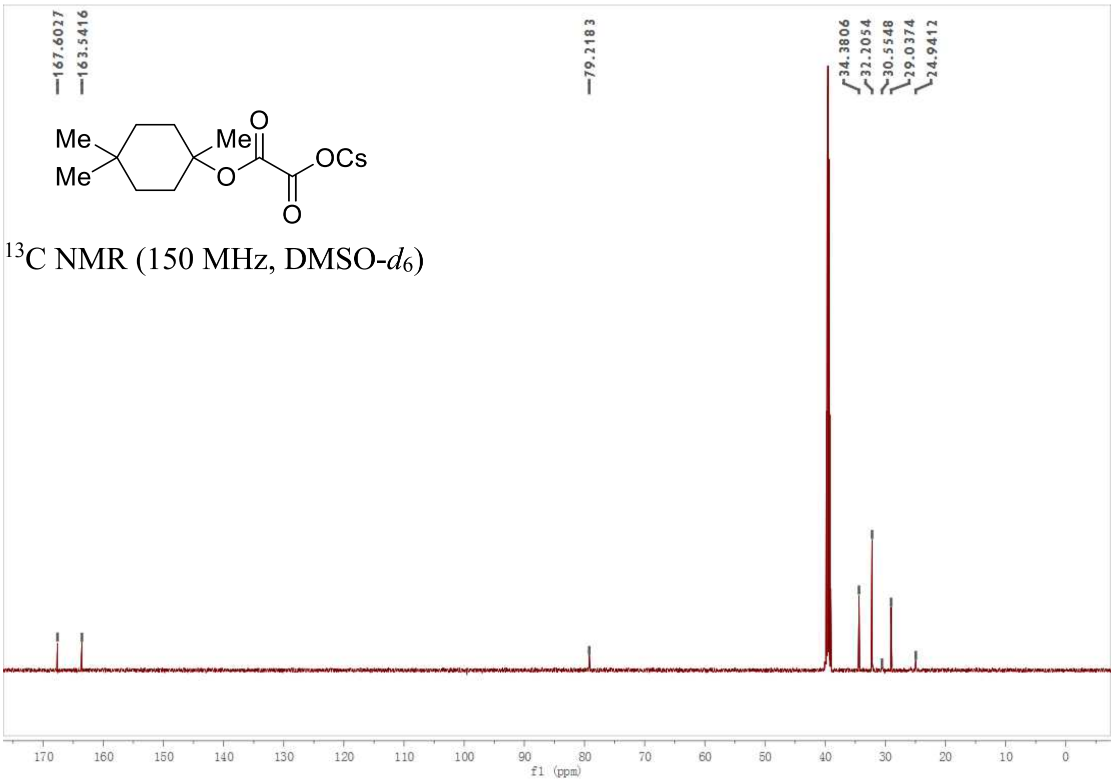

Supplementary Figure 19: ^{13}C NMR Spectra of Cesium 2-oxo-2-((1,4,4-trimethylcyclohexyl)oxy) acetate (S2)

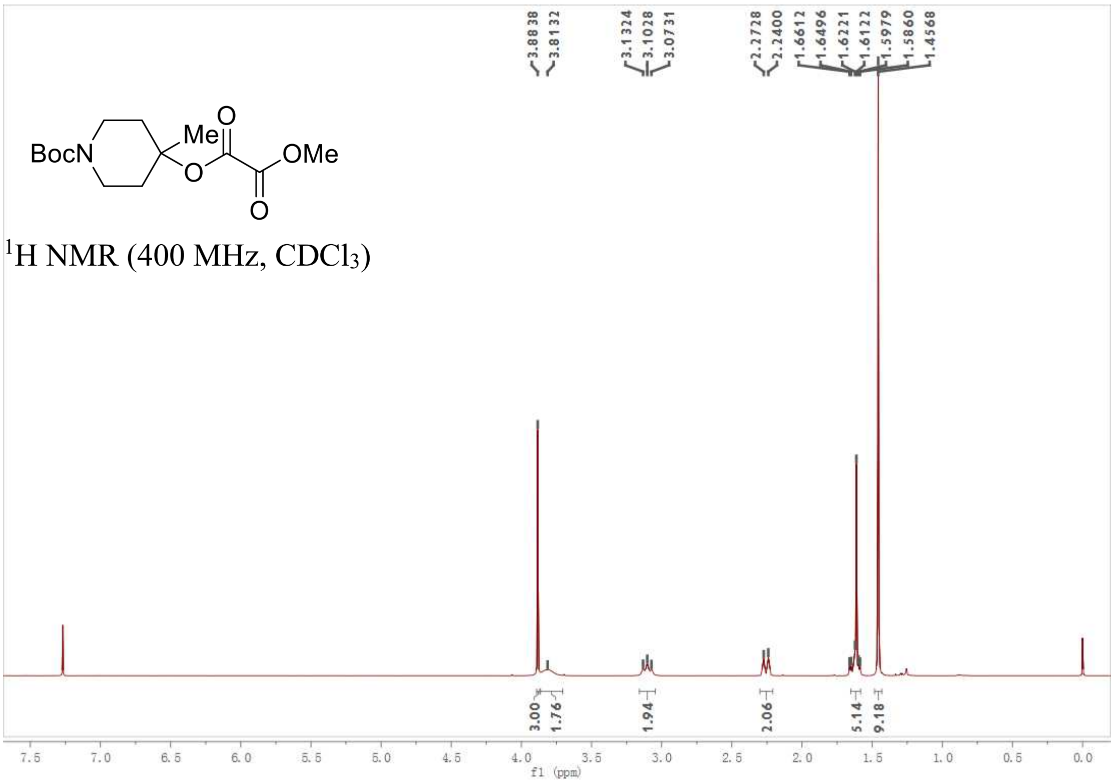

Supplementary Figure 20: ^{1}H NMR Spectra of 1-(tert-Butoxycarbonyl)-4-methylpiperidin-4-yl methyl oxalate (S3)

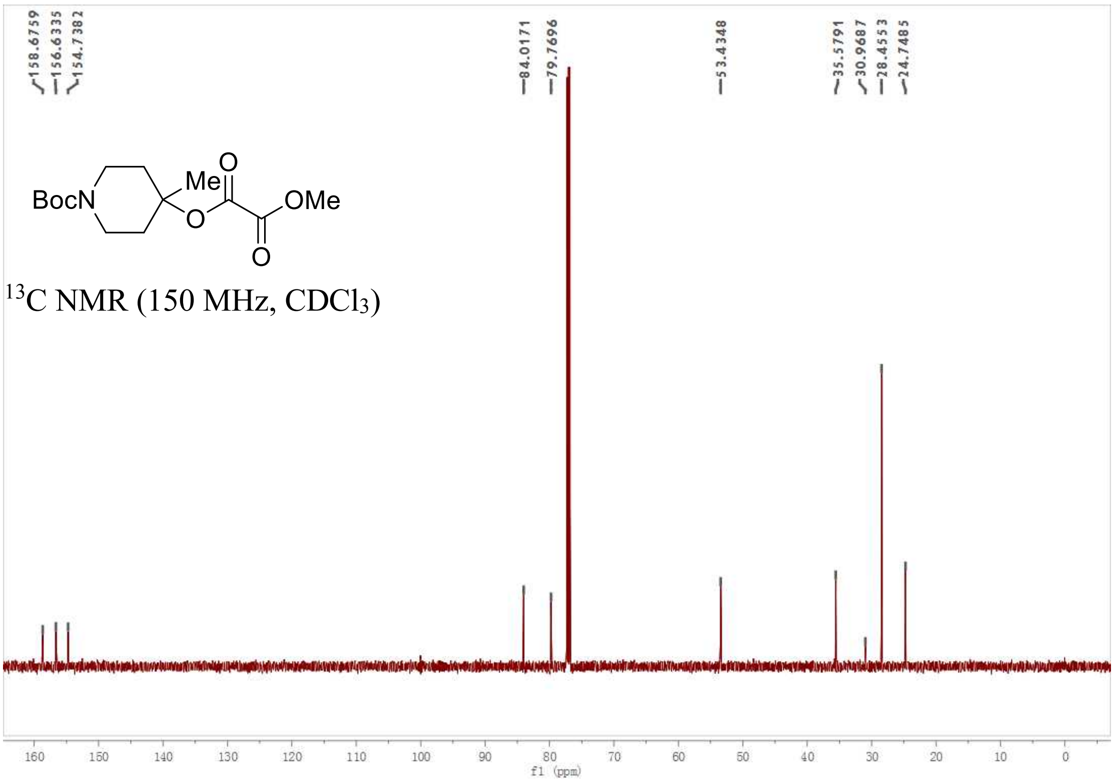

Supplementary Figure 21: ^{13}C NMR Spectra of 1-(tert-Butoxycarbonyl)-4-methylpiperidin-4-yl methyl oxalate (S3)

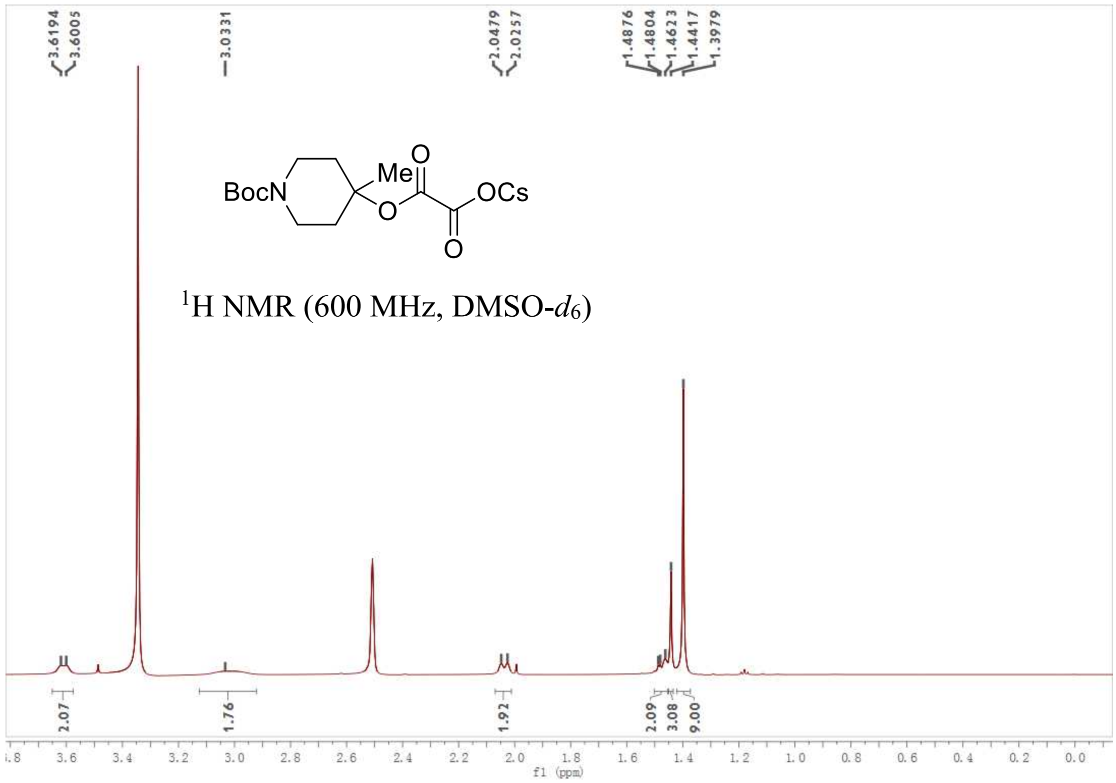

Supplementary Figure 22: ^{1}H NMR Spectra of Cesium 2-((1-(tert-butoxycarbonyl)-4-methylpiperidin-4-yl)oxy)-2-oxoacetate (S4)

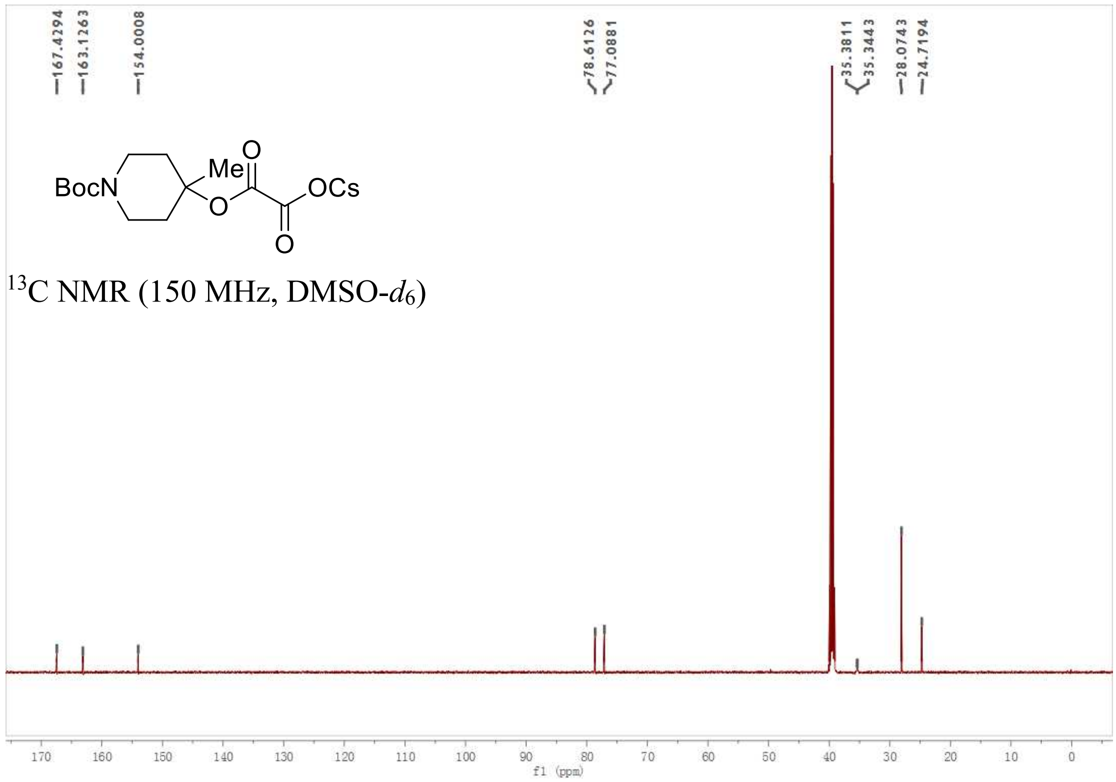

Supplementary Figure 23: ^{13}C NMR Spectra of Cesium 2-((1-(tert-butoxycarbonyl)-4-methylpiperidin-4-yl)oxy)-2-oxoacetate (S4)

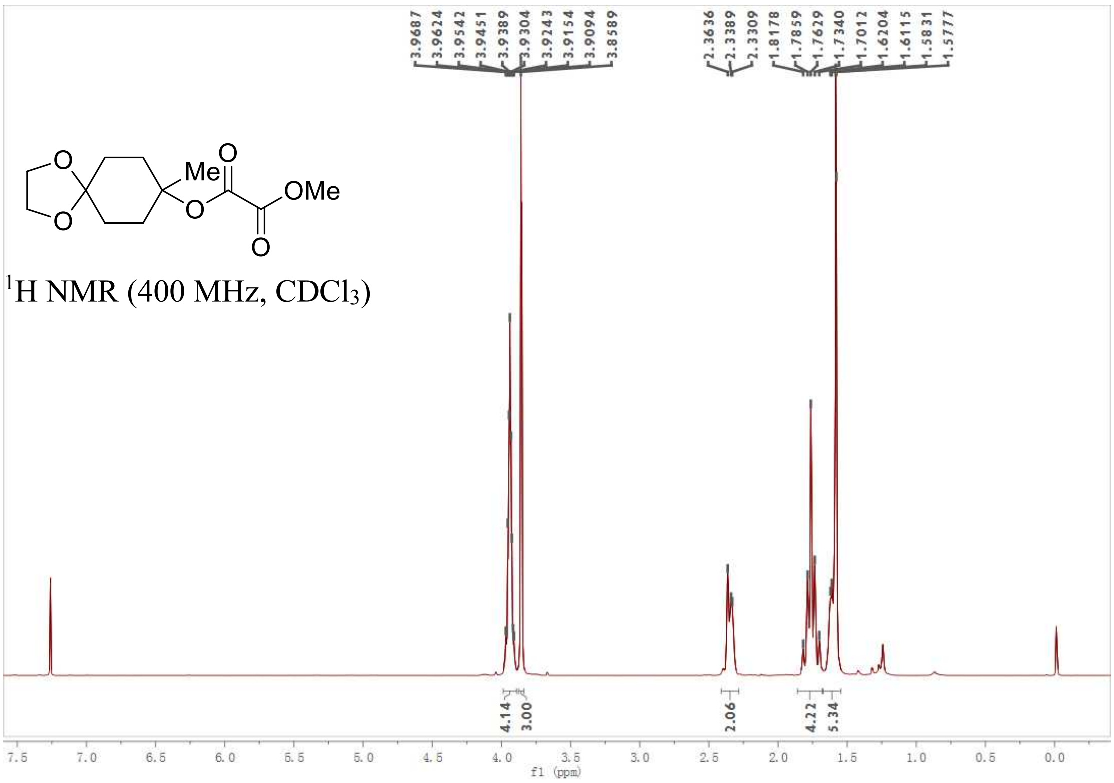

Supplementary Figure 24: ^{1}H NMR Spectra of Methyl (8-methyl-1,4-dioxaspiro[4.5]decan-8-yl) oxalate (S5)

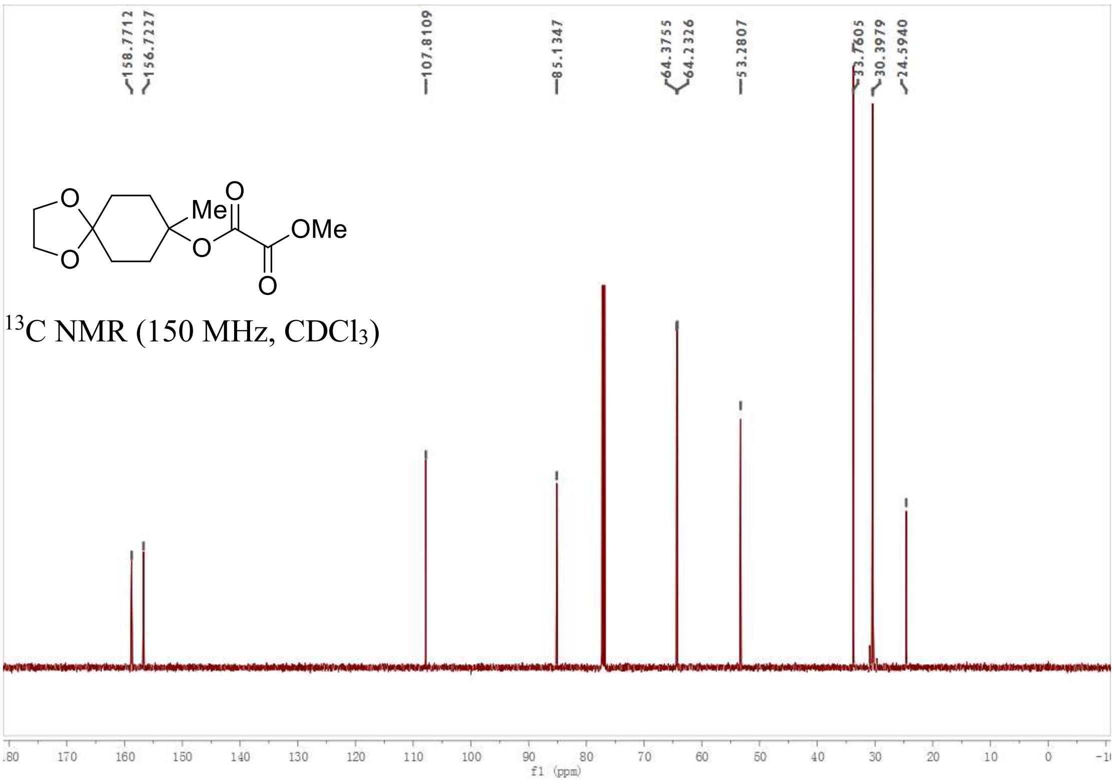

Supplementary Figure 25: ^{13}C NMR Spectra of Methyl (8-methyl-1,4-dioxaspiro[4.5]decan-8-yl) oxalate (S5)

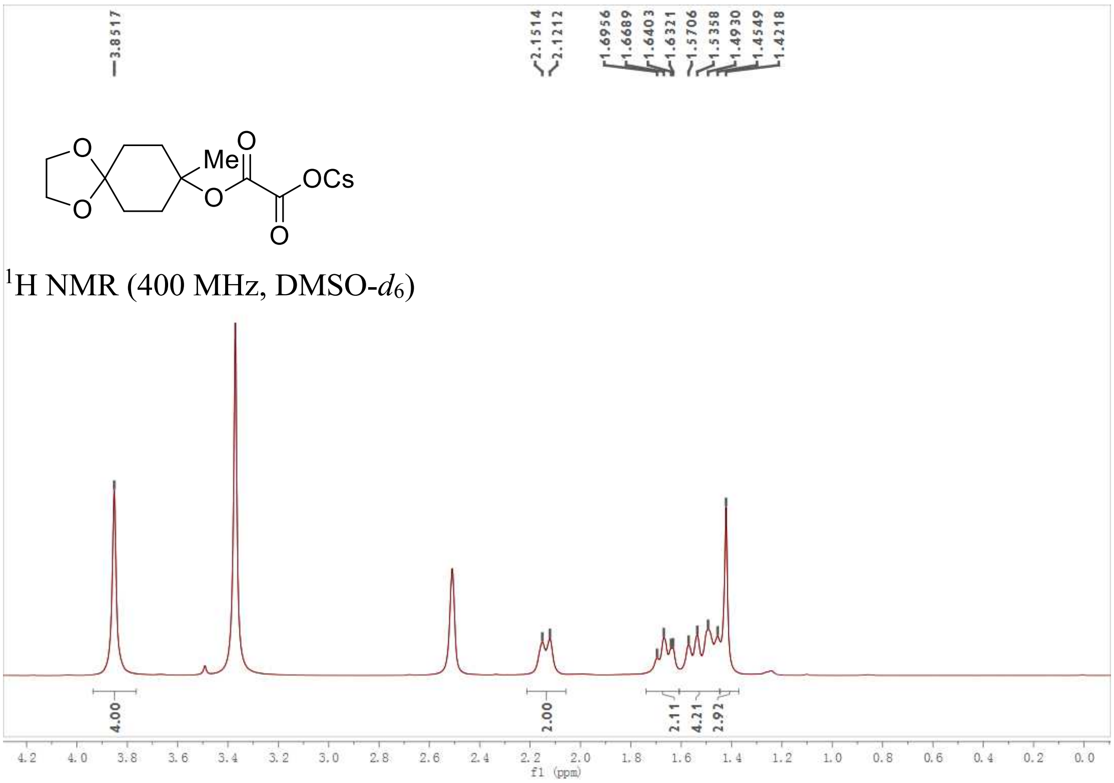

Supplementary Figure 26: ^{1}H NMR Spectra of Cesium-2-((8-methyl-1,4-dioxaspiro[4.5]decan-8-yl)oxy)-2-oxoacetate (S6)

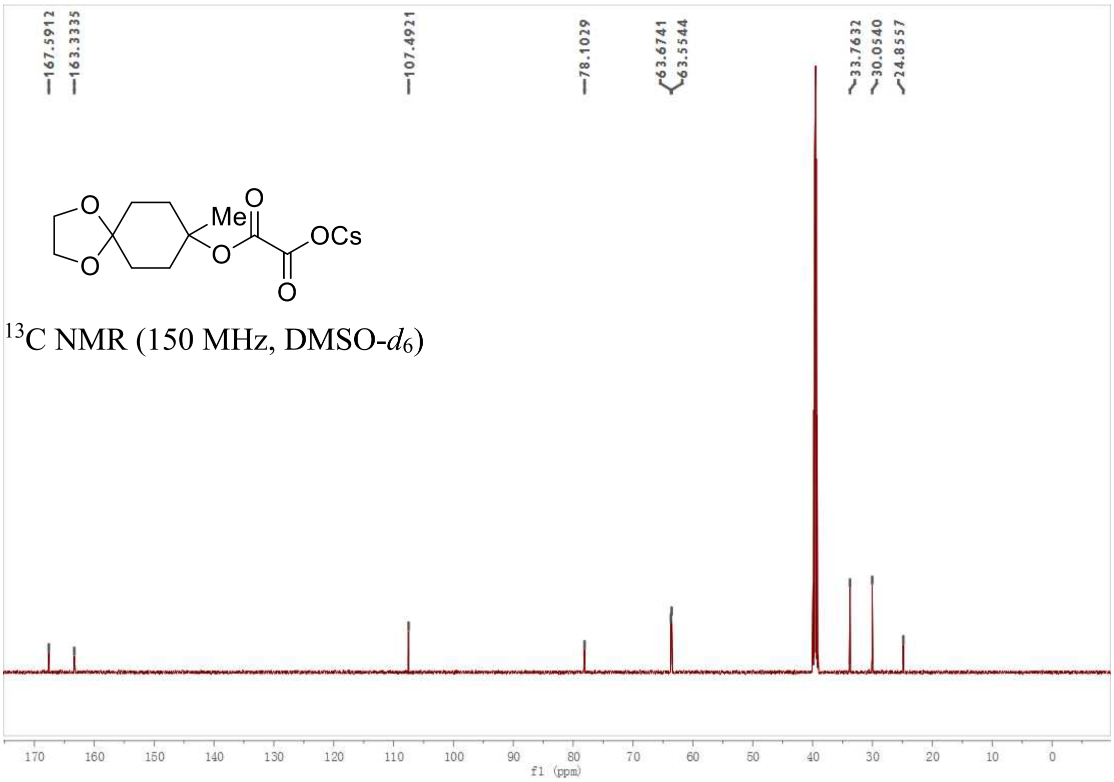

Supplementary Figure 27: ^{13}C NMR Spectra of Cesium-2-((8-methyl-1,4-dioxaspiro[4.5]decan-8-yl)oxy)-2-oxoacetate (S6)

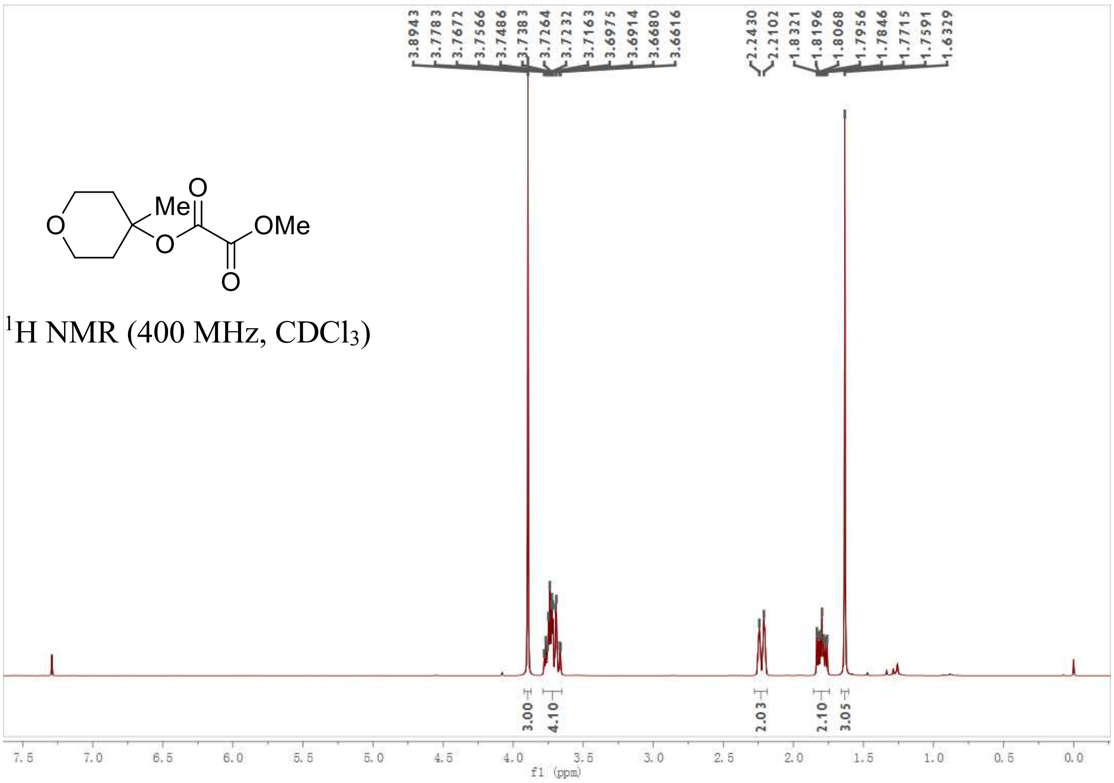

Supplementary Figure 28: ^{1}H NMR Spectra of Methyl (4-methyltetrahydro-2H-pyran-4-yl) oxalate (S7)

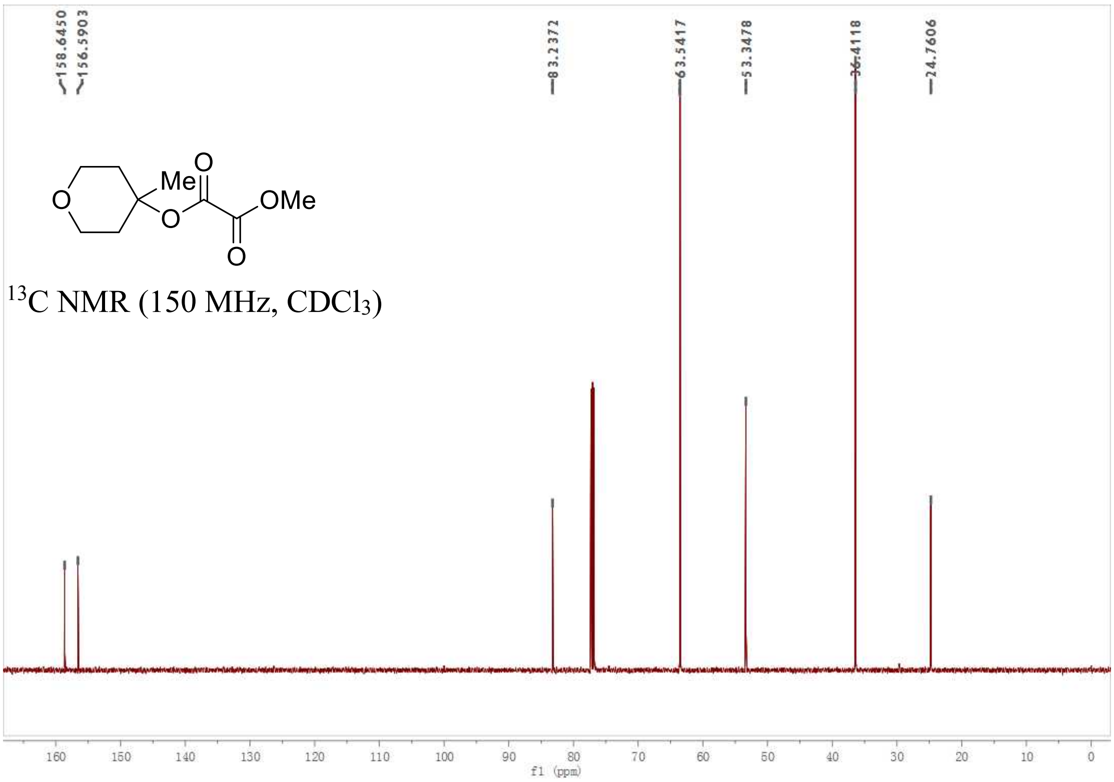

Supplementary Figure 29: ^{13}C NMR Spectra of Methyl (4-methyltetrahydro-2H-pyran-4-yl) oxalate (S7)

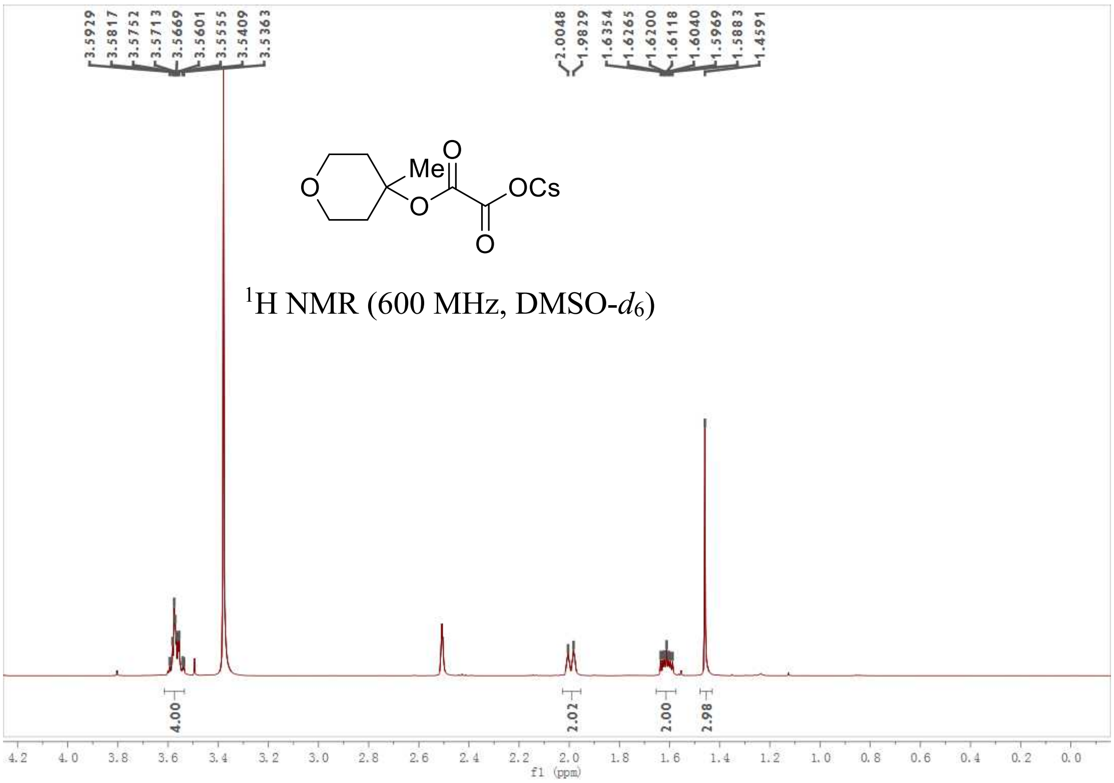

Supplementary Figure 30: ^{1}H NMR Spectra of Cesium 2-((4-methyltetrahydro-2H-pyran-4-yl)oxy)-2-oxoacetate (S8)

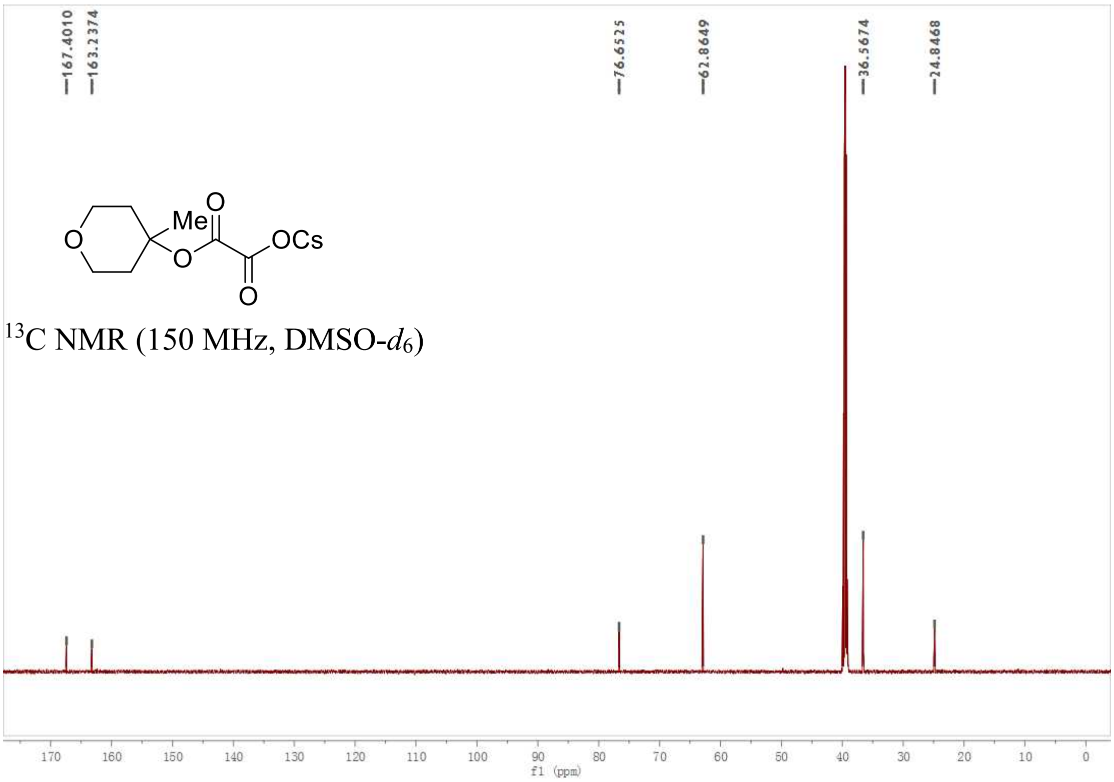

Supplementary Figure 31: ^{13}C NMR Spectra of Cesium 2-((4-methyltetrahydro-2H-pyran-4-yl)oxy)-2-oxoacetate (S8)

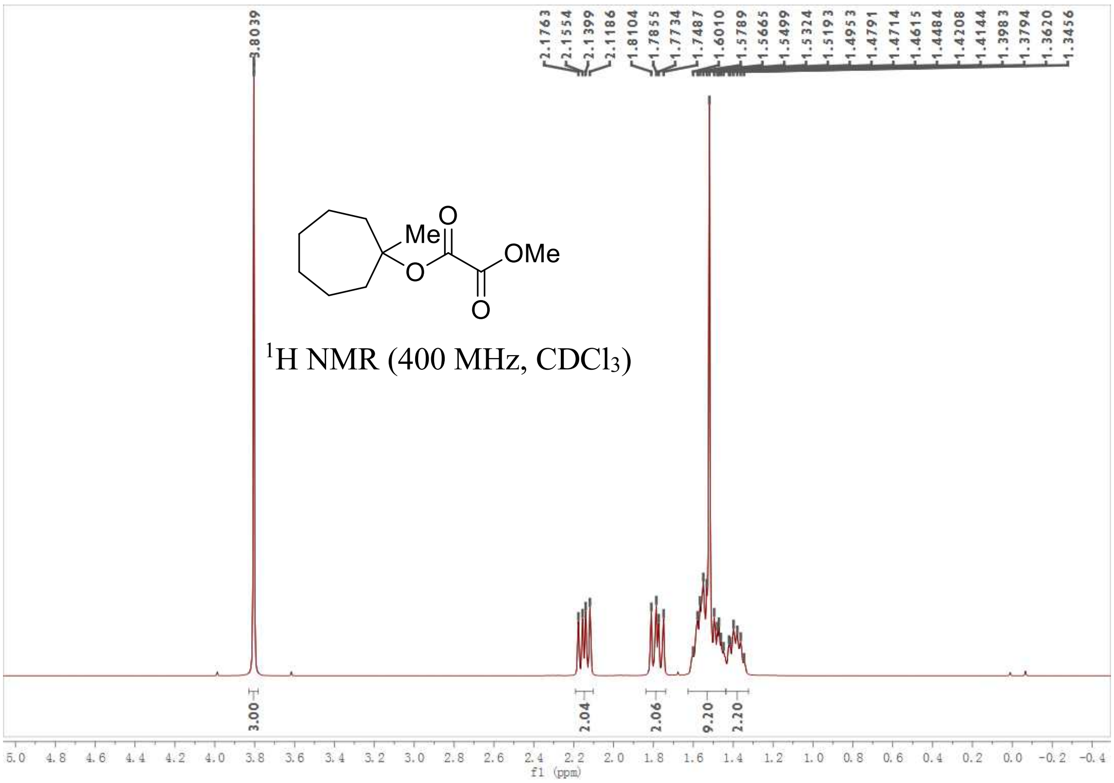

Supplementary Figure 32: ^{1}H NMR Spectra of Methyl (1-methylcycloheptyl) oxalate (S9)

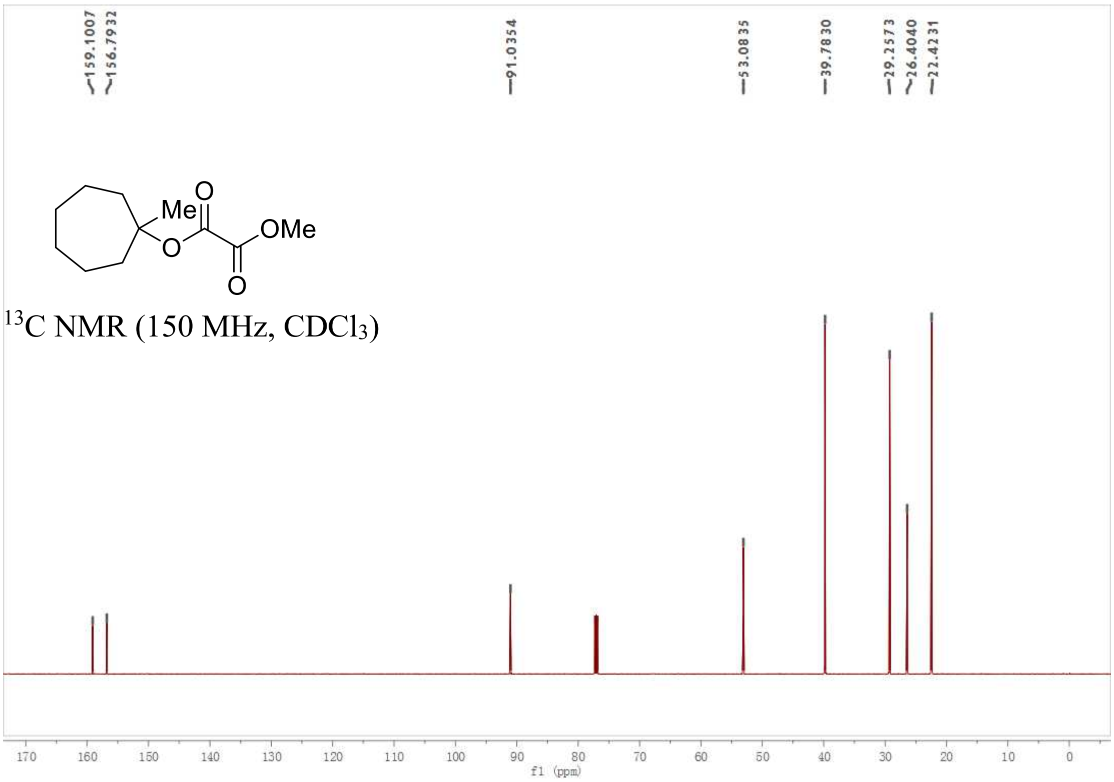

Supplementary Figure 33: ^{13}C NMR Spectra of Methyl (1-methylcycloheptyl) oxalate (S9)

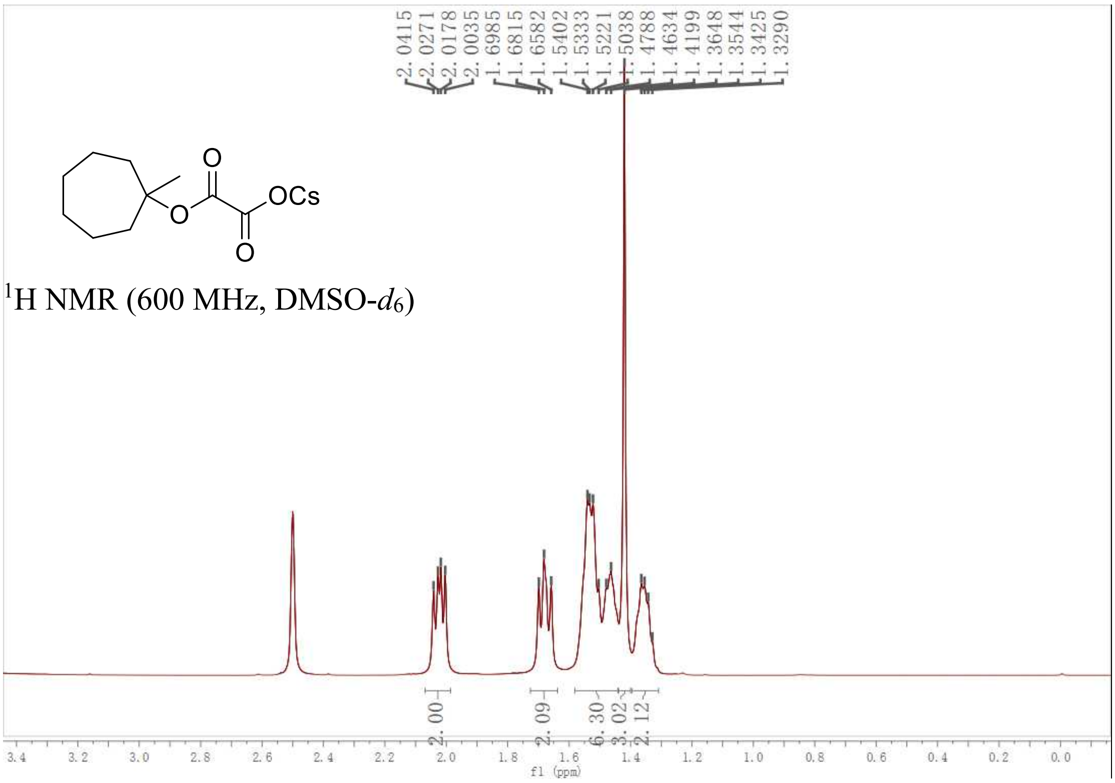

Supplementary Figure 34: ^{1}H NMR Spectra of Cesium 2-((1-methylcycloheptyl)oxy)-2-oxoacetate (S10)

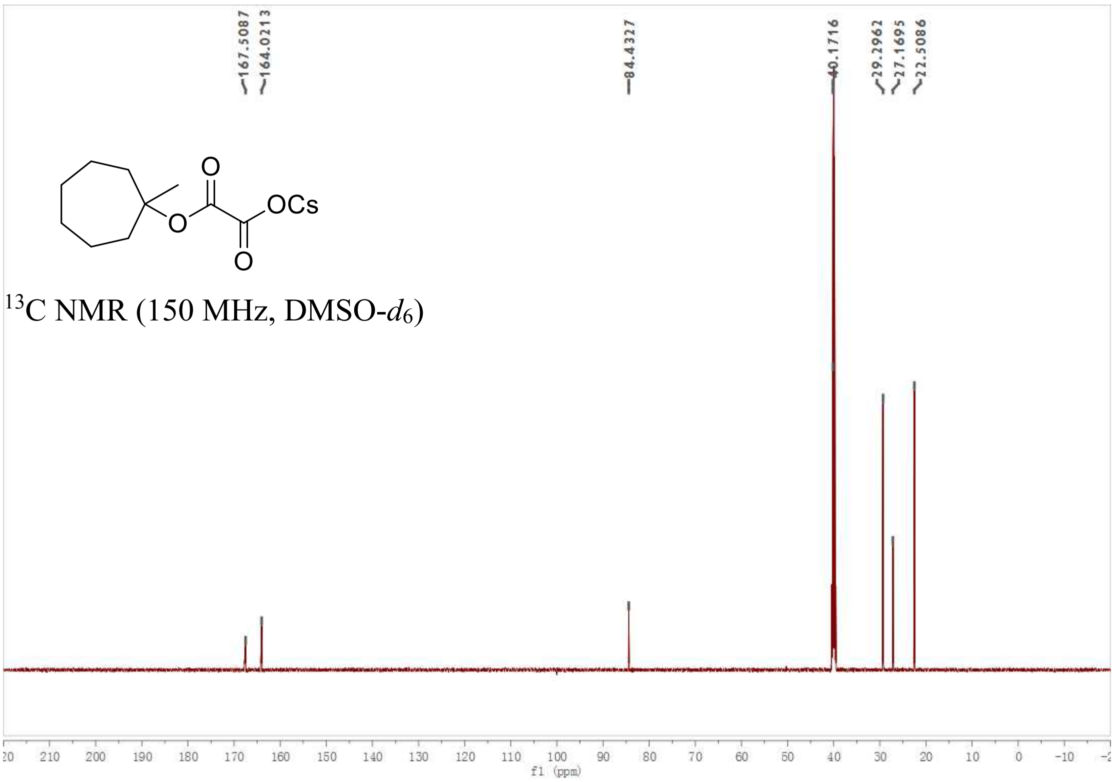

Supplementary Figure 35: ^{13}C NMR Spectra of Cesium 2-((1-methylcycloheptyl)oxy)-2-oxoacetate (S10)

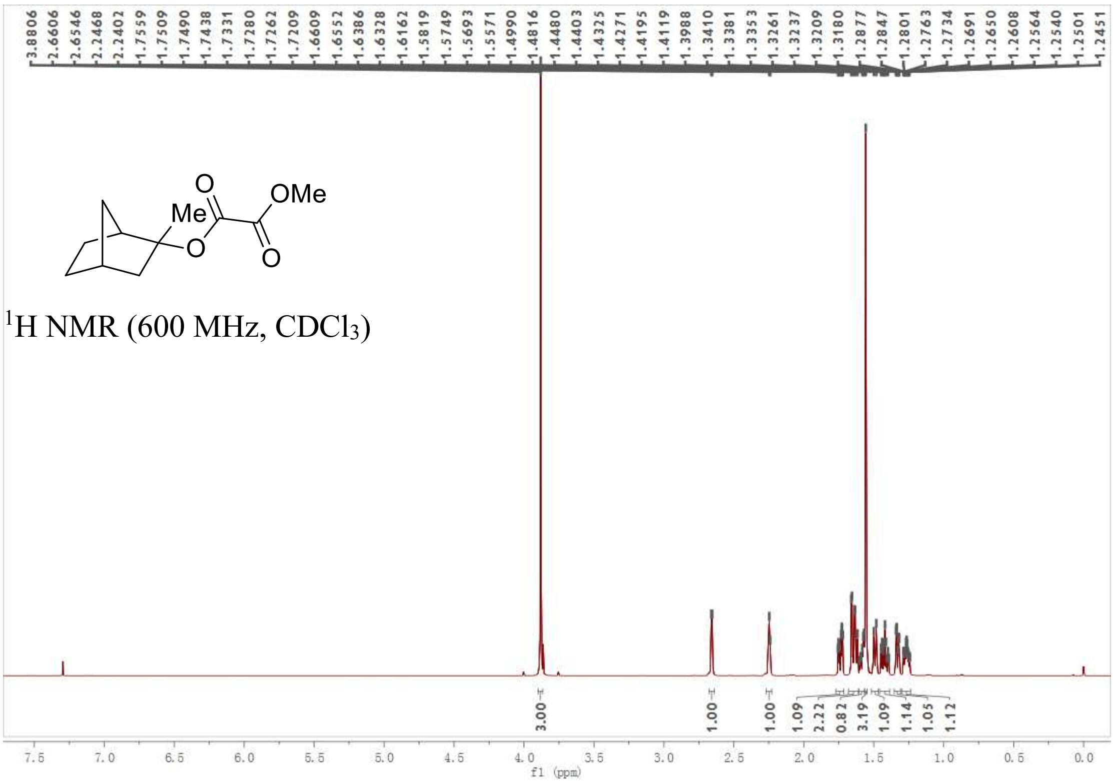

Supplementary Figure 36: ^{1}H NMR Spectra of Methyl ((1S,2R,4R)-2-methylbicyclo[2.2.1]heptan-2-yl) oxalate (S11)

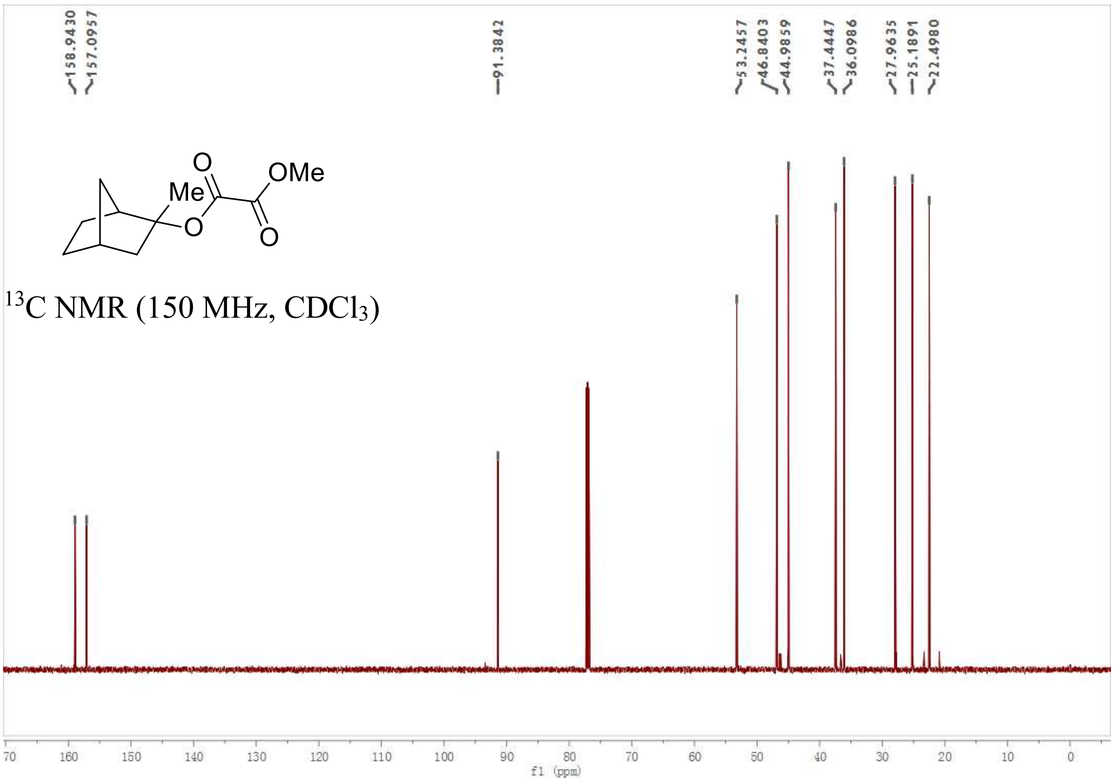

Supplementary Figure 37: ^{13}C NMR Spectra of Methyl ((1S,2R,4R)-2-methylbicyclo[2.2.1]heptan-2-yl) oxalate (S11)

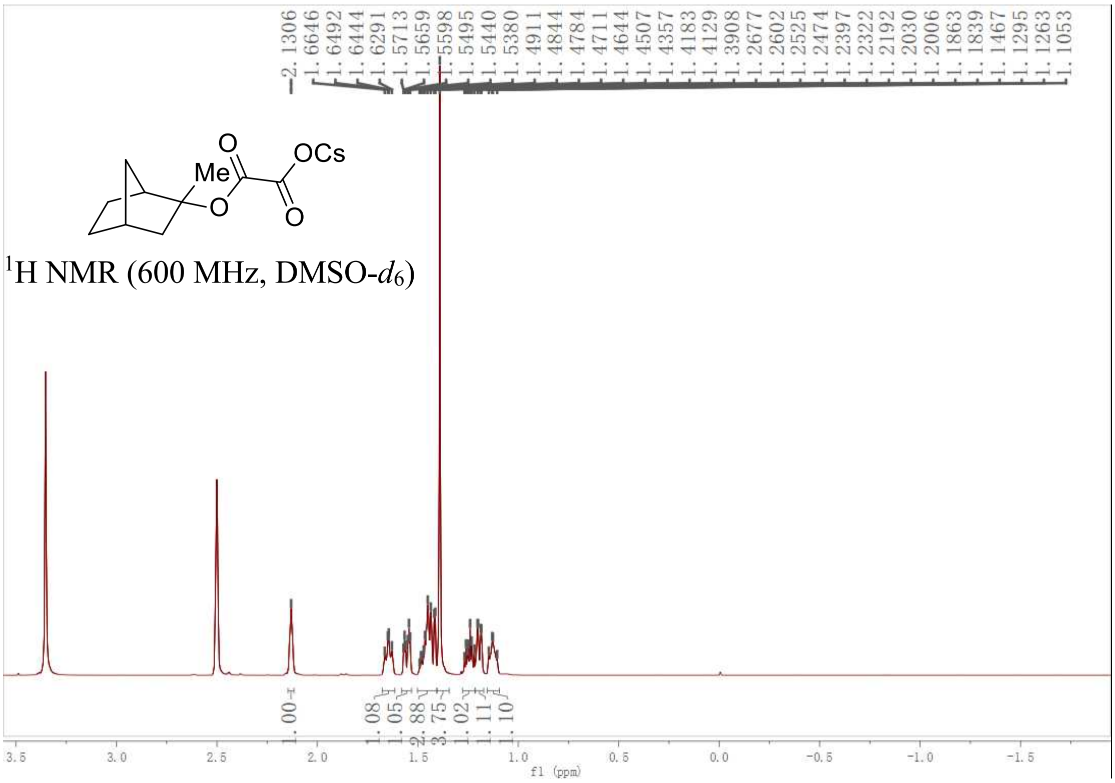

Supplementary Figure 38: ^{1}H NMR Spectra of Cesium 2-(((1S,2R,4R)-2-methylbicyclo[2.2.1]heptan-2-yl)oxy)-2-oxoacetate (S12)

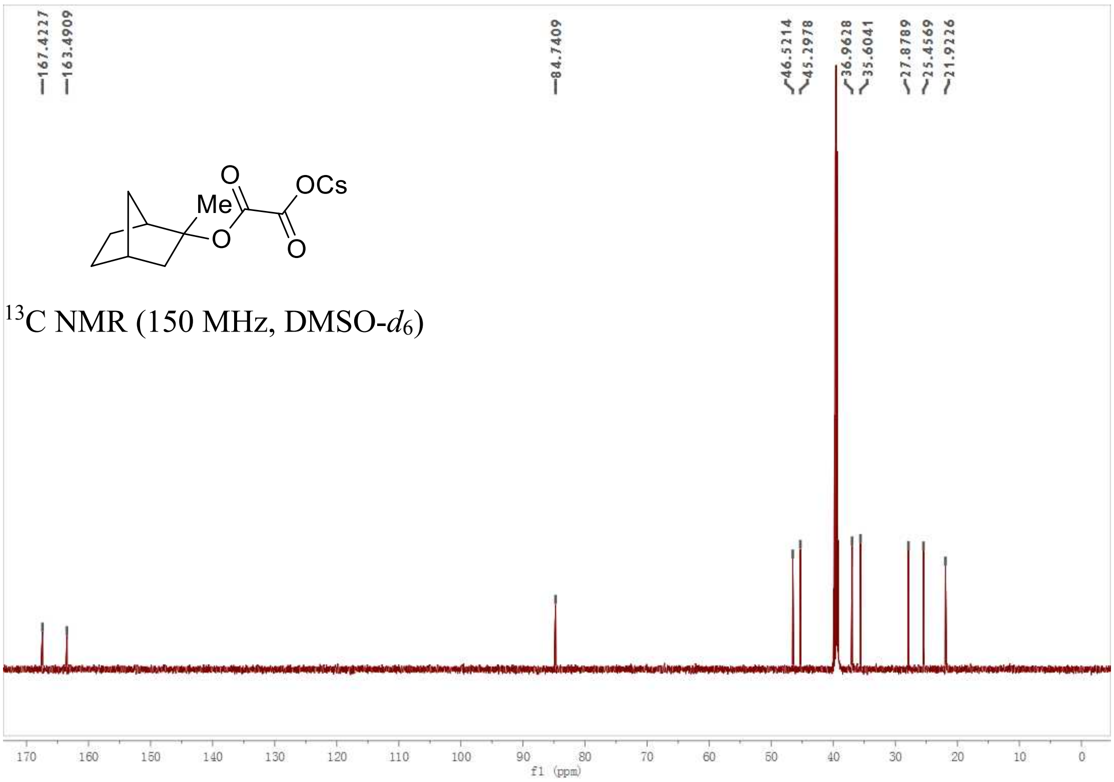

Supplementary Figure 39: ^{13}C NMR Spectra of Cesium 2-(((1S,2R,4R)-2-methylbicyclo[2.2.1]heptan-2-yl)oxy)-2-oxoacetate (S12)

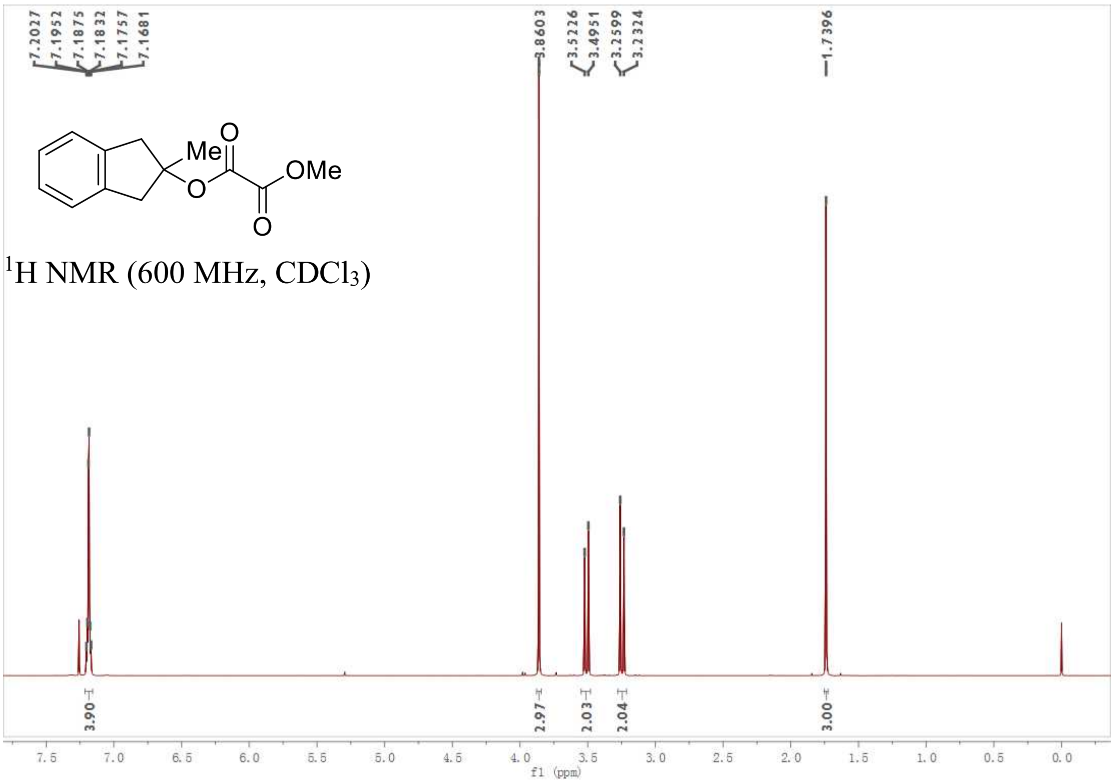

Supplementary Figure 40: ^{1}H NMR Spectra of Methyl (2-methyl-2,3-dihydro-1H-inden-2-yl) oxalate (S13)

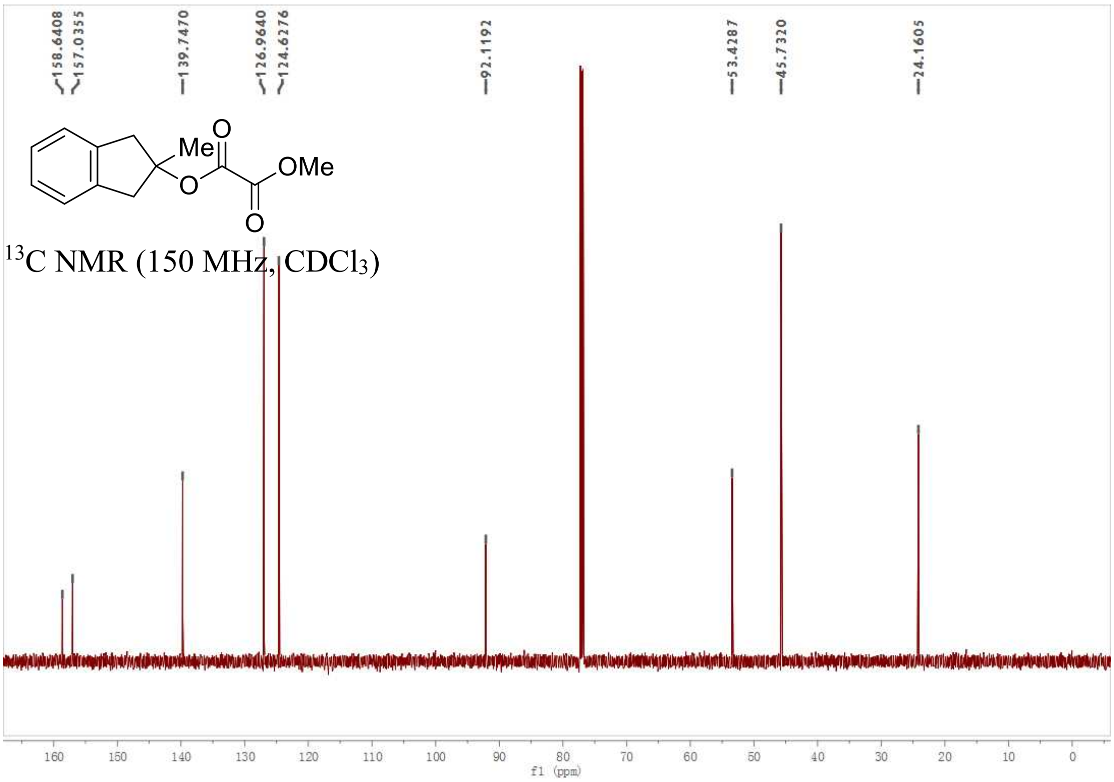

Supplementary Figure 41: ^{13}C NMR Spectra of Methyl (2-methyl-2,3-dihydro-1H-inden-2-yl) oxalate (S13)

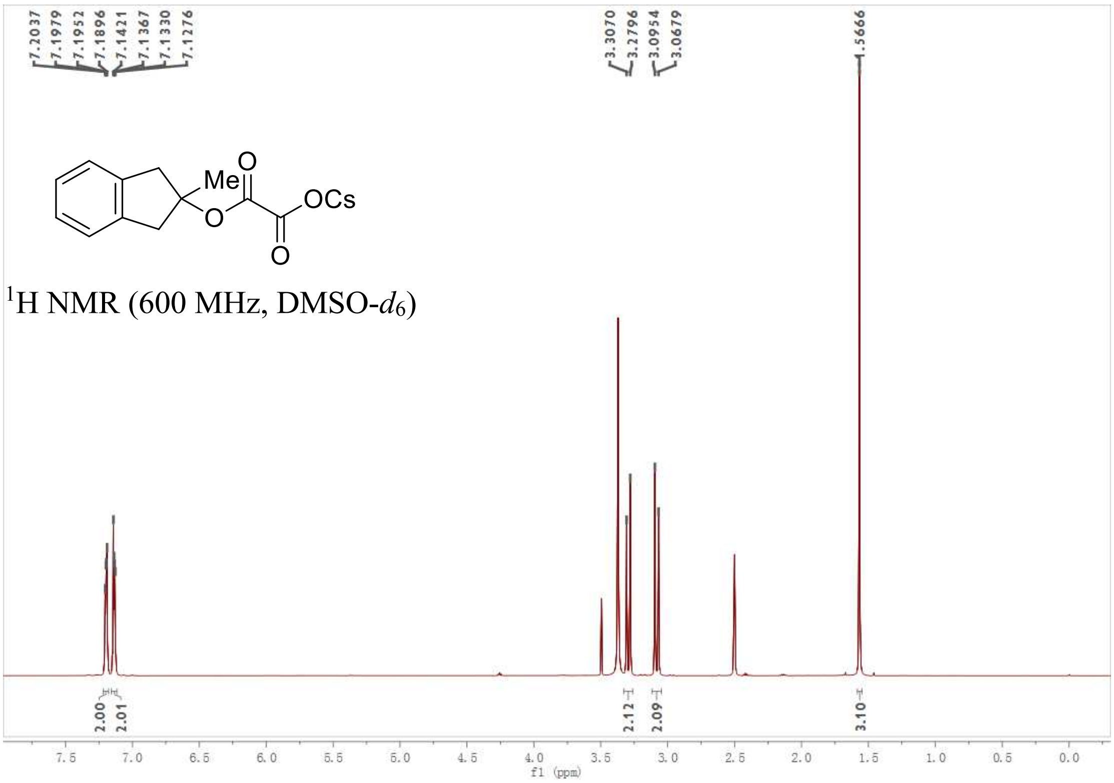

Supplementary Figure 42: ^{1}H NMR Spectra of Cesium 2-((2-methyl-2,3-dihydro-1H-inden-2-yl)oxy)-2-oxoacetate (S14)

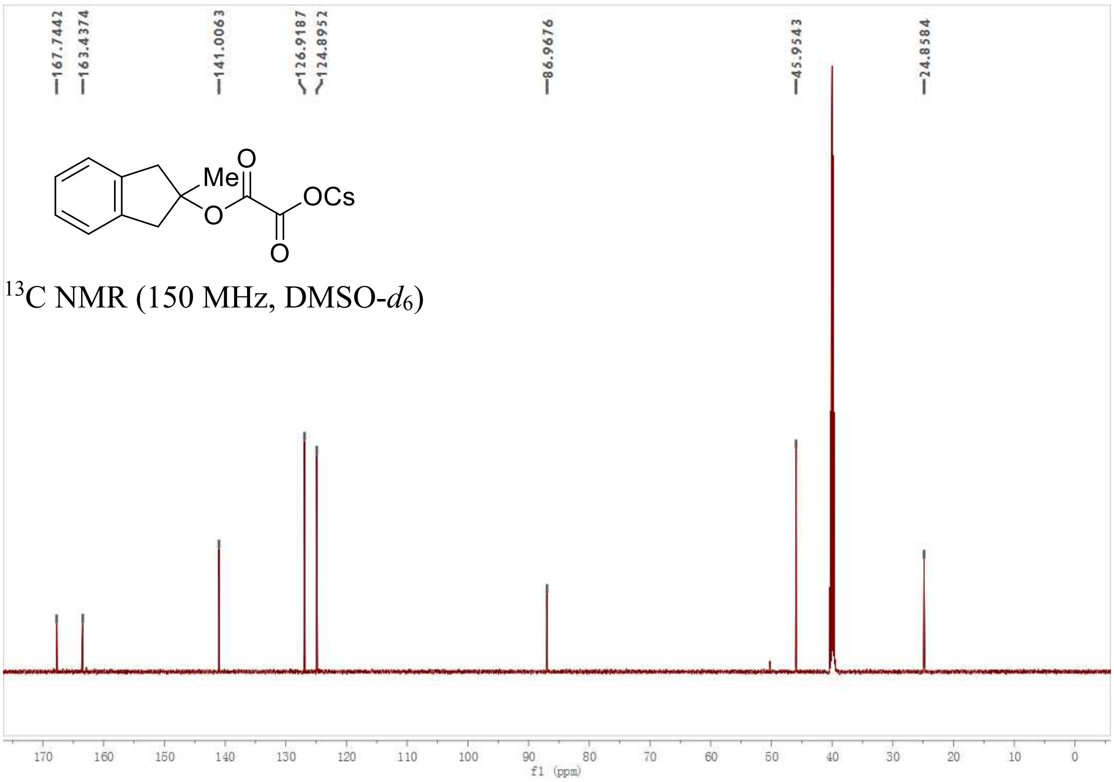

Supplementary Figure 43: ^{13}C NMR Spectra of Cesium 2-((2-methyl-2,3-dihydro-1H-inden-2-yl)oxy)-2-oxoacetate (S14)

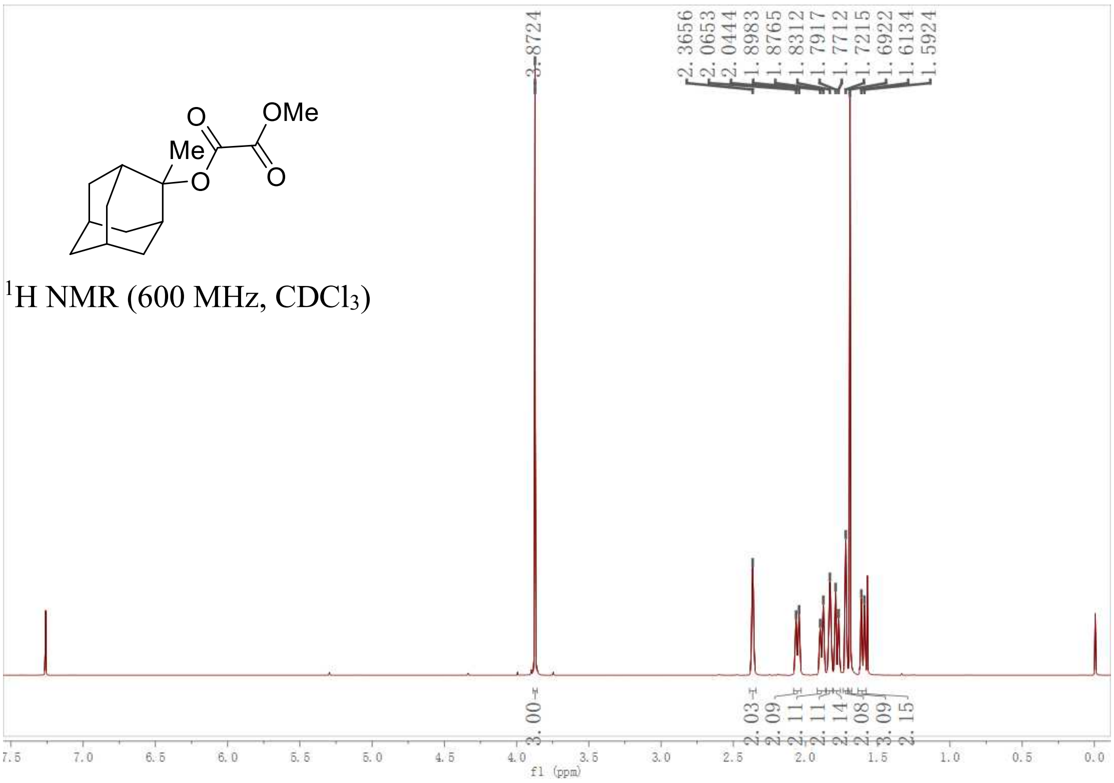

Supplementary Figure 44: ^{1}H NMR Spectra of Methyl ((1r,3r,5r,7r)-2-methyladamantan-2-yl) oxalate (S15)

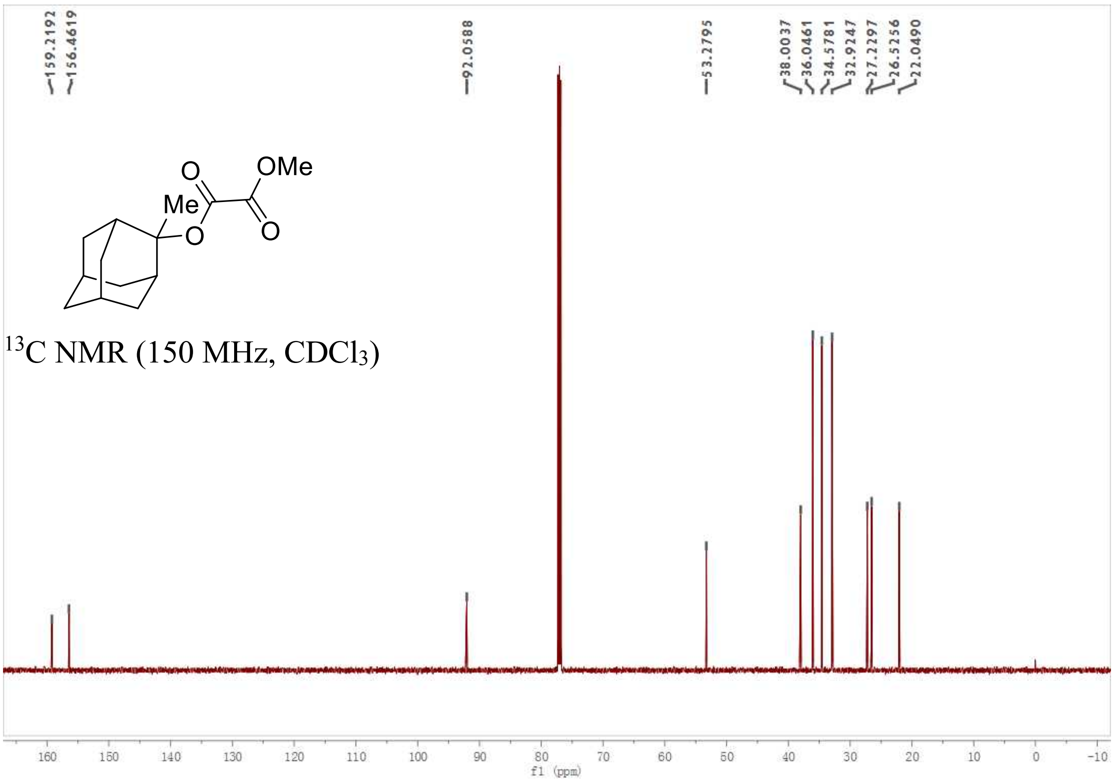

Supplementary Figure 45: ^{13}C NMR Spectra of Methyl ((1r,3r,5r,7r)-2-methyladamantan-2-yl) oxalate (S15)

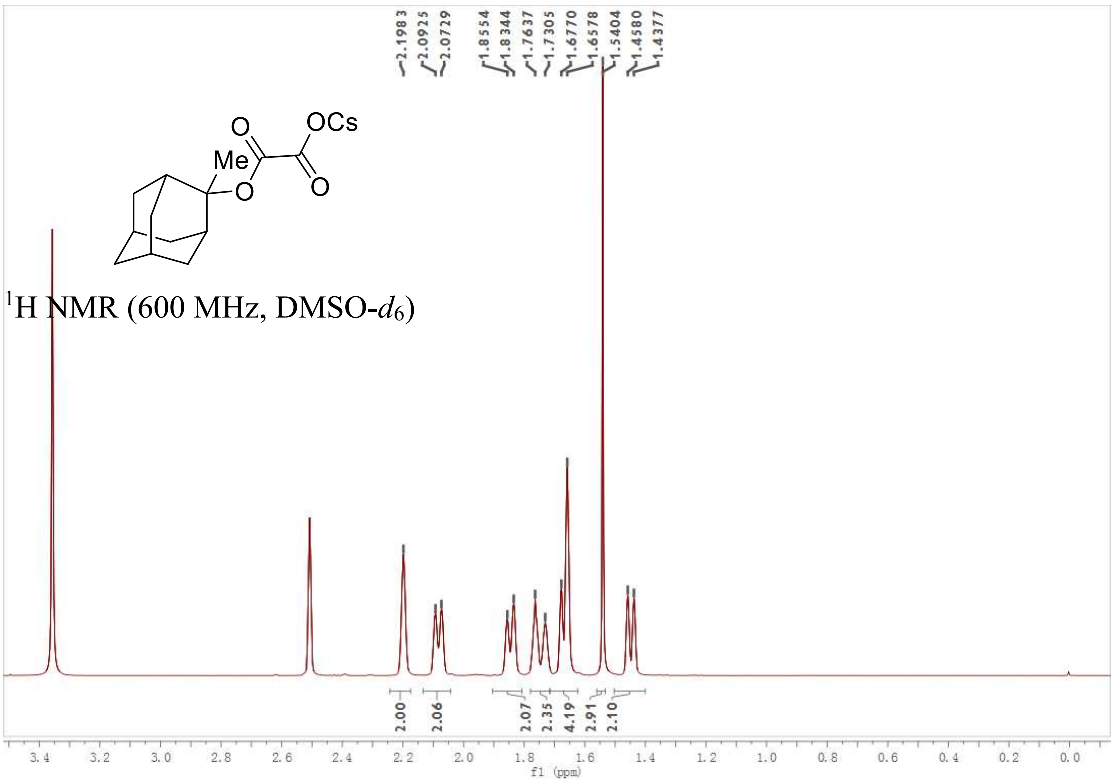

Supplementary Figure 46: ^{1}H NMR Spectra of Cesium 2-(((1r,3r,5r,7r)-2-methyladamantan-2-yl)oxy)-2-oxoacetate (S16)

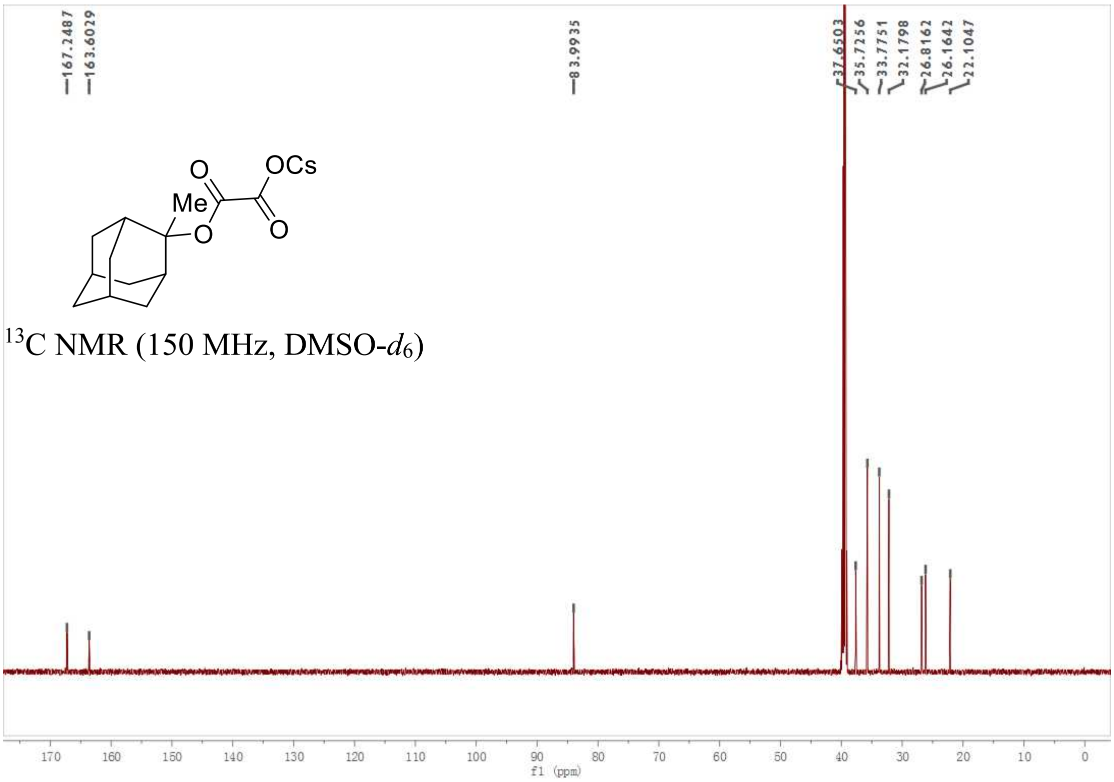

Supplementary Figure 47: ^{13}C NMR Spectra of Cesium 2-(((1r,3r,5r,7r)-2-methyladamantan-2-yl)oxy)-2-oxoacetate (S16)

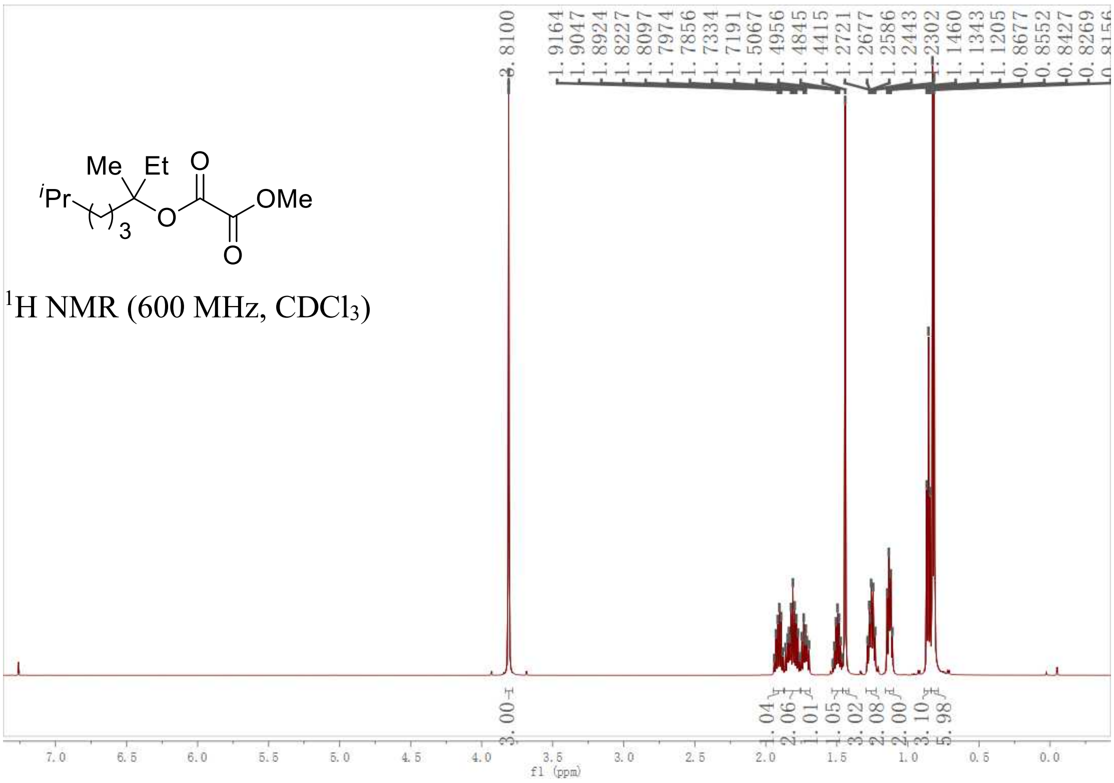

Supplementary Figure 48: ^{1}H NMR Spectra of 3,7-Dimethyloctan-3-yl methyl oxalate (S17)

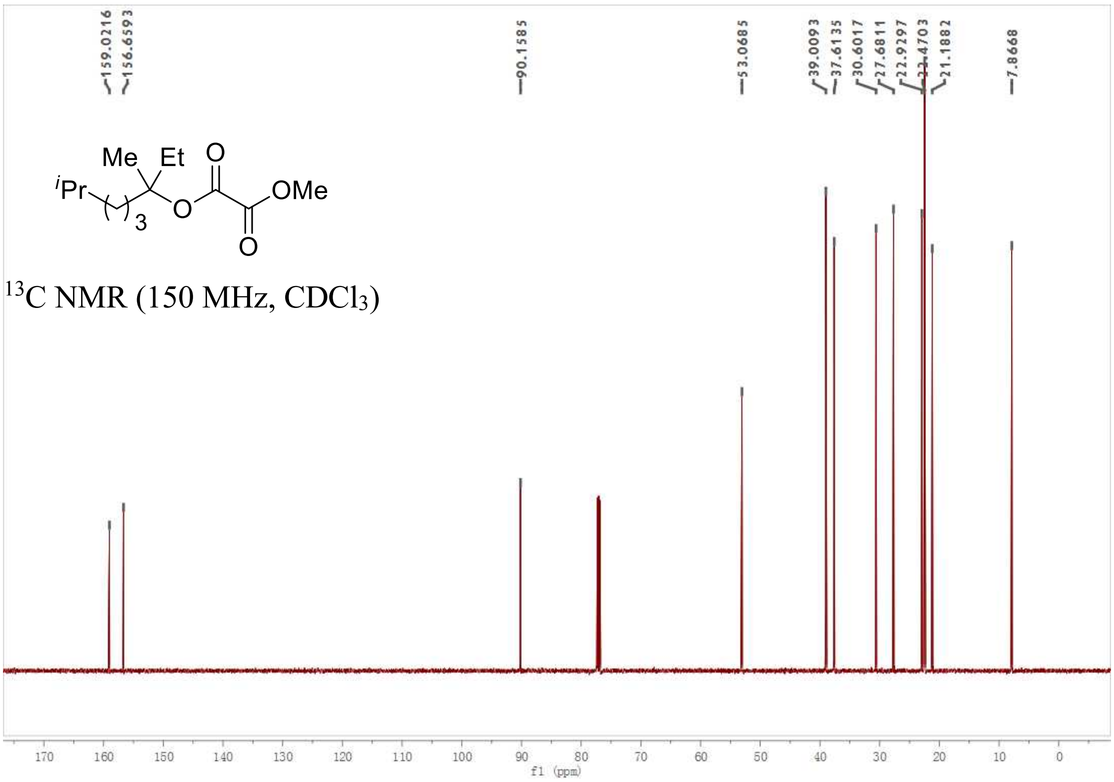

Supplementary Figure 49: ^{13}C NMR Spectra of 3,7-Dimethyloctan-3-yl methyl oxalate (S17)

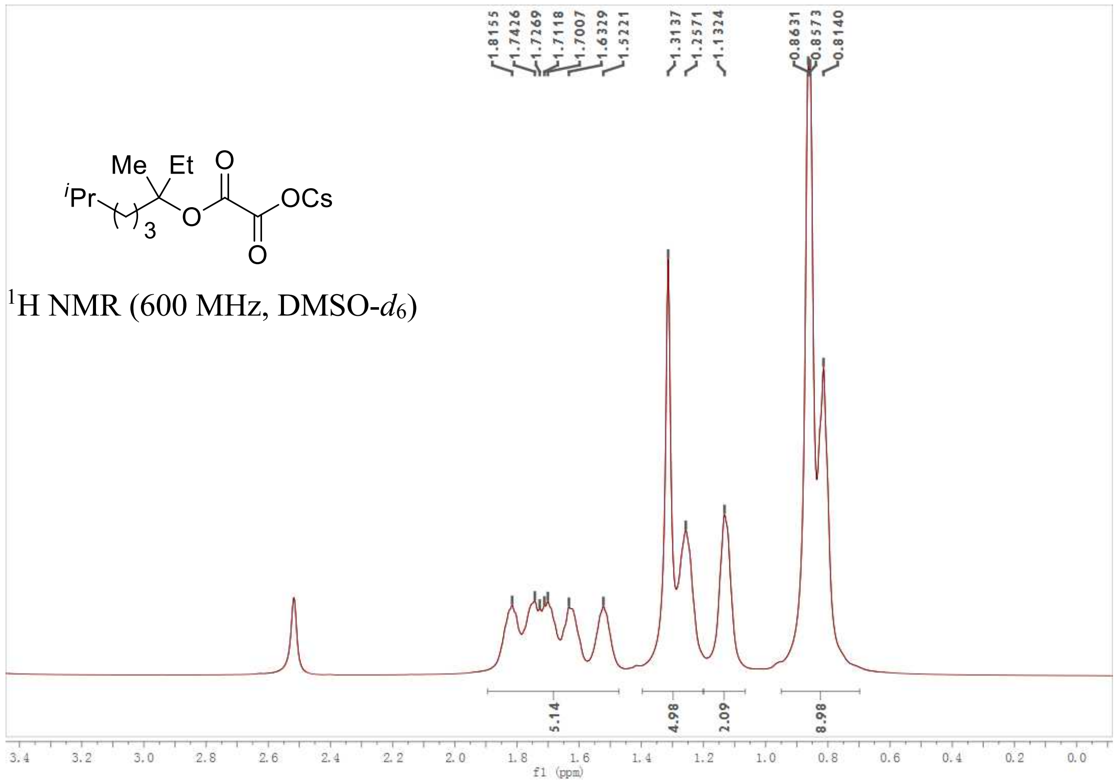

Supplementary Figure 50: ^{1}H NMR Spectra of Cesium 2-((3,7-dimethyloctan-3-yl)oxy)-2-oxoacetate (S18)

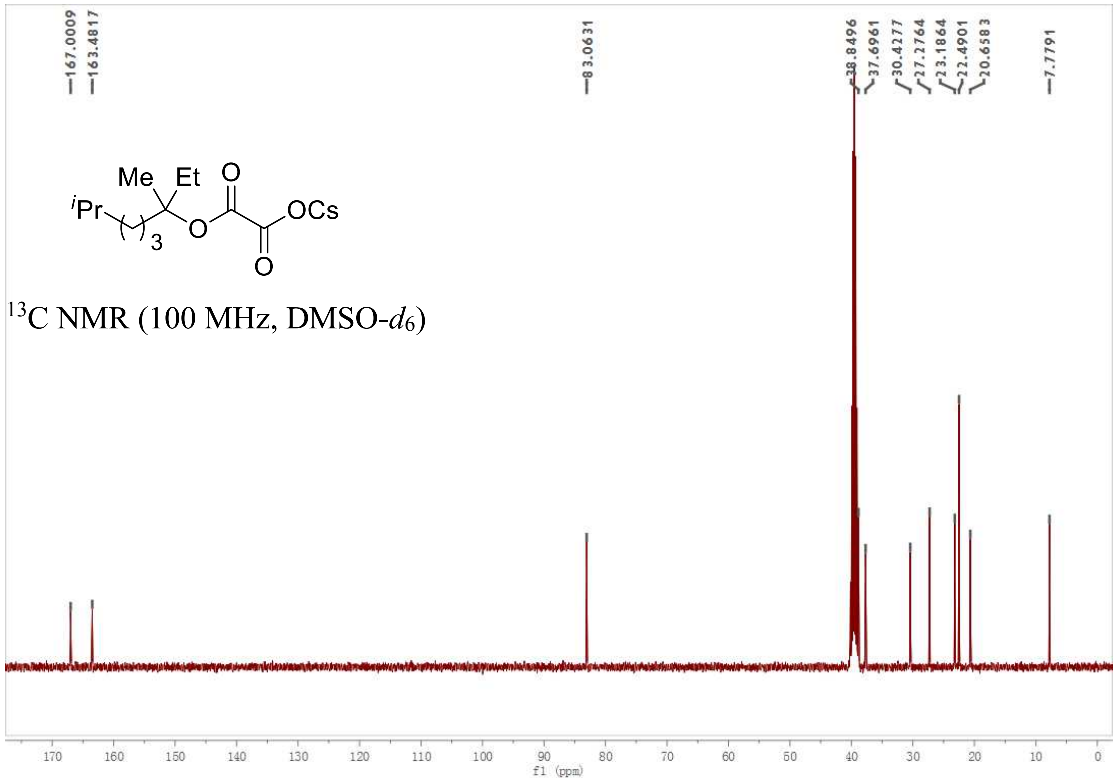

Supplementary Figure 51: ^{13}C NMR Spectra of Cesium 2-((3,7-dimethyloctan-3-yl)oxy)-2-oxoacetate (S18)

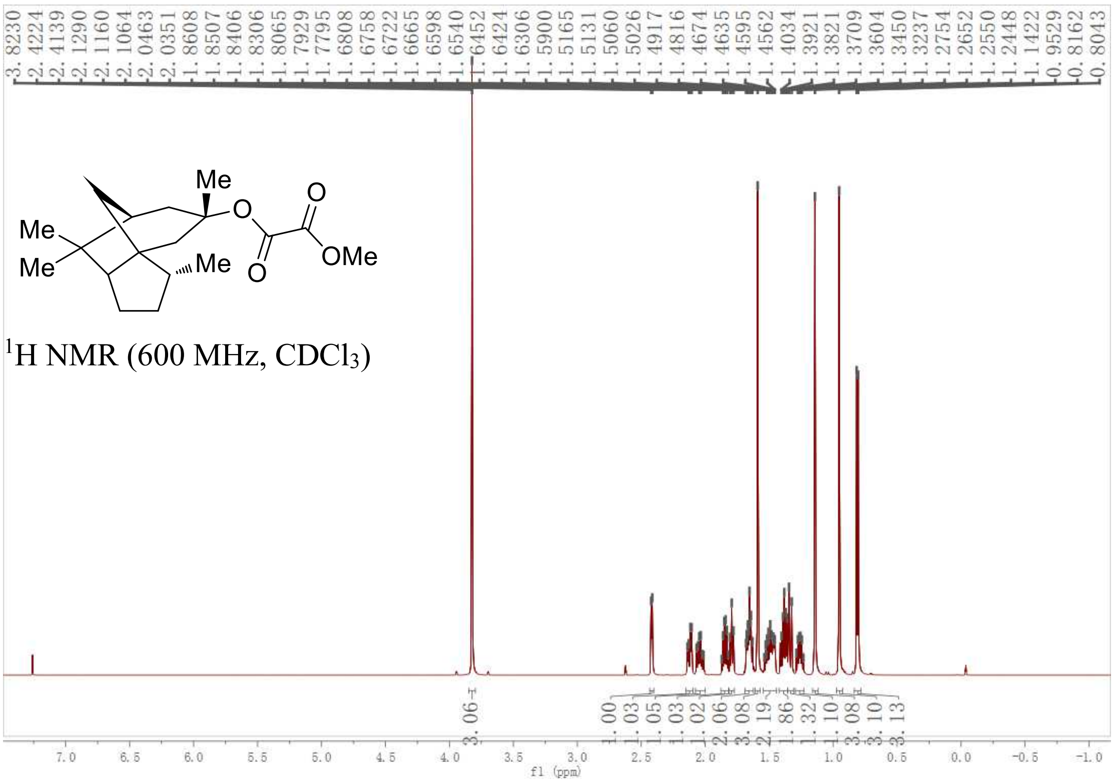

Supplementary Figure 52: ^{1}H NMR Spectra of Methyl((3R,3aS,5S,7S)-3,5,8,8 -tetramethyloctahydro-1H-3a,7-methanoazulen-5-yl) oxalate (S19)

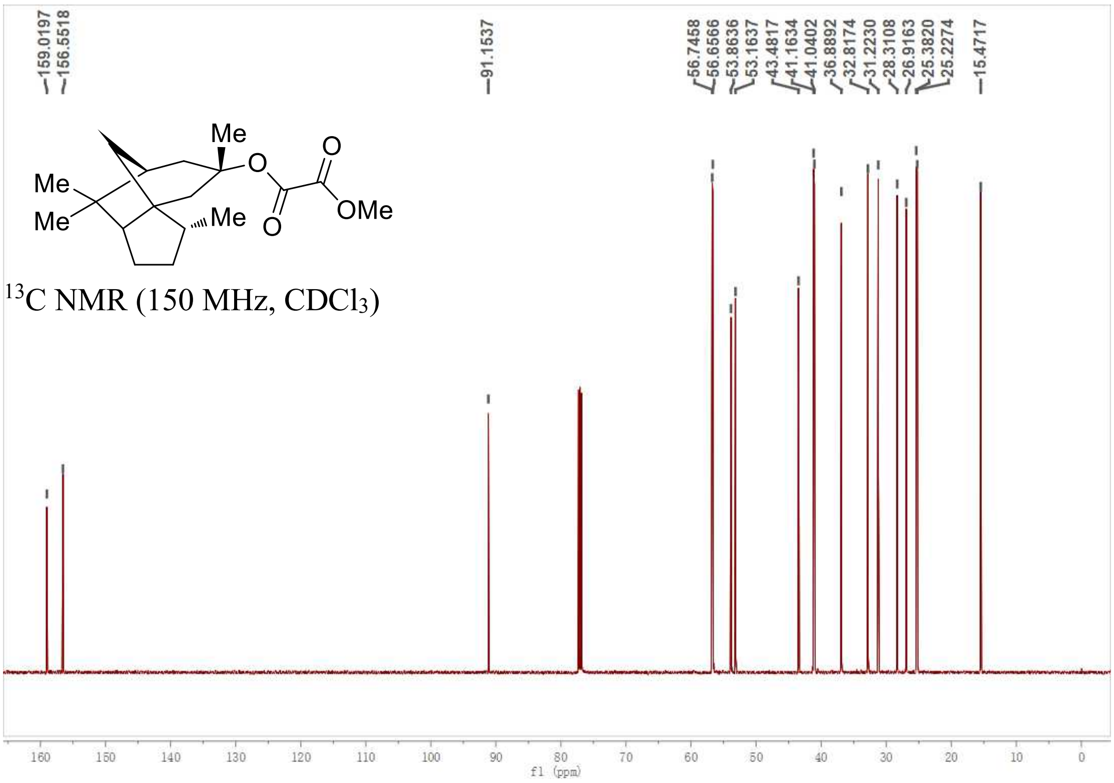

Supplementary Figure 53: ^{13}C NMR Spectra of Methyl((3R,3aS,5S,7S)-3,5,8,8-tetramethyloctahydro-1H-3a,7-methanoazulen-5-yl) oxalate (S19)

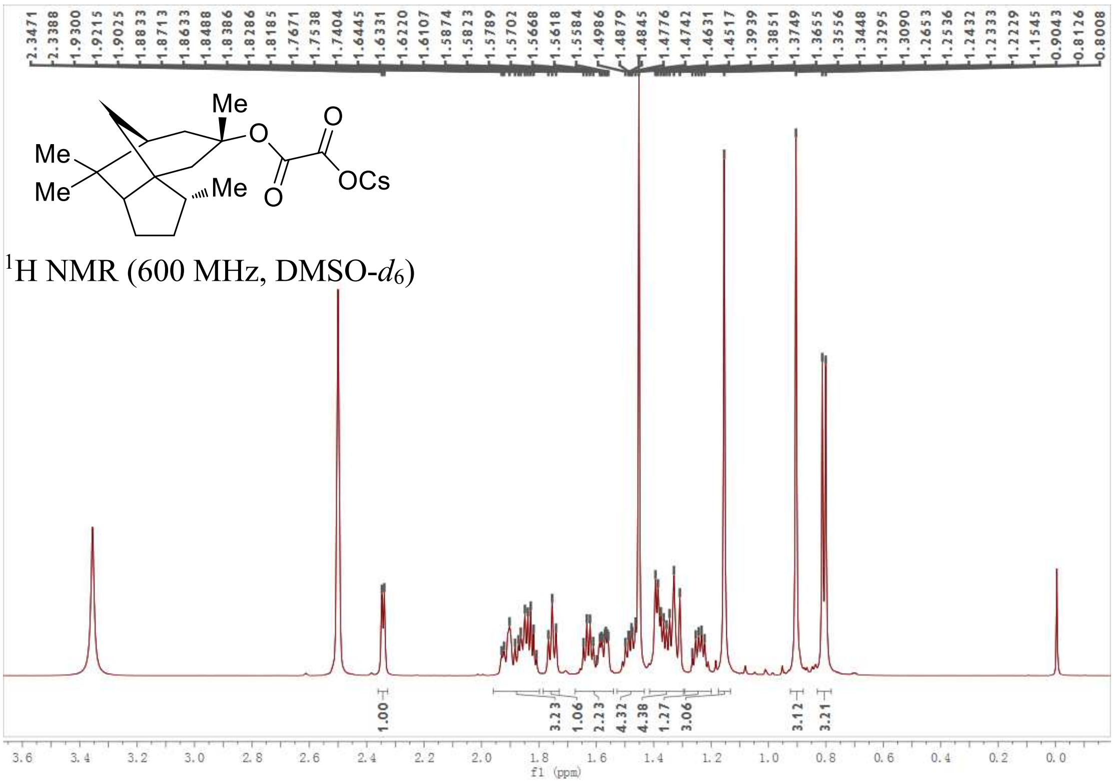

Supplementary Figure 54: ^{1}H NMR Spectra of Cesium-2-oxo-2-(((3R,3aS,5S,7S)-3,5,8,8-tetramethyloctahydro-1H-3a,7-methanoazulen-5-yl)oxy) acetate (S20)

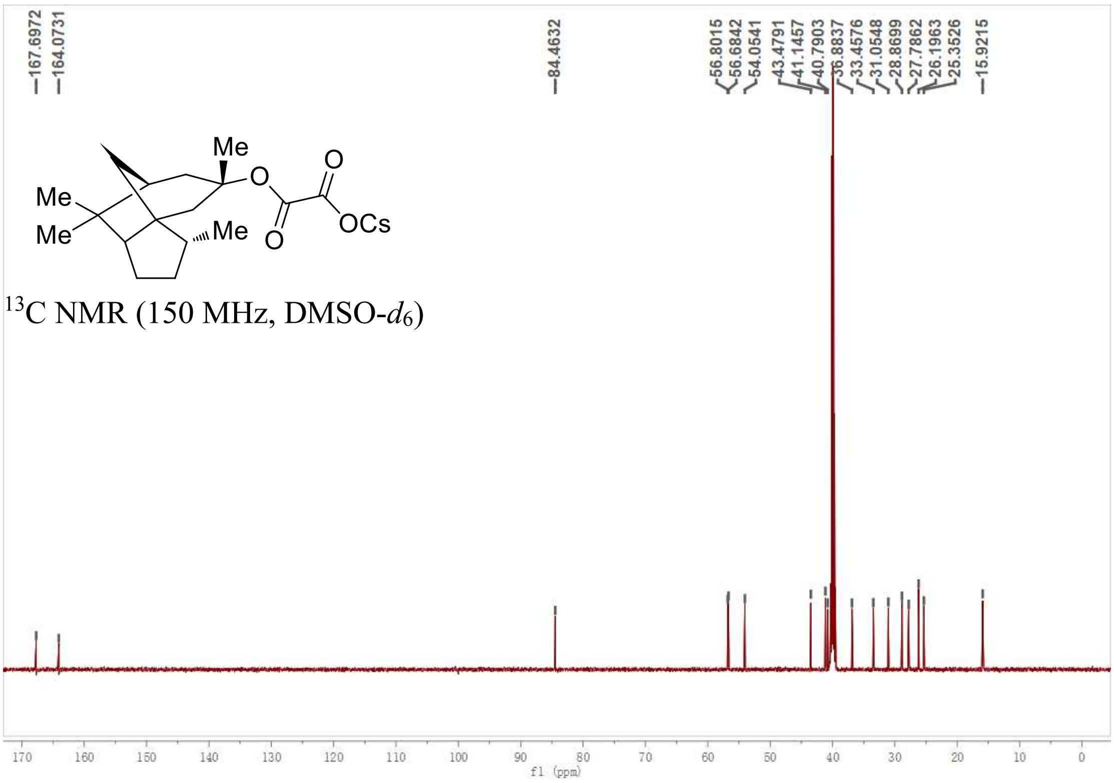

Supplementary Figure 55: ^{13}C NMR Spectra of Cesium-2-oxo-2-(((3R,3aS,5S,7S) -3,5,8,8-tetramethyloctahydro-1H-3a,7-methanoazulen-5-yl)oxy) acetate (S20)

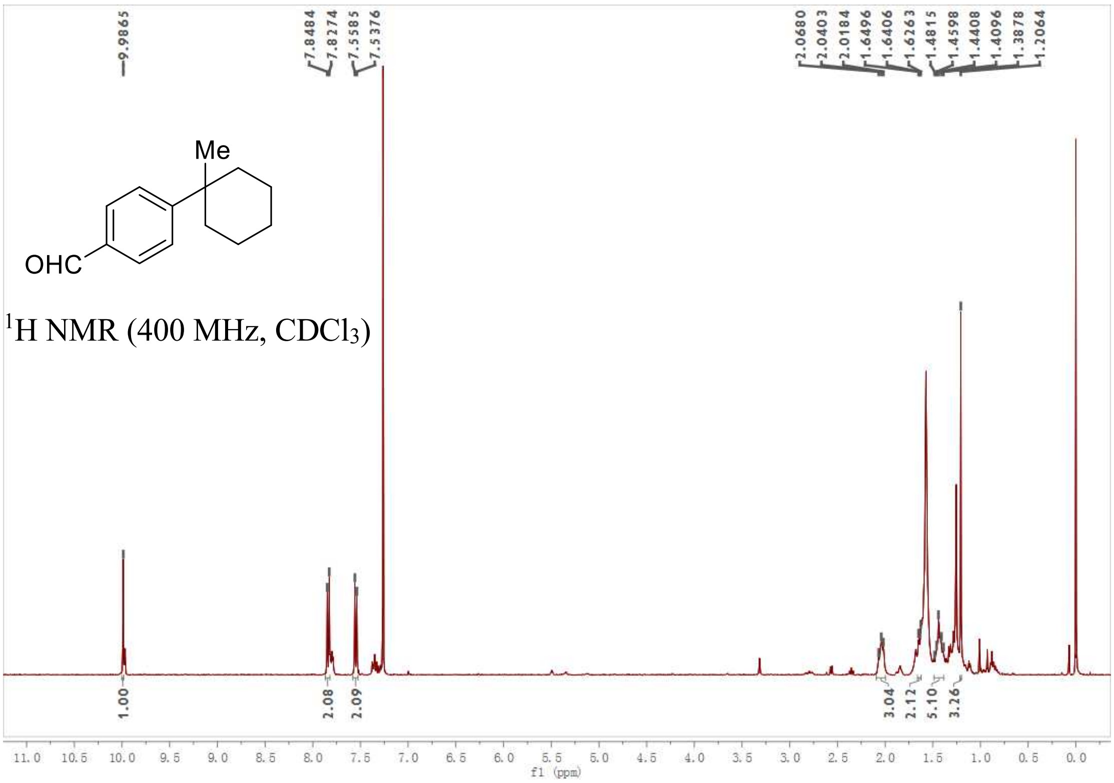

Supplementary Figure 56: ^{1}H NMR Spectra of 4-(1-Methylcyclohexyl)benzaldehyde (S21)

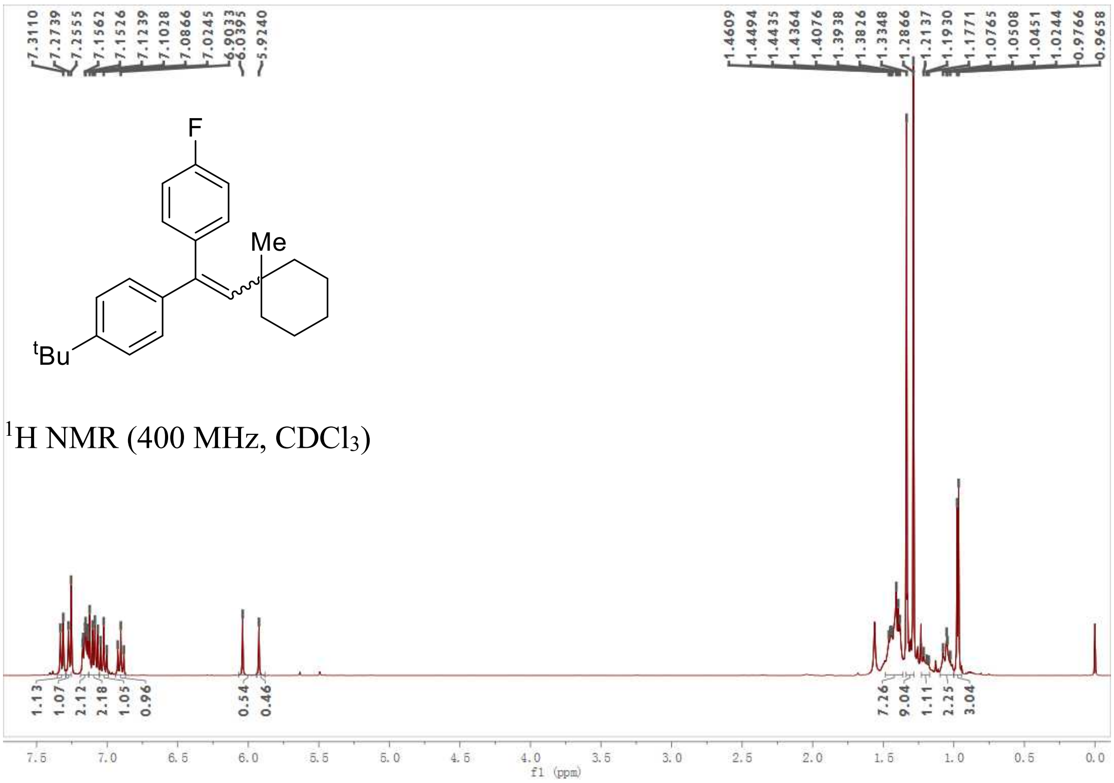

Supplementary Figure 57: ^{1}H NMR Spectra of 1-(tert-Butyl)-4-(1-(4-fluorophenyl)-2-(1-methylcyclohexyl)vinyl)benzene (S22)

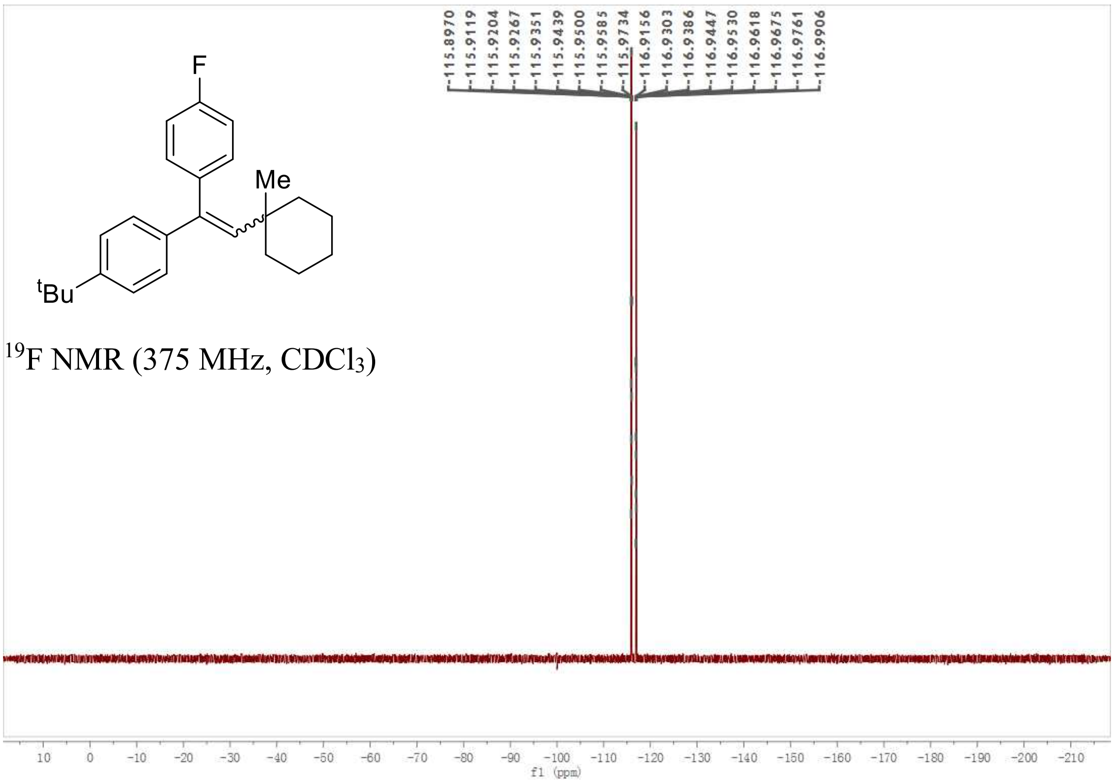

Supplementary Figure 58: ^{19}F NMR Spectra of 1-(tert-Butyl)-4-(1-(4-fluorophenyl)-2-(1-methylcyclohexyl)vinyl)benzene (S22)

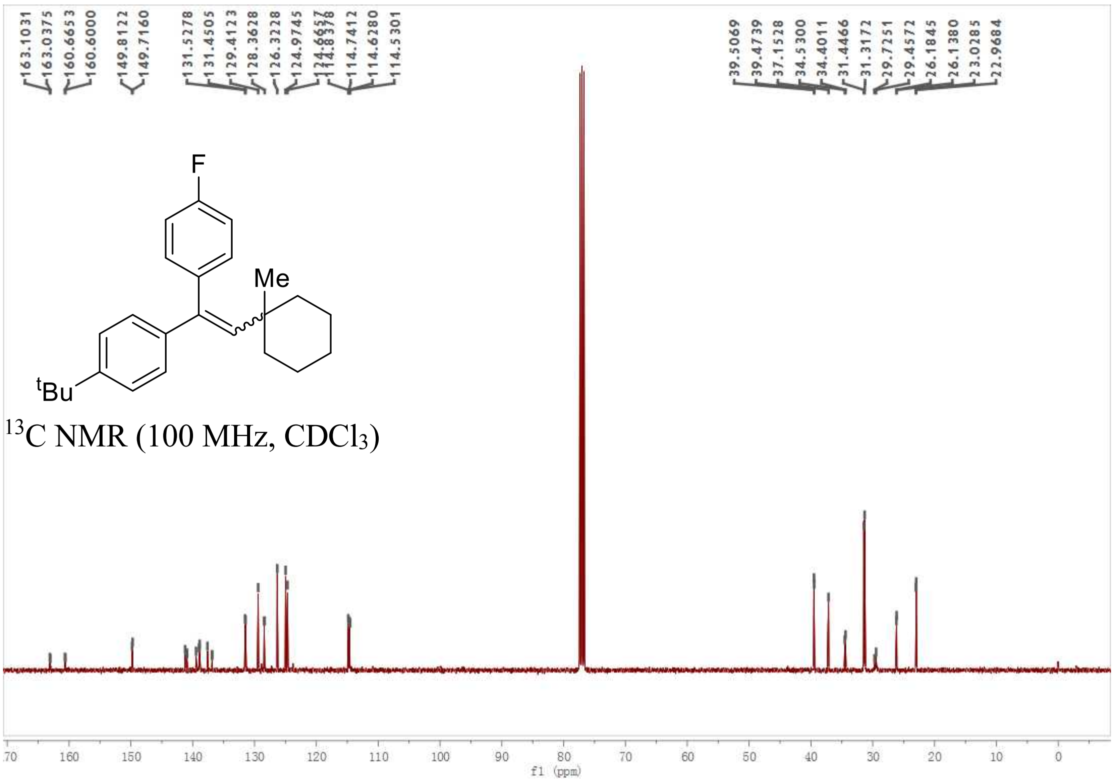

Supplementary Figure 59: ^{13}C NMR Spectra of 1-(tert-Butyl)-4-(1-(4-fluorophenyl)-2-(1-methylcyclohexyl)vinyl)benzene (S22)

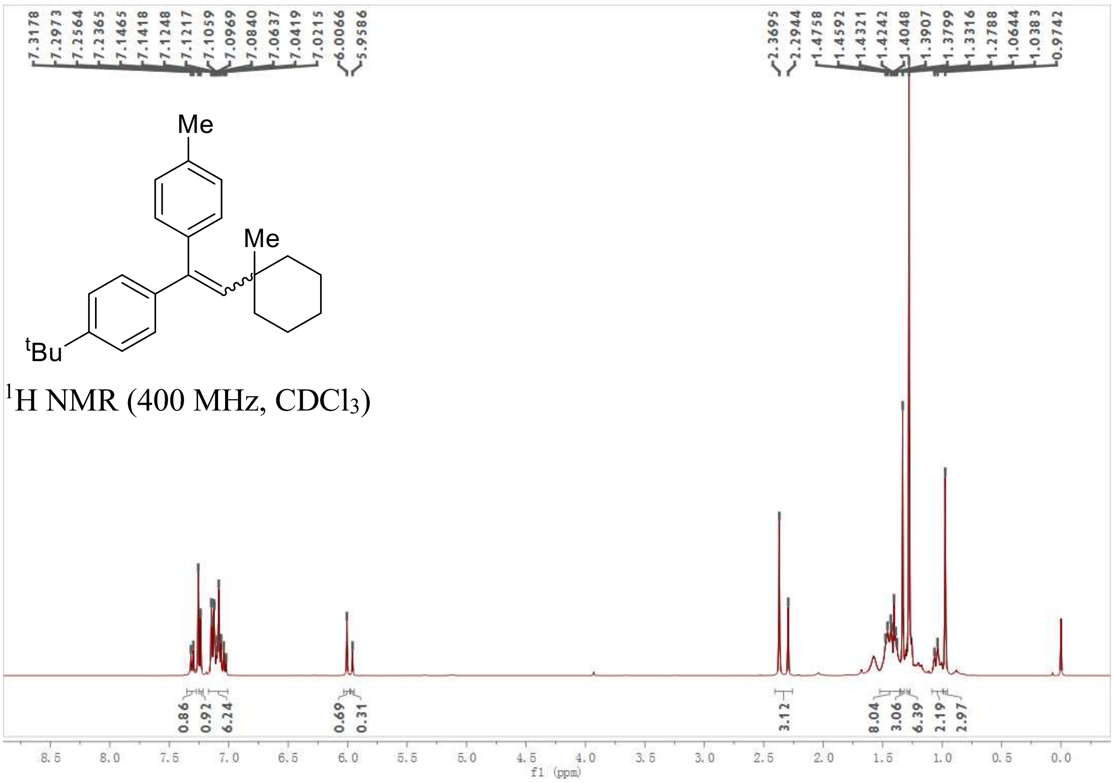

Supplementary Figure 60: ^{1}H NMR Spectra of 1-(tert-Butyl)-4-(2-(1-methylcyclohexyl)-1-(p-tolyl)vinyl)benzene (S23)

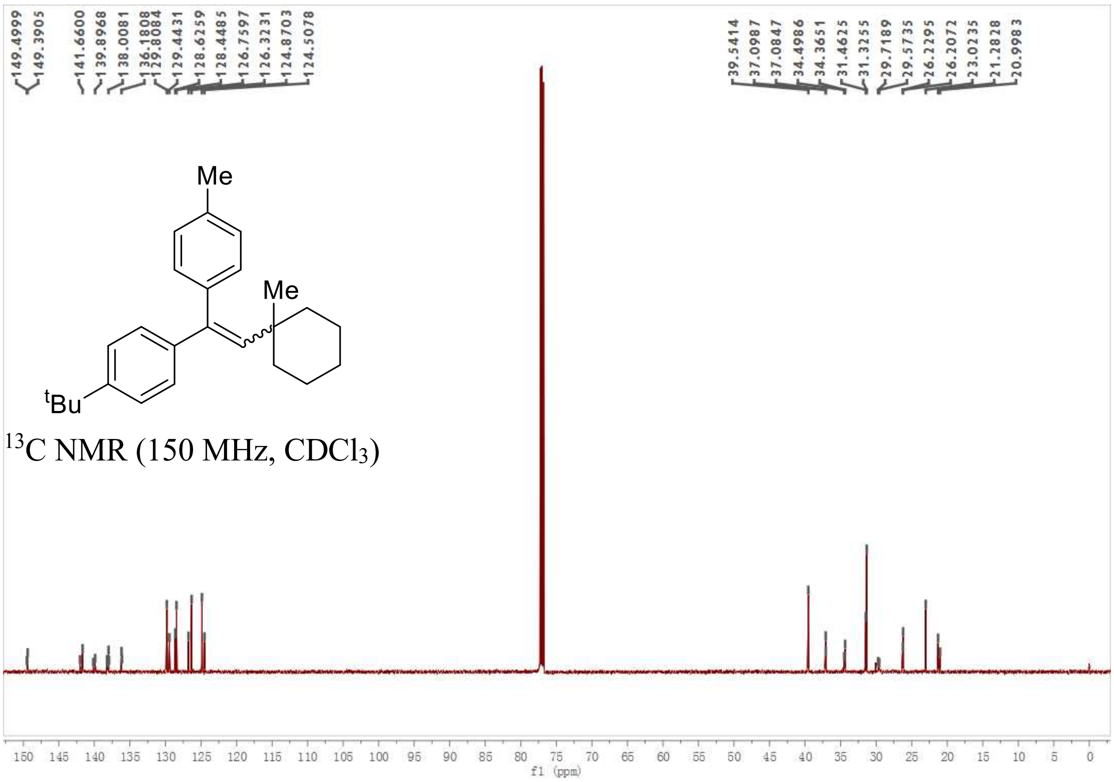

Supplementary Figure 61: ^{13}C NMR Spectra of 1-(tert-Butyl)-4-(2-(1-methylcyclohexyl)-1-(p-tolyl)vinyl)benzene (S23)

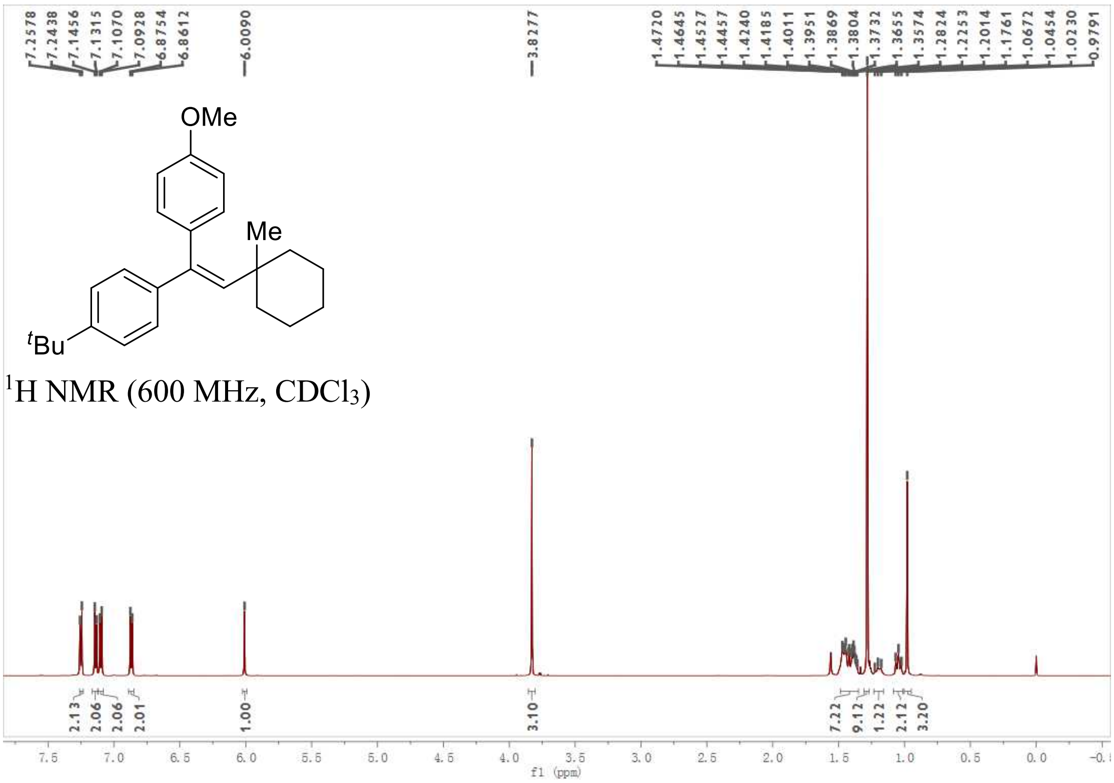

Supplementary Figure 62: ^{1}H NMR Spectra of (Z)-1-(tert-Butyl)-4-(1-(4-methoxyphenyl)-2-(1-methylcyclohexyl)vinyl)benzene (Z-S24)

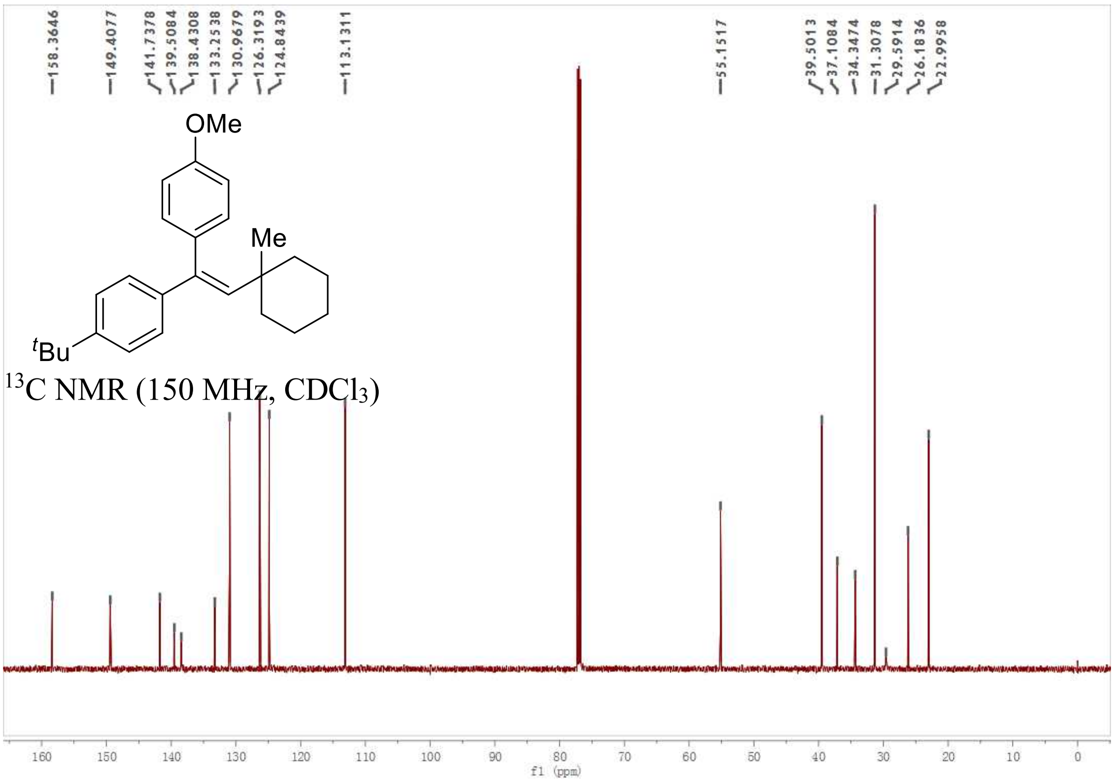

Supplementary Figure 63: ^{13}C NMR Spectra of (Z)-1-(tert-Butyl)-4-(1-(4-methoxyphenyl)-2-(1-methylcyclohexyl)vinyl)benzene (Z-S24)

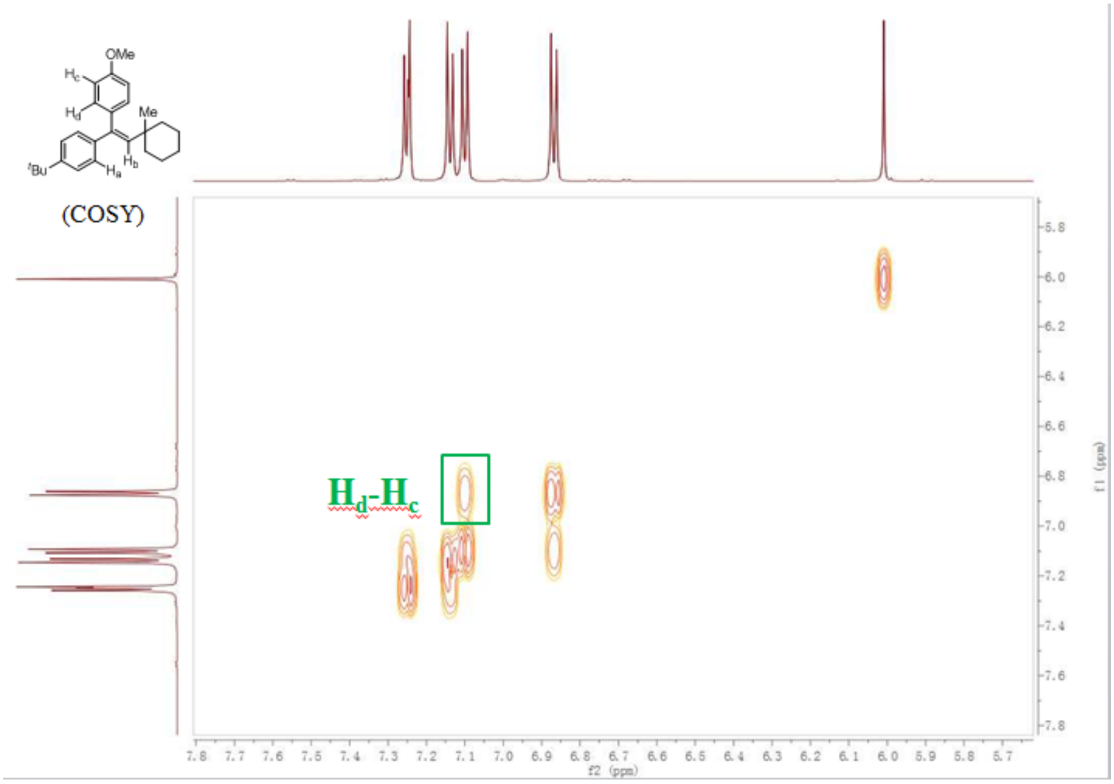

Supplementary Figure 64: COSY Spectra of (Z)-1-(tert-Butyl)-4-(1-(4-methoxyphenyl)-2-(1-methylcyclohexyl)vinyl)benzene (Z-S24)

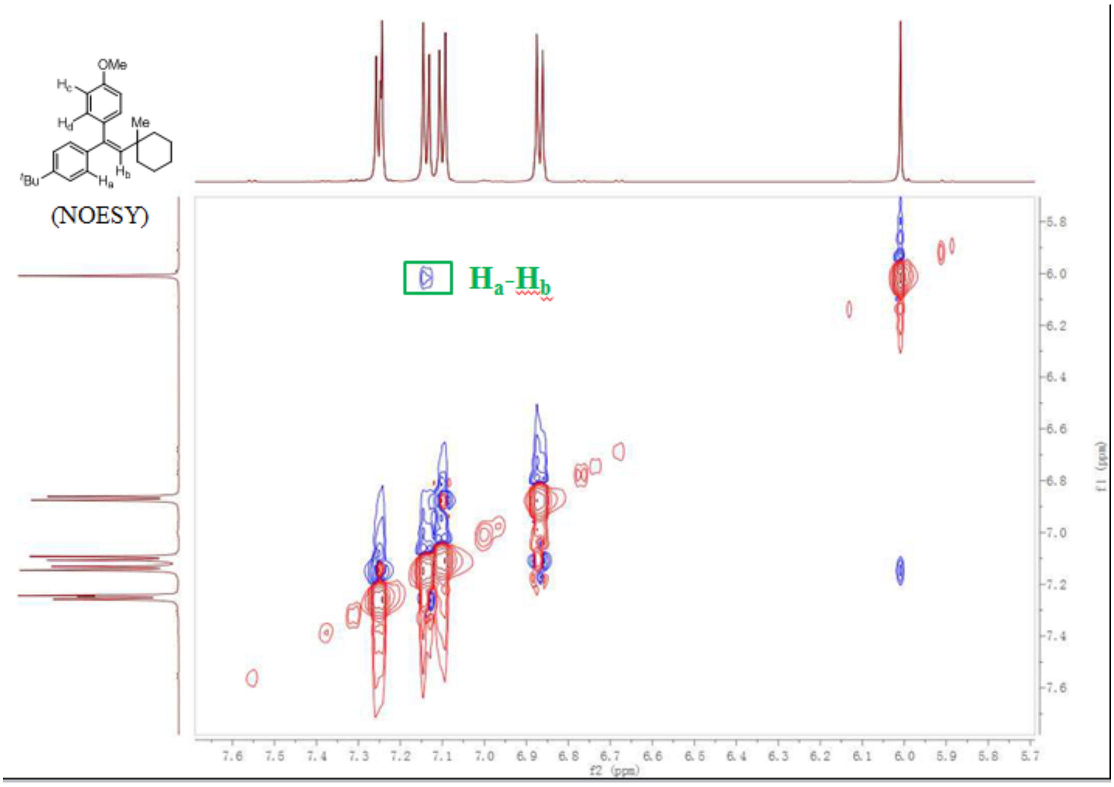

Supplementary Figure 65: NOESY Spectra of (Z)-1-(tert-Butyl)-4-(1-(4-methoxyphenyl)-2-(1-methylcyclohexyl)vinyl)benzene (Z-S24)

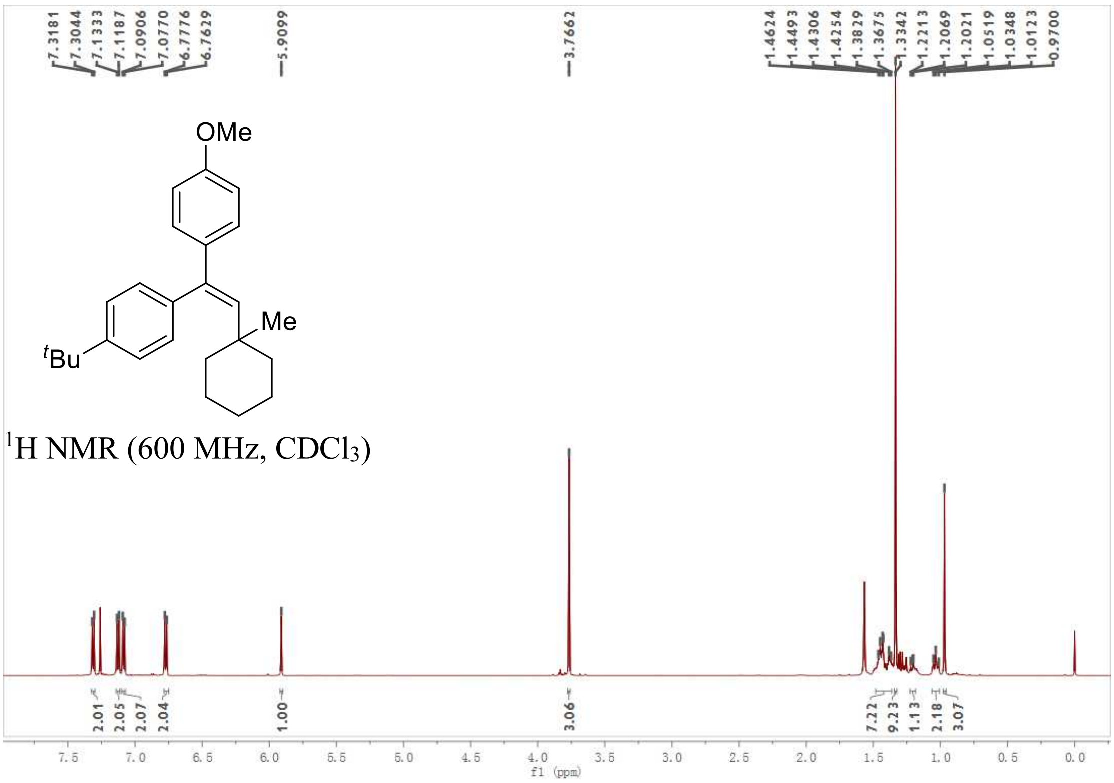

Supplementary Figure 66: ^{1}H NMR Spectra of (*E*)-1-(tert-Butyl)-4-(1-(4-methoxyphenyl)-2-(1-methylcyclohexyl)vinyl)benzene (*E*-S24)

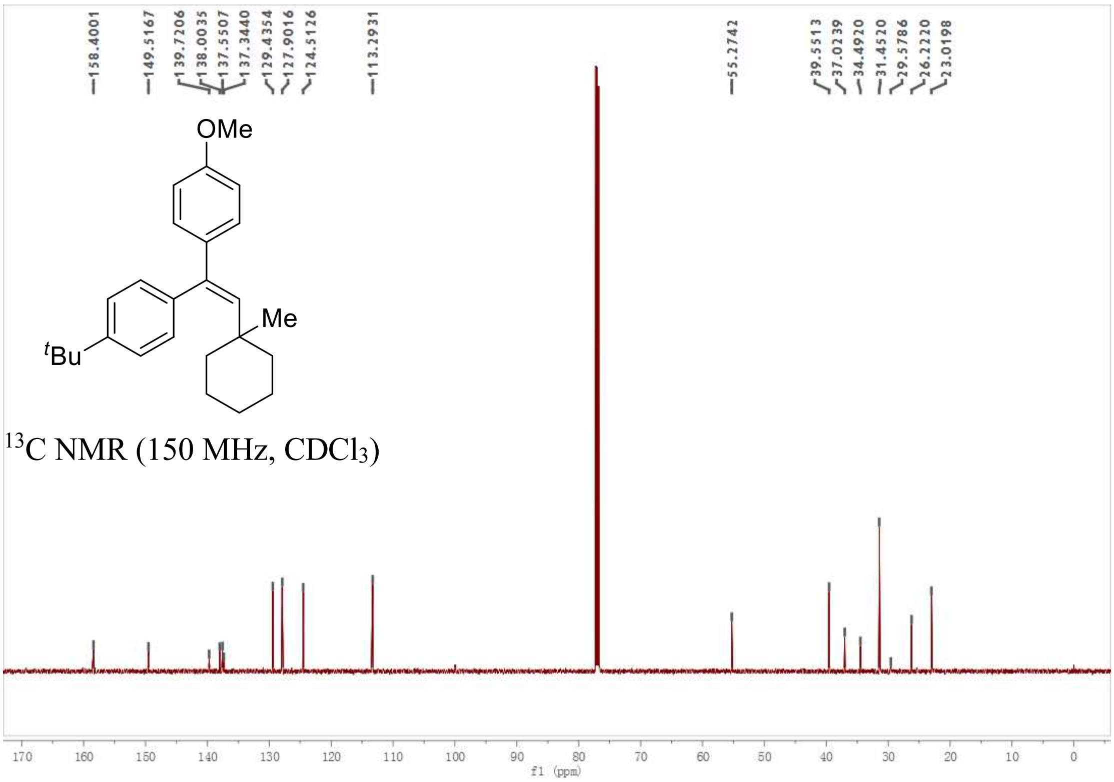

Supplementary Figure 67: ^{13}C NMR Spectra of (*E*)-1-(tert-Butyl)-4-(1-(4-methoxyphenyl)-2-(1-methylcyclohexyl)vinyl)benzene (*E*-S24)

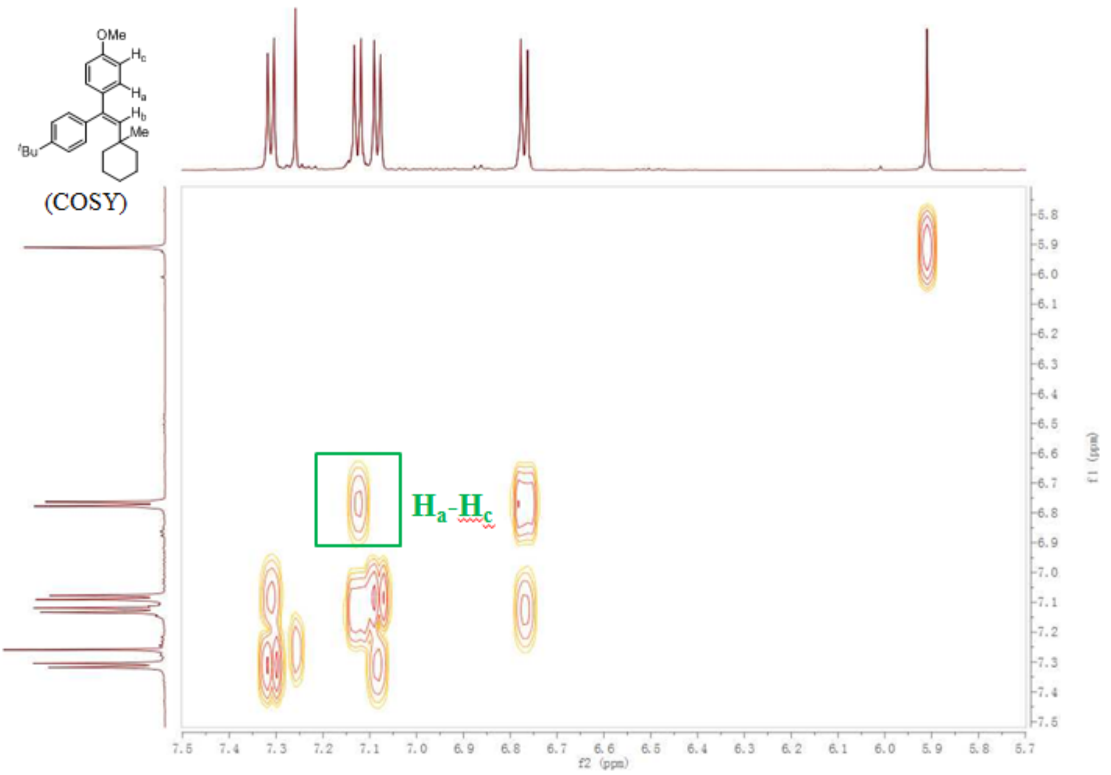

Supplementary Figure 68: COSY Spectra of (*E*)-1-(tert-Butyl)-4-(1-(4-methoxyphenyl)-2-(1-methylcyclohexyl)vinyl)benzene (*E*-S24)

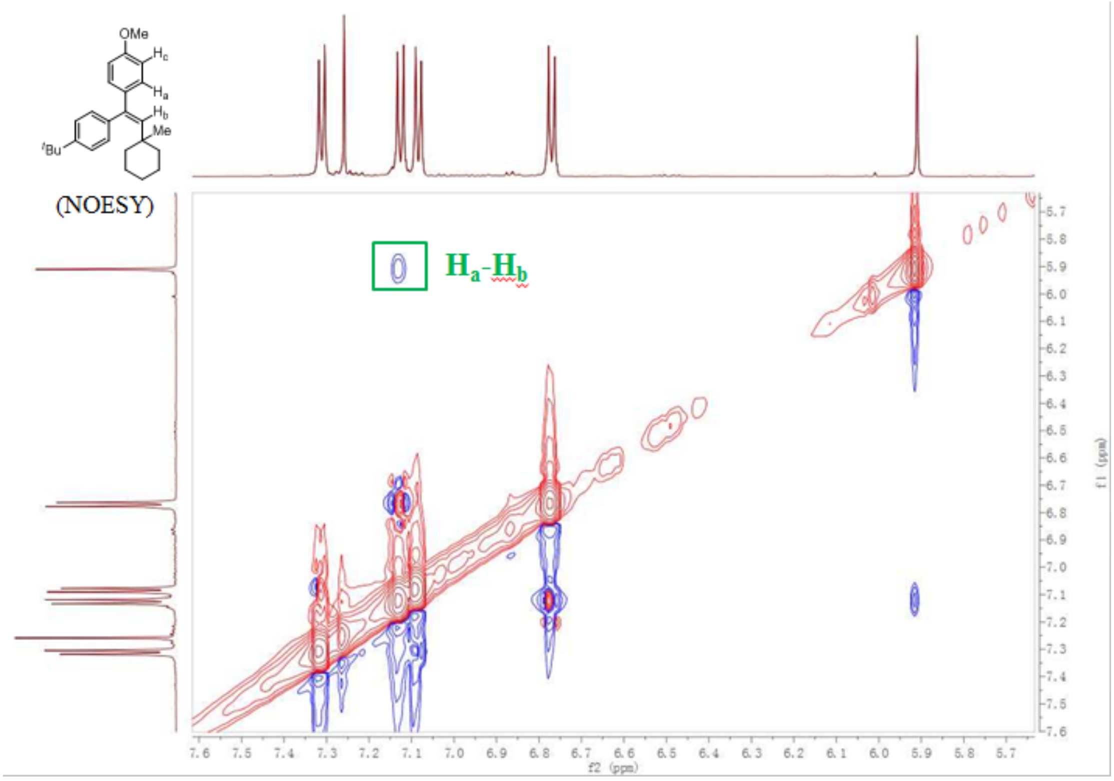

Supplementary Figure 69: NOESY Spectra of (*E*)-1-(tert-Butyl)-4-(1-(4-methoxyphenyl)-2-(1-methylcyclohexyl)vinyl)benzene (*E*-S24)

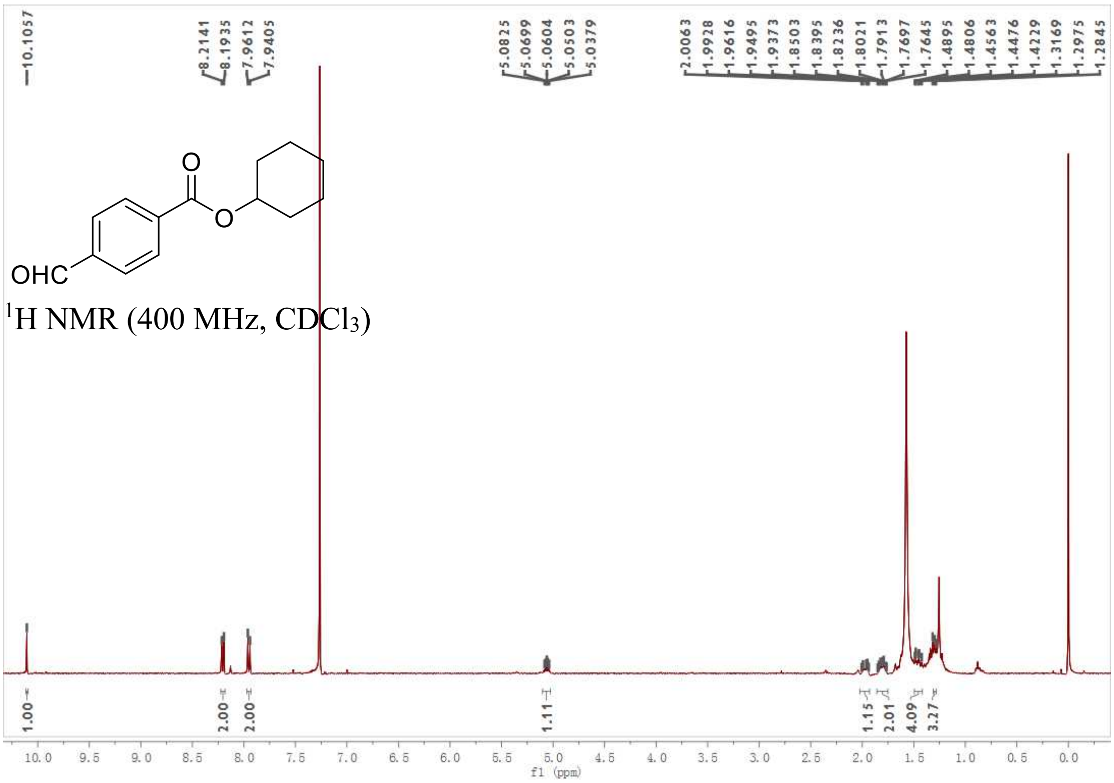

Supplementary Figure 70: ^{1}H NMR Spectra of Cyclohexyl 4-formylbenzoate (S25)

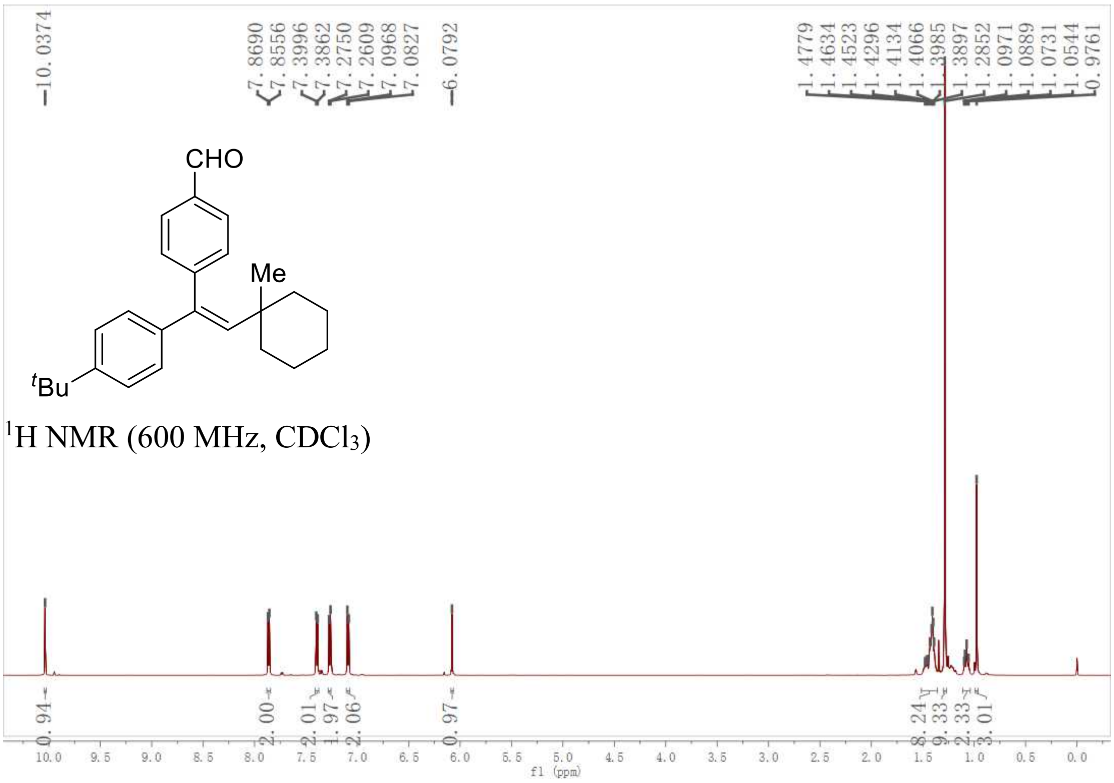

Supplementary Figure 71: ^{1}H NMR Spectra of (*Z*)-4-(1-(4-(tert-Butyl)phenyl)-2-(1-methylcyclohexyl)vinyl)benzaldehyde (19)

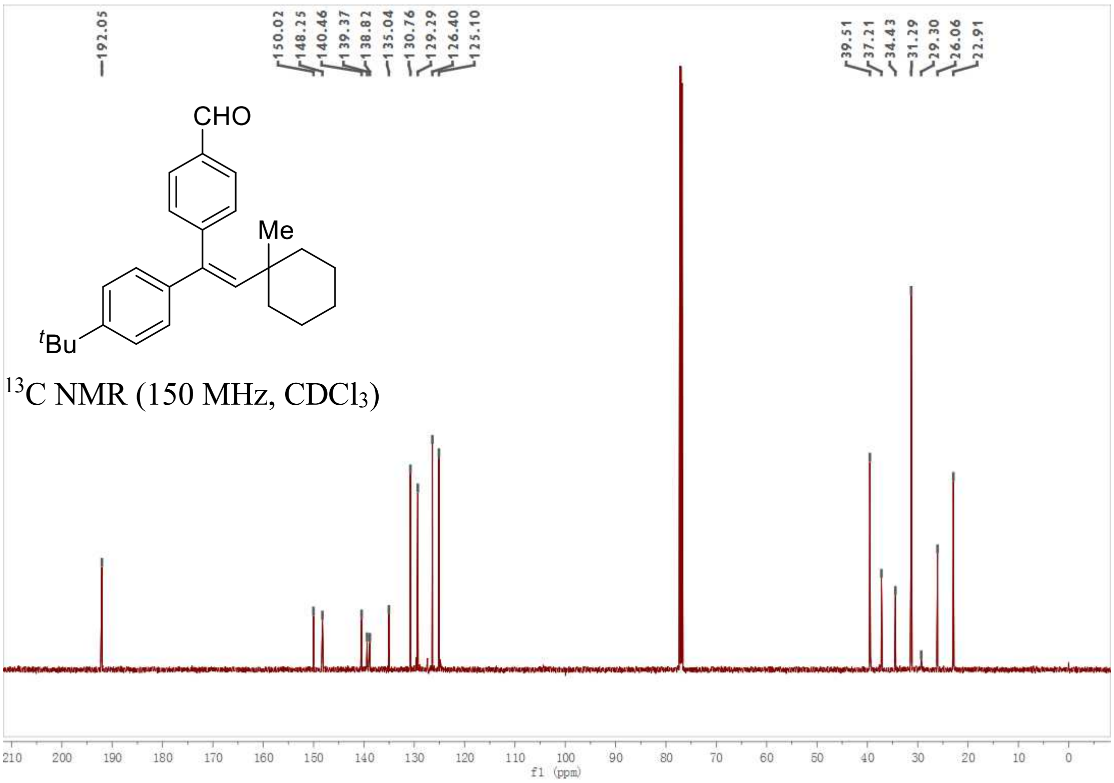

Supplementary Figure 72: ^{13}C NMR Spectra of (*Z*)-4-(1-(4-(tert-Butyl)phenyl)-2-(1-methylcyclohexyl)vinyl)benzaldehyde (19)

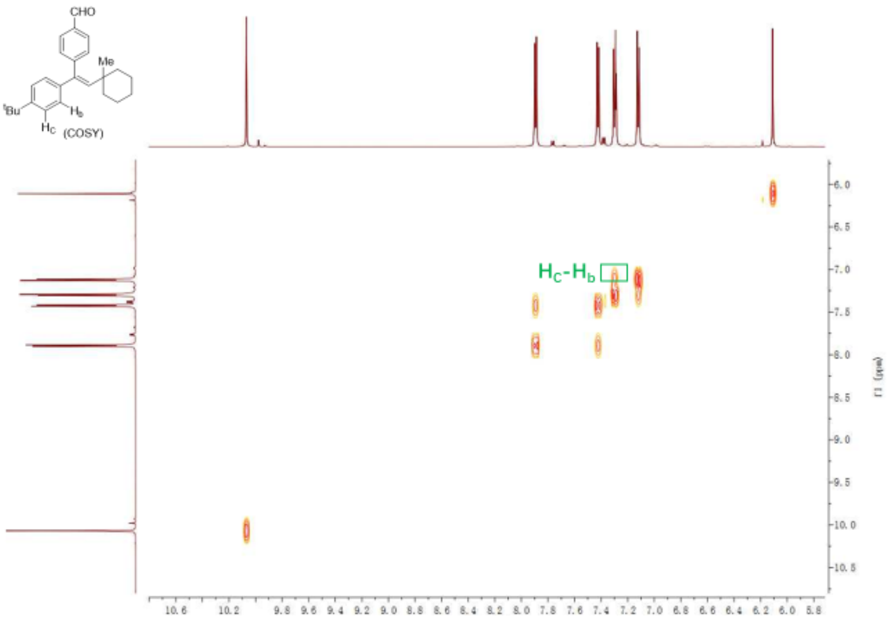

Supplementary Figure 73: COSY Spectra of (*Z*)-4-(1-(4-(tert-Butyl)phenyl) -2-(1-methylcyclohexyl)vinyl)benzaldehyde (19)

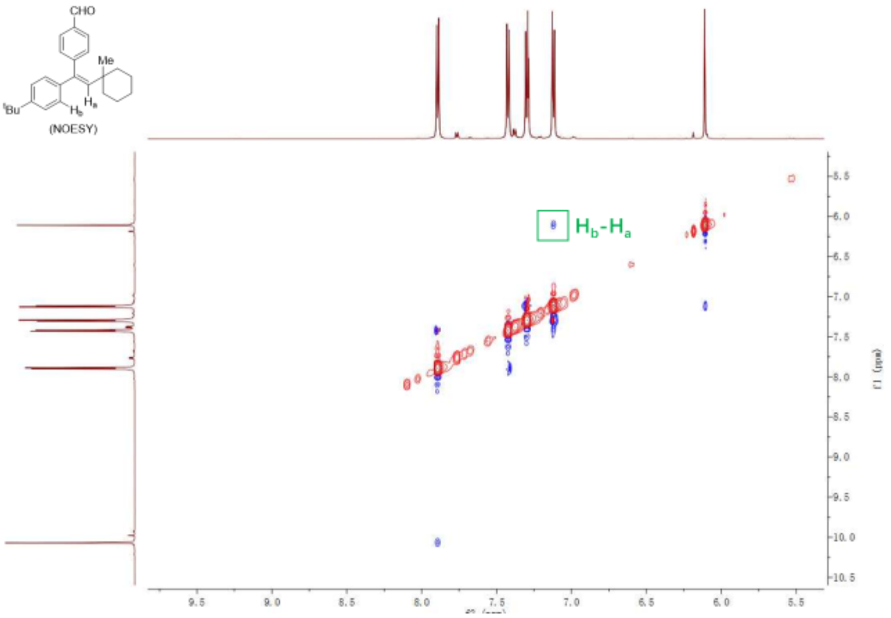

Supplementary Figure 74: NOESY Spectra of (*Z*)-4-(1-(4-(tert-Butyl)phenyl)-2 - (1-methylcyclohexyl)vinyl)benzaldehyde (19)

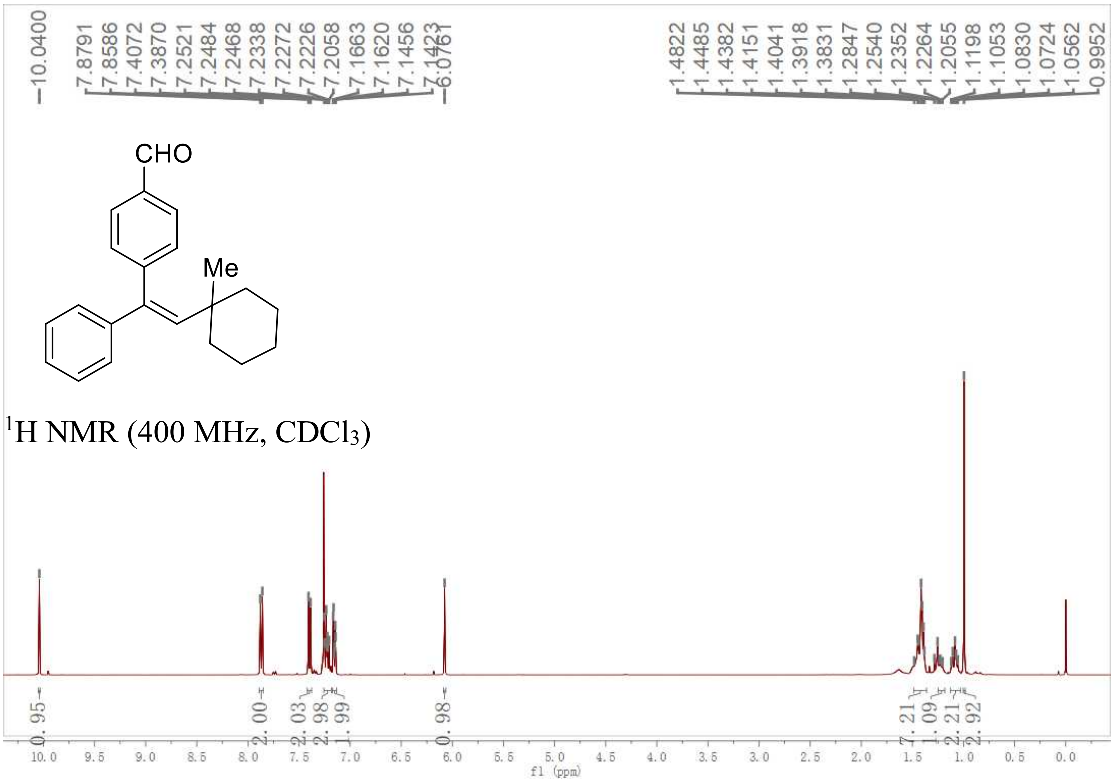

Supplementary Figure 75: ^{1}H NMR Spectra of (Z)-4-(2-(1-Methylcyclohexyl)-1-phenylvinyl)benzaldehyde (20)

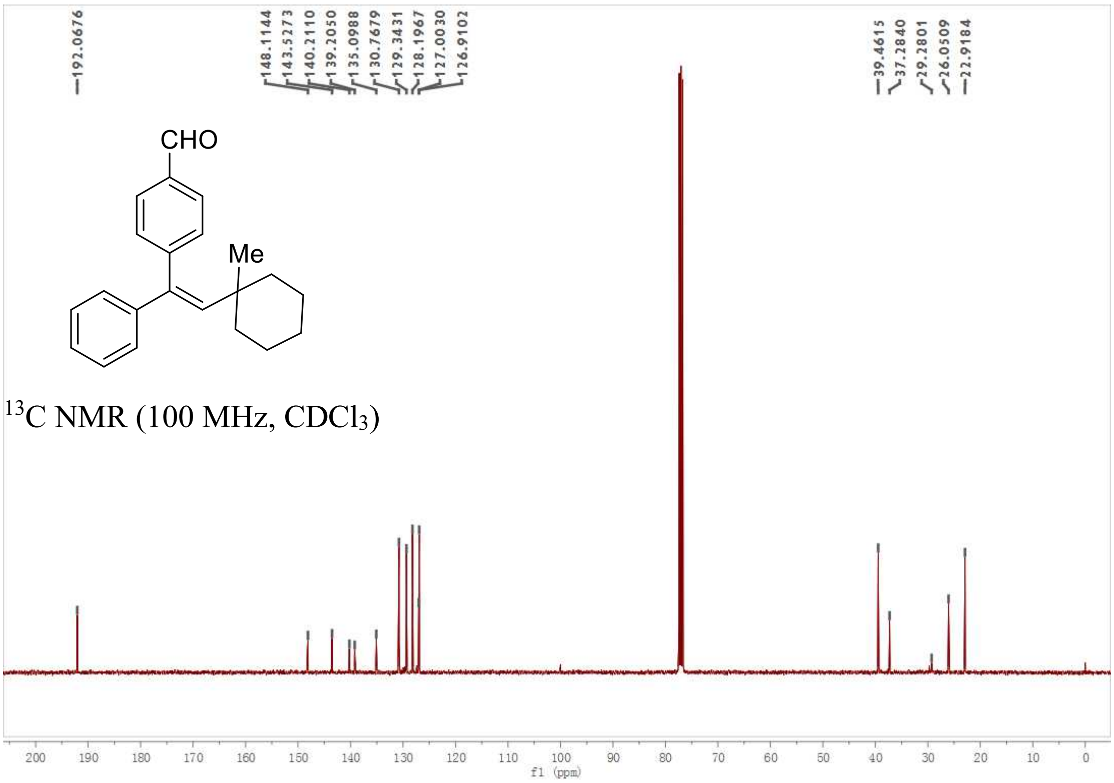

Supplementary Figure 76: ^{13}C NMR Spectra of (Z)-4-(2-(1-Methylcyclohexyl)-1-phenylvinyl)benzaldehyde (20)

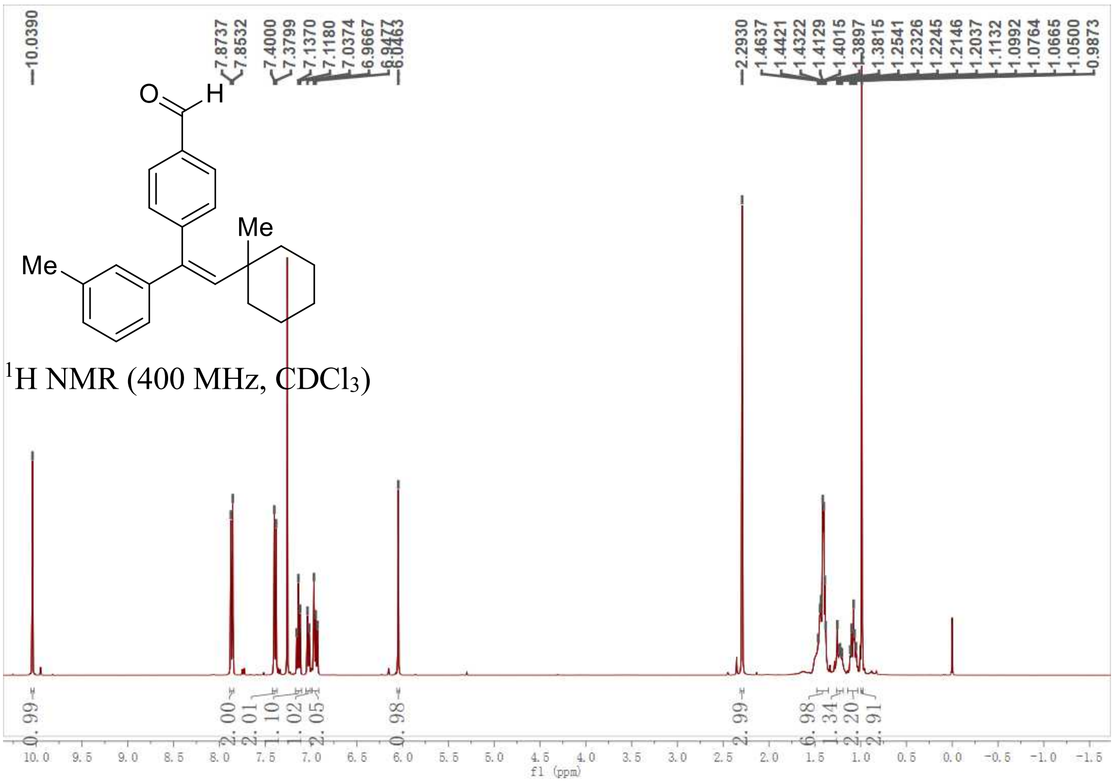

Supplementary Figure 77: ^{1}H NMR Spectra of (*E*)-4-(2-(1- Methyl cyclohexyl) -1-(m-tolyl)vinyl)benzaldehyde (21)

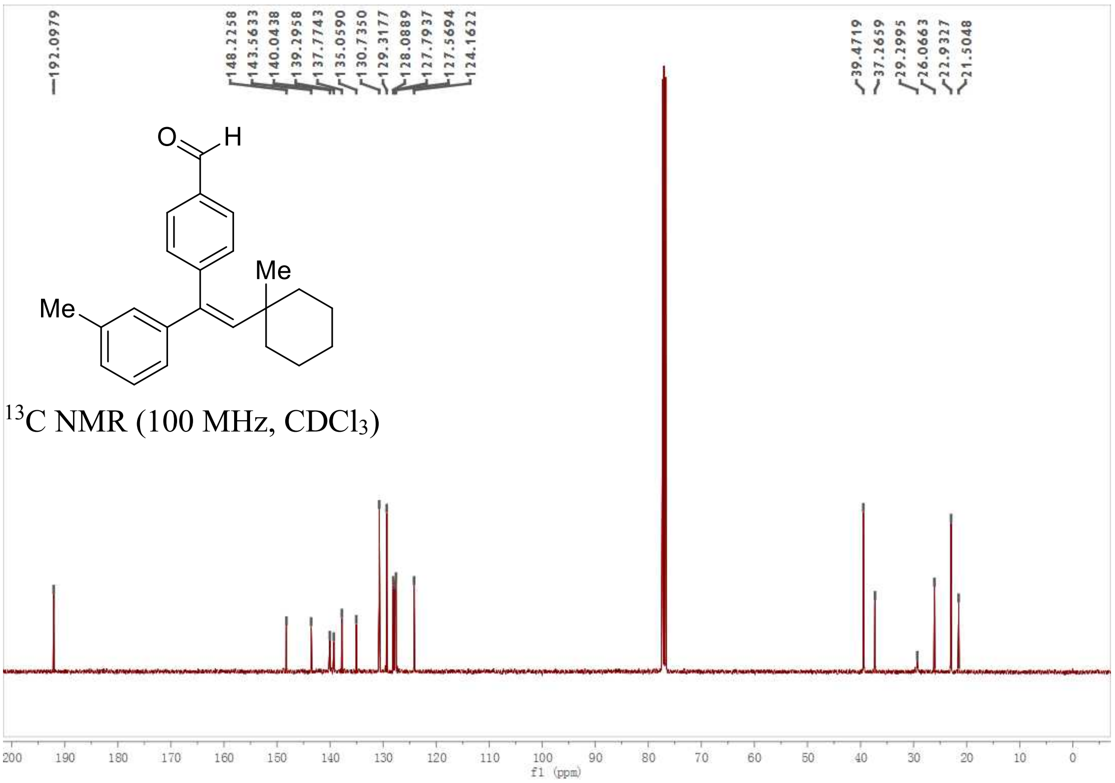

Supplementary Figure 78: ^{13}C NMR Spectra of (*E*)-4-(2-(1-Methylcyclohexyl) -1-(m-tolyl)vinyl)benzaldehyde (21)

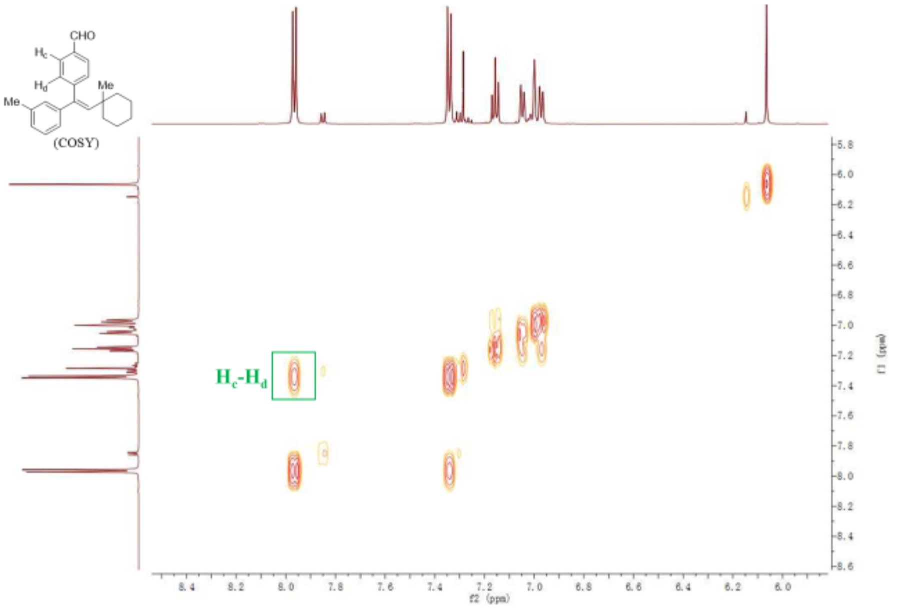

Supplementary Figure 79: COSY Spectra of (*E*)-4-(2-(1-Methylcyclohexyl) -1-(m-tolyl)vinyl)benzaldehyde (21)

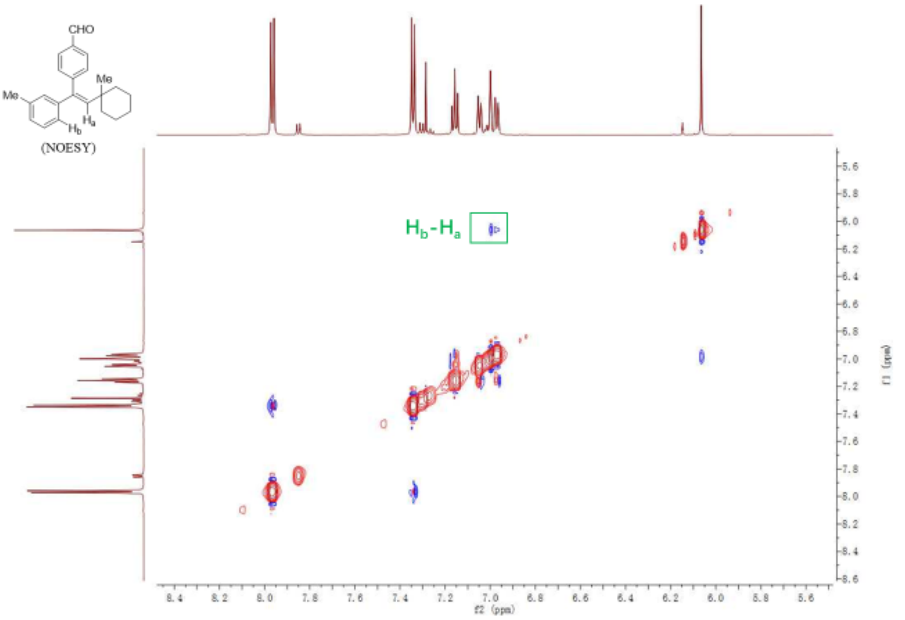

Supplementary Figure 80: NOESY Spectra of (*E*)-4-(2-(1-Methylcyclohexyl) -1-(m-tolyl)vinyl)benzaldehyde (21)

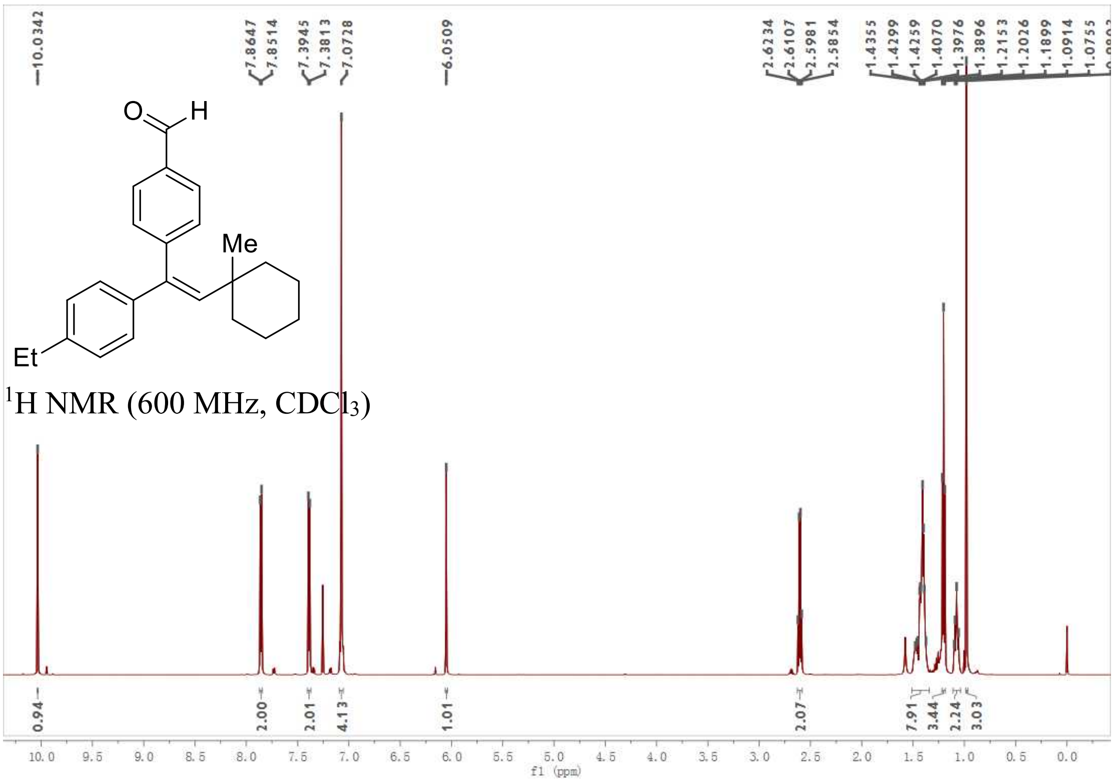

Supplementary Figure 81: ¹H NMR Spectra of (Z)-4-(1-(4-Ethylphenyl) -2-(1-methylcyclohexyl)vinyl)benzaldehyde (22)

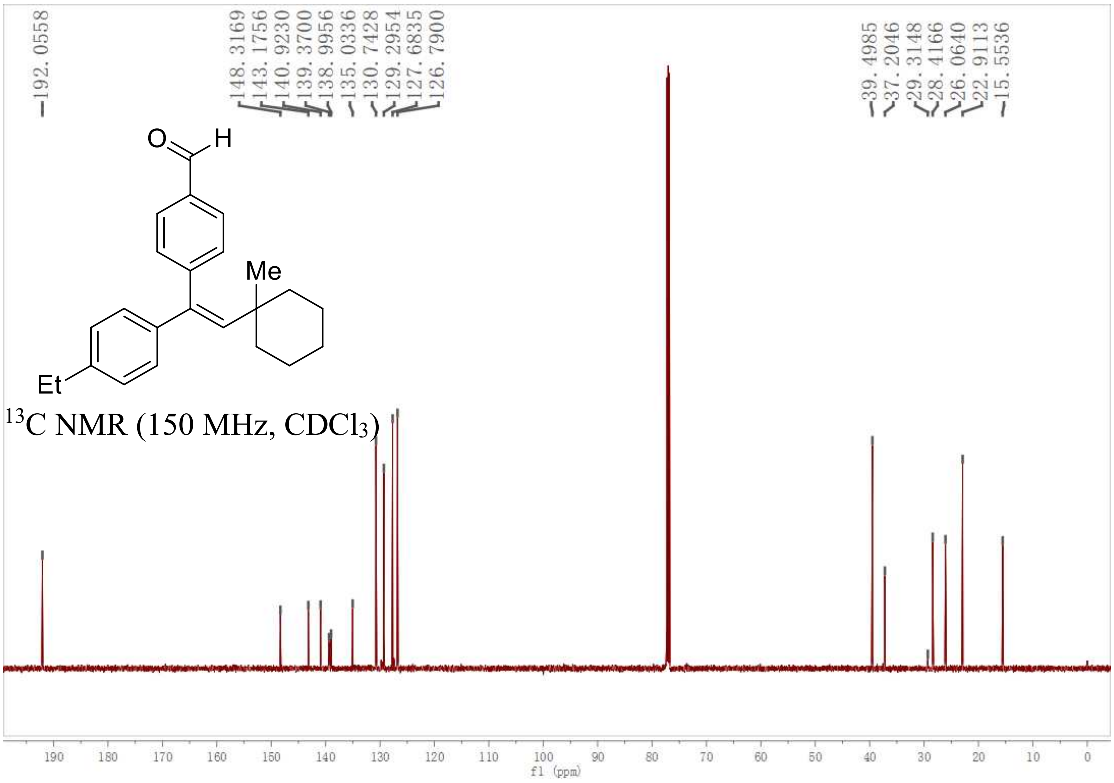

Supplementary Figure 82: ^{13}C NMR Spectra of (Z)-4-(1-(4-Ethylphenyl)-2-(1-methylcyclohexyl)vinyl)benzaldehyde (22)

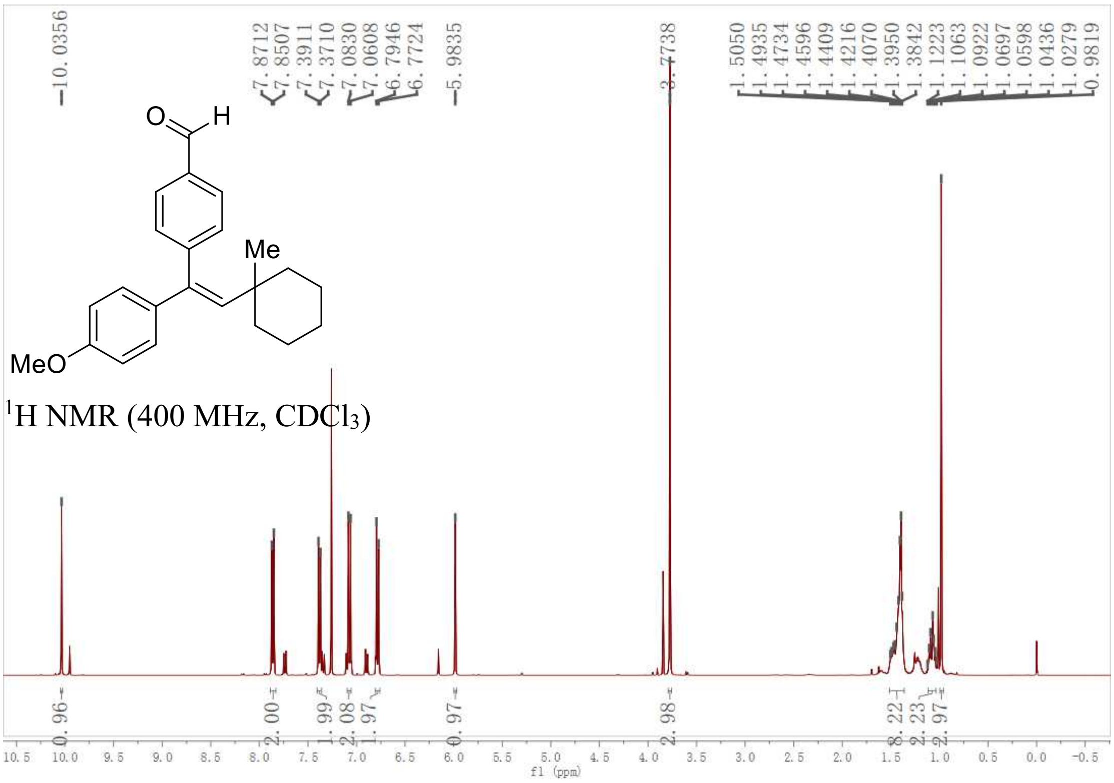

Supplementary Figure 83: ^{1}H NMR Spectra of (*E*)-4-(1-(4-Methoxyphenyl)-2-(1-methylcyclohexyl)vinyl)benzaldehyde (23)

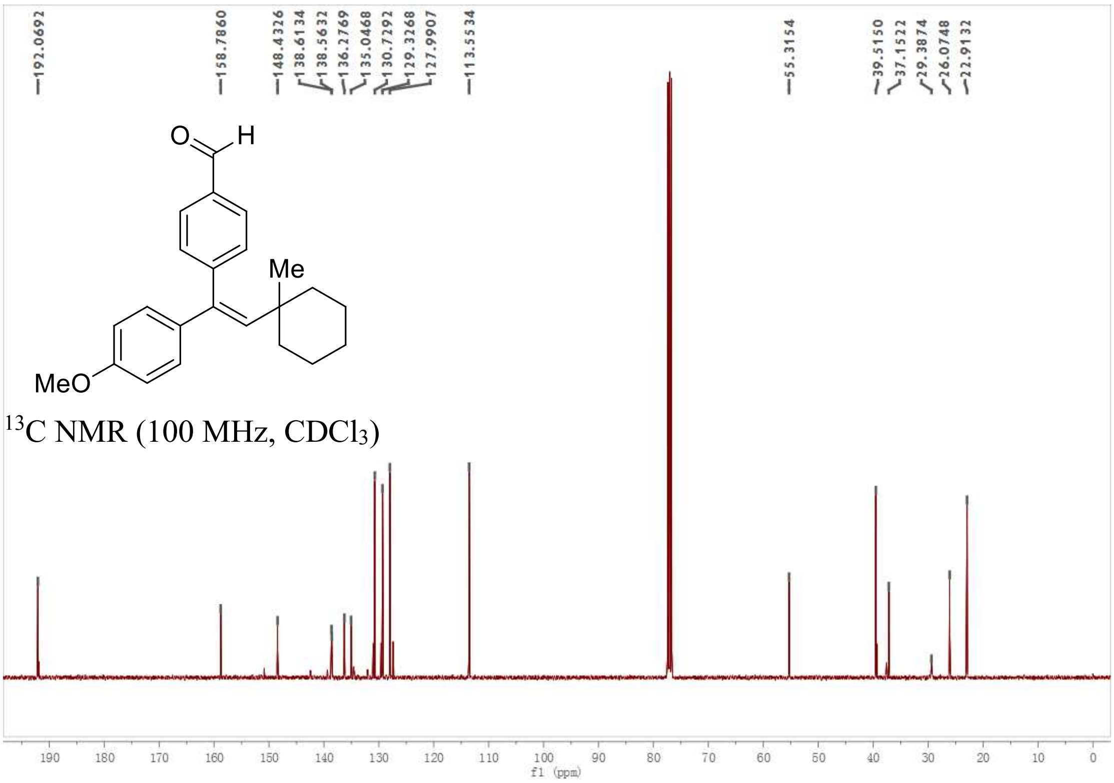

Supplementary Figure 84: ^{13}C NMR Spectra of (*E*)-4-(1-(4-Methoxyphenyl) -2-(1-methylcyclohexyl)vinyl)benzaldehyde (23)

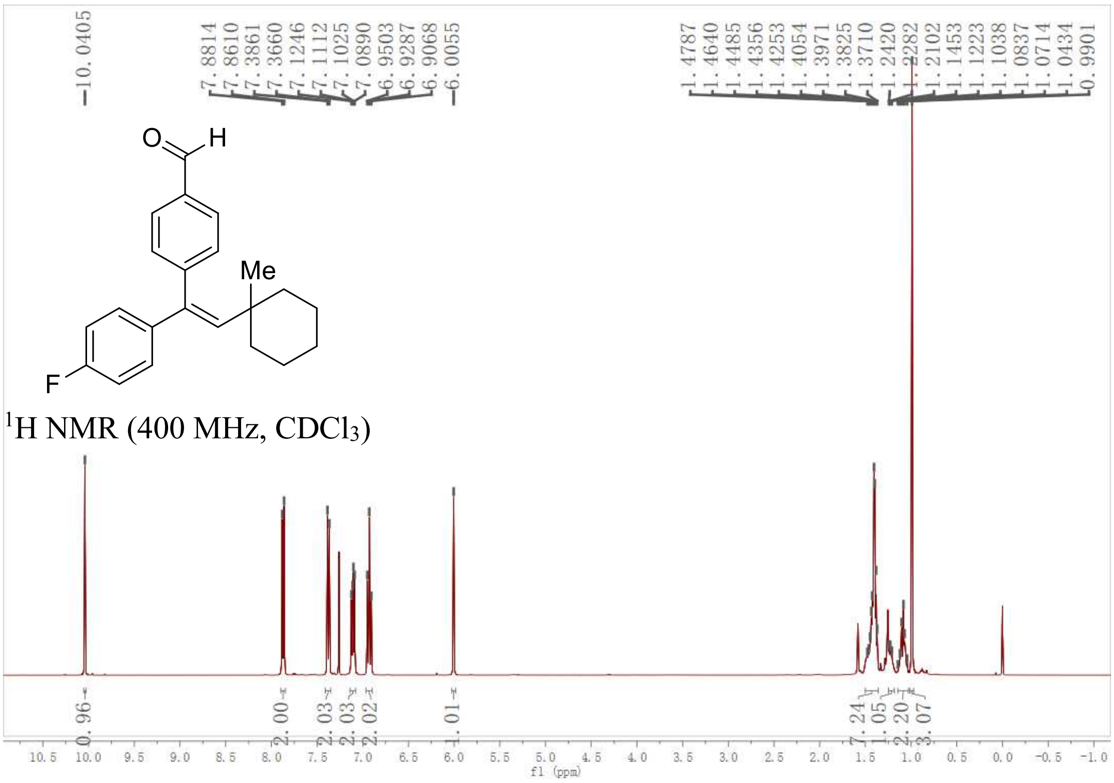

Supplementary Figure 85: ^{1}H NMR Spectra of (*E*)-4-(1-(4-Fluorophenyl) -2-(1-methylcyclohexyl)vinyl)benzaldehyde (24)

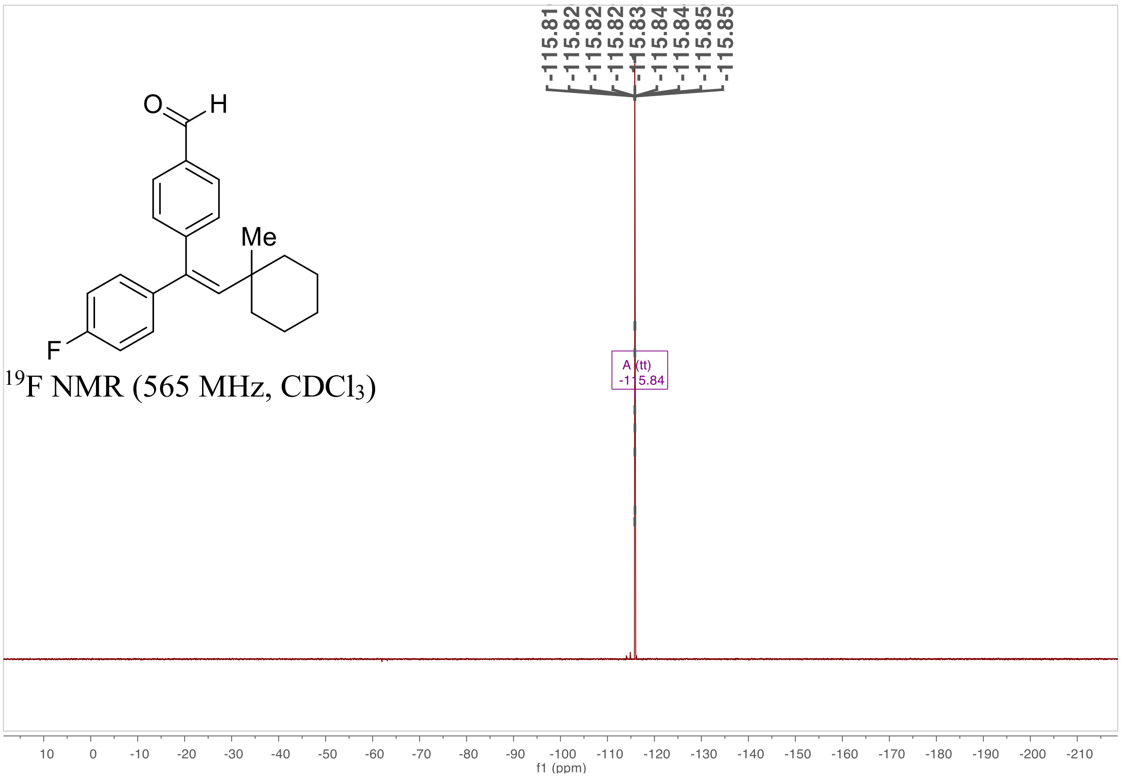

Supplementary Figure 86: ^{19}F NMR Spectra of (*E*)-4-(1-(4-Fluorophenyl)-2-(1-methylcyclohexyl)vinyl)benzaldehyde (24)

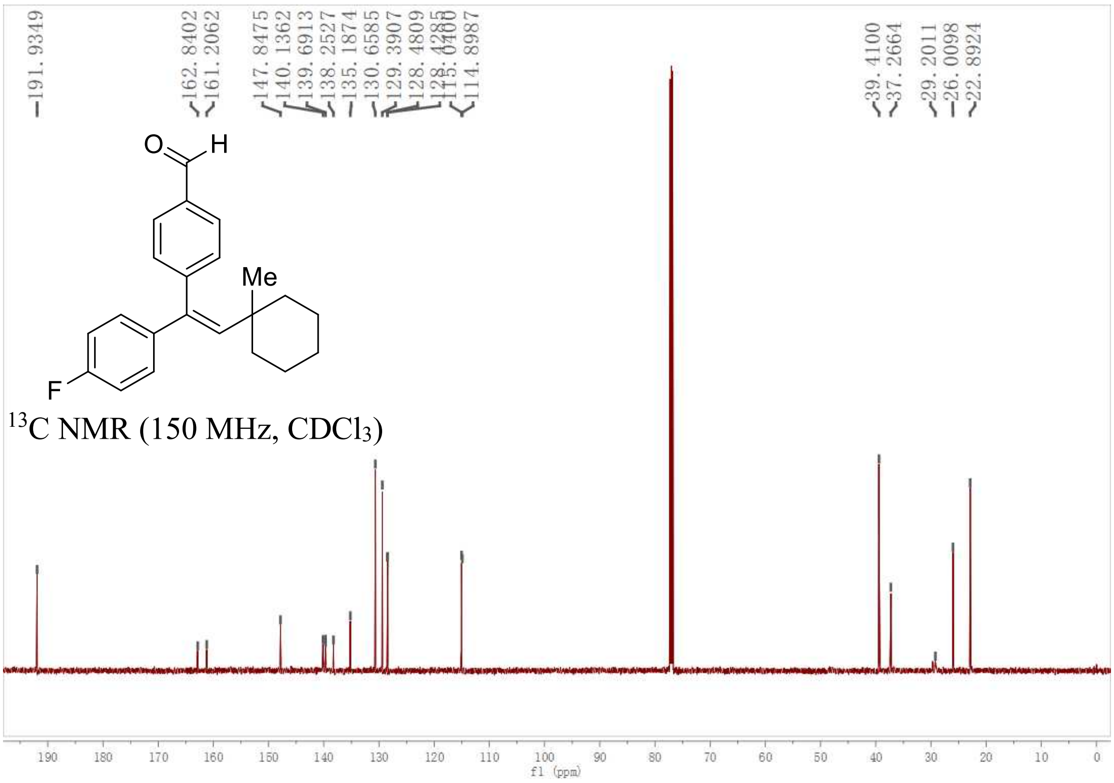

Supplementary Figure 87: ^{13}C NMR Spectra of (*E*)-4-(1-(4-Fluorophenyl)-2-(1-methylcyclohexyl)vinyl)benzaldehyde (24)

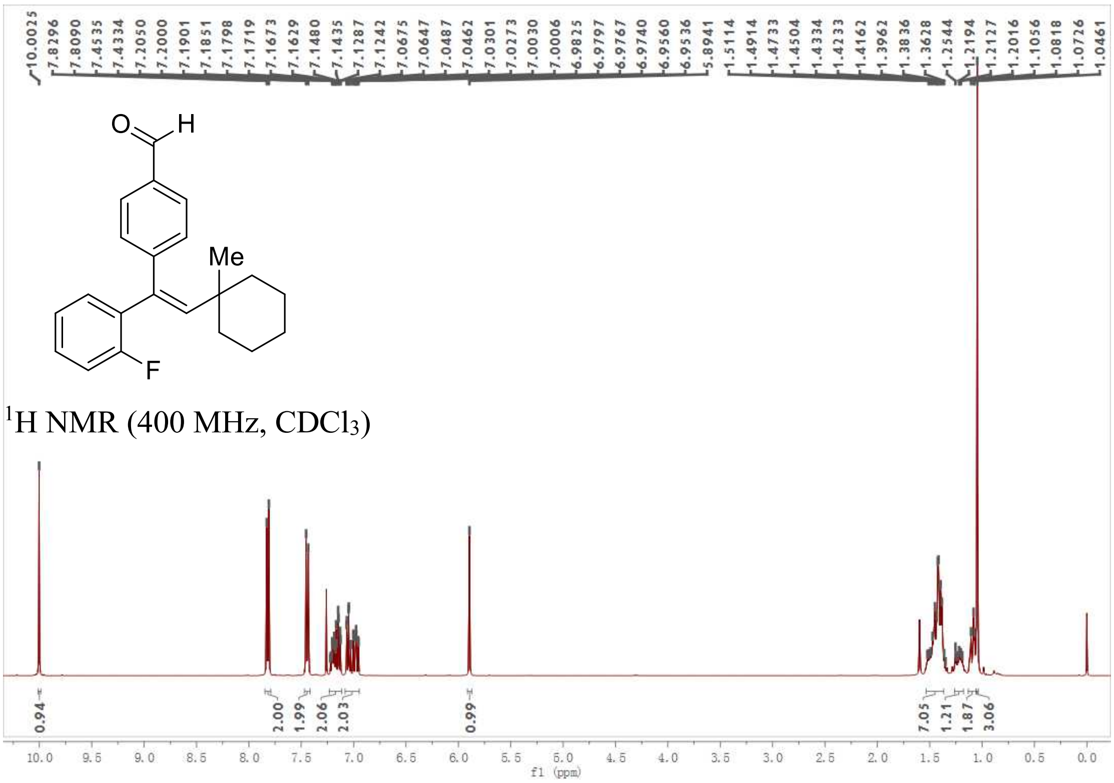

Supplementary Figure 88: ^{1}H NMR Spectra of (*E*)-4-(1-(2-Fluorophenyl) -2-(1-methylcyclohexyl)vinyl)benzaldehyde (25)

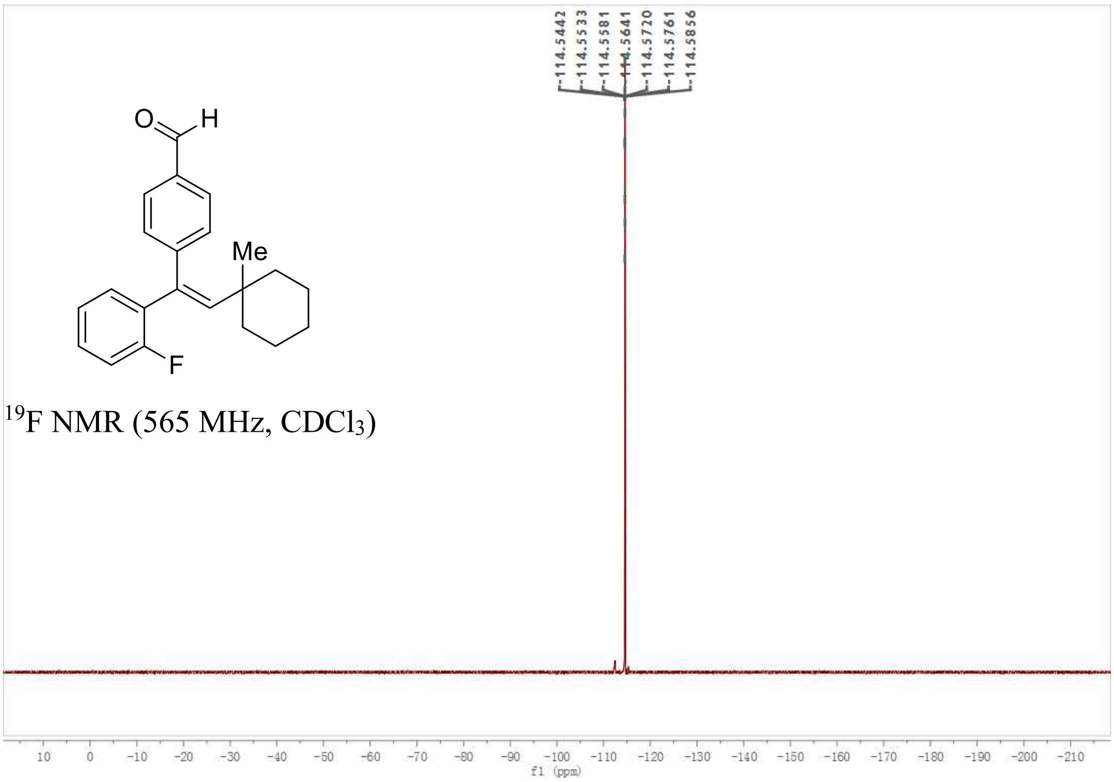

Supplementary Figure 89: ^{19}F NMR Spectra of (*E*)-4-(1-(2-Fluorophenyl) -2-(1-methylcyclohexyl)vinyl)benzaldehyde (25)

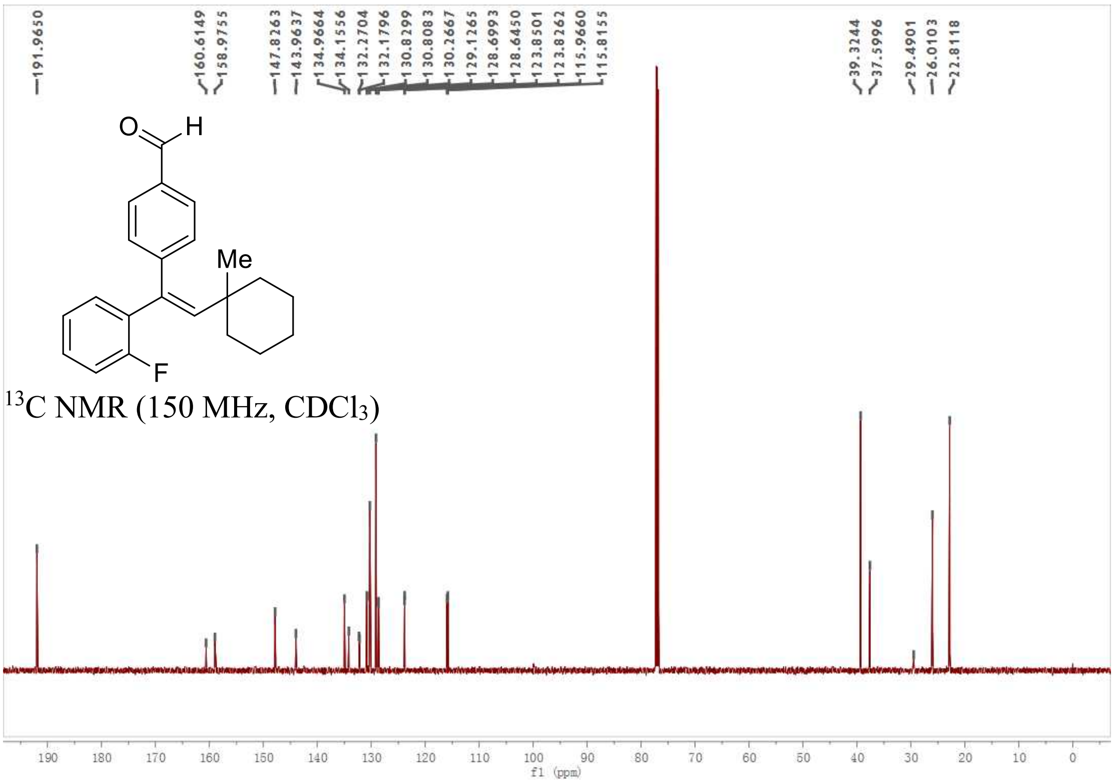

Supplementary Figure 90: ^{13}C NMR Spectra of (*E*)-4-(1-(2-Fluorophenyl) -2-(1-methylcyclohexyl)vinyl)benzaldehyde (25)

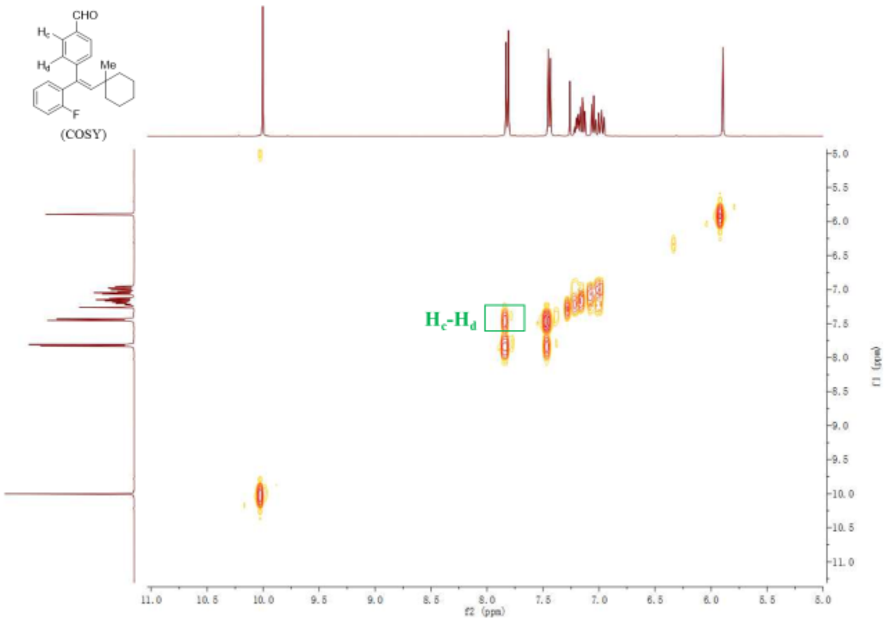

Supplementary Figure 91: COSY Spectra of (*E*)-4-(1-(2-Fluorophenyl) -2-(1-methylcyclohexyl)vinyl)benzaldehyde (25)

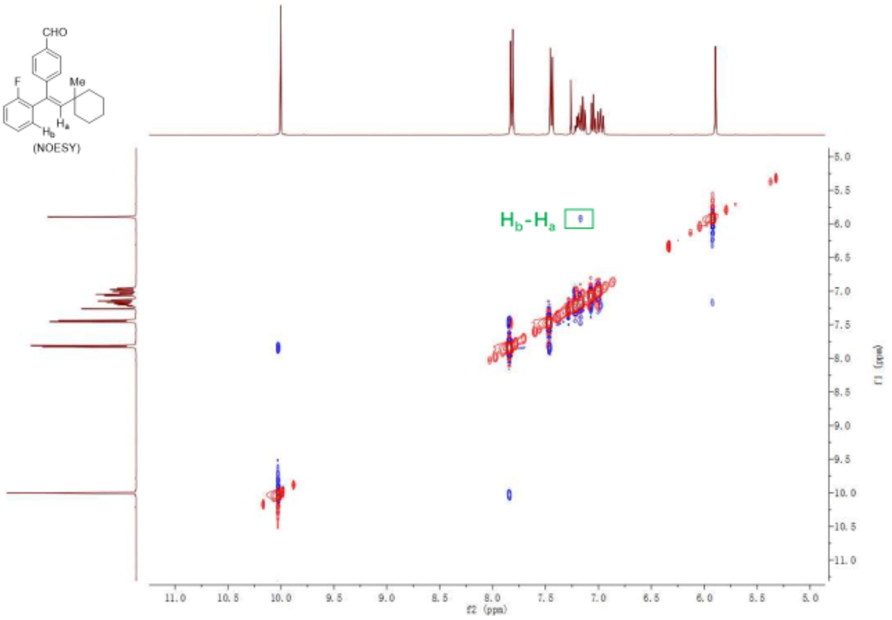

Supplementary Figure 92: NOESY Spectra of (*E*)-4-(1-(2-Fluorophenyl) -2-(1-methylcyclohexyl)vinyl)benzaldehyde (25)

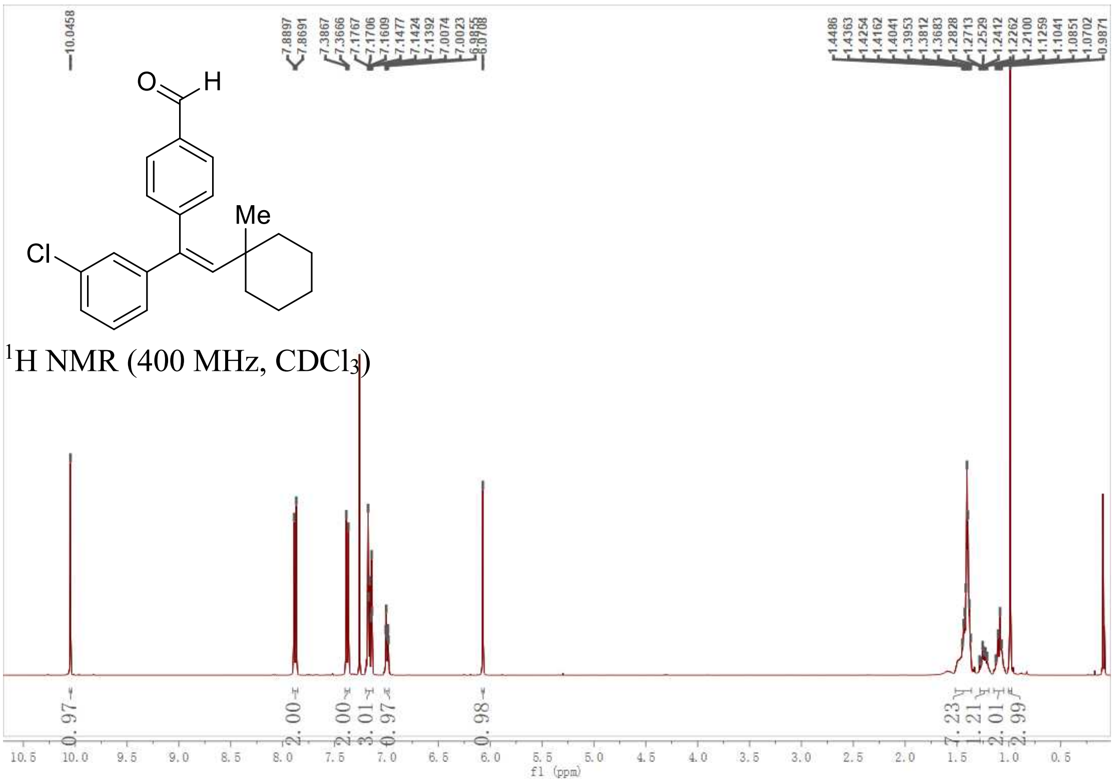

Supplementary Figure 93: ^{1}H NMR Spectra of (*E*)-4-(1-(3-Chlorophenyl) -2-(1-methylcyclohexyl)vinyl)benzaldehyde (26)

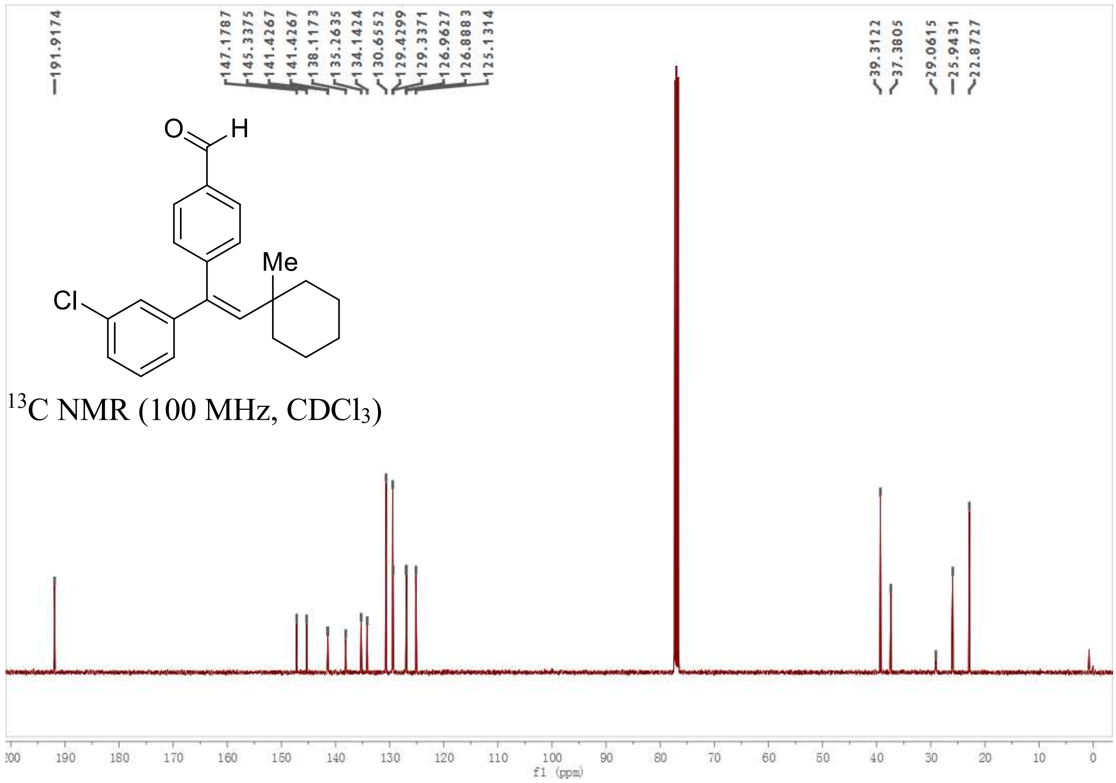

Supplementary Figure 94: ^{13}C NMR Spectra of (*E*)-4-(1-(3-Chlorophenyl) -2-(1-methylcyclohexyl)vinyl)benzaldehyde (26)

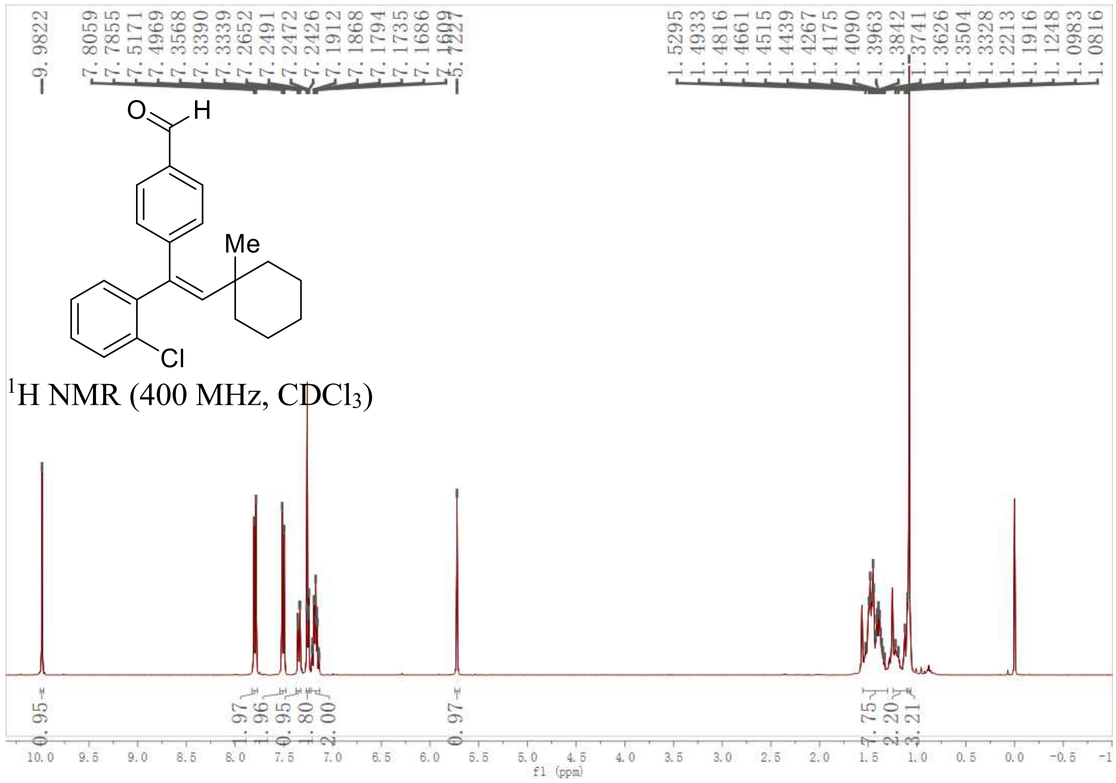

Supplementary Figure 95: ^{1}H NMR Spectra of (*E*)-4-(1-(2-Chlorophenyl) -2-(1-methylcyclohexyl)vinyl)benzaldehyde (27)

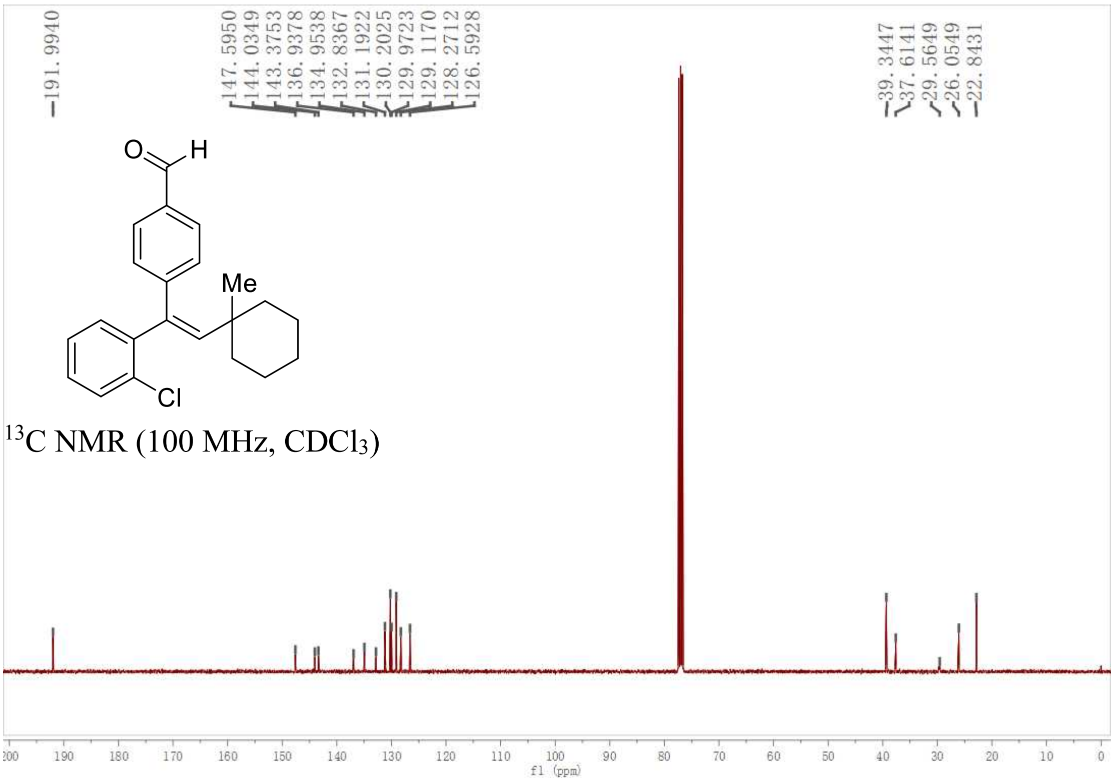

Supplementary Figure 96: ^{13}C NMR Spectra of (*E*)-4-(1-(2-Chlorophenyl) -2-(1-methylcyclohexyl)vinyl)benzaldehyde (27)

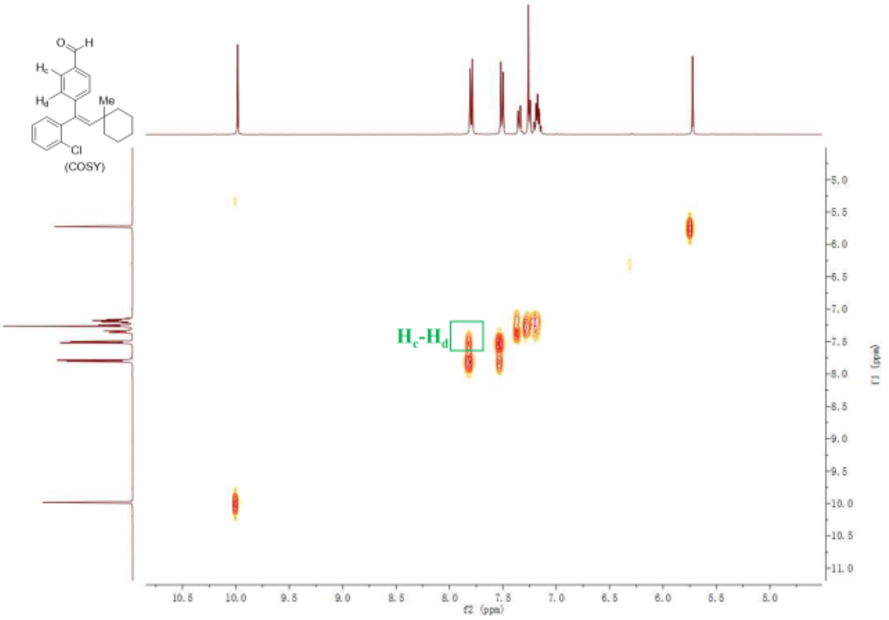

Supplementary Figure 97: COSY Spectra of (*E*)-4-(1-(2-Chlorophenyl) -2-(1-methylcyclohexyl)vinyl)benzaldehyde (27)

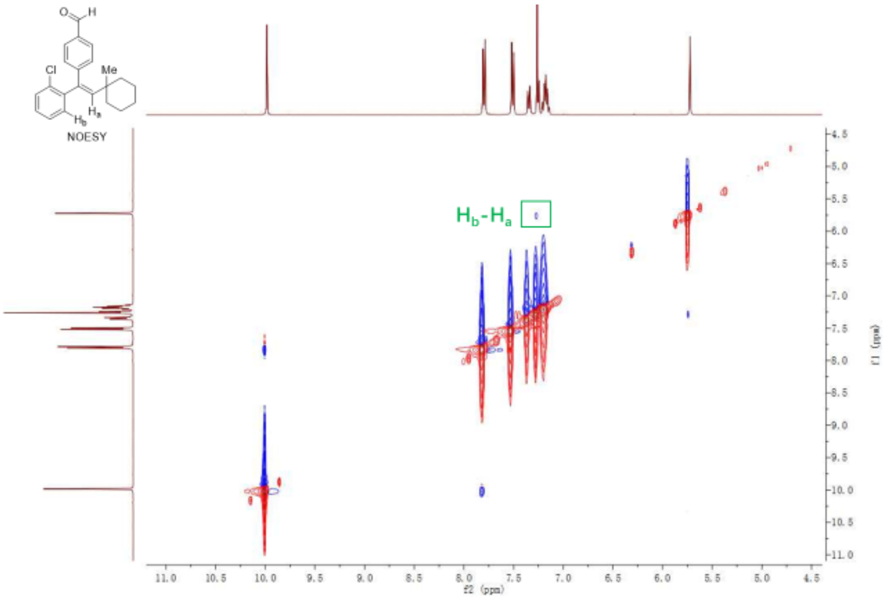

Supplementary Figure 98: NOESY Spectra of (*E*)-4-(1-(2-Chlorophenyl) -2-(1-methylcyclohexyl)vinyl)benzaldehyde (27)

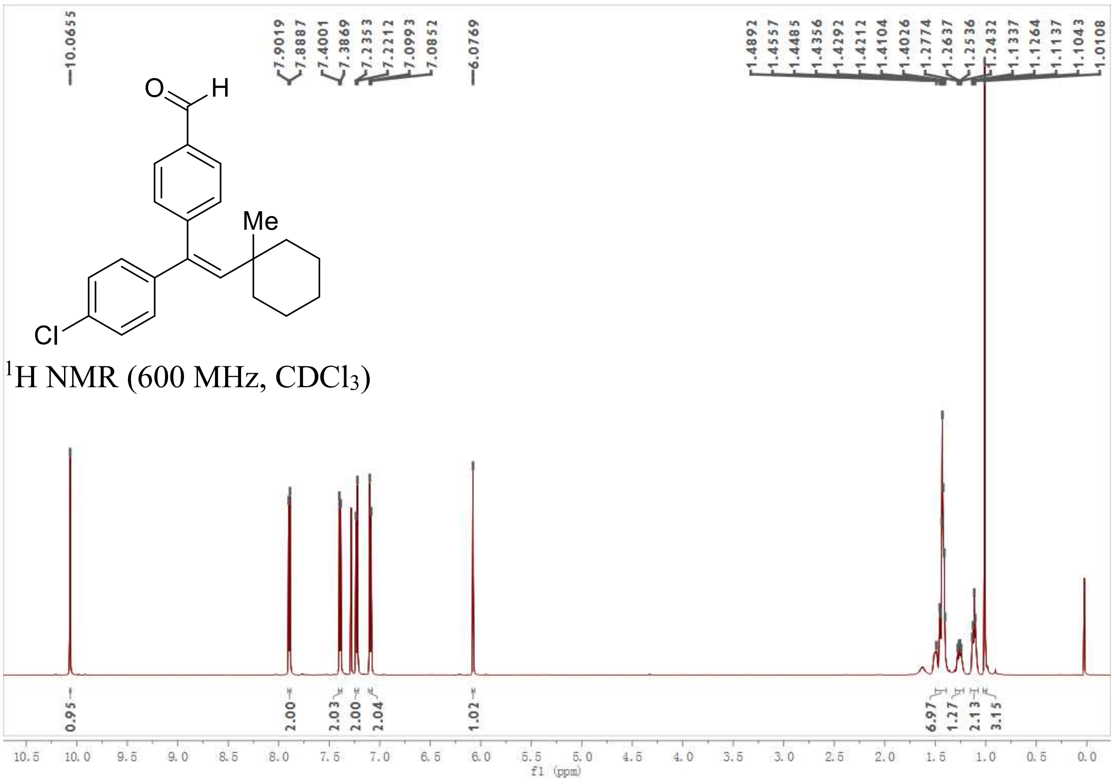

Supplementary Figure 99: ^{1}H NMR Spectra of (*E*)-4-(1-(4-chlorophenyl) -2-(1-methylcyclohexyl)vinyl)benzaldehyde (28)

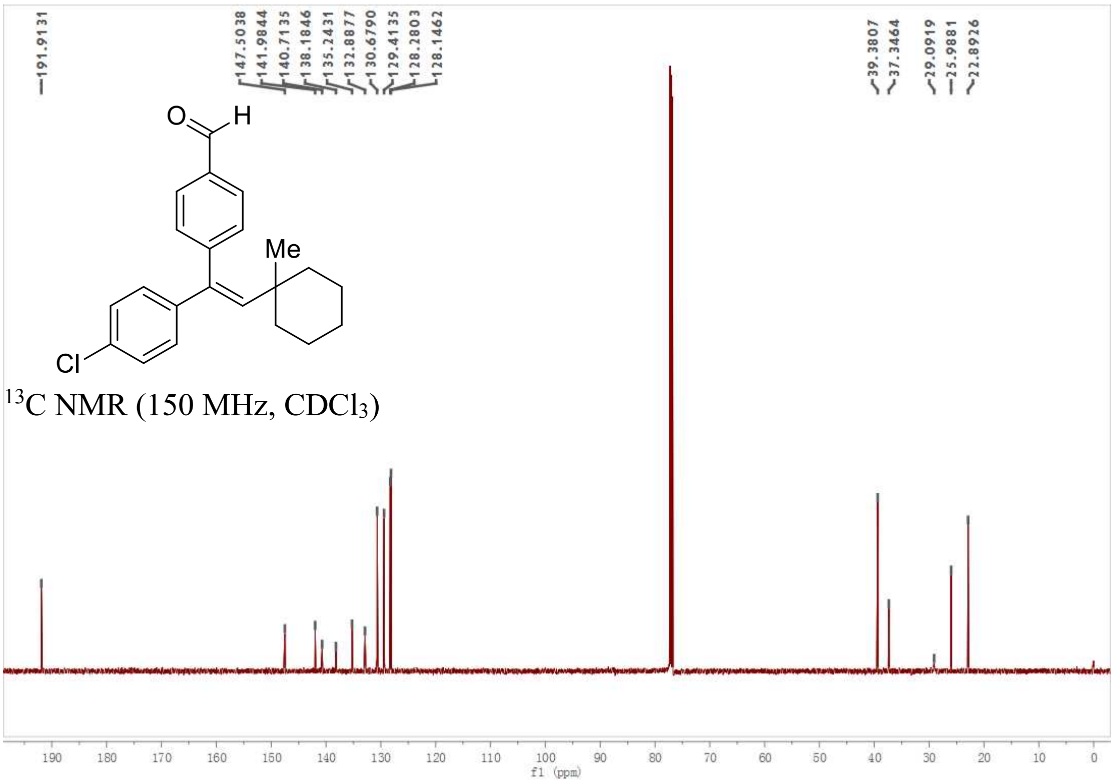

Supplementary Figure 100: ^{13}C NMR Spectra of (*E*)-4-(1-(4-chlorophenyl) -2-(1-methylcyclohexyl)vinyl)benzaldehyde (28)

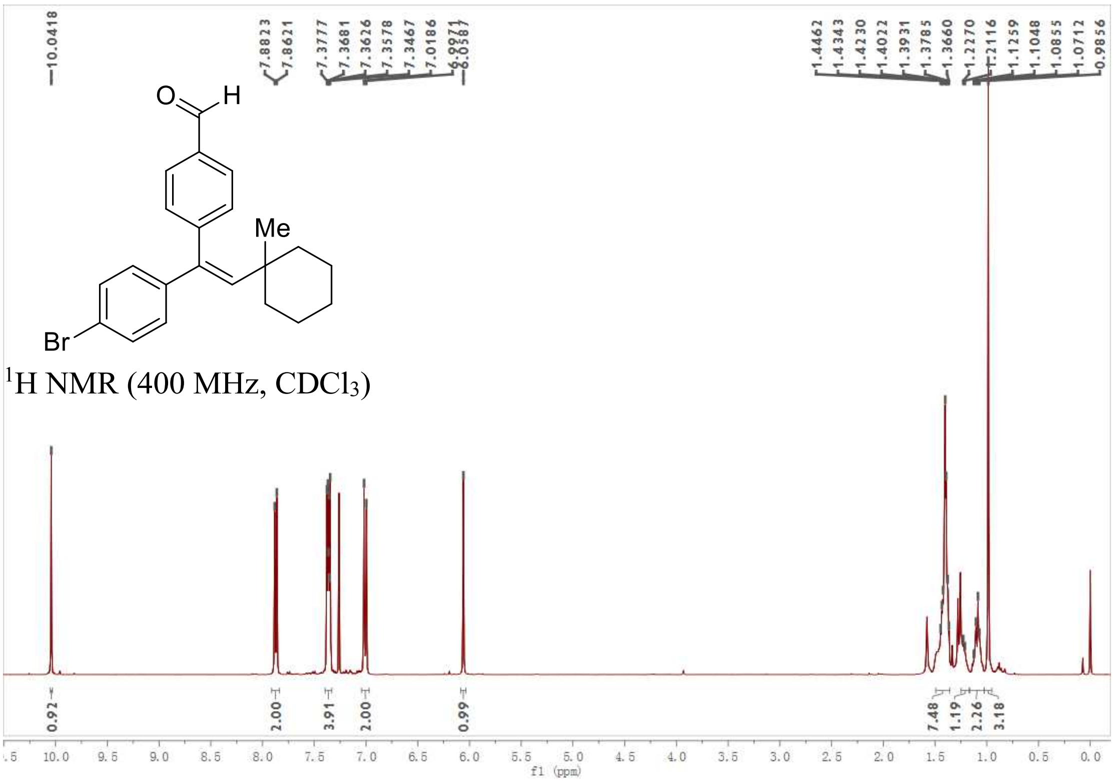

Supplementary Figure 101: ^{1}H NMR Spectra of (*E*)-4-(1-(4-Bromophenyl) -2-(1-methylcyclohexyl)vinyl)benzaldehyde (29)

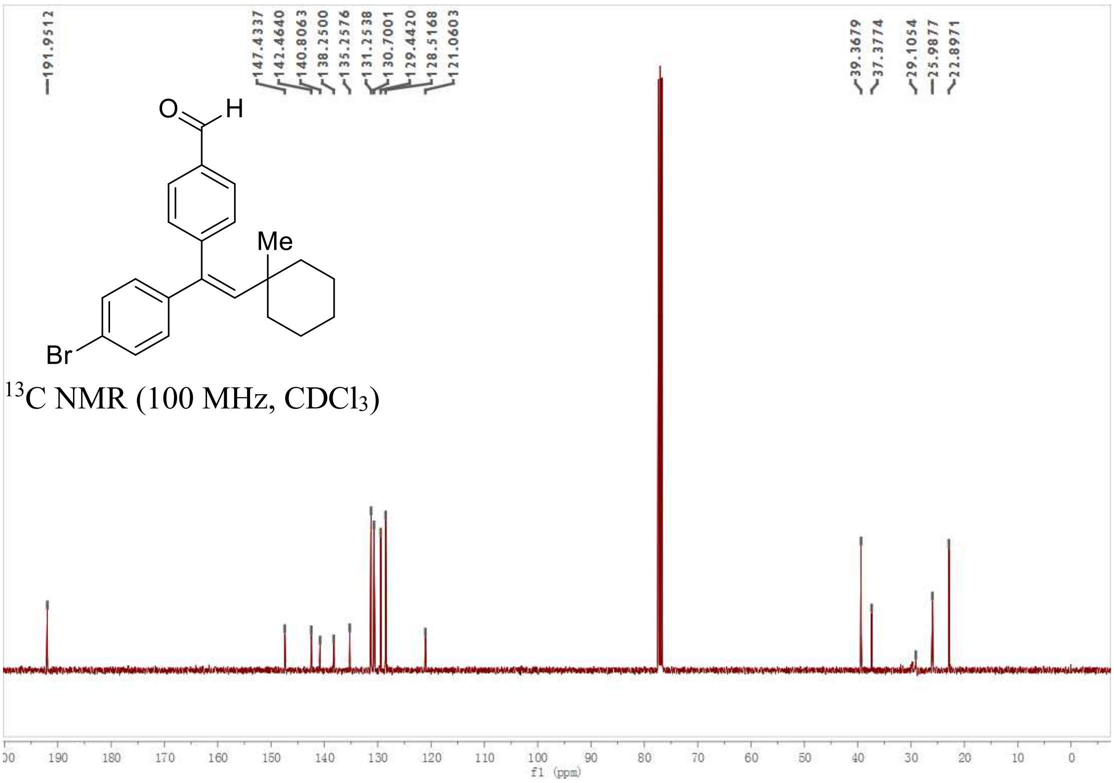

Supplementary Figure 102: ^{13}C NMR Spectra of (*E*)-4-(1-(4-Bromophenyl) -2-(1-methylcyclohexyl)vinyl)benzaldehyde (29)

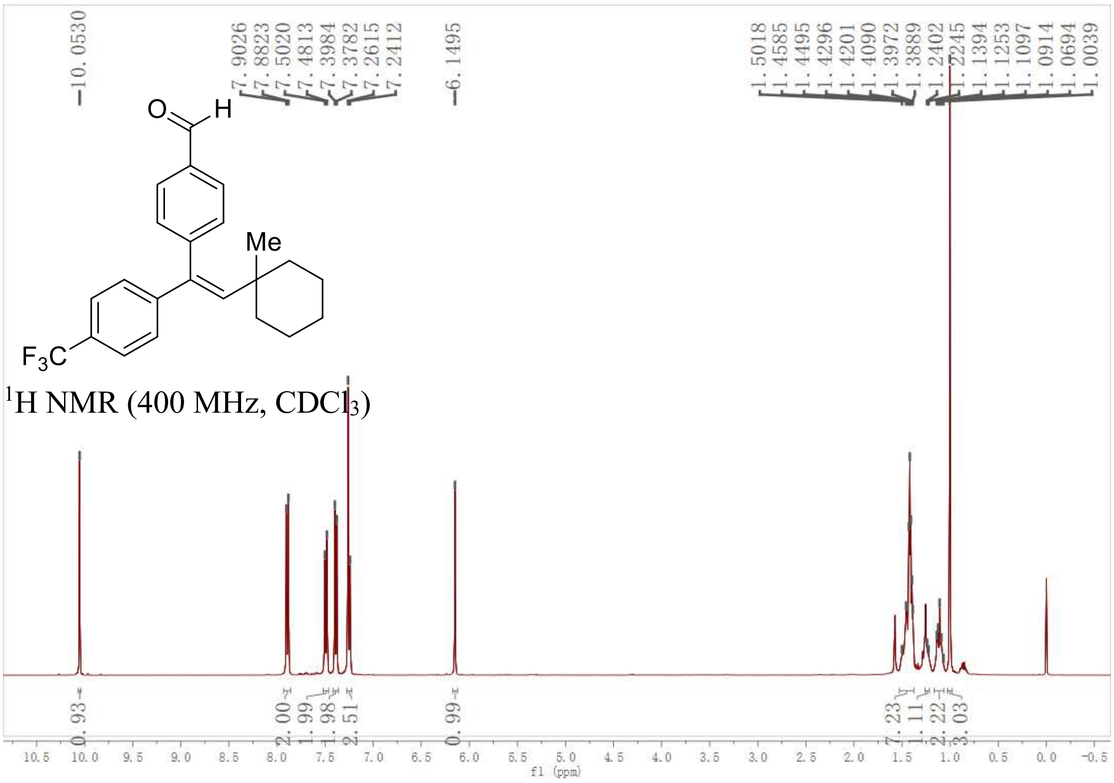

Supplementary Figure 103: ^{1}H NMR Spectra of (*E*)-4-(2-(1-Methylcyclohexyl) -1-(4-(trifluoromethyl)phenyl)vinyl)benzaldehyde (30)

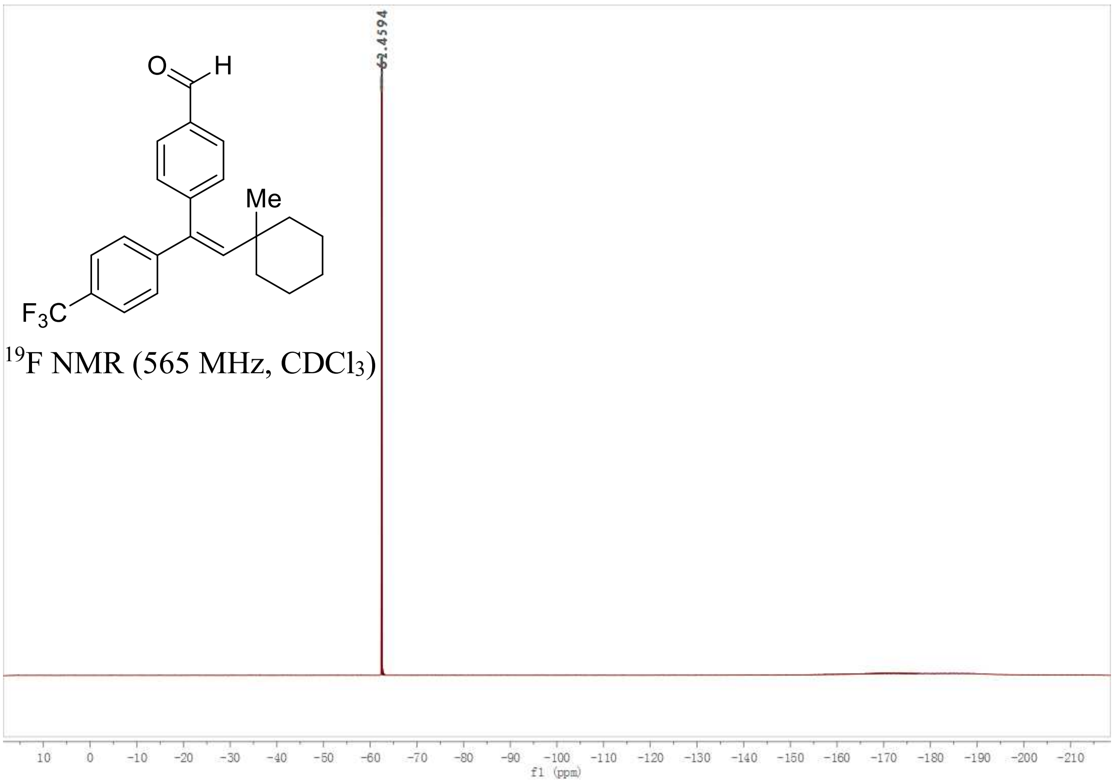

Supplementary Figure 104: ^{19}F NMR Spectra of (*E*)-4-(2-(1-Methylcyclohexyl) -1-(4-(trifluoromethyl)phenyl)vinyl)benzaldehyde (30)

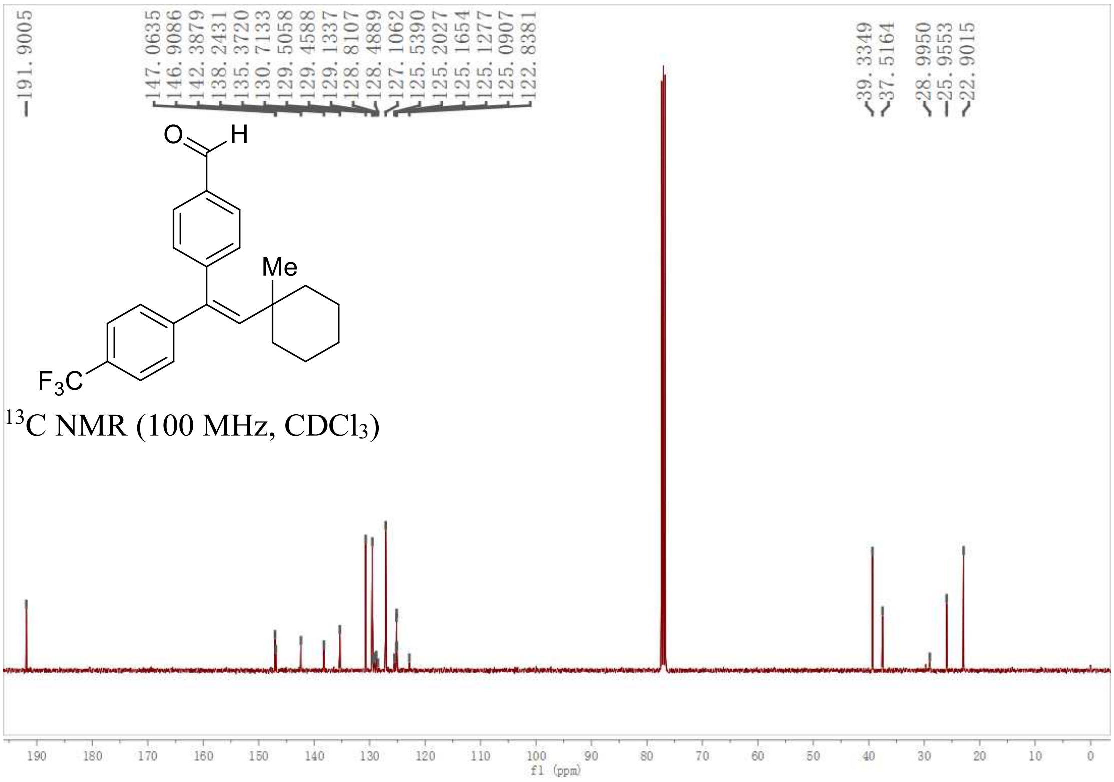

Supplementary Figure 105: ^{13}C NMR Spectra of (*E*)-4-(2-(1-Methylcyclohexyl) -1-(4-(trifluoromethyl)phenyl)vinyl)benzaldehyde (30)

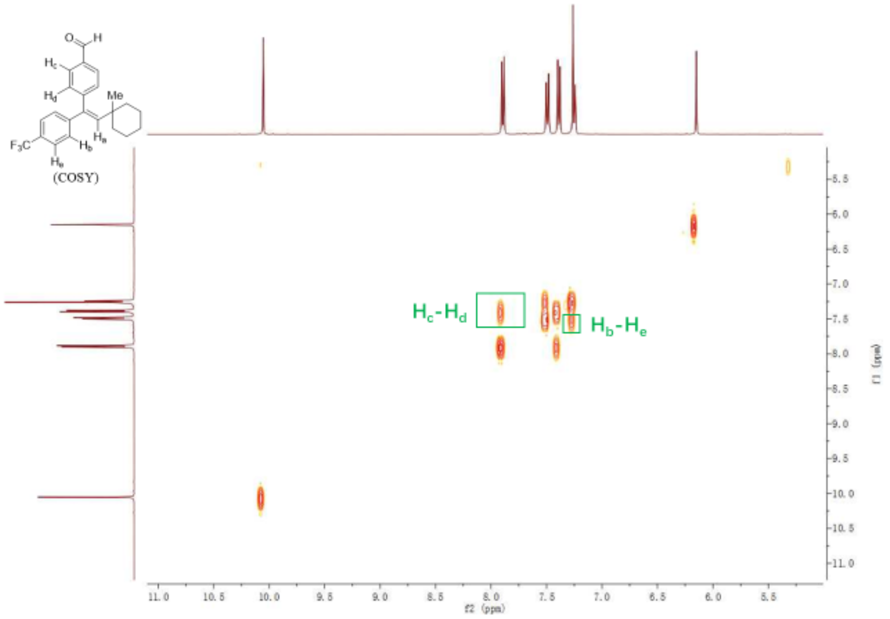

Supplementary Figure 106: COSY Spectra of (*E*)-4-(2-(1-Methylcyclohexyl) -1-(4-(trifluoromethyl)phenyl)vinyl)benzaldehyde (30)

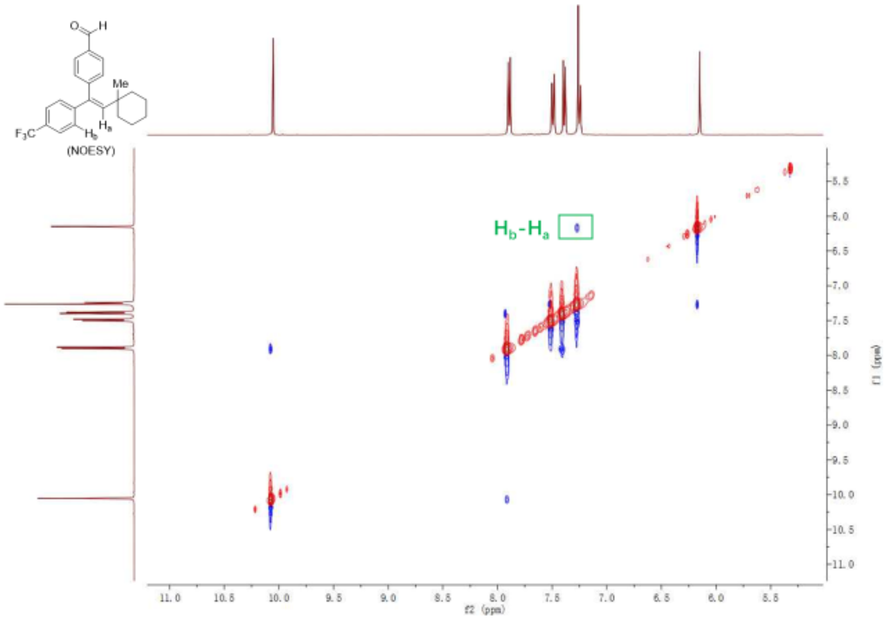

Supplementary Figure 107: NOESY Spectra of (*E*)-4-(2-(1-Methylcyclohexyl) -1-(4-(trifluoromethyl)phenyl)vinyl)benzaldehyde (30)

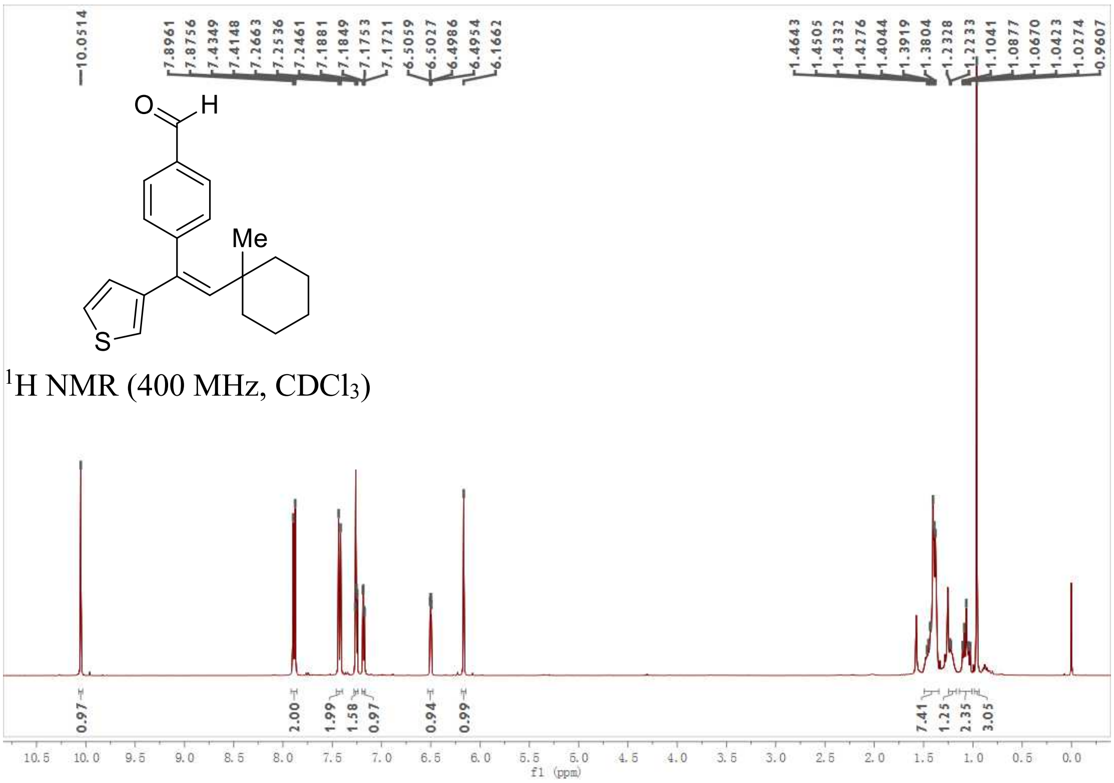

Supplementary Figure 108: ^{1}H NMR Spectra of (*E*)-4-(2-(1-Methylcyclohexyl) -1-(thiophen-3-yl)vinyl)benzaldehyde (*E*-31)

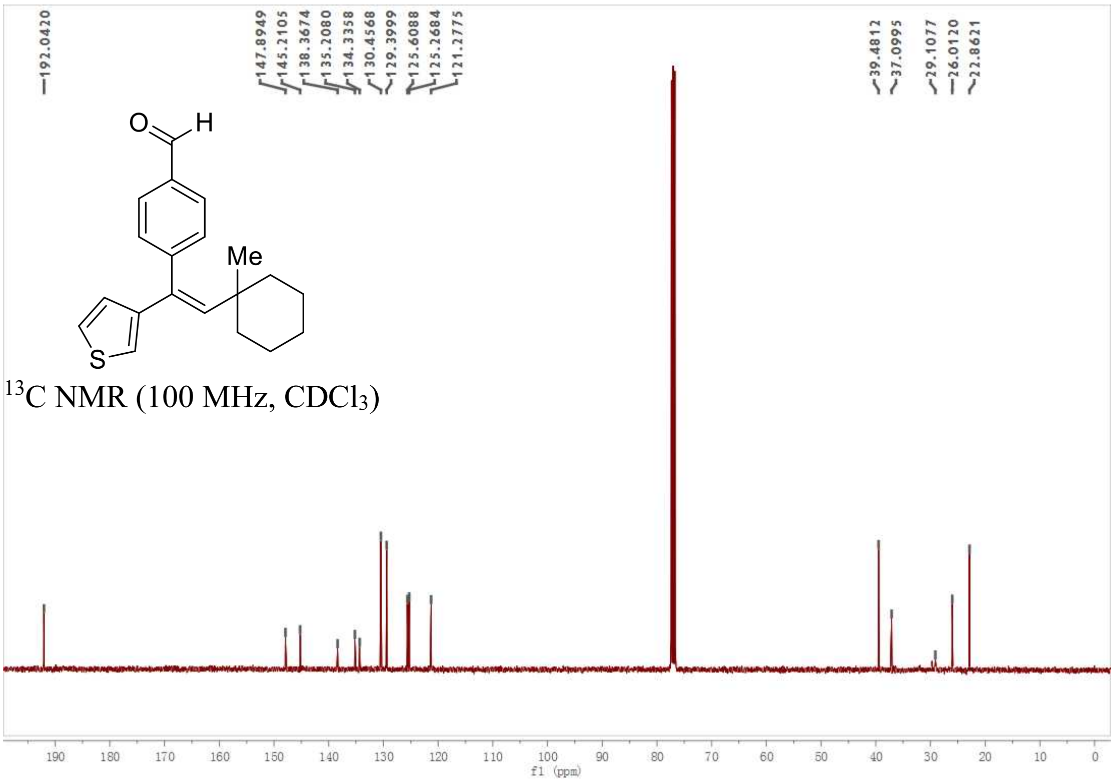

Supplementary Figure 109: ^{13}C NMR Spectra of (*E*)-4-(2-(1-Methylcyclohexyl) -1-(thiophen-3-yl)vinyl)benzaldehyde (*E*-31)

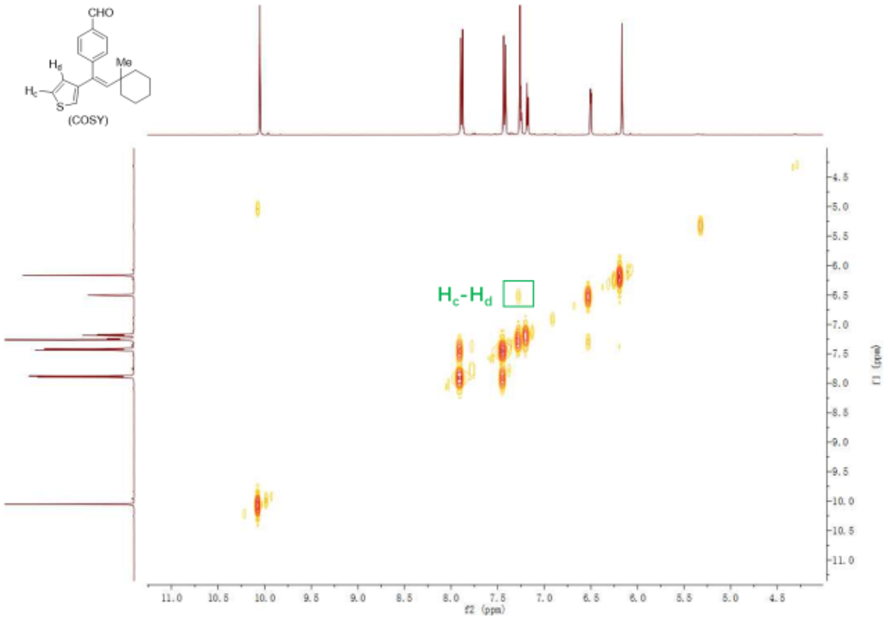

Supplementary Figure 110: COSY Spectra of (*E*)-4-(2-(1-Methylcyclohexyl) -1-(thiophen-3-yl)vinyl)benzaldehyde (*E*-31)

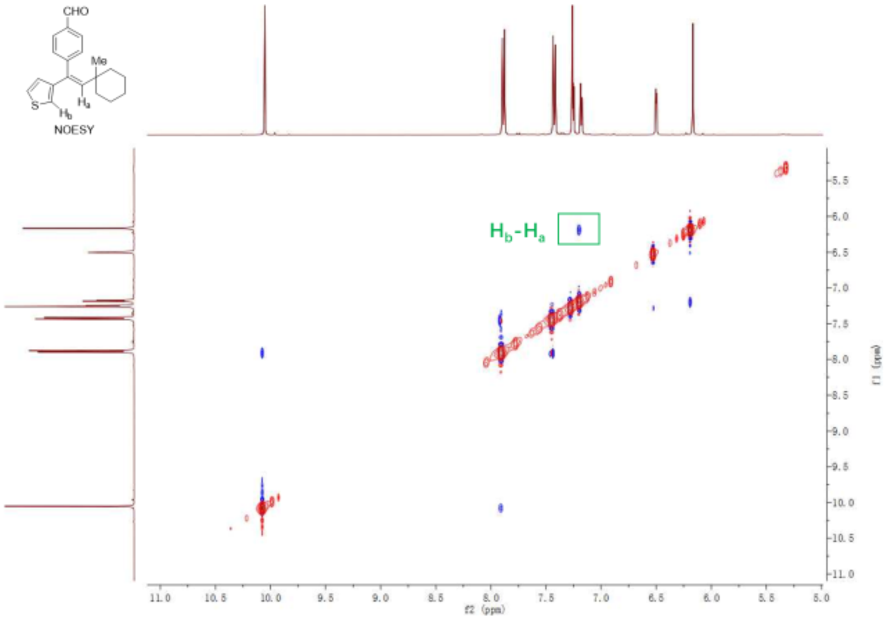

Supplementary Figure 111: NOESY Spectra of (*E*)-4-(2-(1-Methylcyclohexyl) -1-(thiophen-3-yl)vinyl)benzaldehyde (*E*-31)

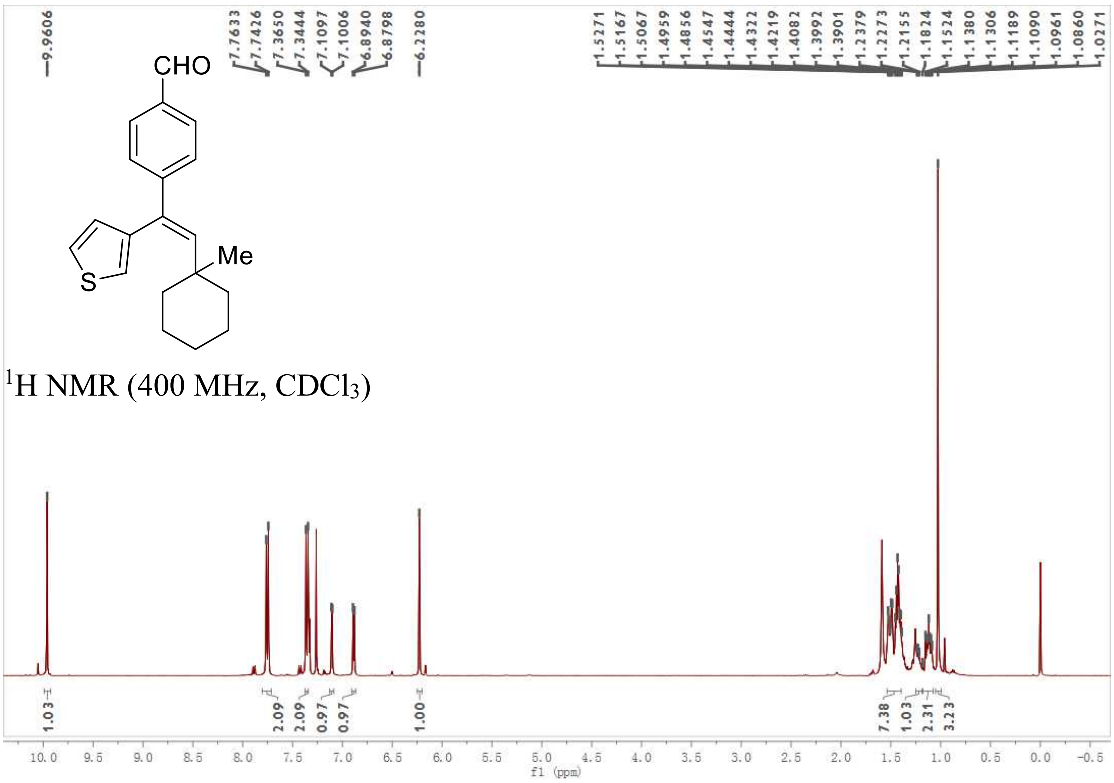

Supplementary Figure 112: ^{1}H NMR Spectra of (Z)-4-(2-(1-methylcyclohexyl) -1-(thiophen-3-yl)vinyl)benzaldehyde (Z-31)

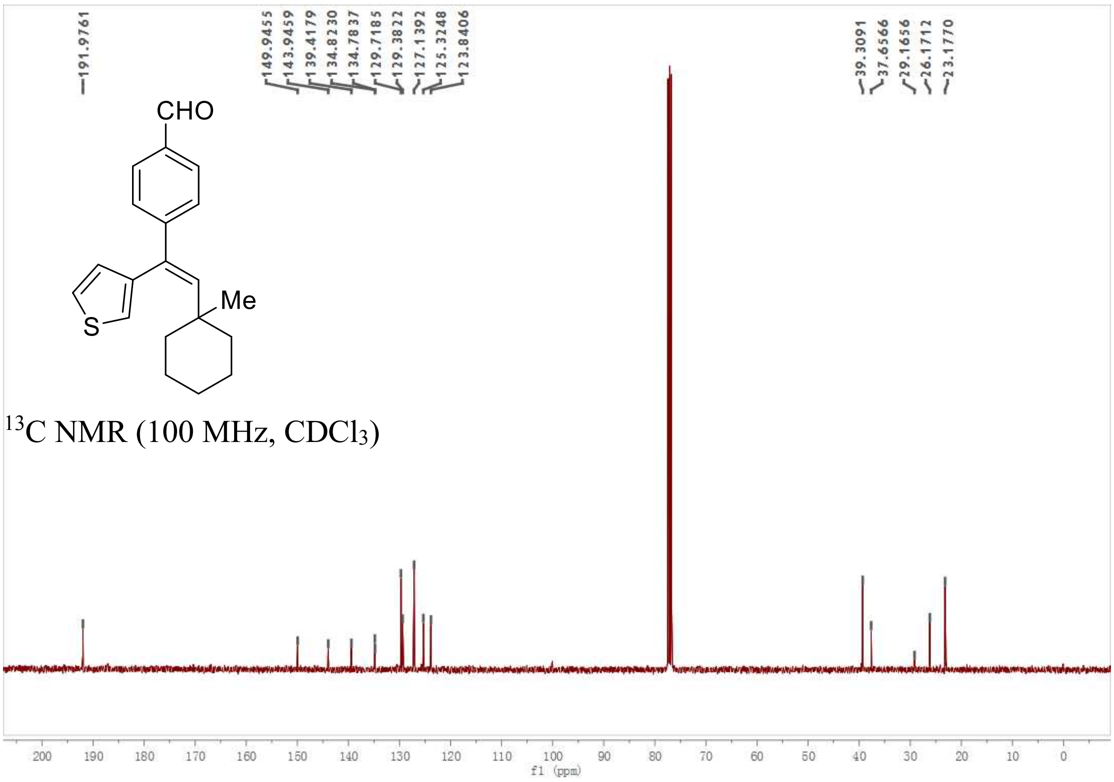

Supplementary Figure 113: ^{13}C NMR Spectra of (Z)-4-(2-(1-methylcyclohexyl) -1-(thiophen-3-yl)vinyl)benzaldehyde (Z-31)

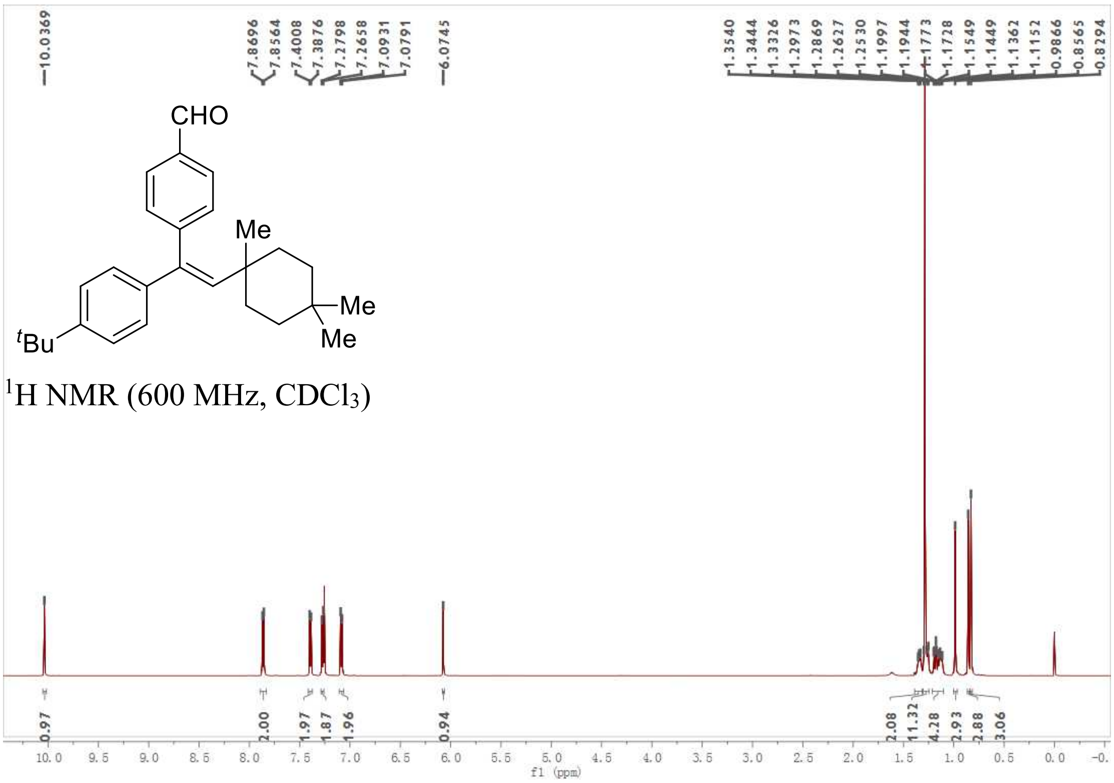

Supplementary Figure 114: ^{1}H NMR Spectra of (Z)-4-(1-(4-(tert-Butyl)phenyl) -2-(1,4,4-trimethylcyclohexyl)vinyl)benzaldehyde (32)

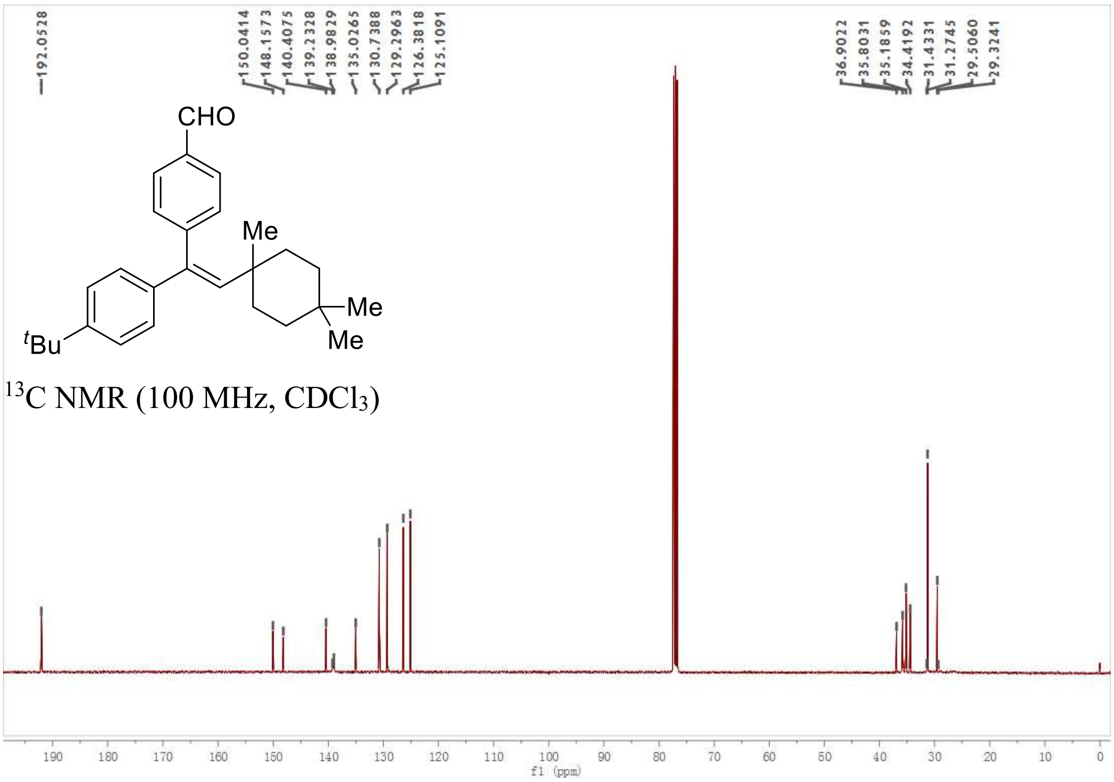

Supplementary Figure 115: ^{13}C NMR Spectra of (Z)-4-(1-(4-(tert-Butyl)phenyl) -2-(1,4,4-trimethylcyclohexyl)vinyl)benzaldehyde (32)

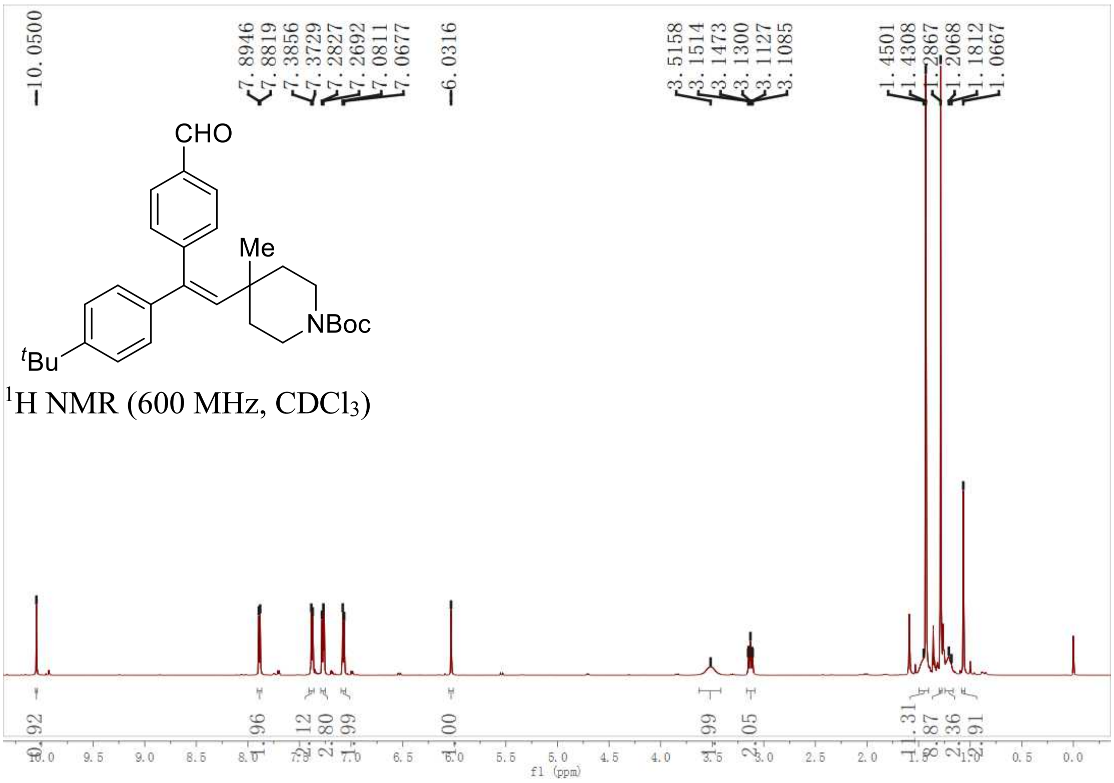

Supplementary Figure 116: ^{1}H NMR Spectra of (Z)-tert-Butyl-4 -(2-(4-(tert-butyl)phenyl)-2-(4-formylphenyl)vinyl)-4-methylpiperidine-1-carboxylate (33)

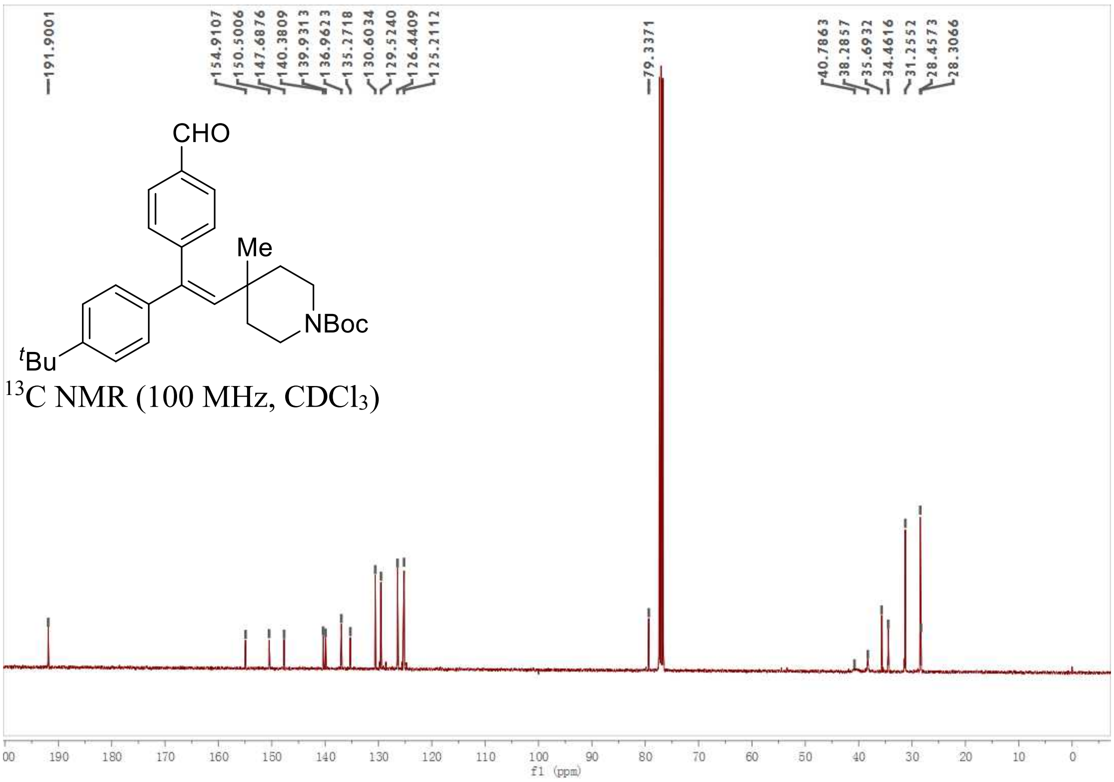

Supplementary Figure 117: ^{13}C NMR Spectra of (Z)-tert-Butyl-4 -(2-(4-(tert-butyl)phenyl)-2-(4-formylphenyl)vinyl)-4-methylpiperidine-1-carboxylate (33)

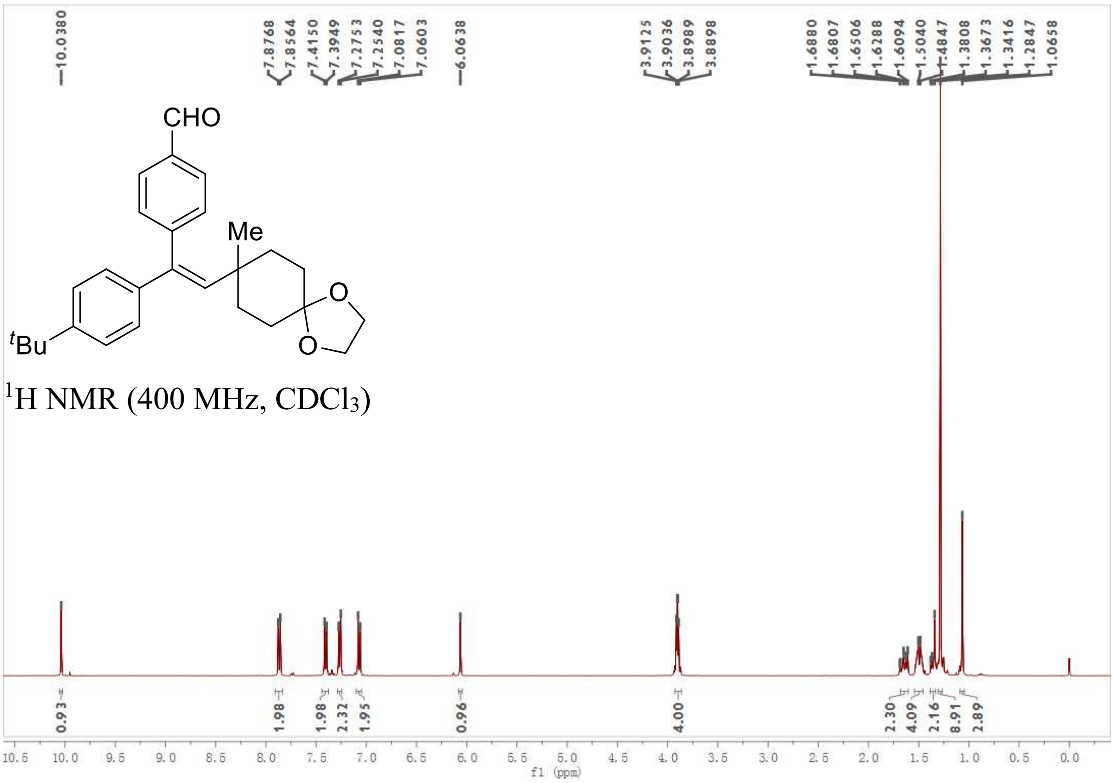

Supplementary Figure 118: ^{1}H NMR Spectra of (Z)-4-(1- (4-(tert-Butyl) phenyl)-2-(8-methyl-1,4-dioxaspiro[4.5]decan-8-yl)vinyl)benzaldehyde (34)

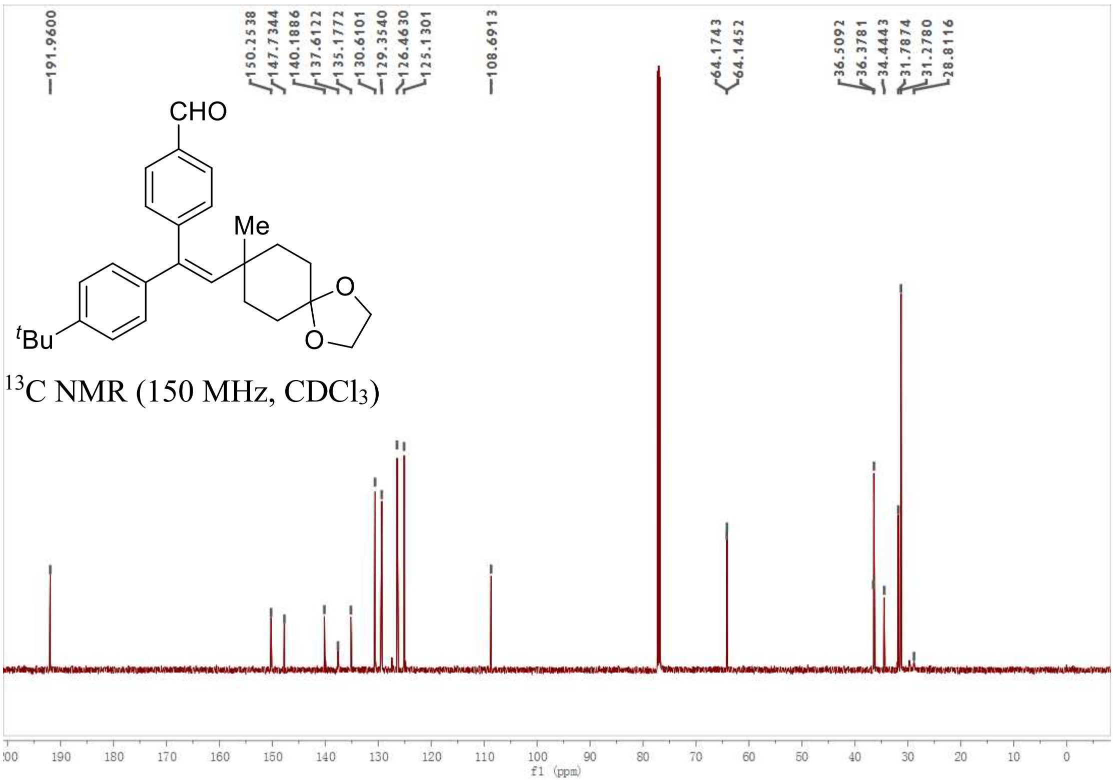

Supplementary Figure 119: ^{13}C NMR Spectra of (Z)-4-(1- (4-(tert-Butyl) phenyl)-2-(8-methyl-1,4-dioxaspiro[4.5]decan-8-yl)vinyl)benzaldehyde (34)

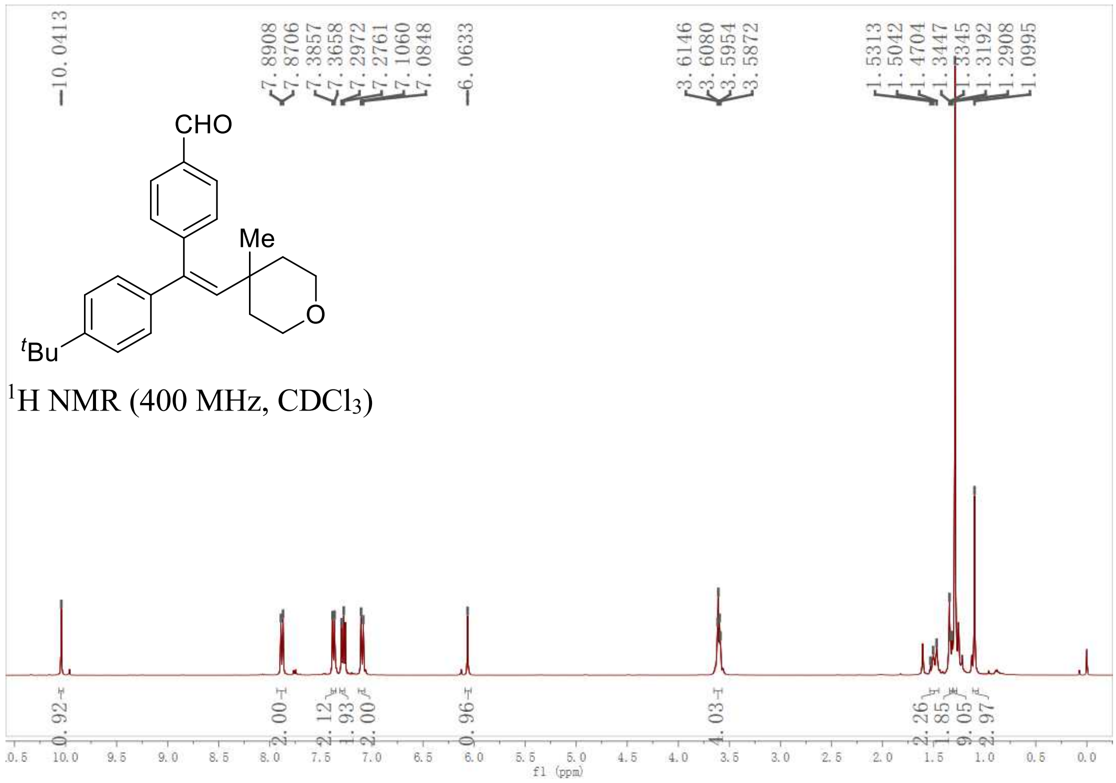

Supplementary Figure 120: ^{1}H NMR Spectra of (Z)-4-(1- (4-(tert-Butyl) phenyl)-2-(4-methyltetrahydro-2H-pyran-4-yl)vinyl)benzaldehyde (35)

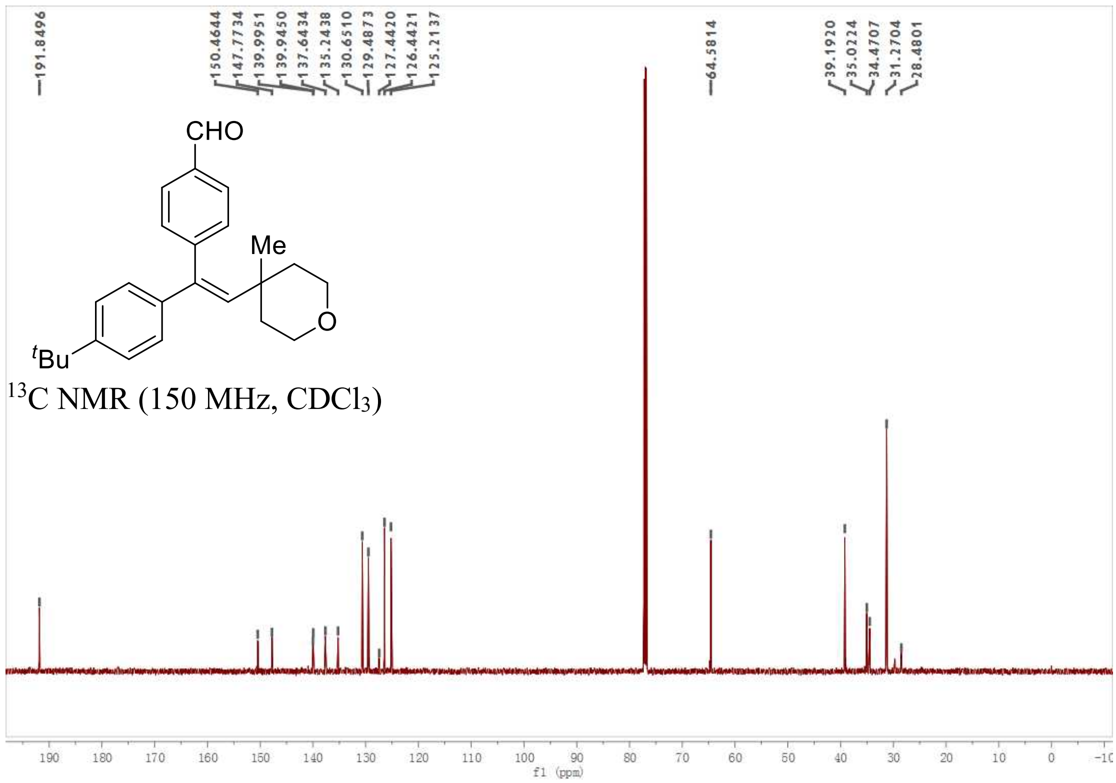

Supplementary Figure 121: ^{13}C NMR Spectra of (Z)-4-(1- (4-(tert-Butyl) phenyl)-2-(4-methyltetrahydro-2H-pyran-4-yl)vinyl)benzaldehyde (35)

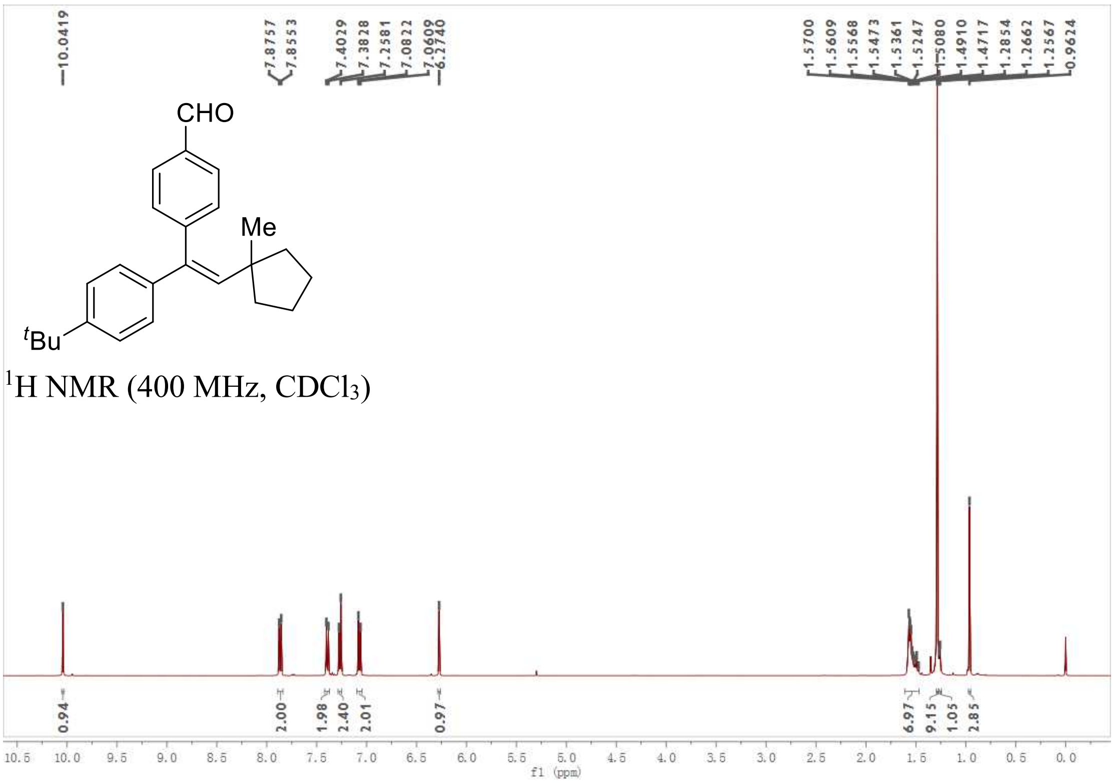

Supplementary Figure 122: ^{1}H NMR Spectra of (Z)-4-(1-(4-(tert-Butyl) phenyl)-2-(1-methylcyclopentyl)vinyl)benzaldehyde (36)

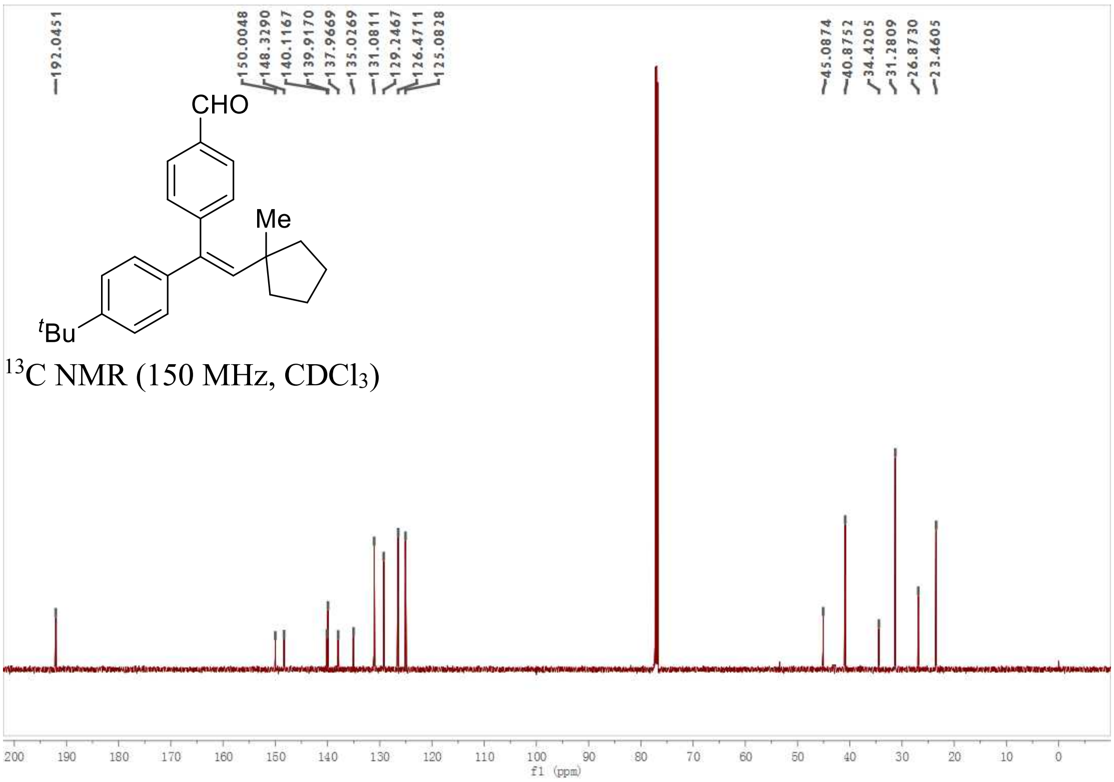

Supplementary Figure 123: ^{13}C NMR Spectra of (Z)-4-(1-(4-(tert-Butyl) phenyl)-2-(1-methylcyclopentyl)vinyl)benzaldehyde (36)

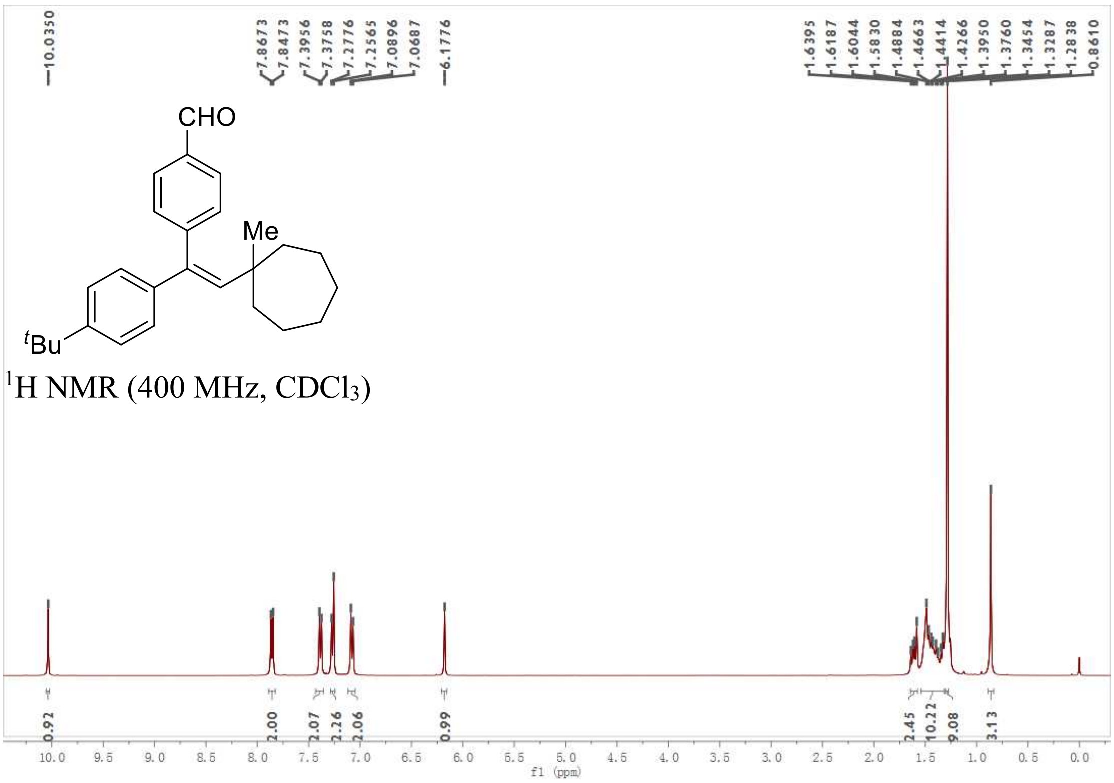

Supplementary Figure 124: ^{1}H NMR Spectra of (Z)-4-(1-(4-(tert-Butyl)phenyl) -2-(1-methylcycloheptyl)vinyl)benzaldehyde (37)

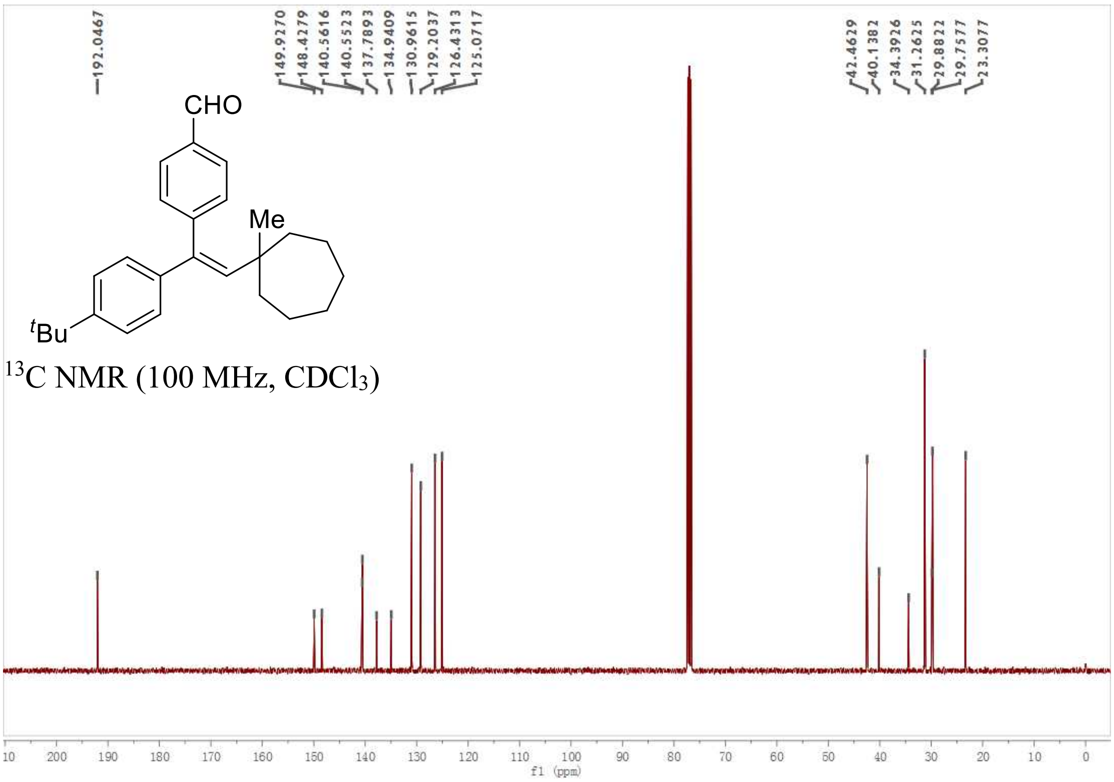

Supplementary Figure 125: ^{13}C NMR Spectra of (Z)-4-(1-(4-(tert-Butyl)phenyl) -2-(1-methylcycloheptyl)vinyl)benzaldehyde (37)

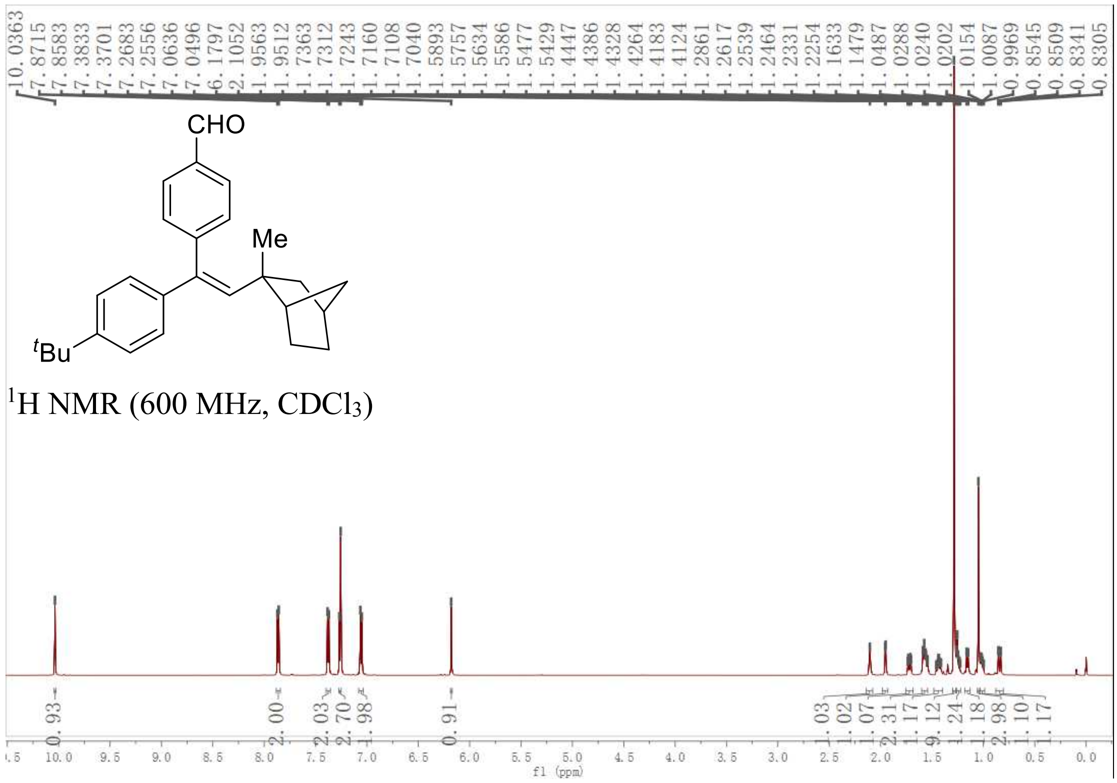

Supplementary Figure 126: ^{1}H NMR Spectra of 4-((Z)-1-(4-(tert-Butyl)phenyl)-2-((1S,2R,4R)-2-methylbicyclo[2.2.1]heptan-2-yl)vinyl)benzaldehyde (38)

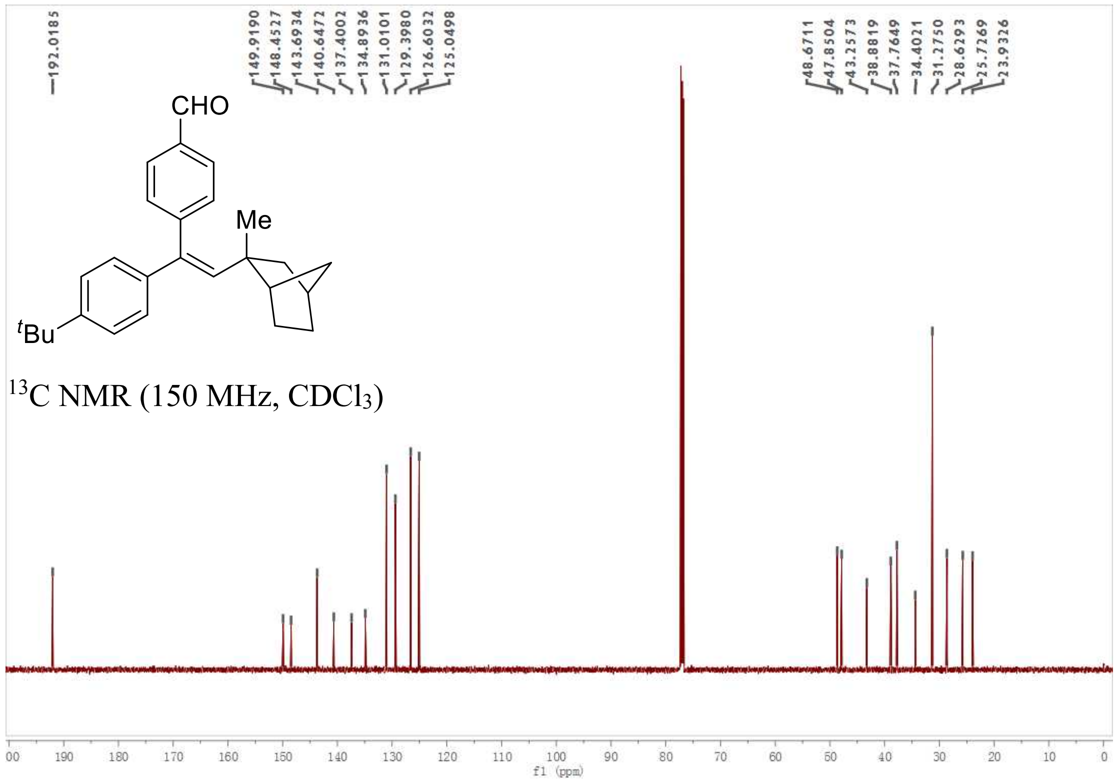

Supplementary Figure 127: ^{13}C NMR Spectra of 4-((Z)-1-(4-(tert-Butyl)phenyl)-2-((1S,2R,4R)-2-methylbicyclo[2.2.1]heptan-2-yl)vinyl)benzaldehyde (38)

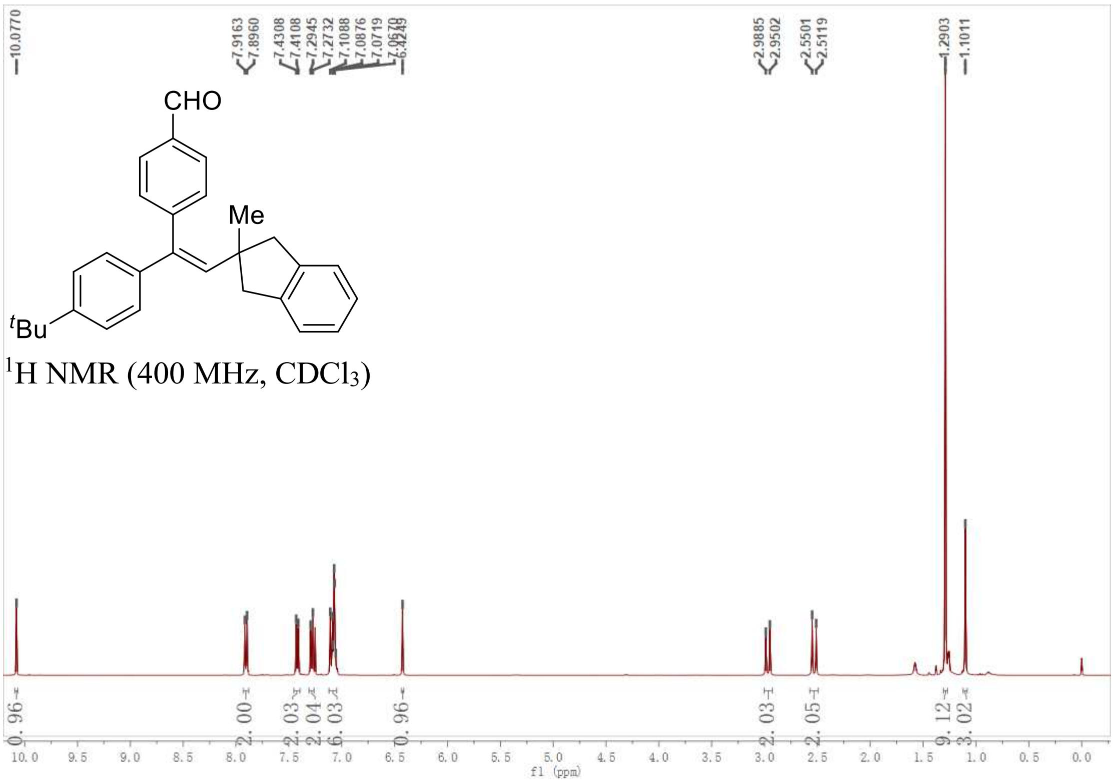

Supplementary Figure 128: ^{1}H NMR Spectra of (Z)-4-(1-(4-(tert-Butyl)phenyl)-2 - (2-methyl-2,3-dihydro-1H-inden-2-yl)vinyl)benzaldehyde (39)

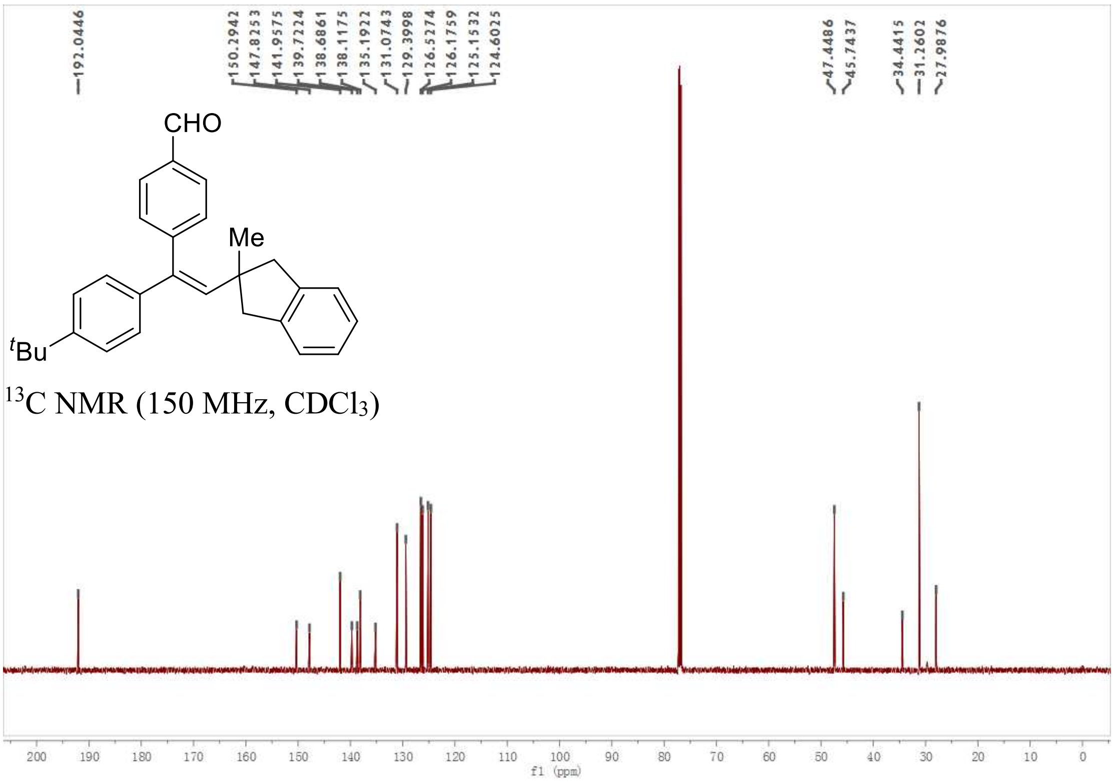

Supplementary Figure 129: ^{13}C NMR Spectra of (Z)-4-(1-(4-(tert-Butyl)phenyl)-2- (2-methyl-2,3-dihydro-1H-inden-2-yl)vinyl)benzaldehyde (39)

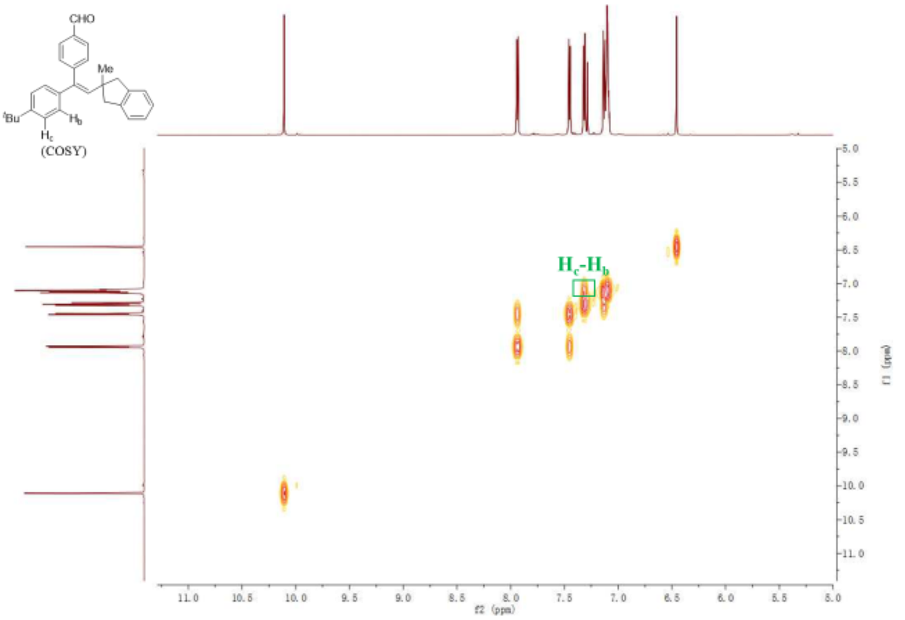

Supplementary Figure 130: COSY Spectra of (Z)-4-(1-(4-(tert-Butyl)phenyl)-2 - (2-methyl-2,3-dihydro-1H-inden-2-yl)vinyl)benzaldehyde (39)

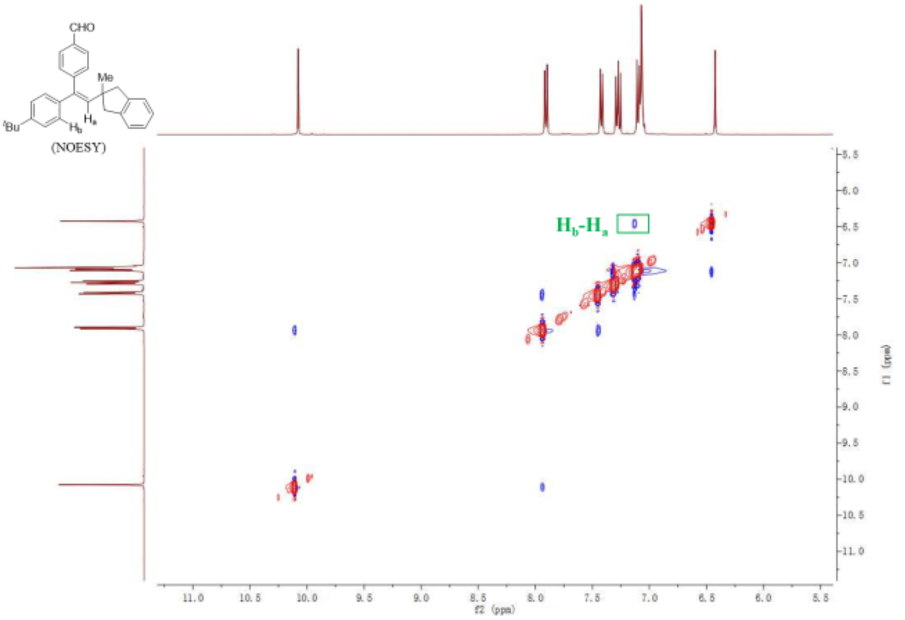

Supplementary Figure 131: NOESY Spectra of (Z)-4-(1-(4-(tert-Butyl)phenyl)-2 -(2-methyl-2,3-dihydro-1H-inden-2-yl)vinyl)benzaldehyde (39)

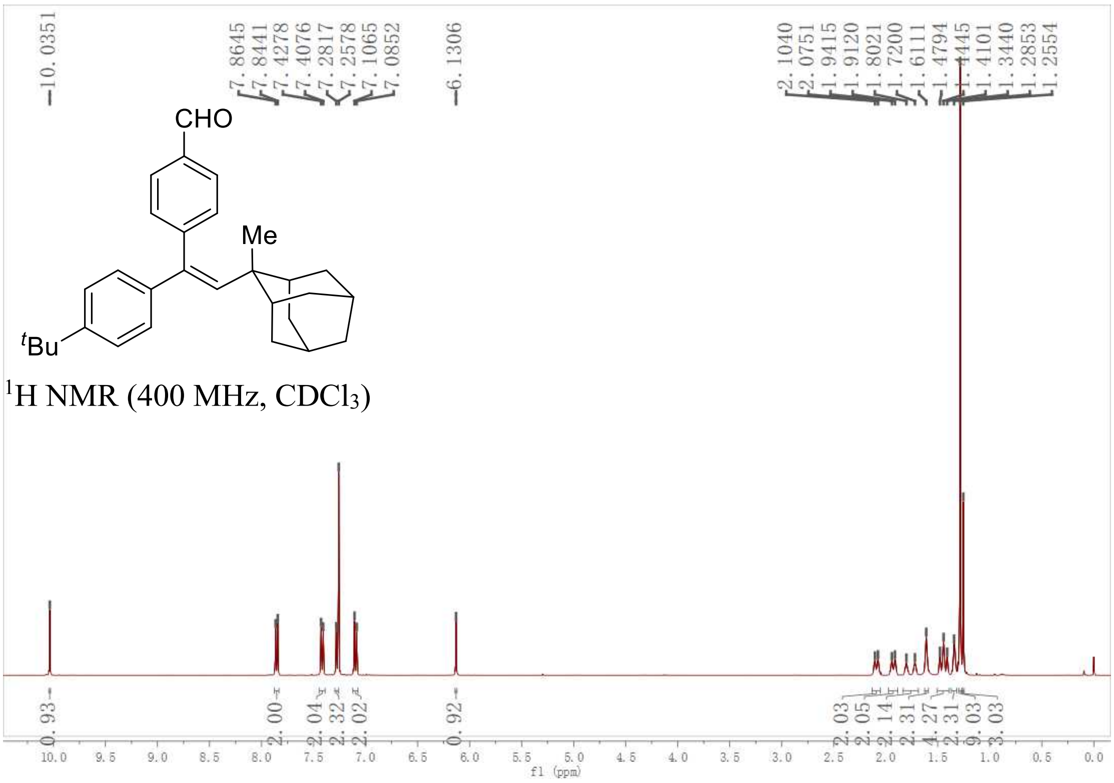

Supplementary Figure 132: ^{1}H NMR Spectra of 4-((Z)-1- (4-(tert-Butyl) phenyl)-2-((1R,3S,5r,7r)-2-methyladamantan-2-yl)vinyl)benzaldehyde (40)

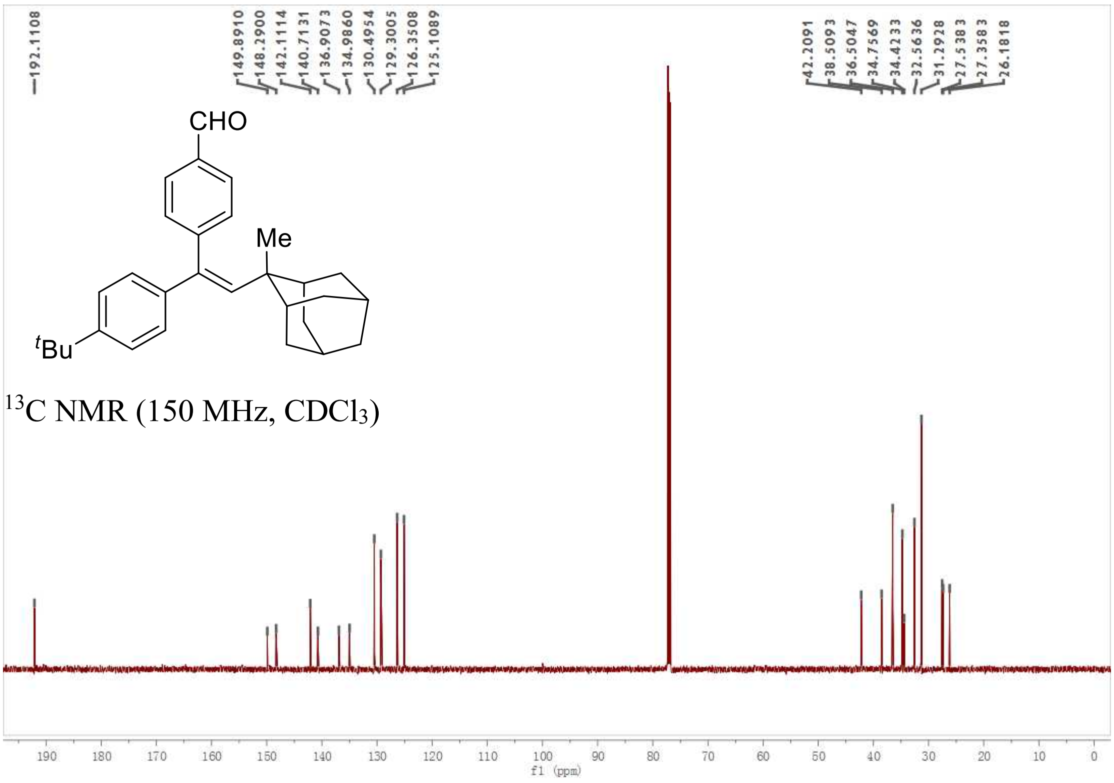

Supplementary Figure 133: ^{13}C NMR Spectra of 4-((Z)-1- (4-(tert-Butyl) phenyl)-2-((1R,3S,5r,7r)-2-methyladamantan-2-yl)vinyl)benzaldehyde (40)

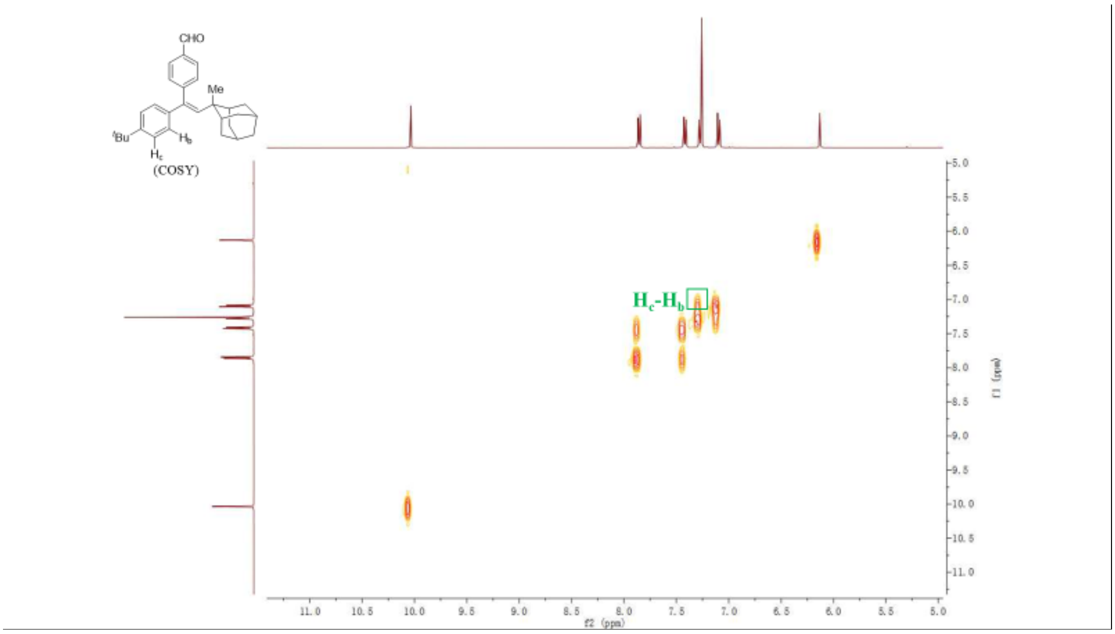

Supplementary Figure 134: COSY Spectra of 4-((Z)-1- (4-(tert-Butyl) phenyl)-2-((1R,3S,5r,7r)-2-methyladamantan-2-yl)vinyl)benzaldehyde (40)

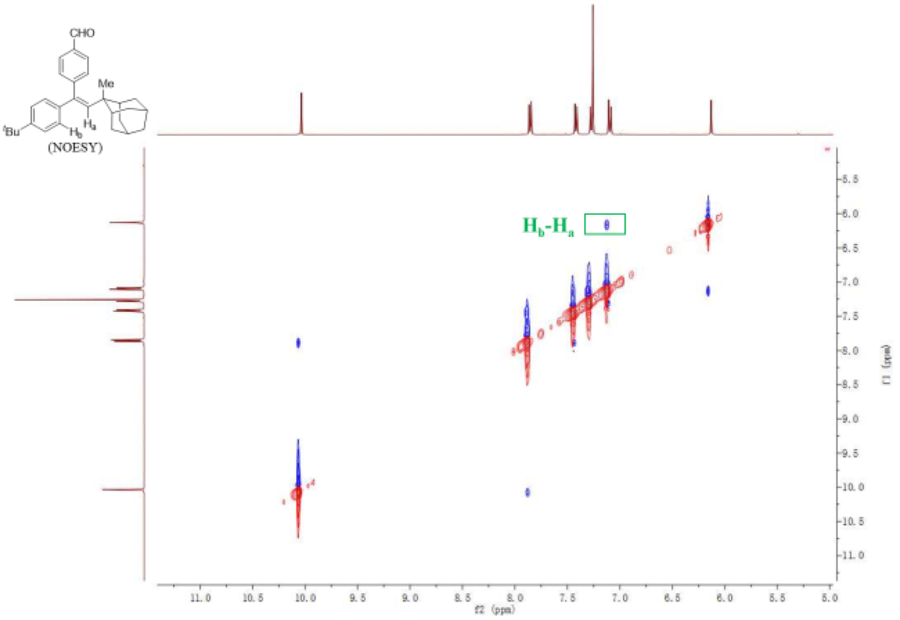

Supplementary Figure 135: NOESY Spectra of 4-((Z)-1- (4-(tert-Butyl) phenyl)-2-((1R,3S,5r,7r)-2-methyladamantan-2-yl)vinyl)benzaldehyde (40)

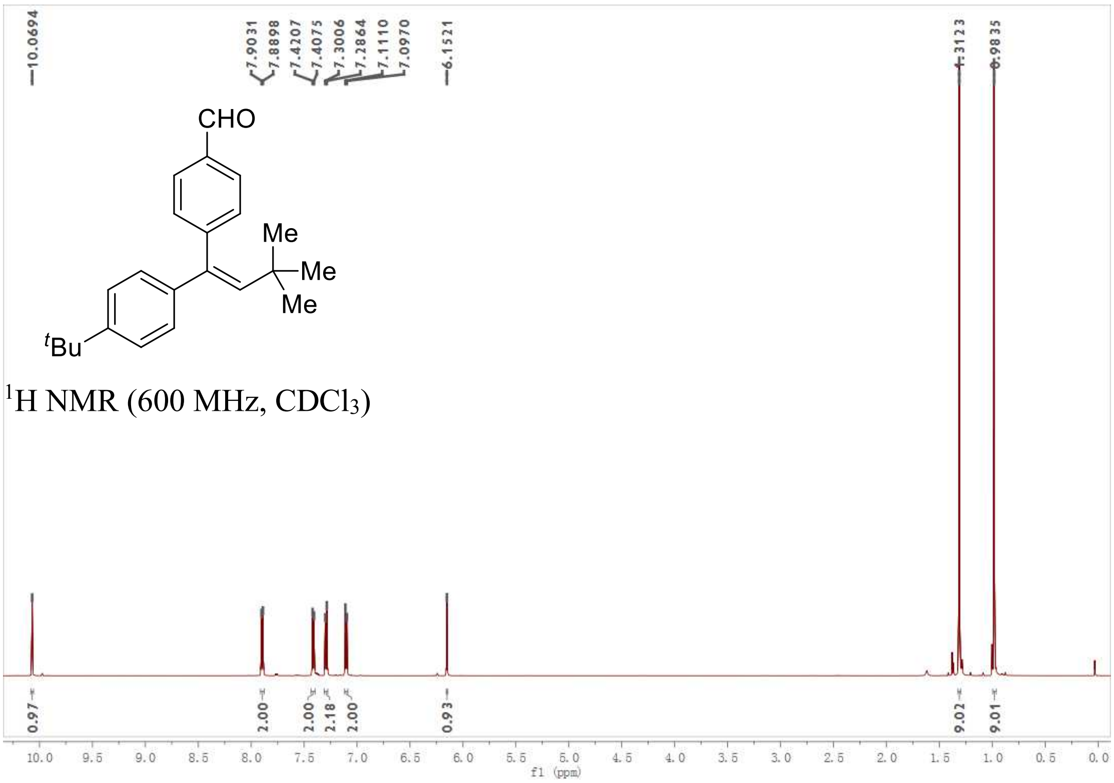

Supplementary Figure 136: ^{1}H NMR Spectra of (Z)-4-(1-(4-(tert-Butyl) phenyl)-3,3-dimethylbut-1-en-1-yl)benzaldehyde (Z-41)

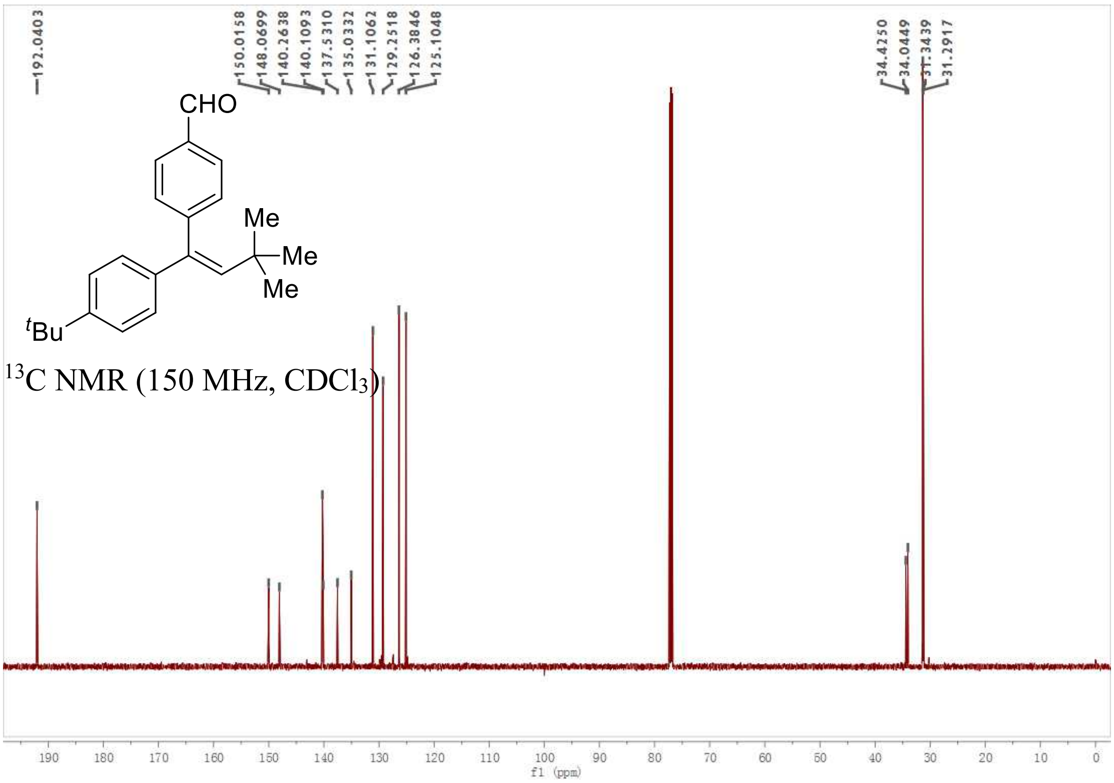

Supplementary Figure 137: ^{13}C NMR Spectra of (Z)-4-(1-(4-(tert-Butyl) phenyl)-3,3-dimethylbut-1-en-1-yl)benzaldehyde (Z-41)

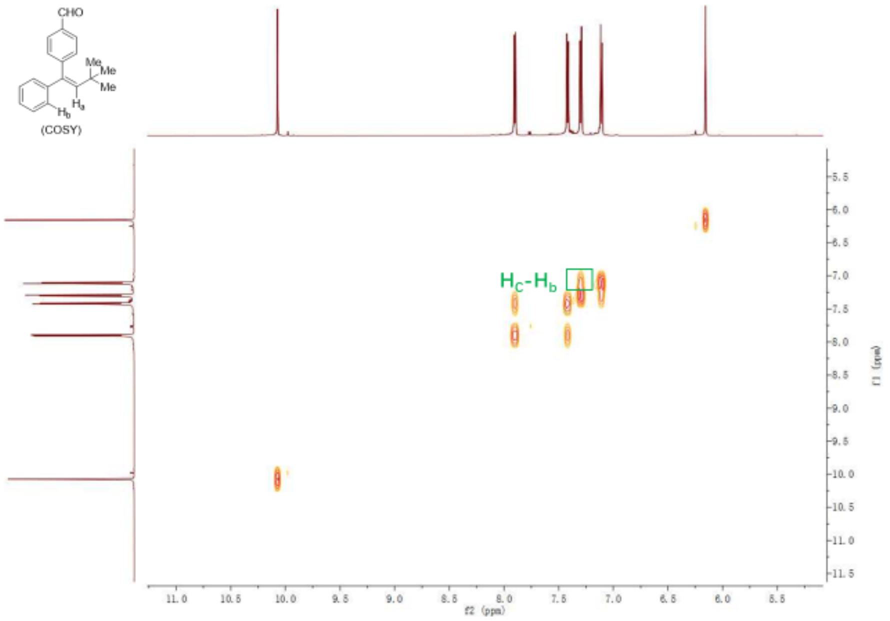

Supplementary Figure 138: COSY Spectra of (Z)-4-(1-(4-(tert-Butyl) phenyl)-3,3-dimethylbut-1-en-1-yl)benzaldehyde (Z-41)

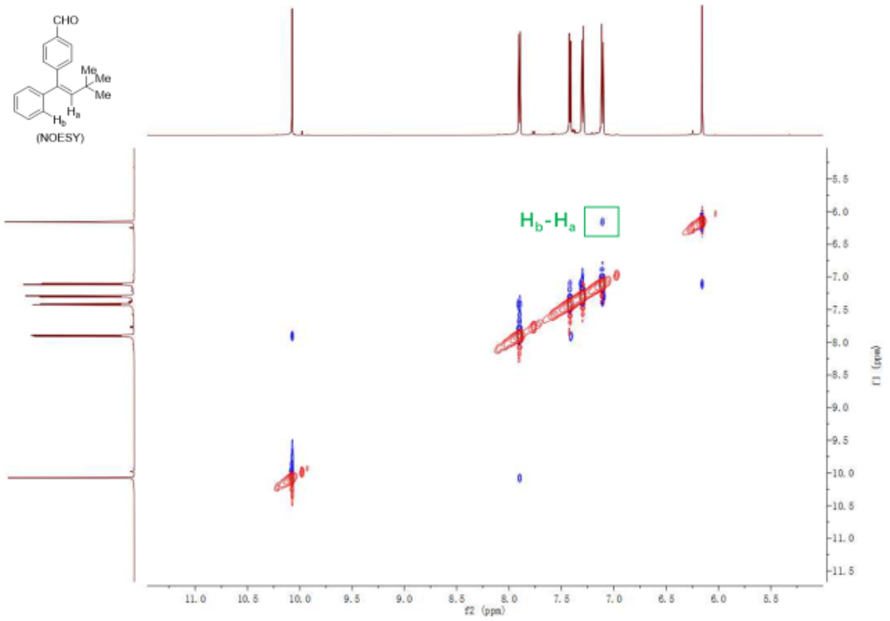

Supplementary Figure 139: NOESY Spectra of (Z)-4-(1-(4-(tert-Butyl) phenyl)-3,3-dimethylbut-1-en-1-yl)benzaldehyde (Z-41)

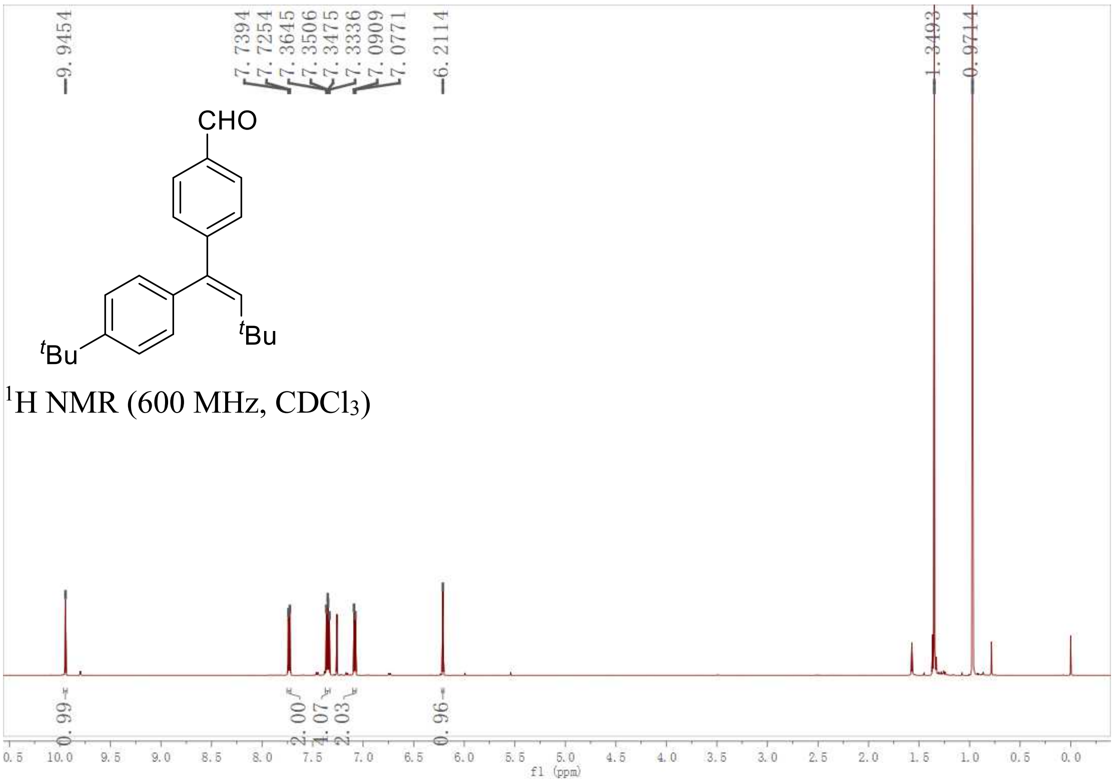

Supplementary Figure 140: ^{1}H NMR Spectra of (*E*)-4-(1-(4-(*tert*-Butyl)phenyl)-3,3-dimethylbut-1-en-1-yl)benzaldehyde (*E*-41)

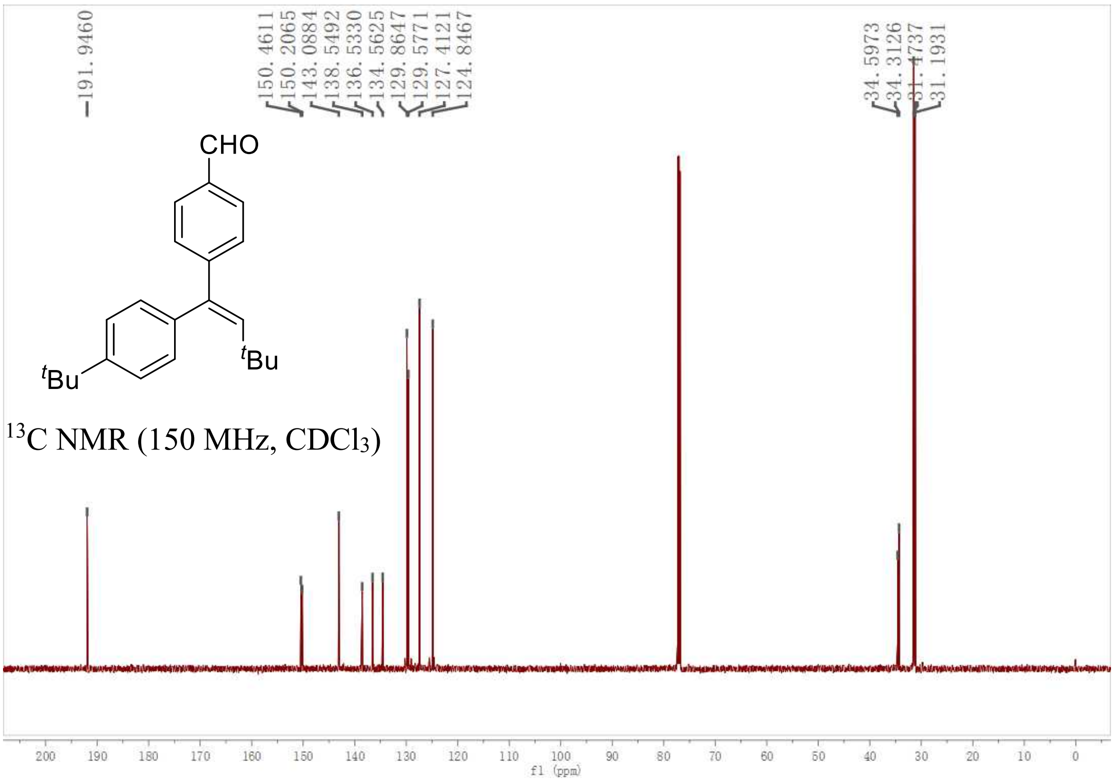

Supplementary Figure 141: ^{13}C NMR Spectra of (*E*)-4-(1-(4-(*tert*-Butyl)phenyl)-3,3-dimethylbut-1-en-1-yl)benzaldehyde (*E*-41)

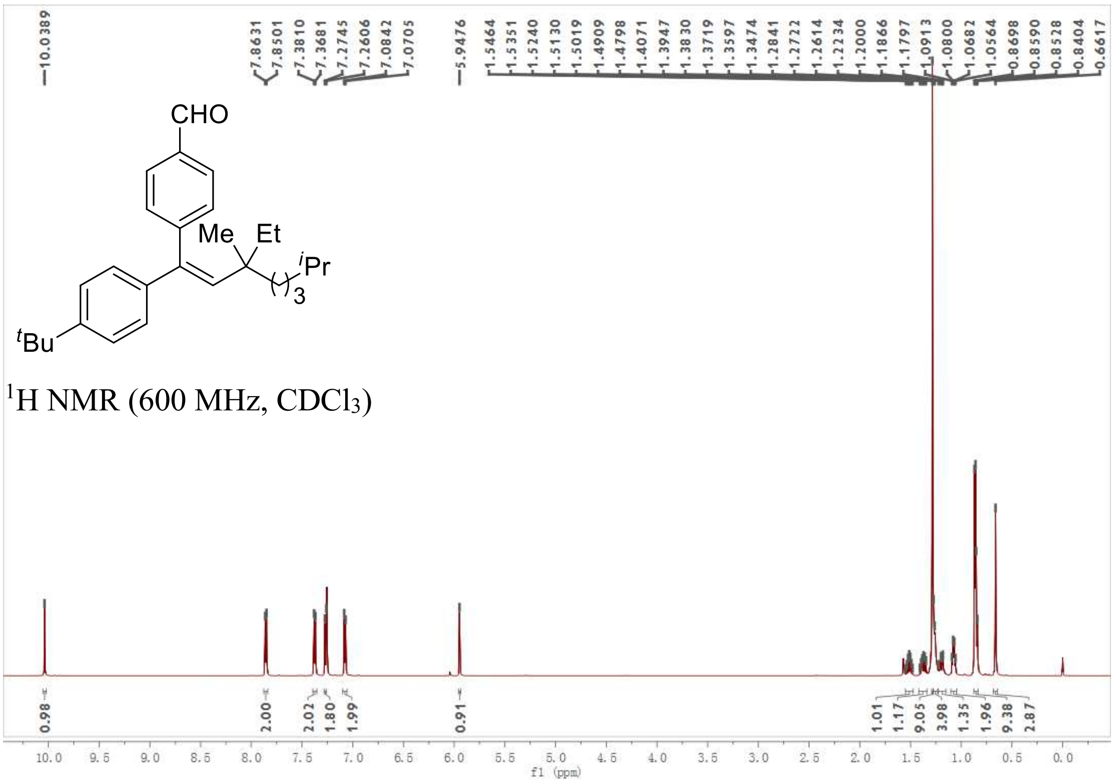

Supplementary Figure 142: ^{1}H NMR Spectra of (Z)-4-(1-(4-(tert-Butyl)phenyl)-3-ethyl-3,7-dimethyloct-1-en-1-yl)benzaldehyde (42)

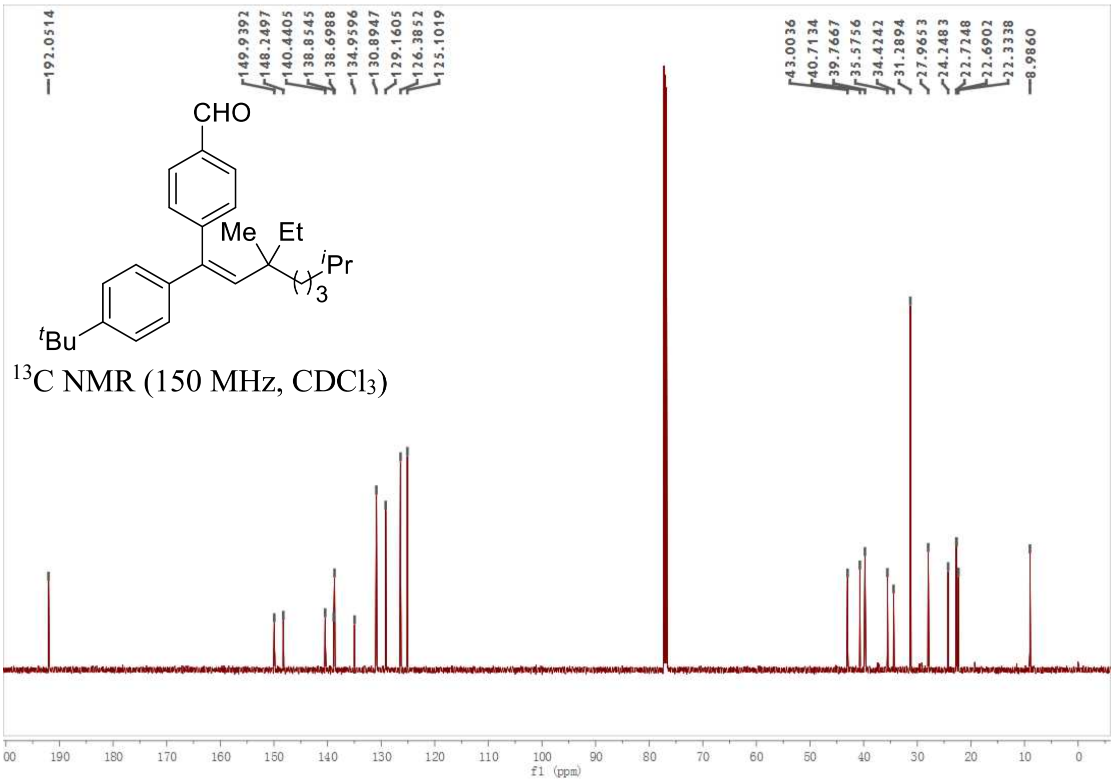

Supplementary Figure 143: ^{13}C NMR Spectra of (Z)-4-(1-(4-(tert-Butyl)phenyl)-3-ethyl-3,7-dimethyloct-1-en-1-yl)benzaldehyde (42)

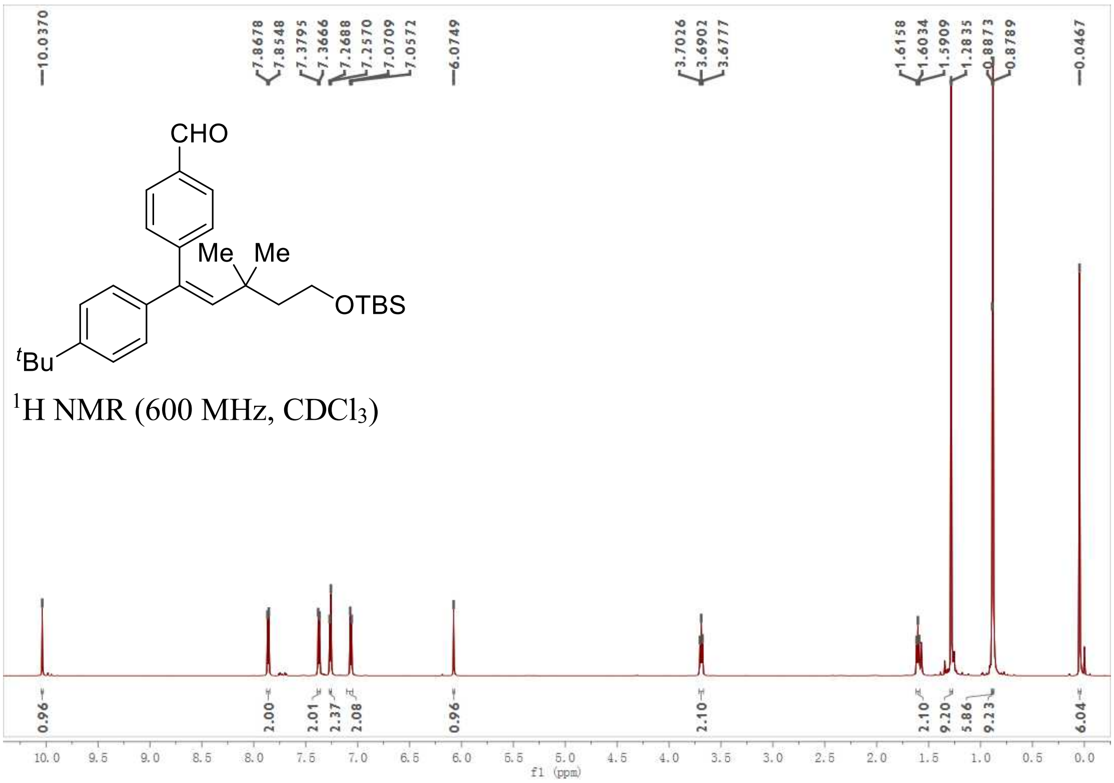

Supplementary Figure 144: ^{1}H NMR Spectra of (Z)-4-(1- (4- (tert -Butyl) phenyl) -5-((tert-butyldimethylsilyl)oxy)-3,3-dimethylpent-1-en-1-yl)benzaldehyde (43)

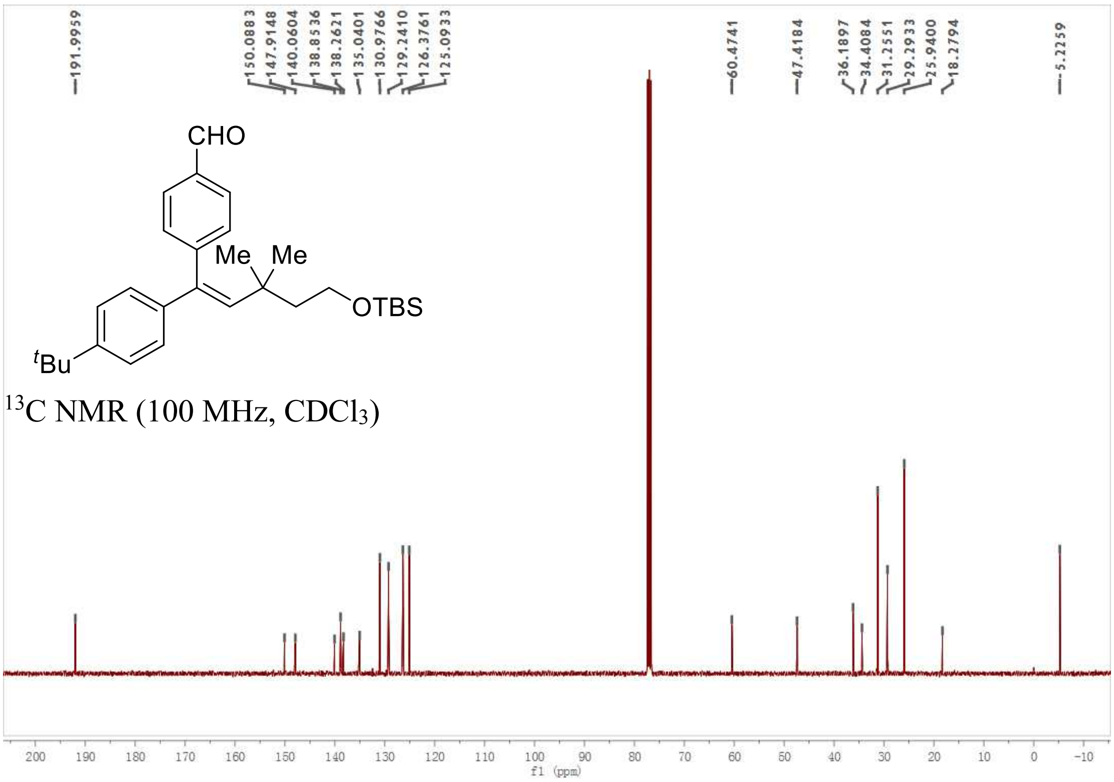

Supplementary Figure 145: ^{13}C NMR Spectra of (Z)-4-(1- (4- (tert -Butyl) phenyl) -5-((tert-butyldimethylsilyl)oxy)-3,3-dimethylpent-1-en-1-yl)benzaldehyde (43)

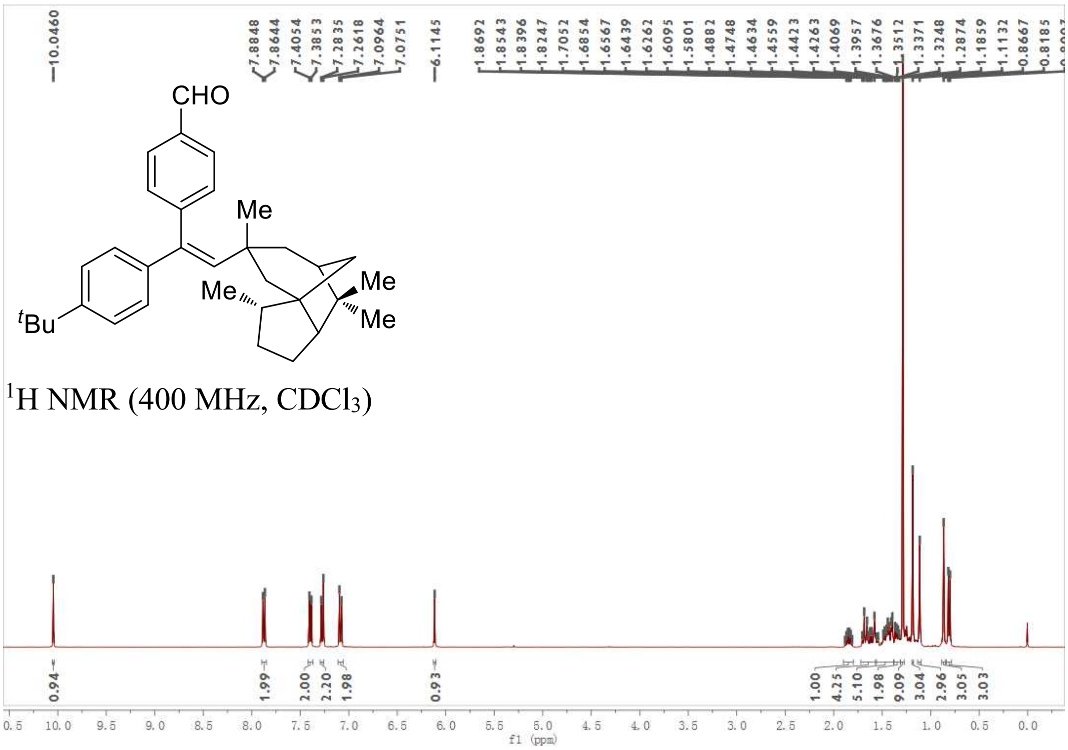

Supplementary Figure 146: ^{1}H NMR Spectra of 4-((Z)-1-(4-(tert-Butyl)phenyl)-2-((3S,3aR,5R,7S)-3,5,8,8-tetramethyloctahydro-1H-3a,7-methanoazulen-5-yl)vinyl)benzaldehyde (44)

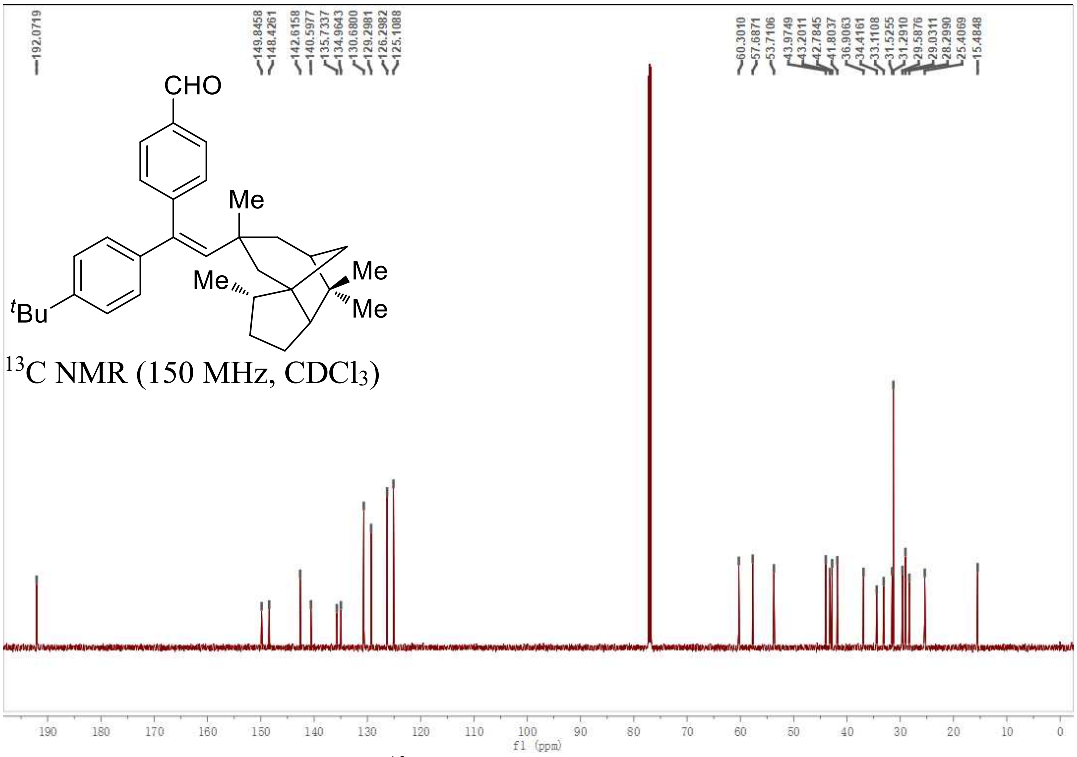

Supplementary Figure 147: ^{13}C NMR Spectra of 4-((Z)-1-(4-(tert-Butyl)phenyl)-2-((3S,3aR,5R,7S)-3,5,8,8-tetramethyloctahydro-1H-3a,7-methanoazulen-5-yl)vinyl)benzaldehyde (44)

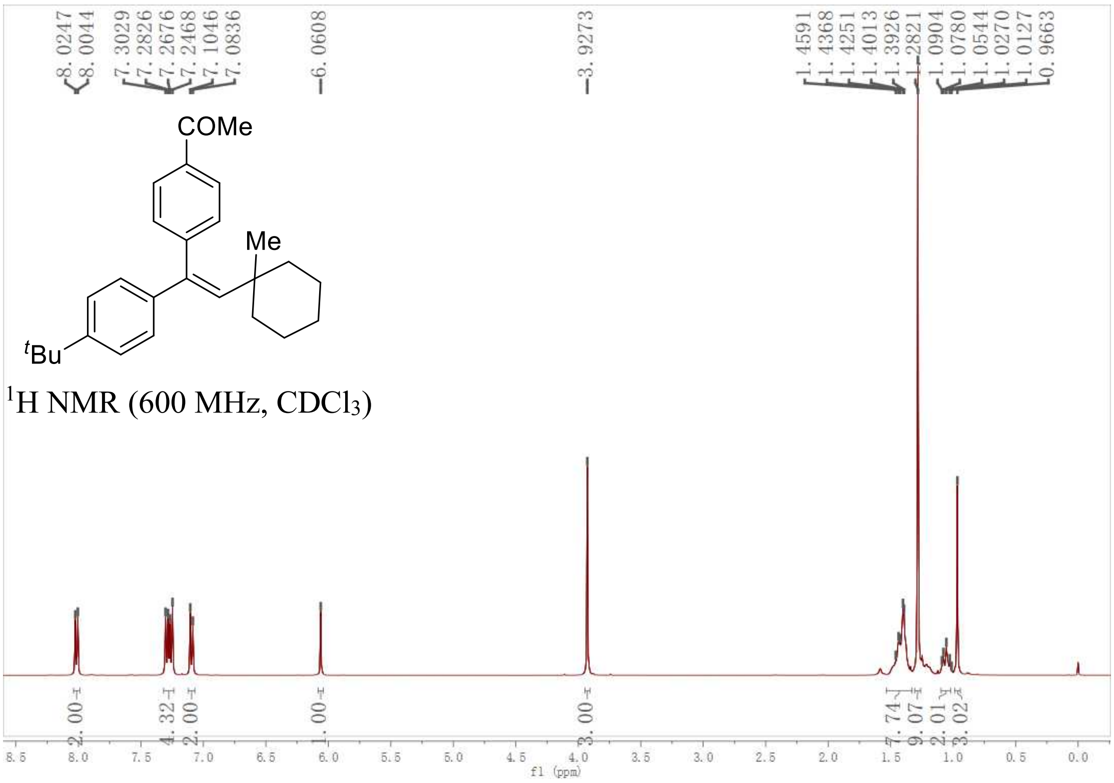

Supplementary Figure 148: ^{1}H NMR Spectra of (Z)-1-(4-(1-(4-(tert-Butyl) phenyl)-2-(1-methylcyclohexyl)vinyl)phenyl)ethenone (45)

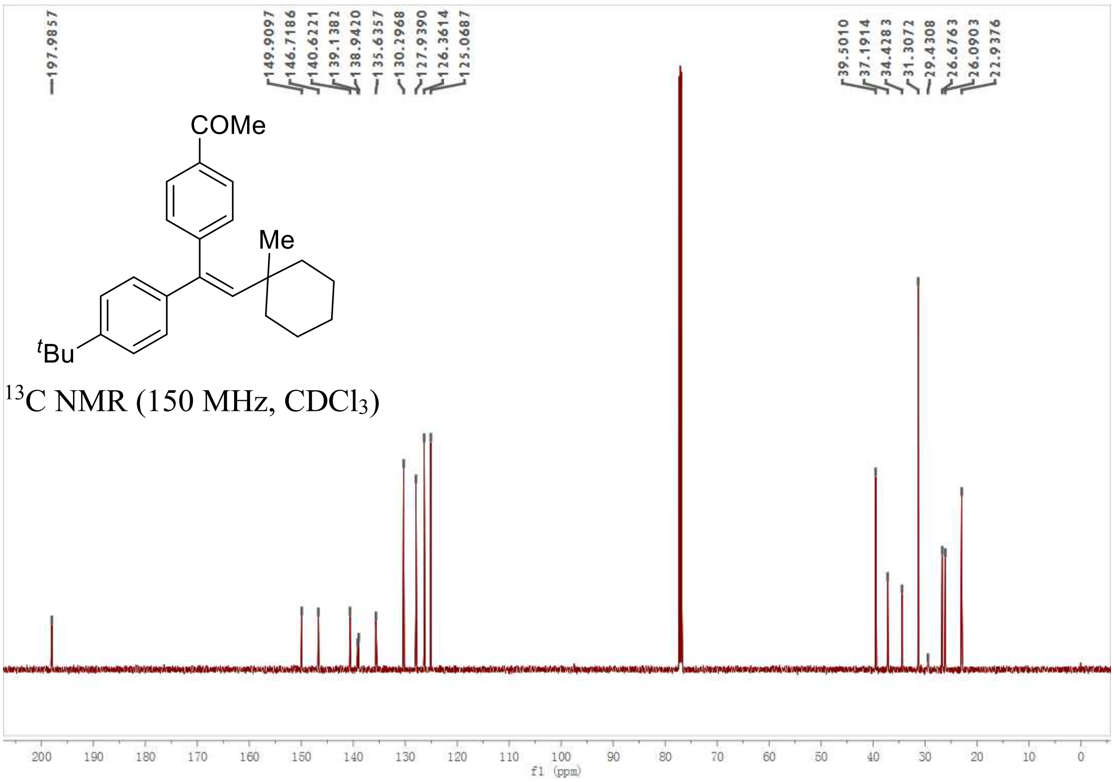

Supplementary Figure 149: ^{13}C NMR Spectra of (Z)-1-(4-(1-(4-(tert-Butyl) phenyl)-2-(1-methylcyclohexyl)vinyl)phenyl)ethenone (45)

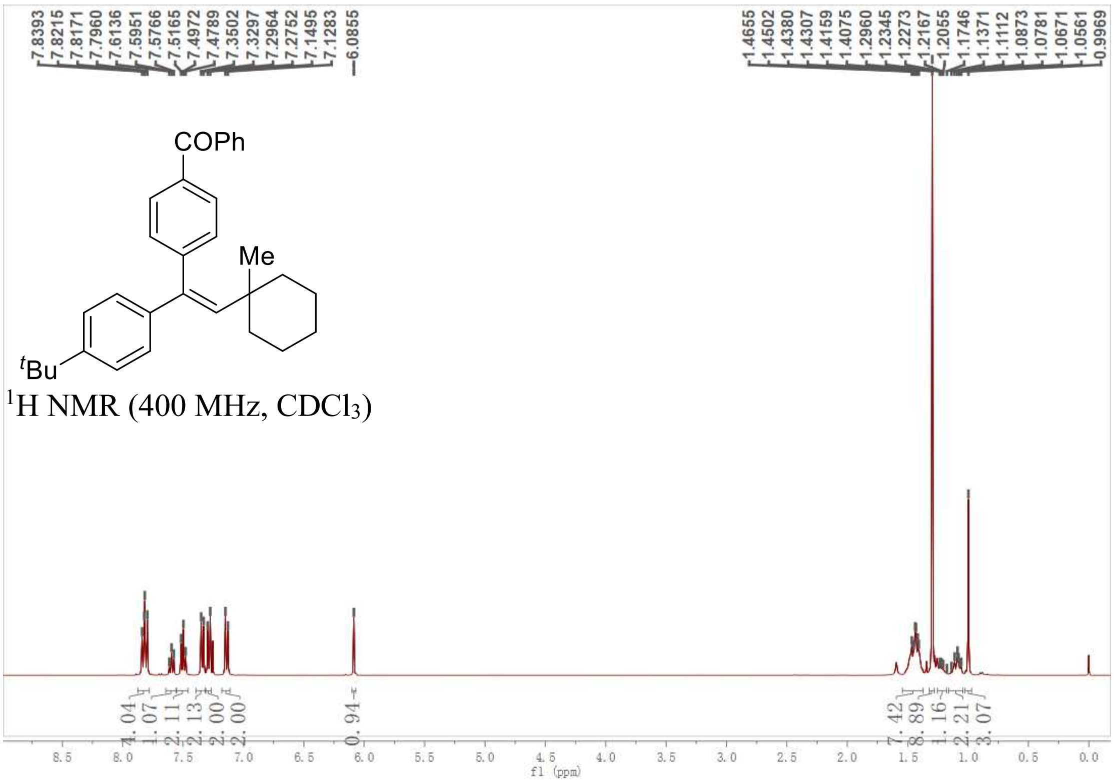

Supplementary Figure 150: ^{1}H NMR Spectra of (Z)-(4-(1-(4-(tert-Butyl) phenyl)-2-(1-methylcyclohexyl)vinyl)phenyl)(phenyl)Methanone (46)

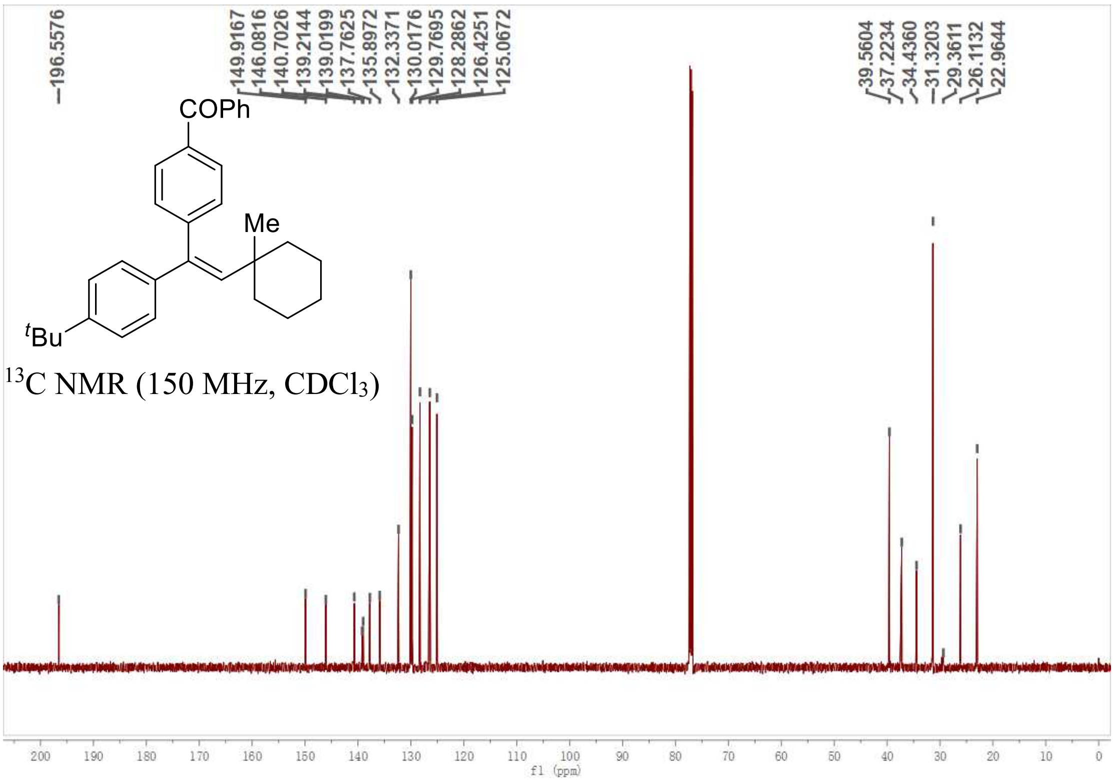

Supplementary Figure 151: ^{13}C NMR Spectra of (Z)-(4-(1-(4-(tert-Butyl) phenyl)-2-(1-methylcyclohexyl)vinyl)phenyl)(phenyl)Methanone (46)

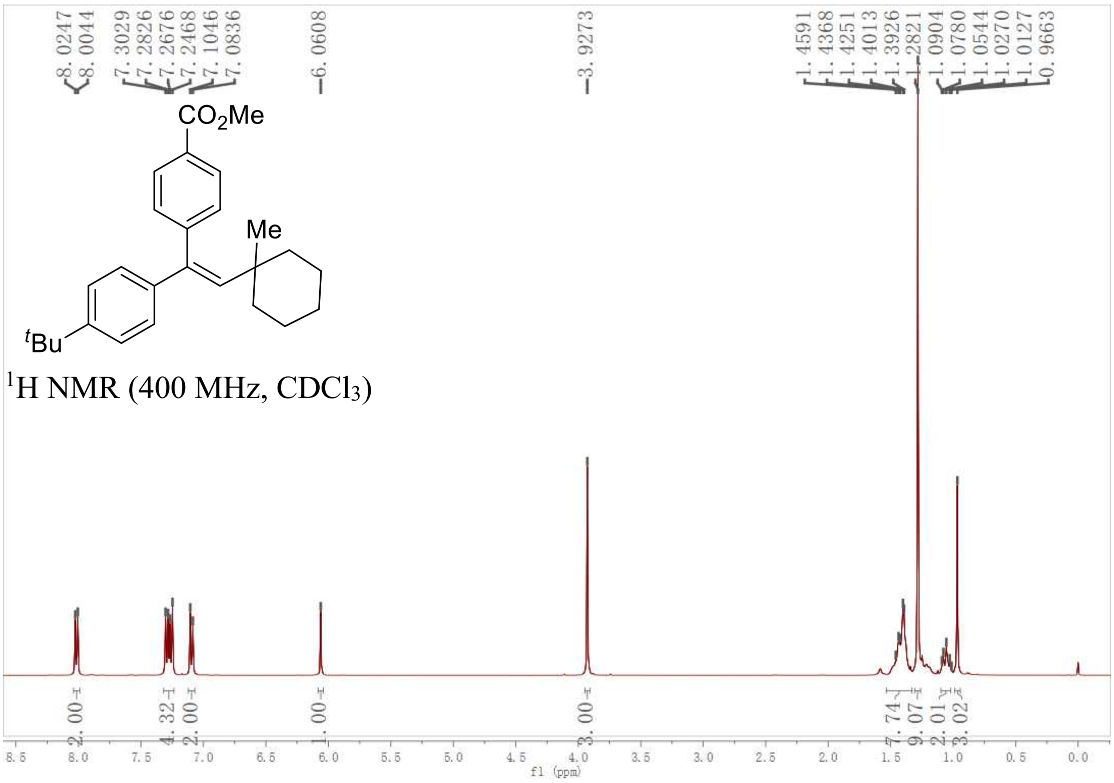

Supplementary Figure 152: ^{1}H NMR Spectra of (Z)-Methyl-4-(1-(4-(tert-butyl) phenyl)-2-(1-methylcyclohexyl)vinyl)benzoate (Z-47)

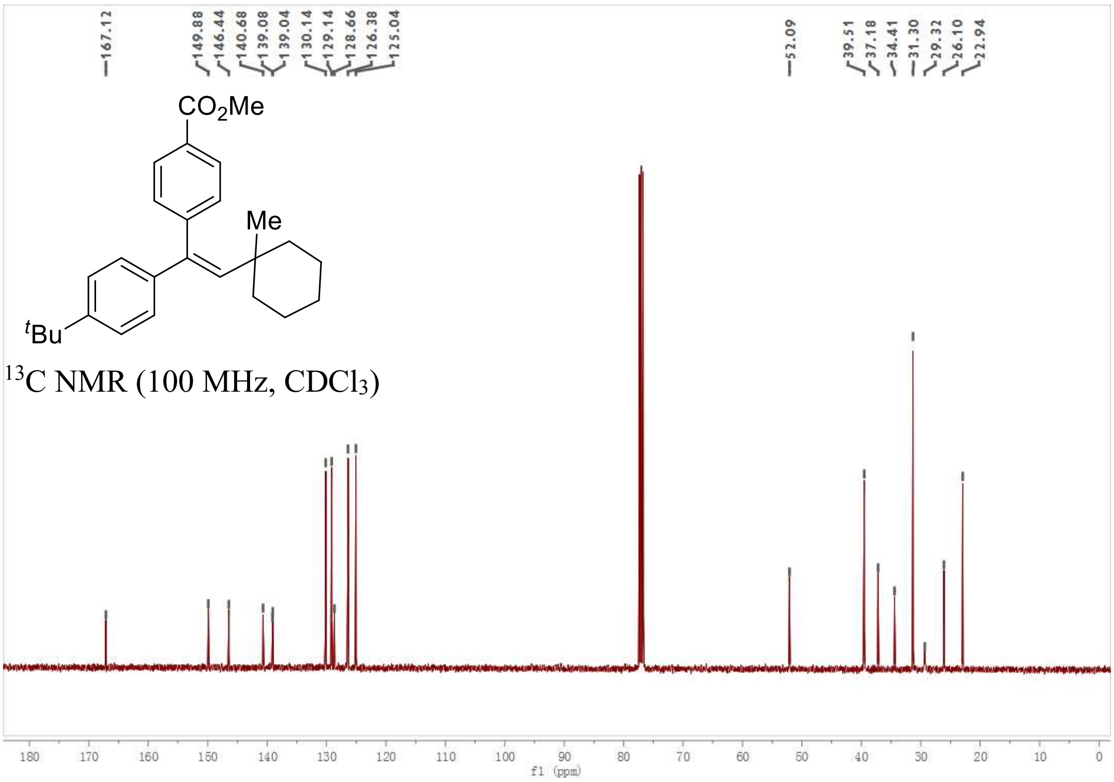

Supplementary Figure 153: ^{13}C NMR Spectra of (Z)-Methyl-4-(1-(4-(tert-butyl) phenyl)-2-(1-methylcyclohexyl)vinyl)benzoate (Z-47)

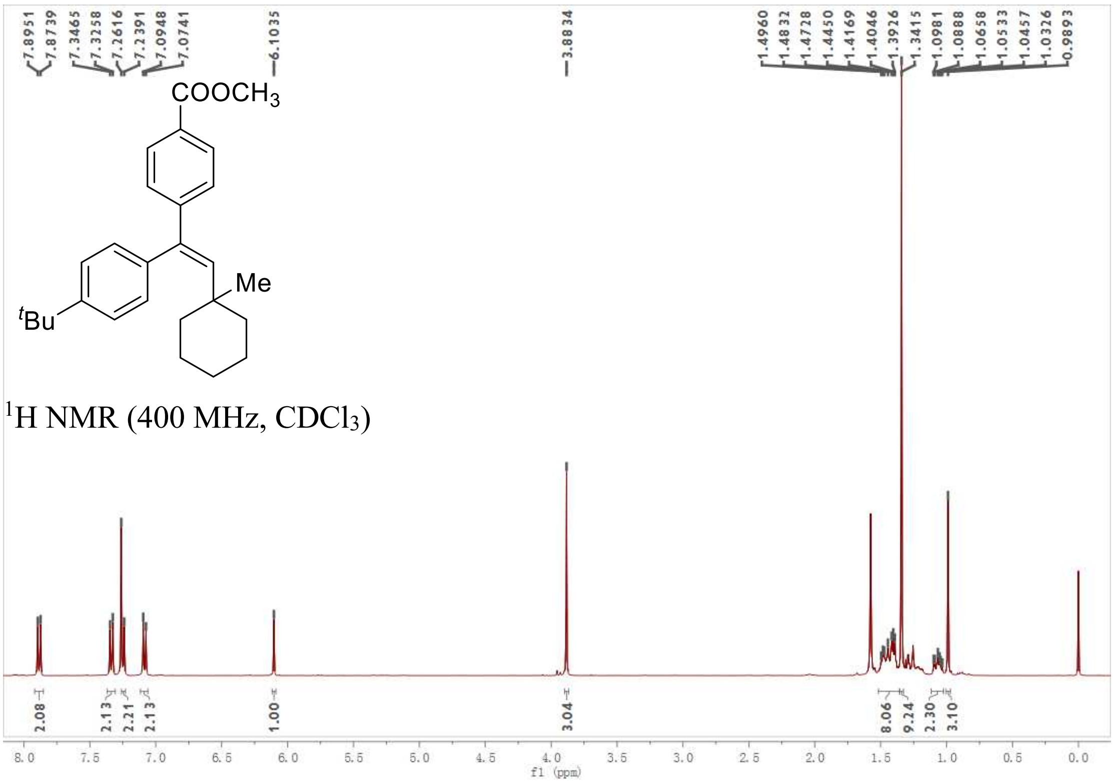

Supplementary Figure 154: ^{1}H NMR Spectra of methyl (E)-4-(1-(4-(tert-butyl) phenyl)-2-(1-methylcyclohexyl)vinyl)benzoate (*E*-47)

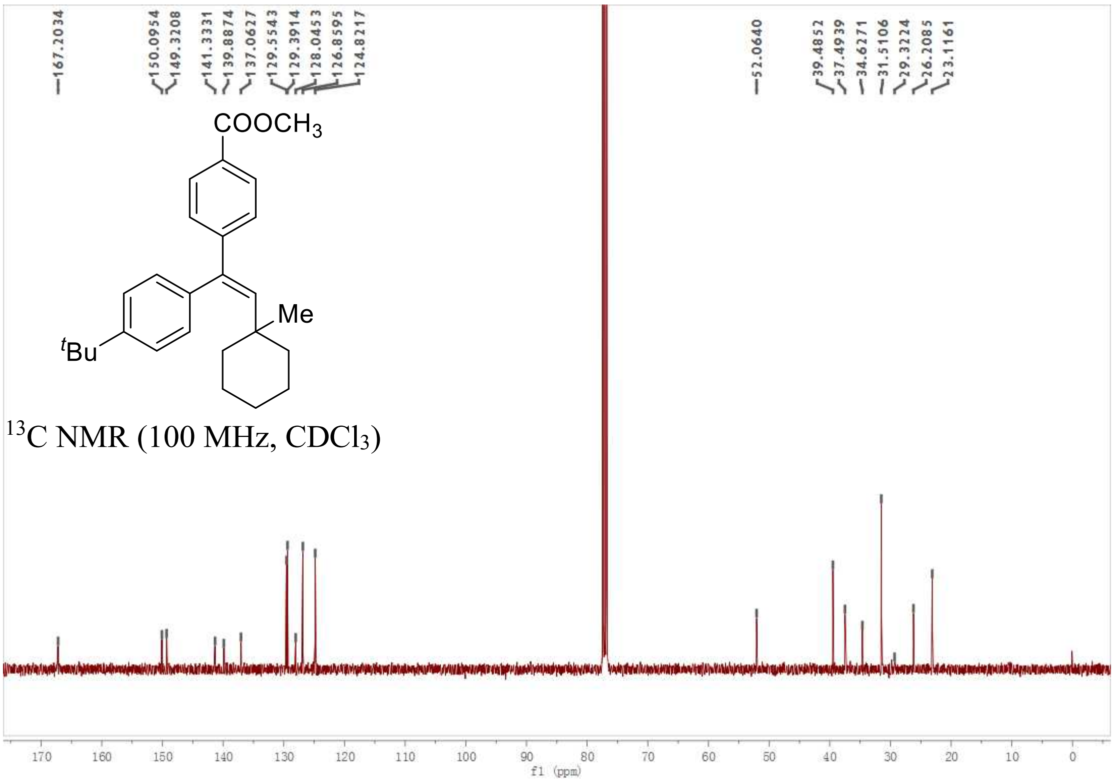

Supplementary Figure 155: ^{13}C NMR Spectra of methyl (E)-4-(1-(4-(tert-butyl) phenyl)-2-(1-methylcyclohexyl)vinyl)benzoate (*E*-47)

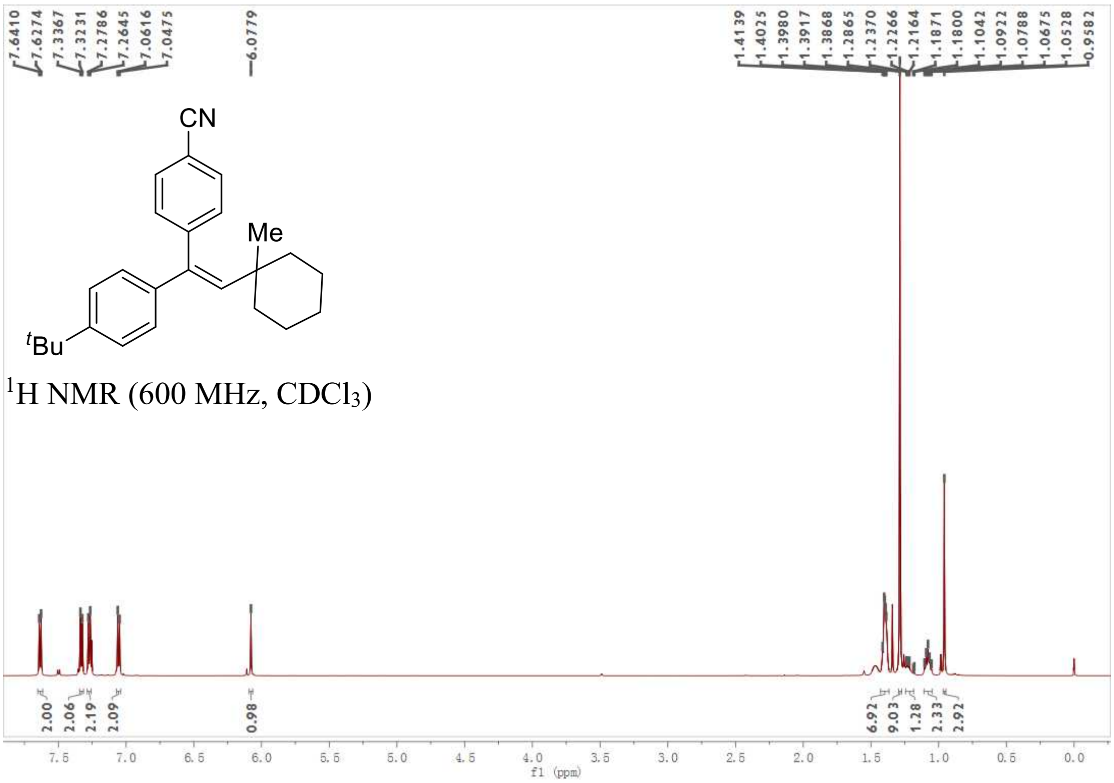

Supplementary Figure 156: ^{1}H NMR Spectra of (Z)-4-(1-(4-(Tert-butyl)phenyl) -2-(1-methylcyclohexyl)vinyl)benzonitrile (48)

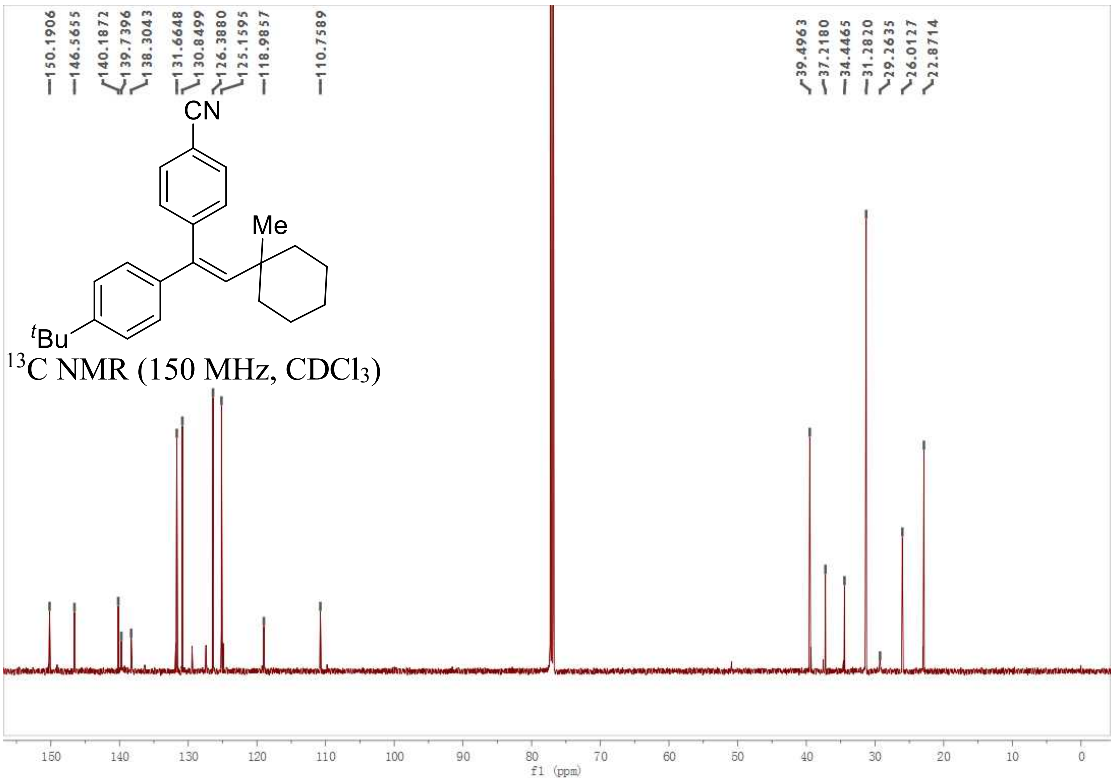

Supplementary Figure 157: ^{13}C NMR Spectra of (Z)-4-(1-(4-(Tert-butyl)phenyl) -2-(1-methylcyclohexyl)vinyl)benzonitrile (48)

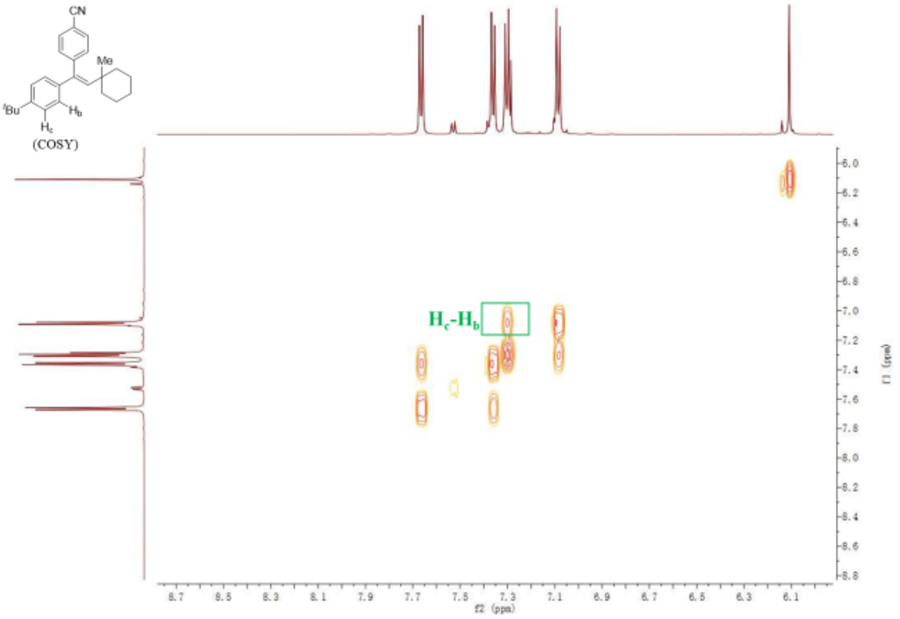

Supplementary Figure 158: COSY Spectra of (Z)-4-(1-(4-(Tert-butyl)phenyl) -2-(1-methylcyclohexyl)vinyl)benzonitrile (48)

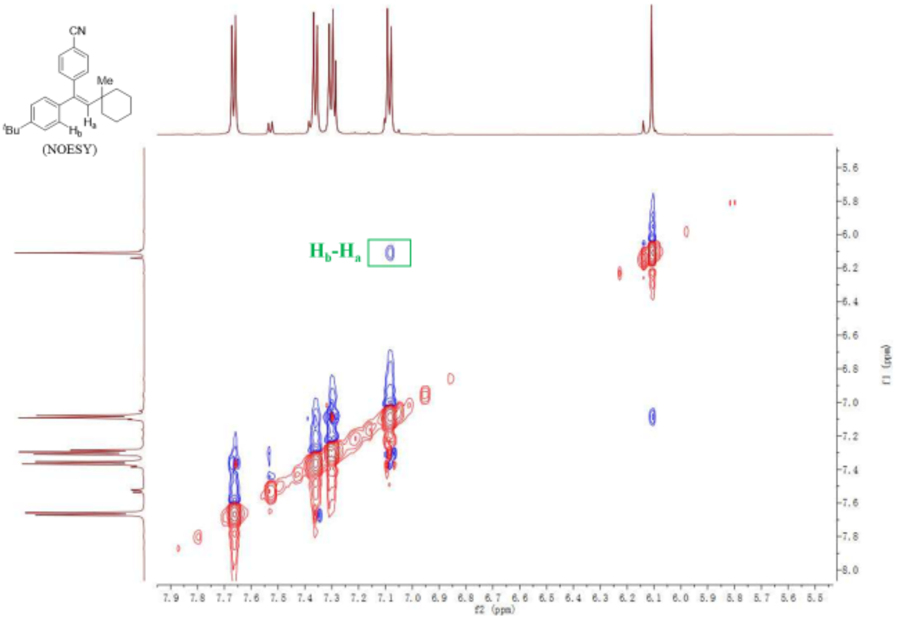

Supplementary Figure 159: NOESY Spectra of (Z)-4-(1-(4-(Tert-butyl)phenyl) -2-(1-methylcyclohexyl)vinyl)benzonitrile (48)

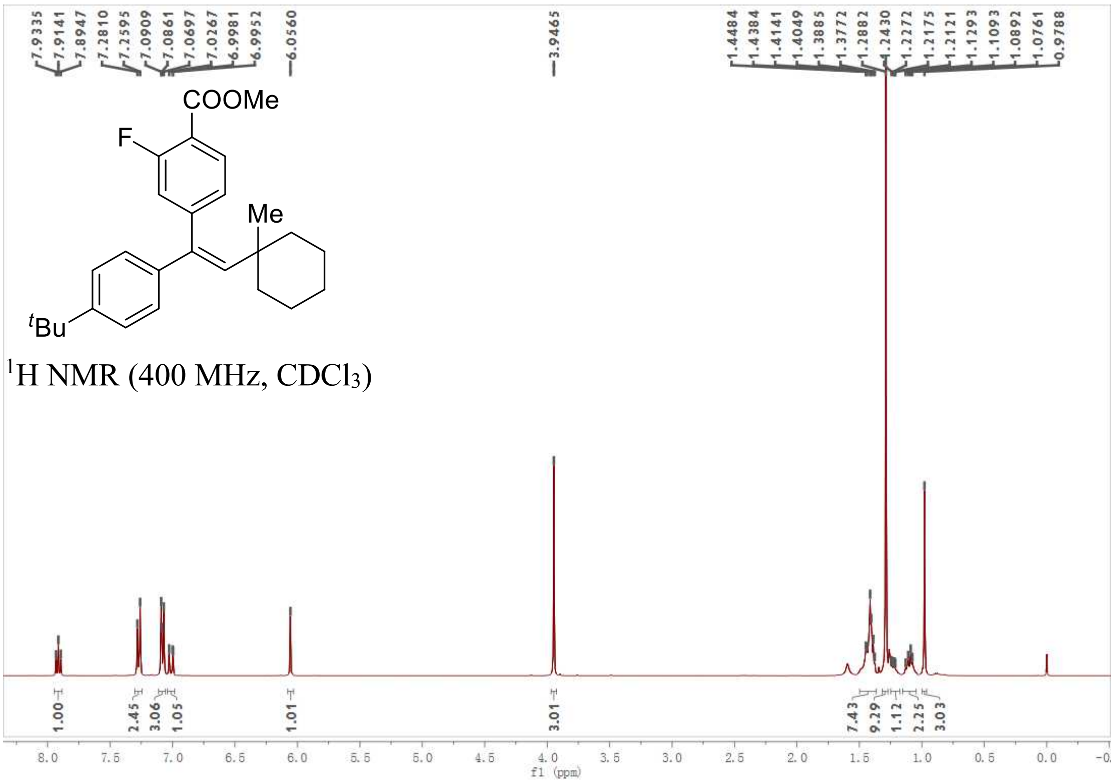

Supplementary Figure 160: ^{1}H NMR Spectra of (Z)-Methyl4-(1-(4-(tert-butyl)phenyl)-2-(1-methylcyclohexyl)vinyl)-2-fluorobenzoate (Z-49)

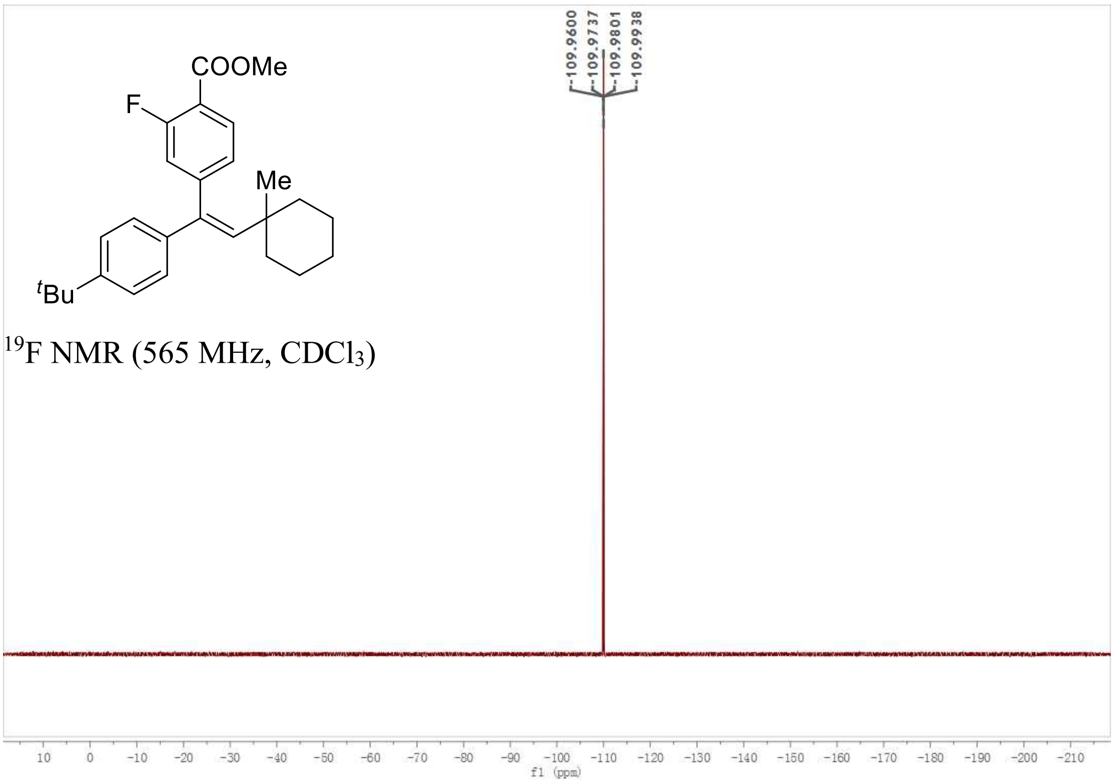

Supplementary Figure 161: ^{19}F NMR Spectra of (Z)-Methyl4-(1-(4-(tert-butyl) phenyl)-2-(1-methylcyclohexyl)vinyl)-2-fluorobenzoate (Z-49)

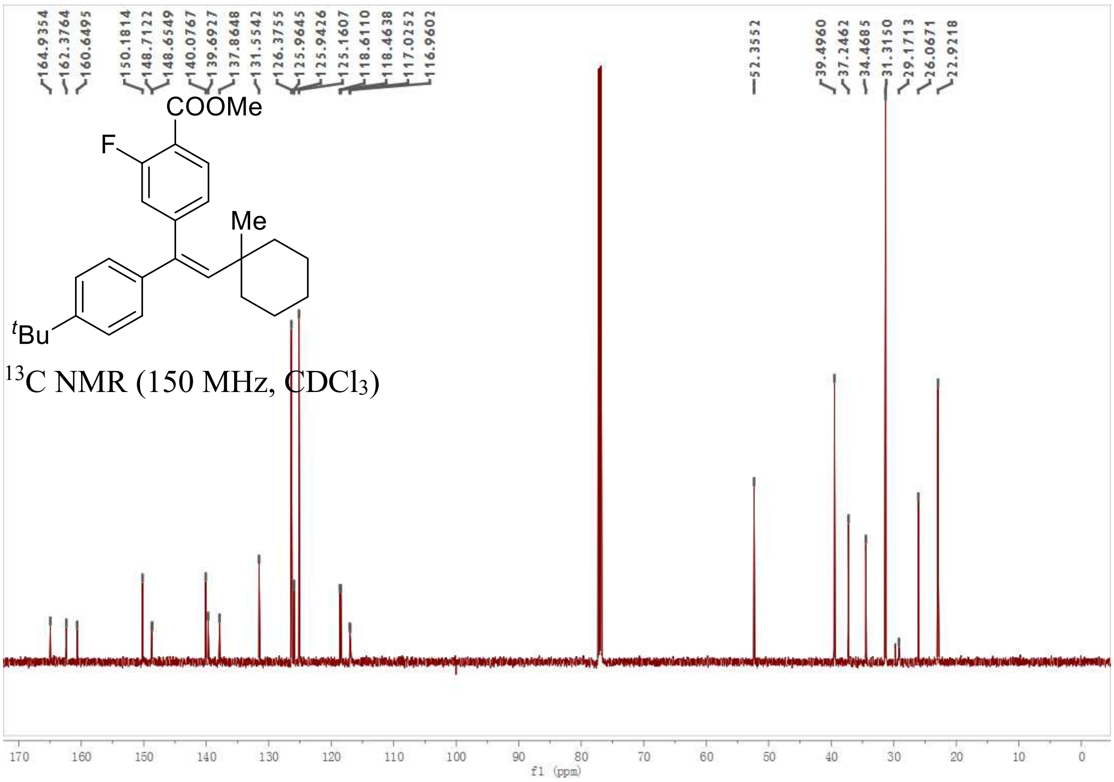

Supplementary Figure 162: ^{13}C NMR Spectra of (Z)-Methyl4-(1-(4-(tert-butyl) phenyl)-2-(1-methylcyclohexyl)vinyl)-2-fluorobenzoate (Z-49)

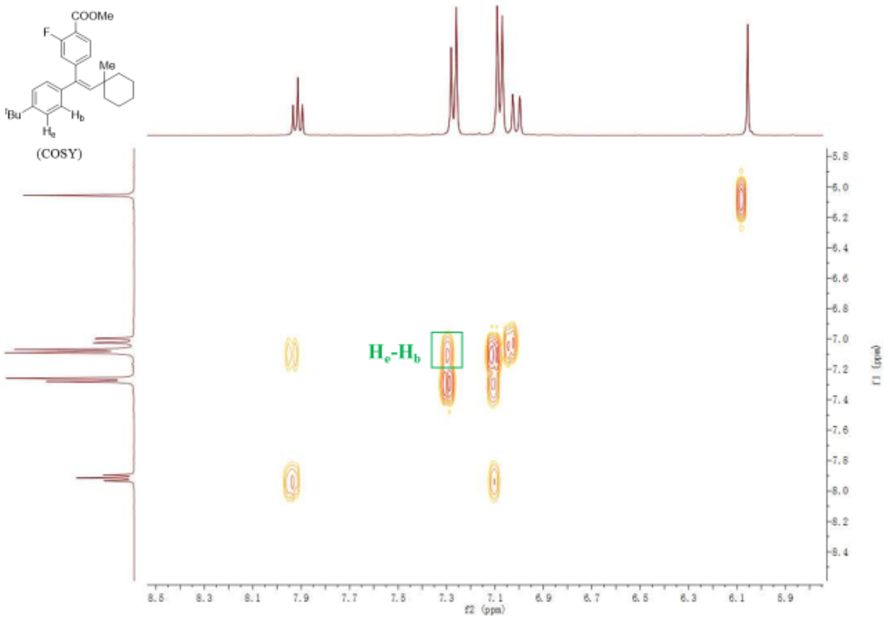

Supplementary Figure 163: COSY Spectra of (Z)-Methyl4-(1-(4-(tert-butyl) phenyl)-2-(1-methylcyclohexyl)vinyl)-2-fluorobenzoate (Z-49)

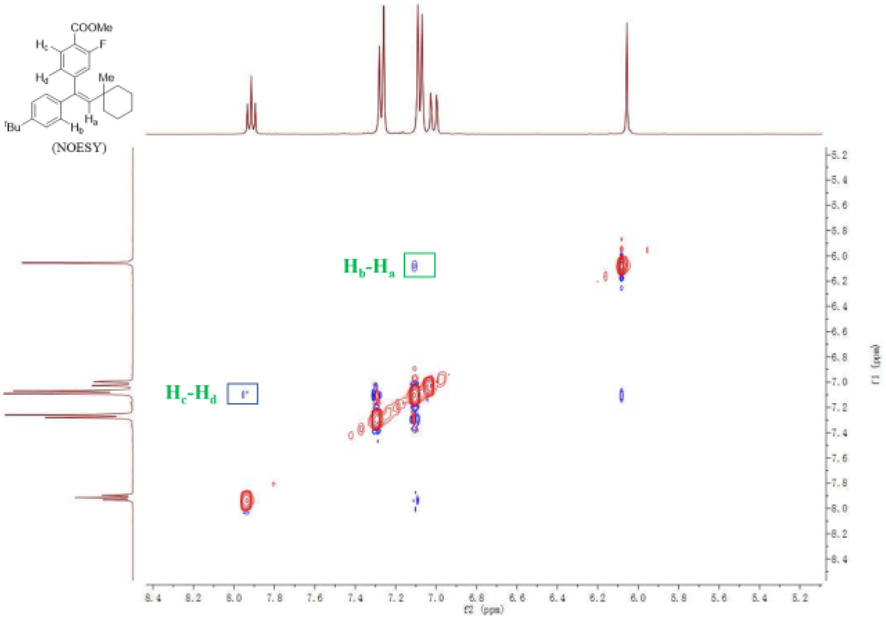

Supplementary Figure 164: NOESY Spectra of (Z)-Methyl4-(1-(4-(tert-butyl) phenyl)-2-(1-methylcyclohexyl)vinyl)-2-fluorobenzoate (Z-49)

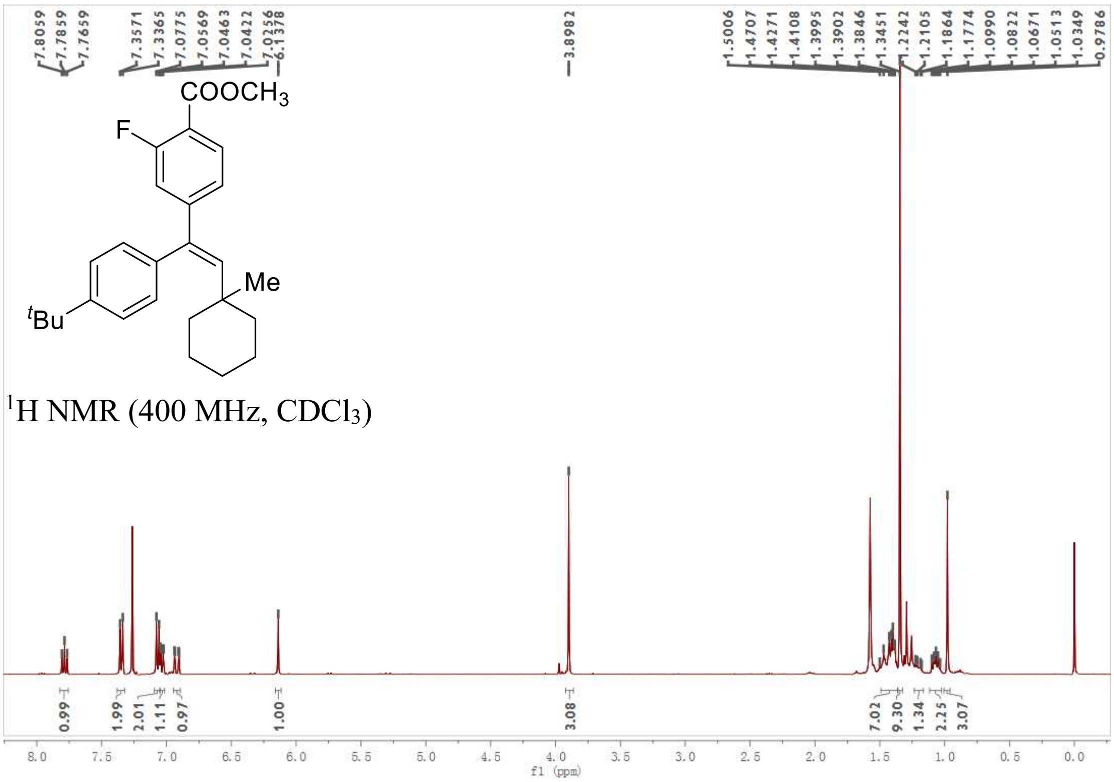

Supplementary Figure 165: ^{1}H NMR Spectra of methyl (E)-4-(1-(4-(tert-butyl) phenyl)-2-(1-methylcyclohexyl)vinyl)-2-fluorobenzoate (*E*-49)

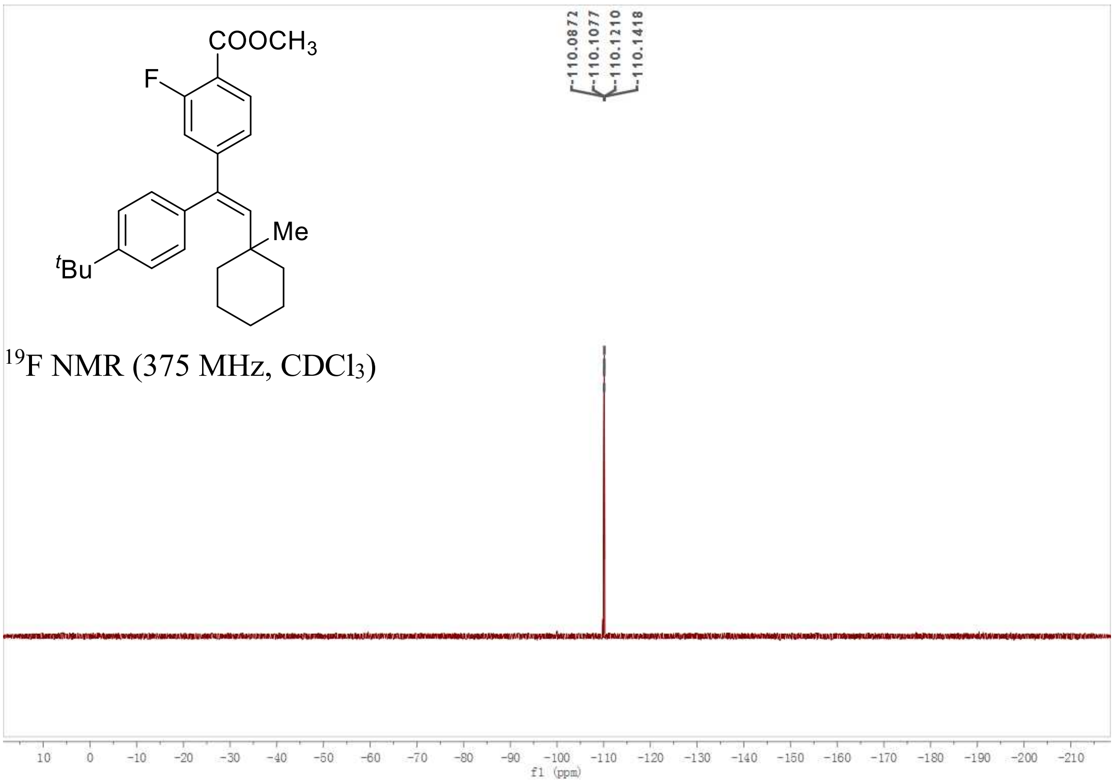

Supplementary Figure 166: ^{19}F NMR Spectra of methyl (E)-4-(1-(4-(tert-butyl) phenyl)-2-(1-methylcyclohexyl)vinyl)-2-fluorobenzoate (*E*-49)

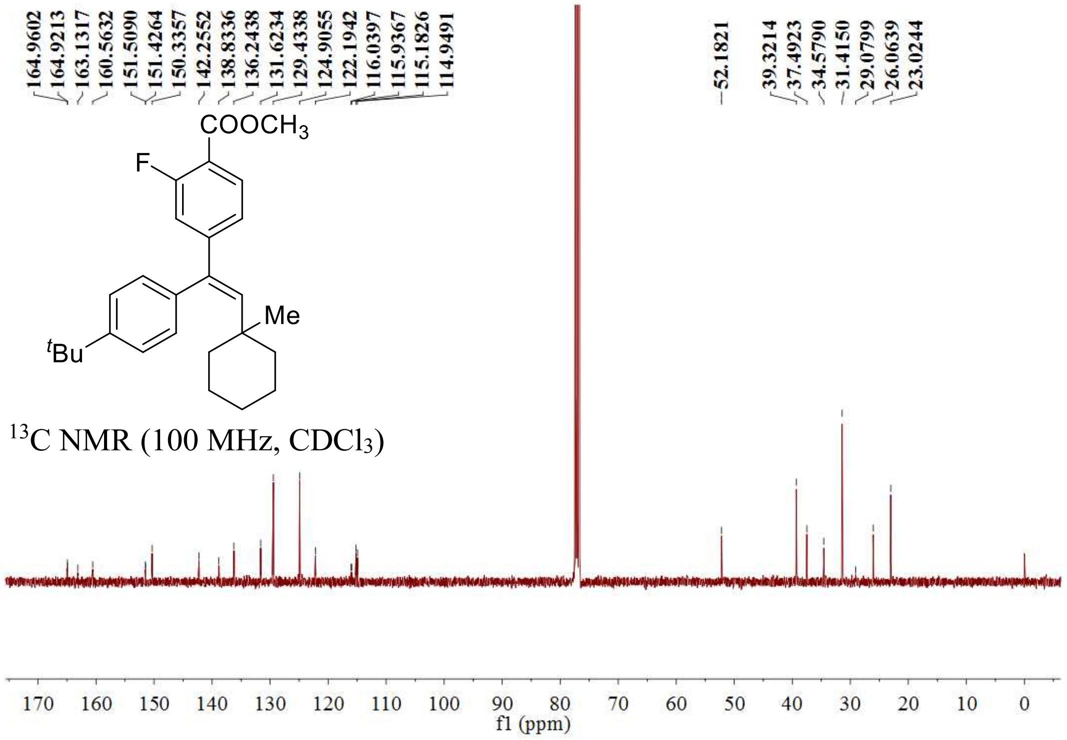

Supplementary Figure 167: ^{13}C NMR Spectra of methyl (E)-4-(1-(4-(tert-butyl) phenyl)-2-(1-methylcyclohexyl)vinyl)-2-fluorobenzoate (*E*-49)

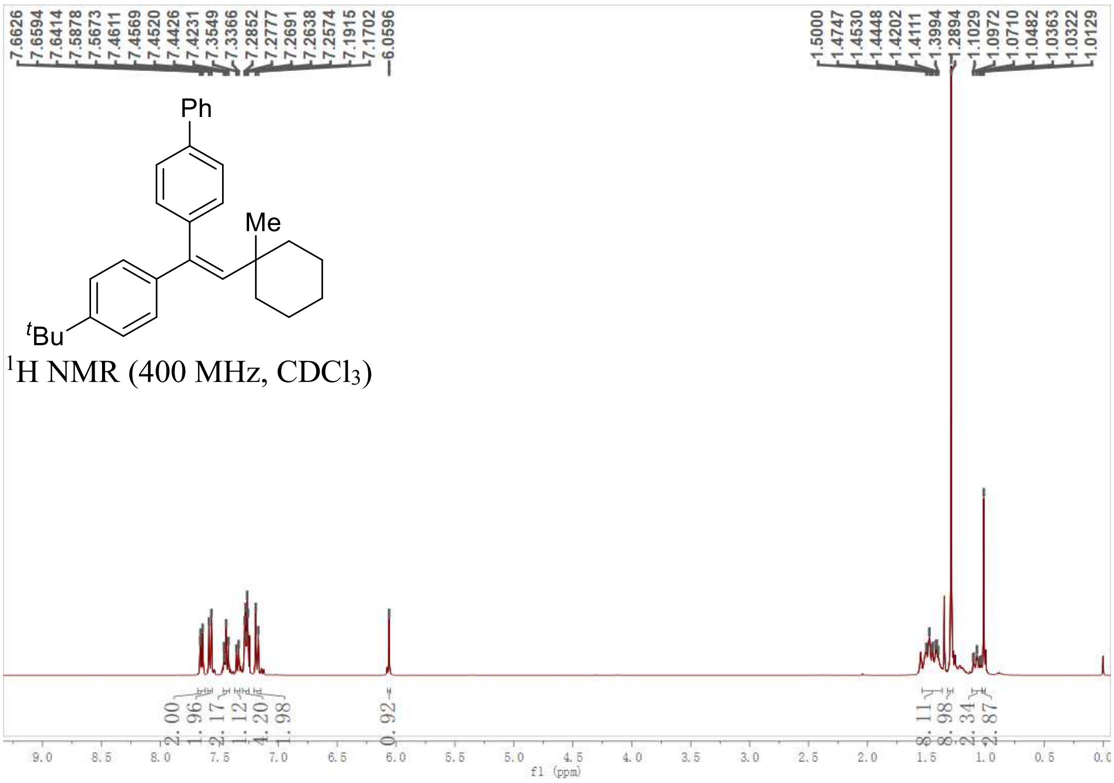

Supplementary Figure 168: ^{1}H NMR Spectra of (Z)-4-(1-(4-(Tert-Butyl) phenyl)-2-(1-methylcyclohexyl)vinyl)-1,1'-biphenyl (50)

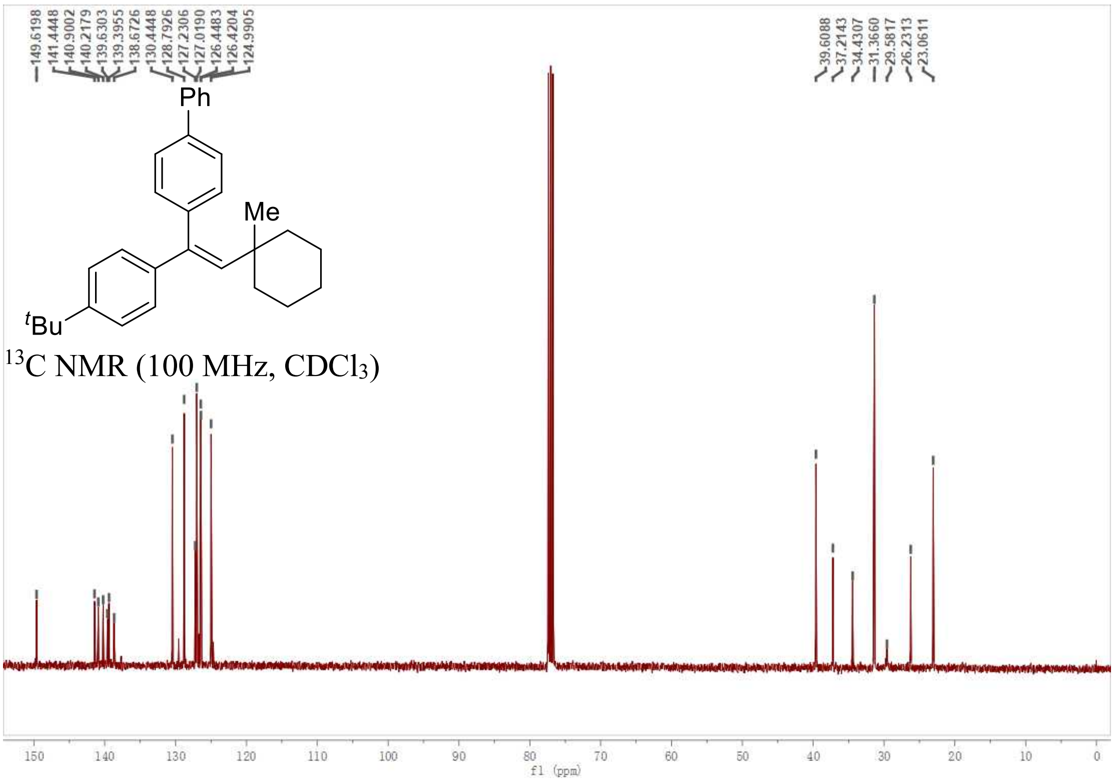

Supplementary Figure 169: ^{13}C NMR Spectra of (Z)-4-(1-(4-(Tert-Butyl) phenyl)-2-(1-methylcyclohexyl)vinyl)-1,1'-biphenyl (50)

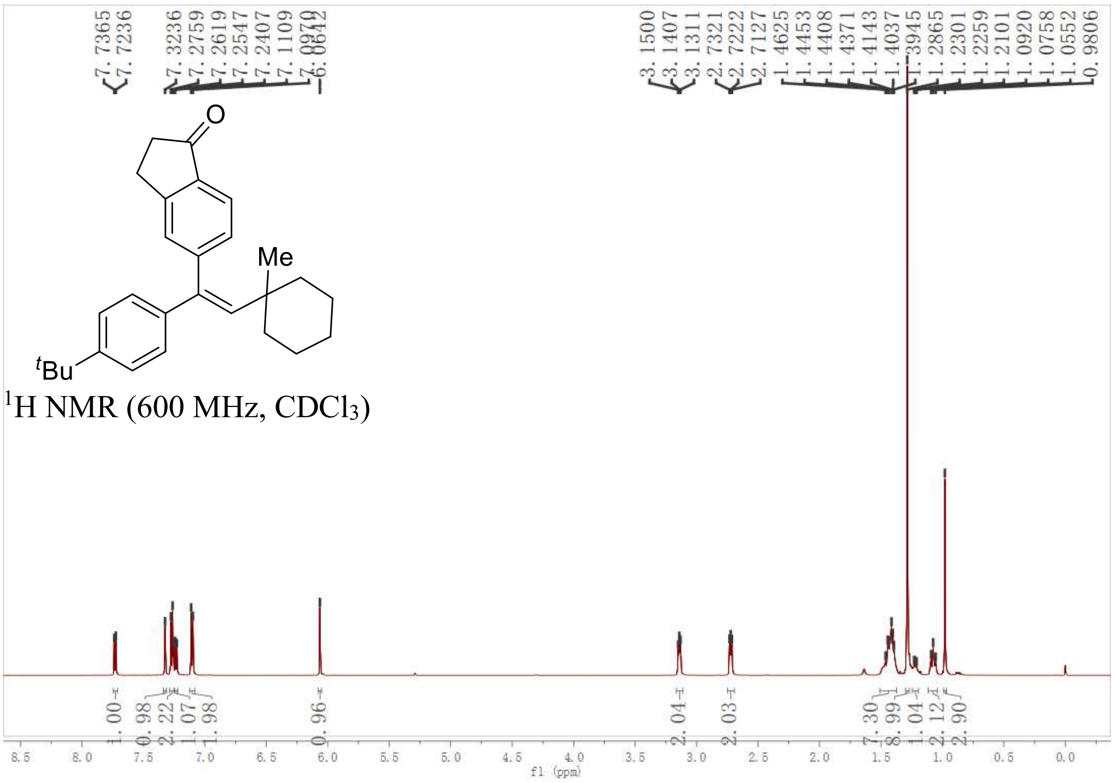

Supplementary Figure 170: ^{1}H NMR Spectra of (Z)-5-(1-(4-(tert-Butyl) phenyl)-2-(1-methylcyclohexyl)vinyl)-2,3-dihydro-1H-inden-1-one (51)

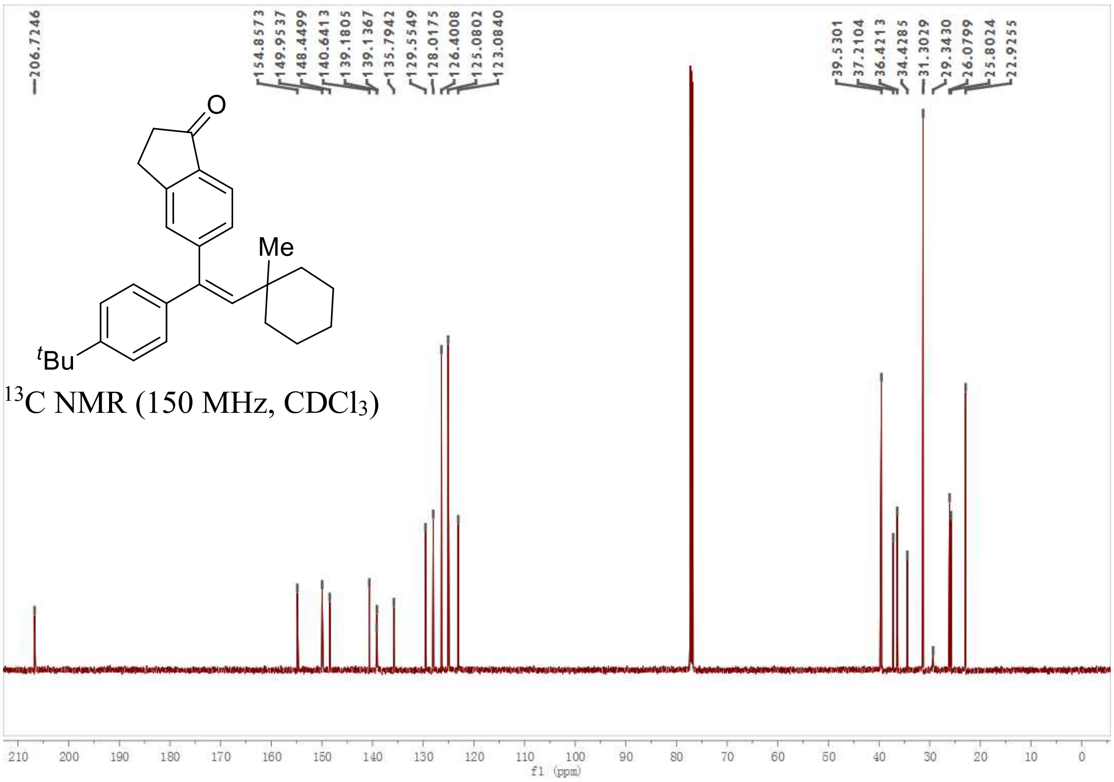

Supplementary Figure 171: ^{13}C NMR Spectra of (Z)-5-(1-(4-(tert-Butyl) phenyl)-2-(1-methylcyclohexyl)vinyl)-2,3-dihydro-1H-inden-1-one (51)

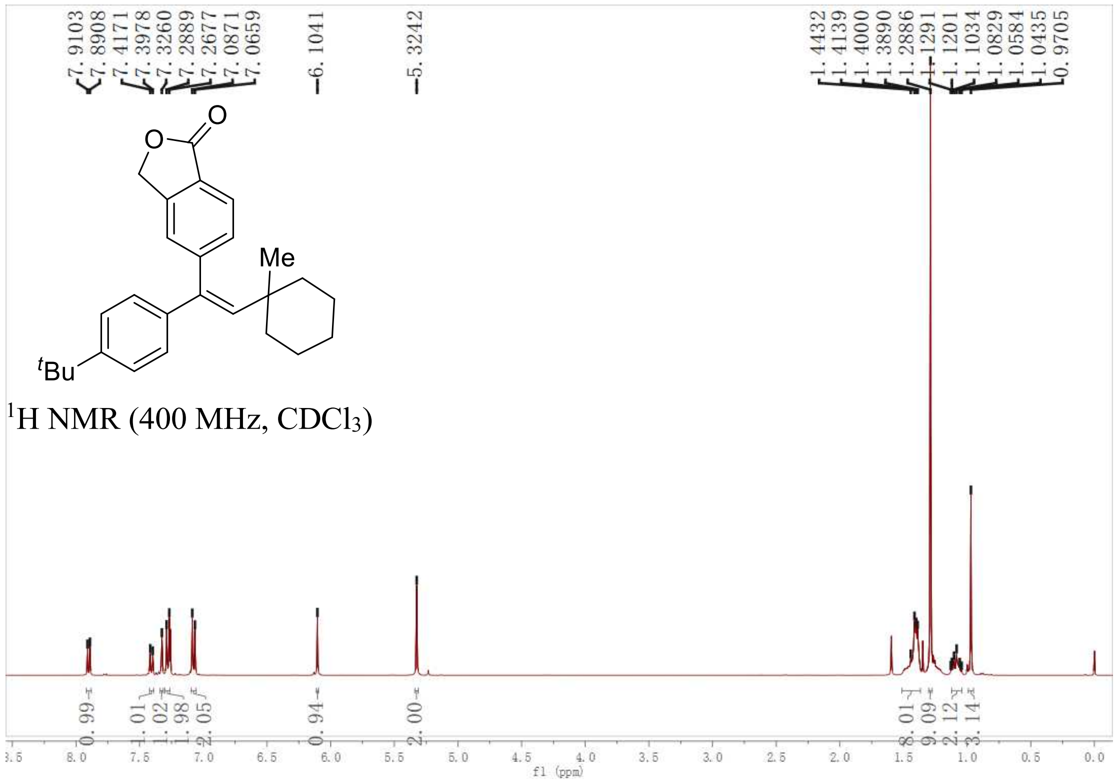

Supplementary Figure 172: ^{1}H NMR Spectra of (Z)-5-(1-(4-(tert-Butyl) phenyl)-2-(1-methylcyclohexyl)vinyl)isobenzofuran-1(3H)-one (Z-52)

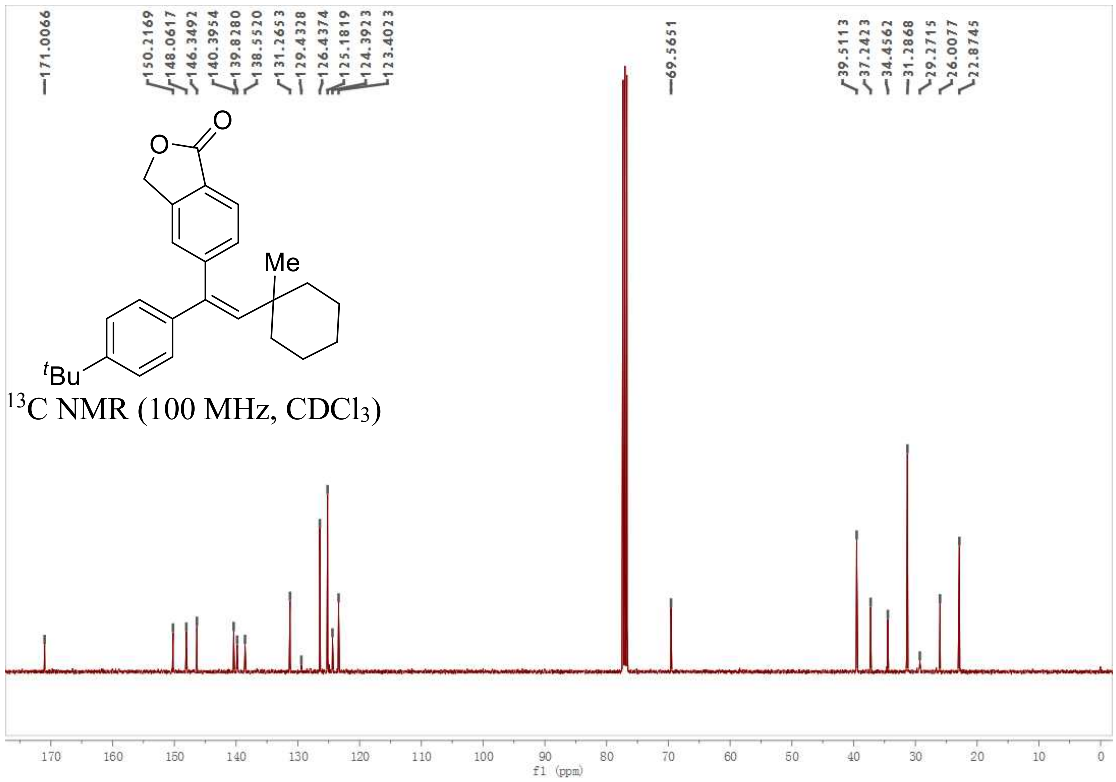

Supplementary Figure 173: ^{13}C NMR Spectra of (Z)-5-(1-(4-(tert-Butyl) phenyl)-2-(1-methylcyclohexyl)vinyl)isobenzofuran-1(3H)-one (Z-52)

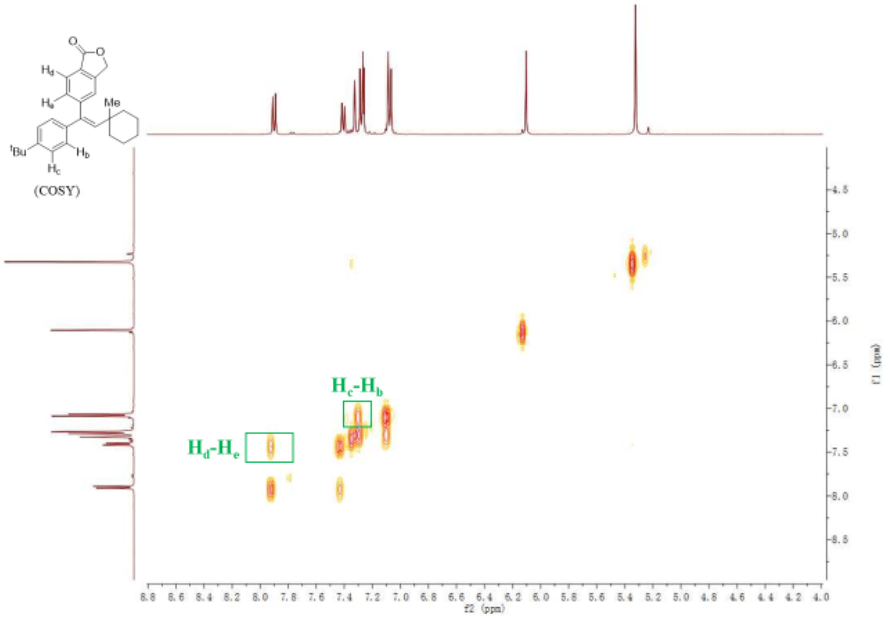

Supplementary Figure 174: COSY Spectra of (Z)-5-(1-(4-(tert-Butyl) phenyl)-2-(1-methylcyclohexyl)vinyl)isobenzofuran-1(3H)-one (Z-52)

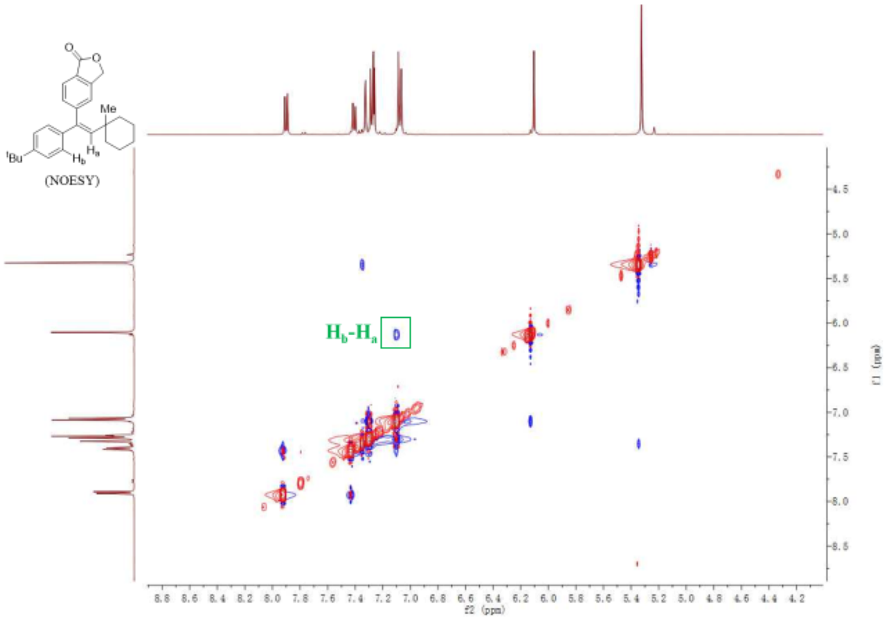

Supplementary Figure 175: NOESY Spectra of (Z)-5-(1-(4-(tert-Butyl) phenyl)-2-(1-methylcyclohexyl)vinyl)isobenzofuran-1(3H)-one (Z-52)

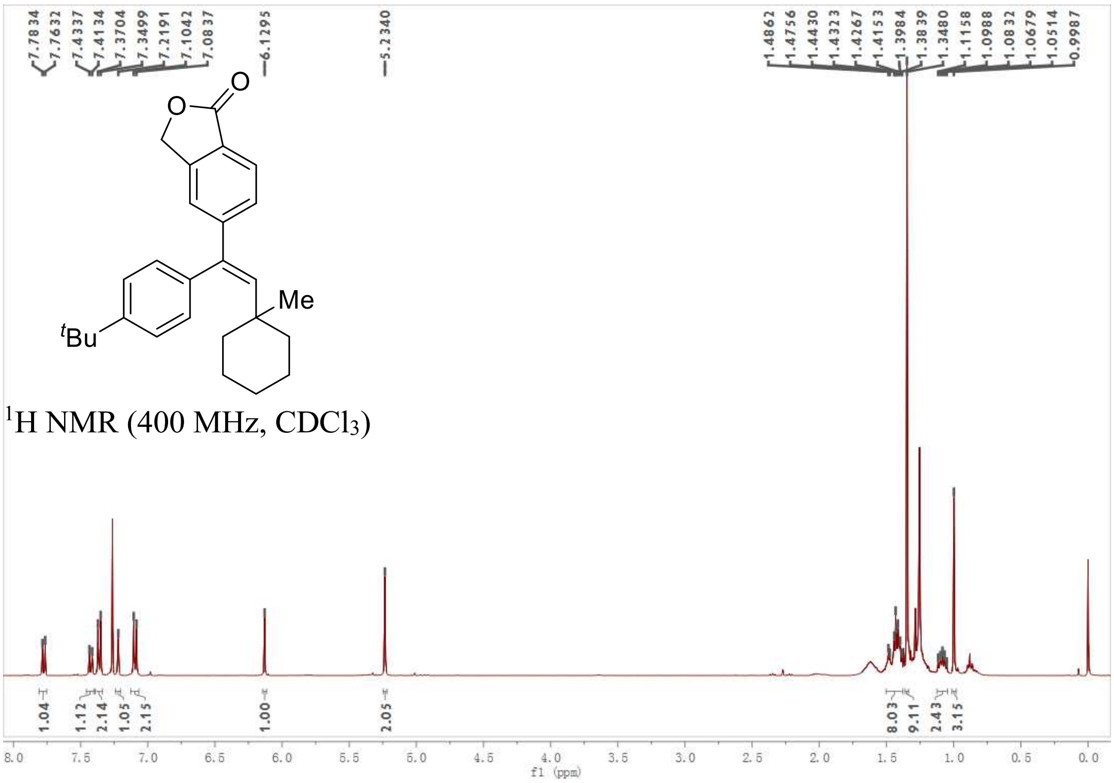

Supplementary Figure 176: ^{1}H NMR Spectra of (*E*)-5-(1-(4-(tert-butyl) phenyl)-2-(1-methylcyclohexyl)vinyl)isobenzofuran-1(3H)-one (*E*-52)

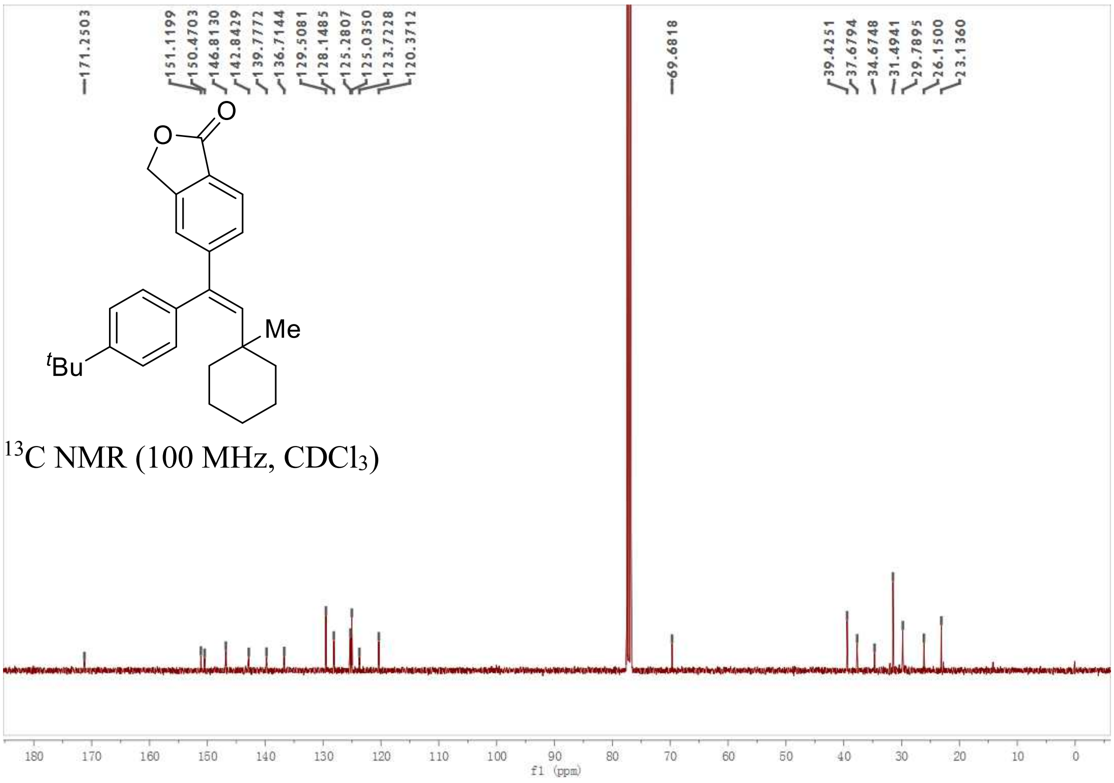

Supplementary Figure 177: ^{1}H NMR Spectra of (*E*)-5-(1-(4-(tert-butyl) phenyl)-2-(1-methylcyclohexyl)vinyl)isobenzofuran-1(3H)-one (*E*-52)

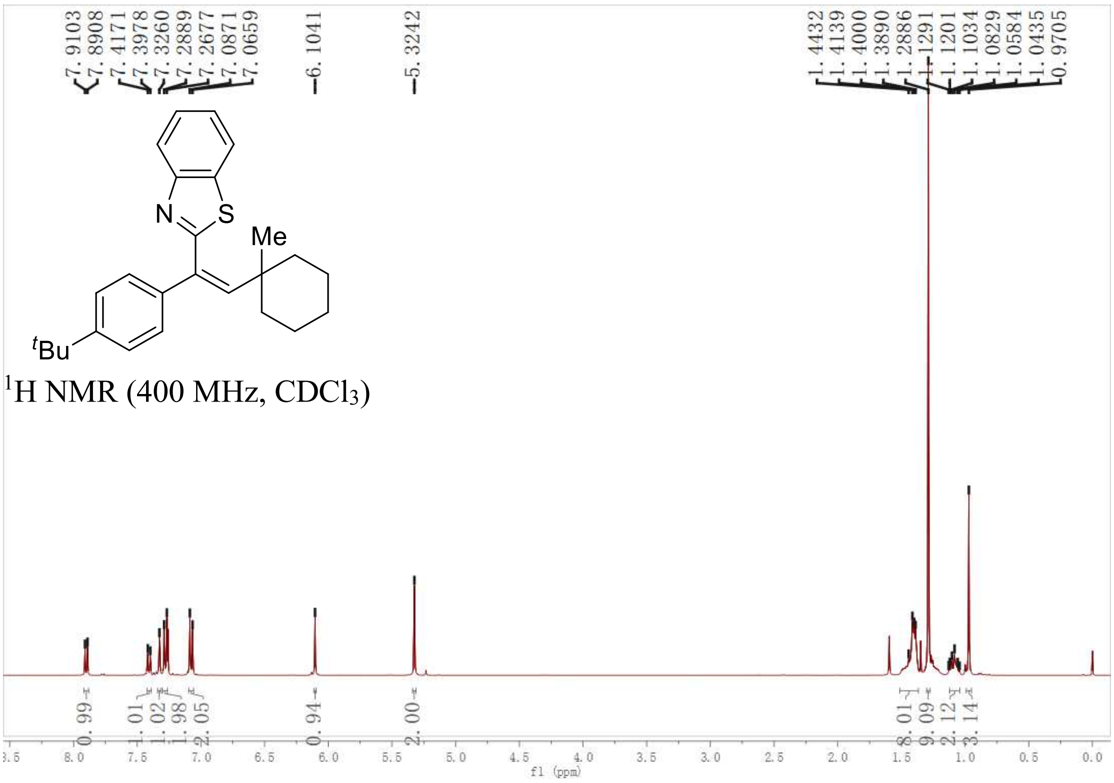

Supplementary Figure 178: ^{1}H NMR Spectra of (Z)-2-(1-(4-(tert-Butyl) phenyl)-2-(1-methylcyclohexyl)vinyl)benzo[d]thiazole (53)

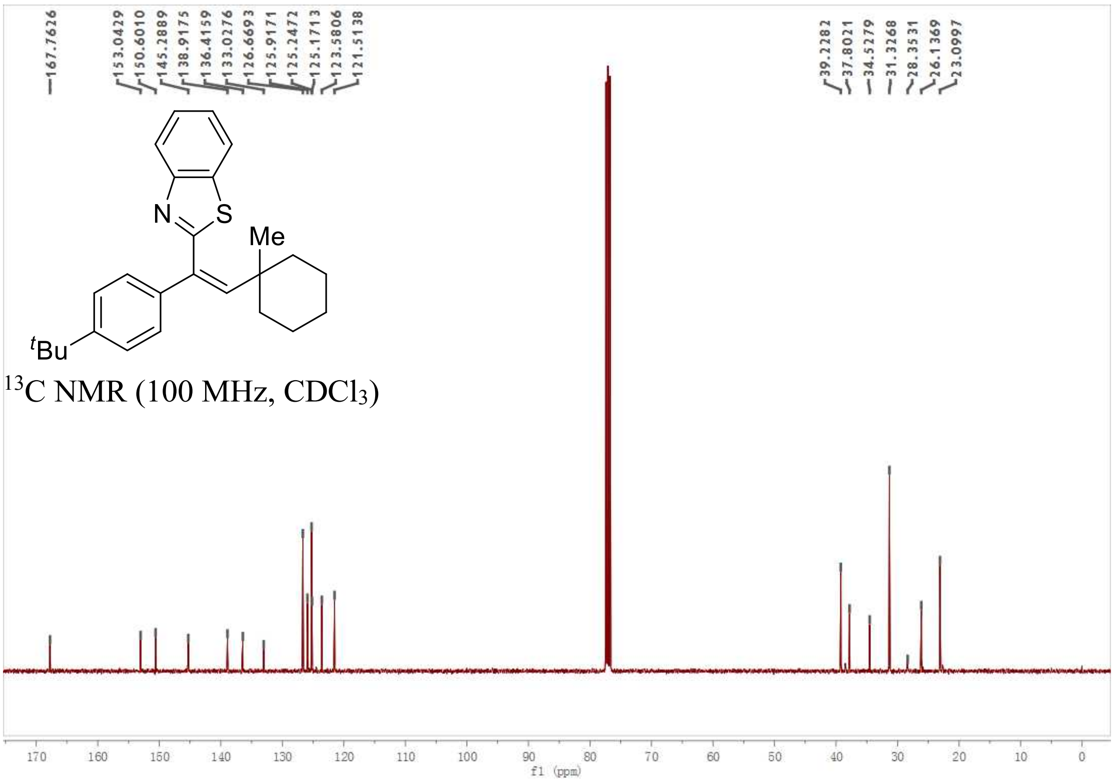

Supplementary Figure 179: ^{13}C NMR Spectra of (Z)-2-(1-(4-(tert-Butyl) phenyl)-2-(1-methylcyclohexyl)vinyl)benzo[d]thiazole (53)

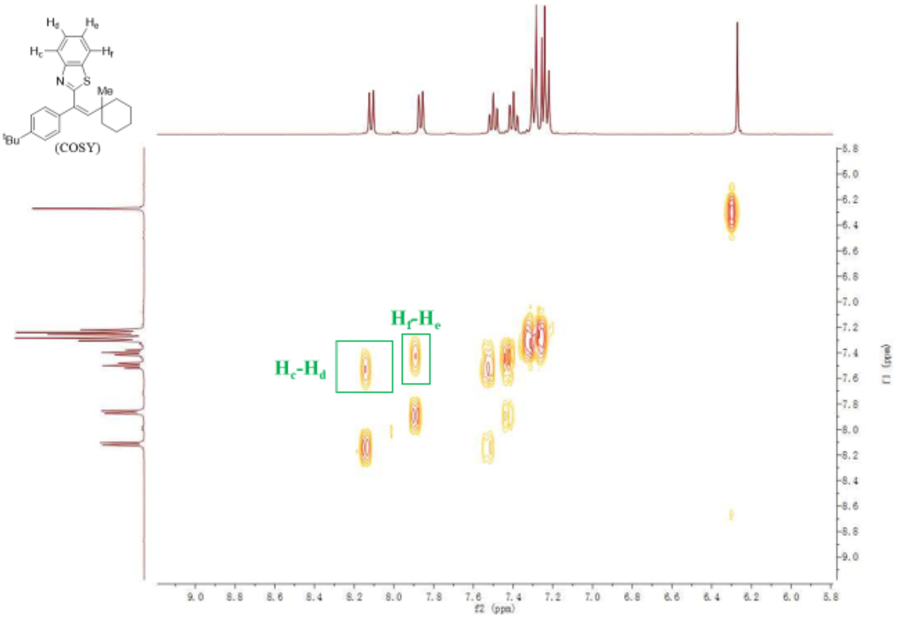

Supplementary Figure 180: COSY Spectra of (Z)-2-(1-(4-(tert-Butyl) phenyl)-2-(1-methylcyclohexyl)vinyl)benzo[d]thiazole (53)

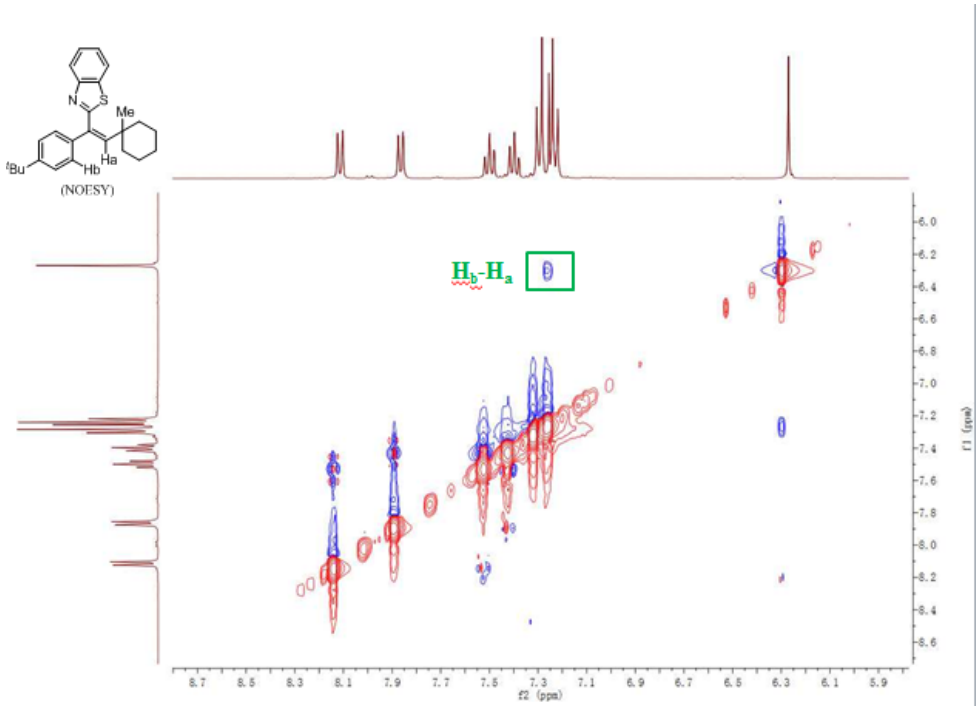

Supplementary Figure 181: NOESY Spectra of (Z)-2-(1-(4-(tert-Butyl) phenyl)-2-(1-methylcyclohexyl)vinyl)benzo[d]thiazole (53)

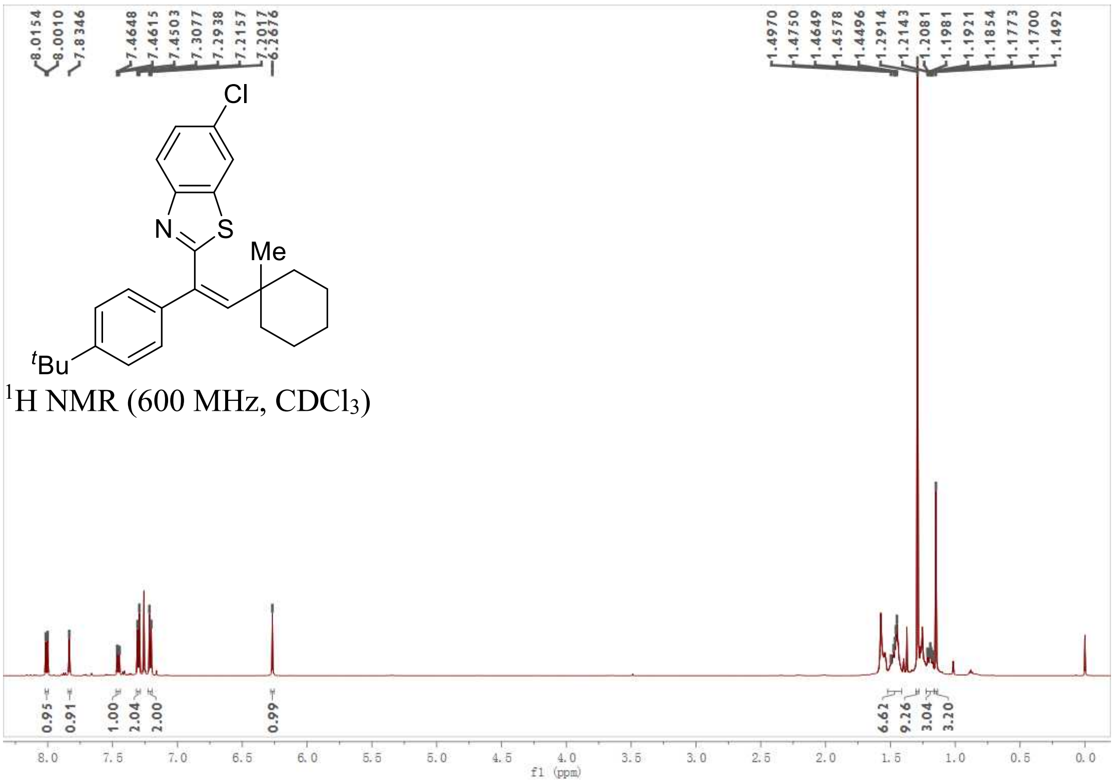

Supplementary Figure 182: ^{1}H NMR Spectra of (Z)-2-(1-(4-(tert-Butyl) phenyl)-2-(1-methylcyclohexyl)vinyl)-6-chlorobenzo[d] thiazole (54)

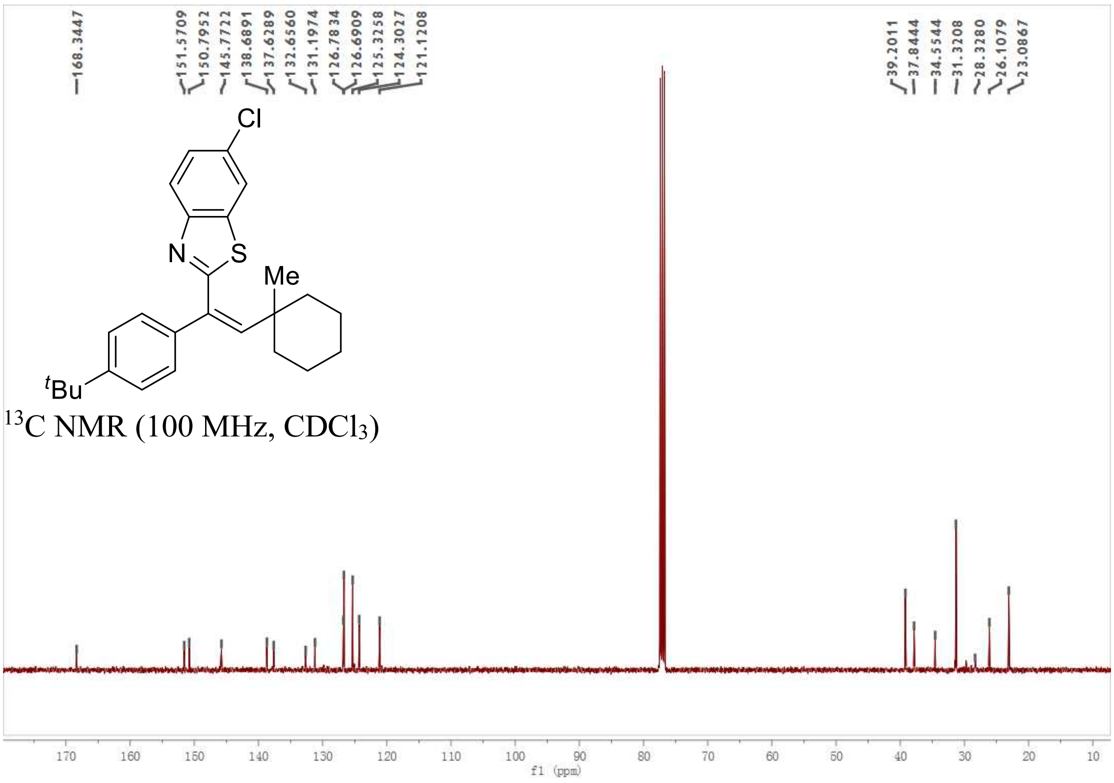

Supplementary Figure 183: ^{13}C NMR Spectra of (Z)-2-(1-(4-(tert-Butyl) phenyl)-2-(1-methylcyclohexyl)vinyl)-6-chlorobenzo[d] thiazole (54)

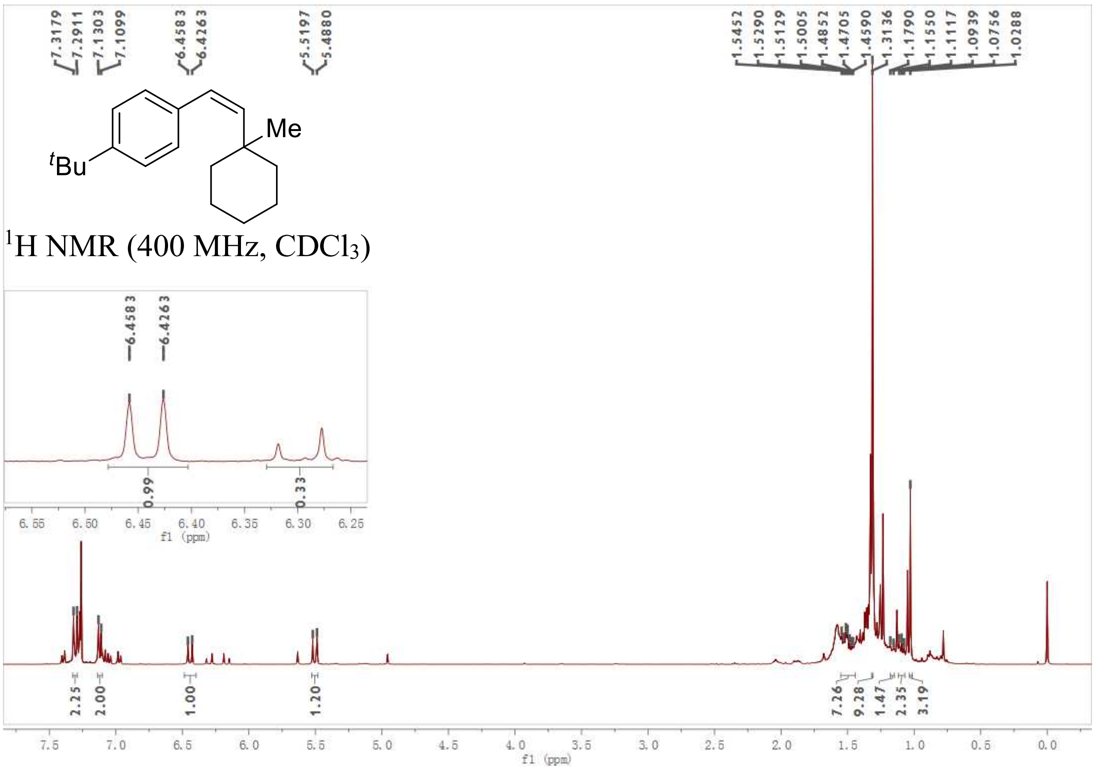

Supplementary Figure 184: ^{1}H NMR Spectra of (Z)-1-(tert-Butyl)-4-(2-(1-methylcyclohexyl)vinyl)benzene (55)

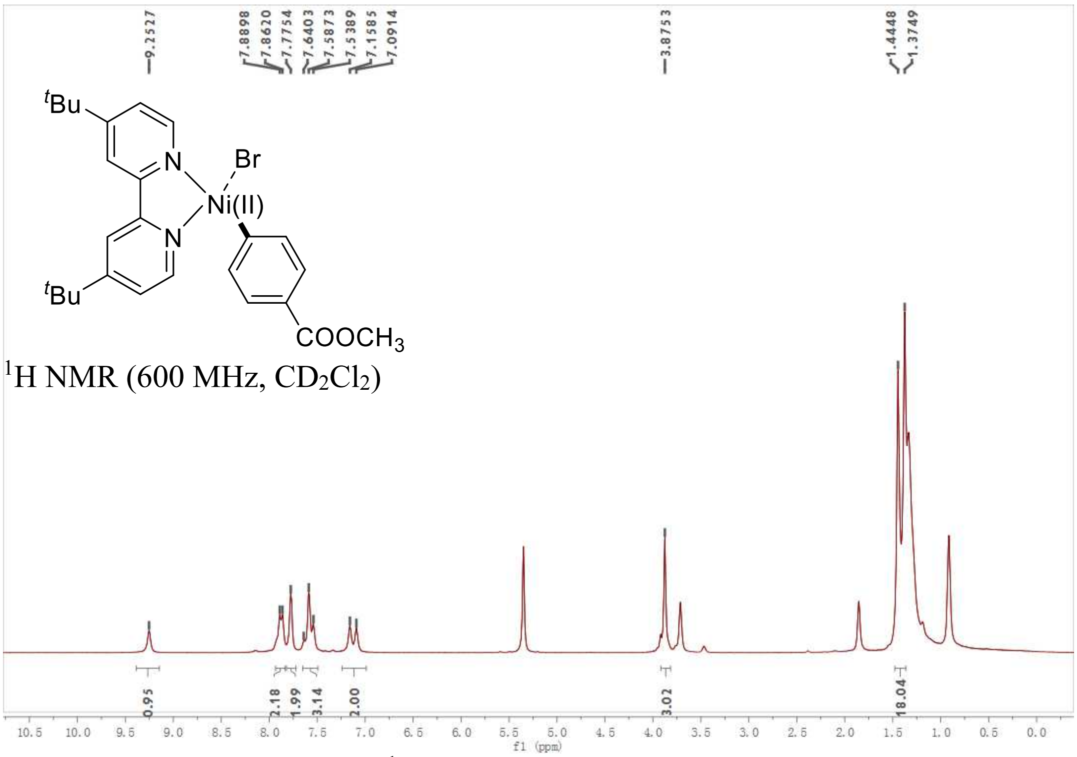

Supplementary Figure 185: ^{1}H NMR Spectra of Ni(II) complex 56

Supplementary References

(1) García-Domínguez, A.; Li, Z.; Nevado, C., Nickel-Catalyzed Reductive Dicarbofunctionalization of Alkenes. *J. Am. Chem. Soc.* **2017,** *139* (20), 6835-6838.

(2) Shang-Zheng, S.; Ruben, M., Nickel-Catalyzed Umpolung Arylation of Ambiphilic α-Bromoalkyl Boronic Esters. *Angew.Chem.Int. Ed.* **2018,** *57* (14), 3622-3625.
