# Supplementary material for: syn-Selective alkylarylation of terminal alkynes via the combination of photoredox and nickel catalysis
Source: Nat Commun. 2018 Oct 31;9:4543. doi: 10.1038/s41467-018-06904-9 (PMC6208420; doi:10.1038/s41467-018-06904-9)
Supplement: Supplementary file 3 — Description of Additional Supplementary Files [file 41467_2018_6904_MOESM3_ESM.pdf]

Description of Additional Supplementary Files

File Name: Supplementary Data 1
Description: Cartesian coordinates and energies
